# Taxonomic revision and cladistic analysis of *Avicularia* Lamarck, 1818 (Araneae, Theraphosidae, Aviculariinae) with description of three new aviculariine genera

**DOI:** 10.3897/zookeys.659.10717

**Published:** 2017-03-02

**Authors:** Caroline Sayuri Fukushima, Rogério Bertani

**Affiliations:** 1 Laboratório Especial de Ecologia e Evolução, Instituto Butantan, Av. Vital Brazil, 1500 CEP 05503-900, São Paulo, Brazil

**Keywords:** Birdspider, Linnaeus, morphology, Mygalomorphae, systematics, tarantula

## Abstract

The genus *Avicularia* Lamarck, 1818 is revised and all species are rediagnosed. The type species, described as *Aranea avicularia* Linnaeus, 1758, is the oldest mygalomorph species described and its taxonomic history is extensive and confusing. Cladistic analyses using both equal and implied weights were carried out with a matrix of 46 taxa from seven theraphosid subfamilies, and 71 morphological and ecological characters. The optimal cladogram found with Piwe and concavity = 6 suggests *Avicularia* and Aviculariinae are monophyletic. Subfamily Aviculariinae includes *Avicularia* Lamarck, 1818, *Typhochlaena* C. L. Koch, 1850, *Tapinauchenius* Ausserer, 1871, *Stromatopelma* Karsch, 1881, *Ephebopus* Simon, 1892, *Psalmopoeus* Pocock, 1895, *Heteroscodra* Pocock, 1899, *Iridopelma* Pocock, 1901, *Pachistopelma* Pocock, 1901, *Ybyrapora* **gen. n.**, *Caribena* **gen. n.**, and *Antillena* **gen. n.** The clade is supported by well-developed scopulae on tarsi and metatarsi, greatly extended laterally. *Avicularia* synapomorphies are juveniles bearing black tarsi contrasting with other lighter articles; spermathecae with an accentuated outwards curvature medially, and male palpal bulb with embolus medial portion and tegulum’s margin form an acute angle in retrolateral view. *Avicularia* is composed of twelve species, including three new species: *Avicularia avicularia* (Linnaeus, 1818), *Avicularia glauca* Simon, 1891, *Avicularia variegata* (F. O. Pickard-Cambridge, 1896) stat. n., *Avicularia minatrix* Pocock, 1903, *Avicularia taunayi* (Mello-Leitão, 1920), *Avicularia juruensis* Mello-Leitão, 1923, *Avicularia rufa* Schiapelli & Gerschman, 1945, *Avicularia purpurea* Kirk, 1990, *Avicularia hirschii* Bullmer et al. 2006, *Avicularia merianae* **sp. n.**, *Avicularia lynnae* **sp. n.**, and *Avicularia caei* **sp. n.**. *Avicularia* species are distributed throughout Mexico, Costa Rica, Panama, Trinidad and Tobago, Venezuela, Guyana, Suriname, French Guiana, Colombia, Ecuador, Peru, Bolivia, and Brazil. Three new genera are erected to accommodate former *Avicularia* species: *Caribena* **gen. n.**, composed of *Caribena laeta* (C. L. Koch, 1842), **comb. n.** and *Caribena versicolor* (Walckenaer, 1837), **comb. n.**; *Antillena* **gen. n.**, with a single species, *Antillena rickwesti* (Bertani & Huff, 2013), **comb. n.**, both from the Caribbean; and *Ybyrapora* **gen. n.**, composed of *Ybyrapora sooretama* (Bertani & Fukushima, 2009), **comb. n.**, *Ybyrapora gamba* (Bertani & Fukushima, 2009), **comb. n.** and *Ybyrapora diversipes* (C. L. Koch, 1842), **comb. n.** from Brazilian rainforest. The subspecies *Avicularia avicularia variegata* F. O. Pickard-Cambridge, 1896 is elevated to species status, resulting in the combination *Avicularia variegata* (F. O. Pickard-Cambridge, 1896) **stat. n.**. The following new synonymies are established: *Avicularia velutina* Simon 1889, *Avicularia exilis* Strand, 1907, *Avicularia ancylochyra* Mello-Leitão, 1923, *Avicularia cuminami* Mello-Leitão, 1930, and *Avicularia nigrotaeniata* Mello-Leitão, 1940 are junior synonyms of *Avicularia avicularia; Avicularia bicegoi* Mello-Leitão, 1923 is a junior synonym of *Avicularia variegata* **stat. n.**, and *Avicularia urticans* Schmidt, 1994 is a junior synonym of *Avicularia juruensis* Mello-Leitão, 1923. Species transferred to other genera: *Avicularia affinis* (Nicolet, 1849) is transferred to *Euathlus* Ausserer, 1875, making the new combination *Euathlus affinis* (Nicolet, 1849), **comb. n.**; *Avicularia subvulpina* Strand, 1906 is transferred to *Grammostola* Simon, 1892, making the new combination *Grammostola subvulpina* (Strand, 1906), **comb. n.**; *Avicularia aymara* (Chamberlin, 1916) is transferred to *Thrixopelma* Schmidt, 1994, making the new combination *Thrixopelma aymara* (Chamberlin, 1916), **comb. n.**; *Avicularia leporina* (C. L. Koch, 1841) and *Avicularia plantaris* (C. L. Koch, 1842) are transferred to *Iridopelma* Pocock, 1901, making the new combinations *Iridopelma leporina* (C. L. Koch, 1841), **comb. n.** and *Iridopelma plantaris* (C. L. Koch, 1842), **comb. n.**; the two last species are considered *nomina dubia*. The following species are considered *nomina dubia*: *Avicularia hirsutissima* (C. L. Koch, 1842) *nomen dubium*; *Ischnocolus hirsutum* Ausserer, 1875 *nomen dubium*; *Ischnocolus gracilis* Keyserling, 1891 *nomen dubium*; *Avicularia arabica* (Strand, 1908) *nomen dubium*; *Araneus hirtipes* (Fabricius, 1787) *nomen dubium*; *Avicularia ochracea* (Perty, 1833) *nomen dubium*; *Avicularia walckenaerii* (Perty, 1833) *nomen dubium*; *Avicularia testacea* (C. L. Koch, 1841) *nomen dubium*; *Avicularia detrita* (C. L. Koch, 1842) *nomen dubium*; *Ischnocolus doleschalli* Ausserer, 1871 *nomen dubium*; *Avicularia metallica* Ausserer, 1875 *nomen dubium*; *Avicularia rapax* (Ausserer, 1875) *nomen dubium*; *Avicularia holmbergi* Thorell, 1890 *nomen dubium*; *Avicularia aurantiaca* Bauer, 1996 *nomen dubium*; *Avicularia azuraklaasi* Tesmoingt, 1996 *nomen dubium*; *Avicularia huriana* Tesmoingt, 1996 *nomen dubium*; *Avicularia ulrichea* Tesmoingt, 1996 *nomen dubium*; *Avicularia braunshauseni* Tesmoingt, 1999 *nomen dubium*; *Avicularia geroldi* Tesmoingt, 1999 *nomen dubium*; *Avicularia soratae* Strand, 1907 *nomen dubium*; *Avicularia fasciculata* Strand, 1907 *nomen dubium*; *Avicularia fasciculata clara* Strand, 1907 *nomen dubium*; and *Avicularia surinamensis* Strand, 1907 *nomen dubium*. *Avicularia vestiaria* (De Geer, 1778) is considered *nomen nudum*. Keys are provided for identification of all aviculariine genera, as well as to *Avicularia*, *Caribena* **gen. n.**, *Ybyrapora* **gen. n.** and *Antillena* **gen. n.** species. Maps with records and information on species habitat are also presented. Lectotypes are newly designed for *Avicularia avicularia*, *Avicularia variegata* **stat. n.**, *Avicularia juruensis*, *Caribena laeta* **comb. n.**, *Euathlus affinis* **comb. n.** and a neotype is established for *Caribena versicolor* **comb. n.**

## Introduction

The genus *Avicularia* Lamarck, 1818 was erected for some species formerly included in *Mygale* Latreille, 1802. The type species, described as *Aranea avicularia* Linnaeus, 1758, was the first mygalomorph species described. Thus, its taxonomic history is extensive. It is also confusing; reflecting the knowledge and history of arachnology throughout the centuries.

The original description of *Avicularia* is vague (Lamarck 1818), resulting in confusion about which species should be included in the genus. Lamarck (1818) described *Avicularia*, among other characters, as large spiders, with eight eyes in St. André’s cross format (i. e. in an “X” shape), lacking rastellum on the chelicerae and tarsi with velvet scopulae. Lamarck (1818) also stated that these spiders can be found in cavities on the ground or in trees, and are mostly wandering animals, which do not build permanent retreats unlike the “*Mygale*” species. He included three species in the genus, in order: *Avicularia canceridea* (Latreille, 1806); *Avicularia blondii* (Latreille, 1804); and *Avicularia fasciata* (Latreille, 1804). Under the name *Avicularia canceridea* he listed *Aranea avicularia*, a species described by Linnaeus (1758) in *Systema Naturae*.

At that time, most mygalomorph spiders were described in the genus *Mygale* Latreille, 1802. The name *Mygale* (“les Mygales”, in French) is a non-scientific name used by Walckenaer (Bonnet 1957) to distinguish the “mineuses” and “aviculaires” spiders from the others (Walckenaer 1802). This name was subsequently used by Latreille (1802) to designate a spider genus, and the first species mentioned as an example of a *Mygale* species was *Aranea avicularia* Linnaeus, 1758. Olivier (1811) followed Walckenaer’s system, but he only considered in *Mygale* what he called the “mineuses” spiders. For the “aviculaire” spiders he used the name *Aranea*. Lamarck (1818) followed Olivier (1811), naming as *Mygale* only “*les Araignées mineuses*”; for “*le Araignées aviculaires*” he erected a new genus, *Avicularia*. Even after Lamarck (1818) erected *Avicularia*, many arachnologists continued to use the name *Mygale* when describing new *Avicularia* species. From 1833 to 1849, ten *Avicularia* species were originally described as *Mygale*: *Mygale walckenaerii* Perty, 1833; *Mygale ochracea* Perty, 1833; *Mygale versicolor* Walckenaer, 1837; *Mygale leporina* C. L. Koch, 1841; *Mygale caesia* C. L. Koch, 1842; *Mygale detrita* C. L. Koch, 1842; *Mygale diversipes* C. L. Koch, 1842; *Mygale laeta* C. L. Koch, 1842; *Mygale plantaris* C. L. Koch, 1842; and *Mygale affinis* Nicolet, 1849. However, the name *Mygale* was unavailable; it is a junior homonym of a mammal genus described by Cuvier (1800).

**Figures 1–2.**
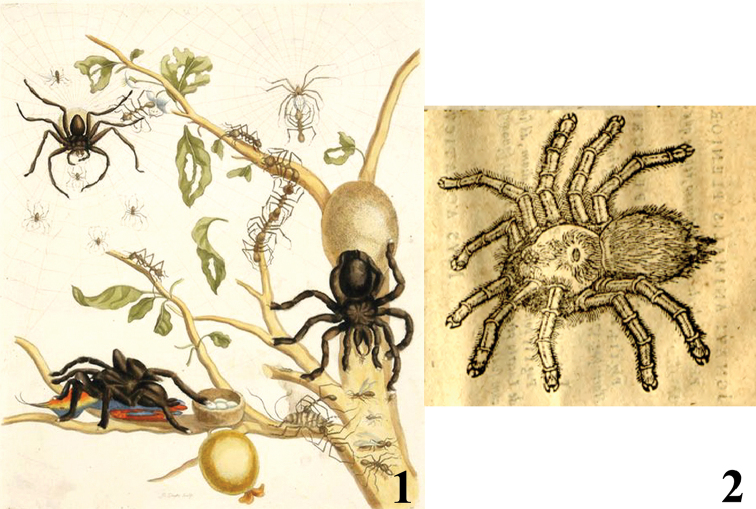
Historical aviculariine drawings. **1** Maria Sybilla Merian’s plate from *Metamorphosis insectorum Surinamensium* (1705) depicting an *Avicularia avicularia* eating a bird **2** Clusius’ (1611) drawing of a possible *Pachistopelma* sp., the oldest known illustration of a tarantula (Theraphosidae, Aviculariinae) in the New World.

About 50 years later the name *Avicularia* was used again (Ausserer 1871). Apparently, there was a gradual acceptance of this name together with the awareness that the name *Mygale* should not be used for spiders. The reluctance in accepting the name *Avicularia* proposed by Lamarck had cultural foundations. According to Smith (2000a), the name *Avicularia* is derived from the earlier usage of the word by Linnaeus (1758) when he described the species *Aranea avicularia*—from the latin *avicula*, or little bird—plus *aria*, meaning "which refers to" (Beechhold 1997 *apud* Smith 2000a). Thorell (1870) explained the genus name as a derivation of *avicularium*, meaning bird-keeper, but in the signification adopted, bird-catcher. With the name “*avicularia*” Linnaeus (1758) directly referenced the famous illustration made by Maria Sibylla Merian (1705) in *Metamorphosis Insectorum Surinamensium* (Smith 2000a), in which she drew a tarantula feeding on a bird (Fig. 1). The German word *Vogelspinne* is used to refer to spiders of the infraorder Mygalomorphae and translated literally as “bird spider”, which is likely also derived from Merian’s engraving. When Lamarck (1818) erected the new genus, he also mentioned that these spiders could feed on little birds on their nests, perhaps based on Merian’s illustration and explanations. The idea of a bird-eating spider was ridiculed by Langsdorff and others at the time, who considered it a regression to a science full of mythology and legends (Smith 2000a,b). However, now we know that Merian drew a real predation event.

Even though *Avicularia* was a nomenclatorally available and valid name, only in 1928 with the publication of Opinion 104 of the International Commission on Zoological Nomeclature (ICZN 1928) was the genus *Avicularia* included in the Official List of Generic Names. The Direction 67 of this same Commission (ICZN 1957) established that the specific name of the type species of *Avicularia* is *avicularia* Linnaeus, 1758 as published in the combination *Aranea avicularia*.

Some of the most renowned arachnologists have studied the genus throughout the 19^th^ Century and beginning of the 20^th^ Century. Ausserer (1875) described two new species, *Avicularia rutilans* Ausserer, 1875 from Colombia and *Avicularia metallica* Ausserer, 1875 from Suriname; Thorell (1890) described *Avicularia holmbergi* Thorell, 1890 from French Guiana; Simon (1889) described *Avicularia velutina* Simon, 1889 from Venezuela and few years afterwards, *Avicularia glauca* Simon, 1891 from Panama (Simon 1891). Some years afterwards, F. O. Pickard-Cambridge (1896) described *Avicularia avicularia variegata* F. O. Pickard-Cambridge, 1896 from Brazil; Pocock (1903) described *Avicularia minatrix* Pocock, 1903 from Venezuela; and Strand (1906, 1907abcd) described five more species and one subspecies of the genus—*Avicularia subvulpina* Strand, 1906, *Avicularia exilis* Strand, 1907, *Avicularia fasciculata* Strand, 1907, *Avicularia fasciculata clara* Strand, 1907 (all from “South America”); *Avicularia soratae* Strand, 1907 from Bolivia, and *Avicularia surinamensis* Strand, 1907 from Suriname.

Mello-Leitão studied the genus between 1920–1945. In the revision of Brazilian “theraphosoidea”, Mello-Leitão (1923) described three species: *Avicularia ancylochyra* Mello-Leitão, 1923; *Avicularia bicegoi* Mello-Leitão, 1923; and *Avicularia juruensis* Mello-Leitão, 1923. Some years later he described *Avicularia cuminami* Mello-Leitão, 1930 and *Avicularia pulchra* Mello-Leitão, 1933, both from Brazil; *Avicularia nigrotaeniata* Mello-Leitão 1940, from Guyana; and *Avicularia palmicola* Mello-Leitão, 1945 also from Brazil (Mello-Leitão 1930, 1933, 1940, 1945). Subsequently, Schiapelli and Gerschman (1945) described a new species from Brazil, *Avicularia rufa* Schiapelli & Gerschman, 1945. Until 1942, Roewer’s Catalog counted 29 species and two subspecies, which were distributed throughout Trinidad, Martinique and Guadeloupe Islands, USA, Puerto Rico, Panama, Bolivia, Suriname, Colombia and Brazil (Roewer 1942).

Despite the many authors who have studied the *Avicularia* species, only a few of them proposed a diagnosis for the genus. Ausserer (1871) was the first to offer a proposal, diagnosing *Avicularia* as having well-developed tarsal and metatarsal scopula and males with spines on the tibia (likely referring to male tibial apophysis). F. O. Pickard-Cambridge (1896) diagnosed *Avicularia* as having an “anterior row of eyes strongly procurved”, “legs of first pair shorter than those forth”, and “habits arboreal”. Simon (1903) characterized the genus as having posterior lateral eye smaller than anterior, anterior row of eyes strongly procurved, and males with tibial apophysis oblicuous, obtuse, with small spines. Mello-Leitão (1923) also published a diagnosis for the genus, but it is mainly a Portuguese translation of Simon’s (1903) diagnosis. Valerio (1979) diagnosed the genus as lacking stridulatory apparatus and males presenting medial spur on tibia I.

Over the course of many years, contributions to taxonomy of *Avicularia* were sparse. A significant alteration occurred only when Raven (1985) considered *Eurypelma* C. L. Koch, 1850 as a junior synonym of *Avicularia*. In fact, Thorell (1870) already had considered *Eurypelma* C. L. Koch as a junior synonym of *Avicularia*, but apparently this was overlooked by subsequent authors. *Eurypelma* was an old specious unrevised genus having about 50 species that were transferred to *Avicularia* at once, significantly raising the number of species in the genus.

However, Raven (1985) did not analyze all the *Eurypelma* species transferred, only the type species of the genus. Thus, the identity of the species transferred to *Avicularia* was not checked one by one and the taxonomic confusion was merely transferred and added from *Eurypelma* to *Avicularia*. As the general concept of *Eurypelma* was significantly distinct from *Avicularia* (since *Eurypelma* was considered formerly a grammostoline, now theraphosine, genus), it was clear that most of the species should not be included in *Avicularia*, and a series of transferences took place thereafter. Several species were transferred from *Avicularia* to *Aphonopelma* Pocock, 1901 (Schmidt 1993, Smith 1995), to *Brachypelma* Simon, 1891 (Smith 1993, 1986; Hancock and Hancock 1989) and to *Chromatopelma* Schmidt, 1995 (Schmidt 1995b). Despite this, several species formerly included in *Eurypelma* still remained in *Avicularia*: *Avicularia affinis* (Nicolet, 1849); *Avicularia alticeps* (Keyserling, 1878); *Avicularia anthracina* (C. L. Koch, 1842); *Avicularia aymara* (Chamberlin, 1916); *Avicularia borelli* (Simon, 1897); *Avicularia diversipes* (C. L. Koch, 1842); *Avicularia doleschalli* (Ausserer, 1871); *Avicularia gracilis* (Keyserling, 1891); *Avicularia guyana* (Simon, 1892); *Avicularia hirsuta* (Ausserer, 1875); *Avicularia obscura* (Ausserer, 1875); *Avicularia ochracea* (Perty, 1833); *Avicularia panamensis* (Simon, 1891); *Avicularia parva* (Keyserling, 1878); *Avicularia plantaris* (C. L. Koch, 1842); *Avicularia rapax* (Ausserer, 1875); and *Avicularia tigrina* (Pocock, 1903).

In this same work, Raven (1985) synonymized the genus *Ancylochirus* Mello-Leitão, 1920 with *Avicularia* and the single species *Ancylochirus taunayi* Mello-Leitão, 1920 was transferred, making the new combination *Avicularia taunayi* (Mello-Leitão, 1920).

Only in 1990 was a new species of *Avicularia* described: *Avicularia purpurea* Kirk, 1990, from Ecuador (Kirk 1990). Since then, many other species were described such as *Avicularia urticans* Schmidt, 1994, *Avicularia aurantiaca* Bauer, 1996, and *Avicularia azuraklaasi* Tesmoingt, 1996, all from Peru (Schmidt 1994; Bauer 1996; Tesmoingt 1996a); *Avicularia recifiensis* Struchen & Brändle, 1996 and *Avicularia ulrichea* Tesmoingt, 1996, both from Brazil (Struchen and Brändle 1996; Tesmoingt 1996b); *Avicularia huriana* Tesmoingt, 1996, from Ecuador (Tesmoingt 1996bc); *Avicularia braunshauseni* Tesmoingt, 1999 and *Avicularia geroldi* Tesmoingt, 1999, both also from Brazil (Tesmoingt 1999ab). At the same time, Lucas, Silva & Bertani (1992) transferred *Ephebopus violaceus* Mello-Leitão, 1930 to *Avicularia*, making the new combination *Avicularia violacea* (Mello-Leitão, 1930).

More recently, *Avicularia hirschii* Bullmer et al. 2006 was described from Ecuador (Bullmer et al. 2006); and two years after, *Avicularia violacea* (Mello-Leitão, 1930) was transferred to *Tapinauchenius* Ausserer, 1871 by West et al. (2008); *Avicularia obscura* (Ausserer, 1875) was transferred to *Ami* Pérez-Miles, 2008 (Pérez-Miles et al. 2008) and Gallon (2008) considered the monotypic genus *Avicuscodra* Strand, 1908 (Strand 1908) as a junior synonym of *Avicularia*, making the new combination *Avicularia arabica* (Strand, 1908). In 2009, *Avicularia borelli* (Simon, 1897) was transferred to *Grammostola* Simon, 1892 (Theraphosinae) (Gabriel 2009a); *Avicularia guyana* (Simon, 1892) was transferred to *Eupalaestrus* Pocock, 1901 (Gabriel 2009b) and *Avicularia panamensis* was transferred to *Sericopelma* Ausserer, 1875 (Gabriel 2009b). In this same year, two new species were described from the Atlantic Rainforest of Brazil (Bertani and Fukushima 2009): *Avicularia sooretama* Bertani & Fukushima, 2009 and *Avicularia gamba* Bertani & Fukushima, 2009. In 2011, after a study of all *Avicularia* species from Uruguay, *Avicularia anthracina* (C. L. Koch, 1842) was transferred to *Grammostola* Simon, 1892 and *Avicularia parva* was transferred to *Catumiri* Guadanucci, 2004 by Fukushima et. al (2011). The names *Pterinopelma tigrinum* Pocock, 1903 and *Ischnocolus alticeps* Keyserling, 1878, formerly belonging to *Avicularia*, were considered as *nomina dubia* (Fukushima et al. 2011).

The last taxonomic changes in the genus were proposed by Bertani (2012), after a revision of three Aviculariinae genera. The author considered *Avicularia pulchra* Mello-Leitão, 1933 and *Avicularia recifiensis* Struchen & Brändle, 1996 as junior synonyms of *Pachistopelma rufonigrum* Pocock, 1901, and *Avicularia palmicola* Mello-Leitão, 1945 as a junior synonym of *Iridopelma hirsutum* Pocock, 1901 (Bertani 2012).

The last *Avicularia* species described was *Avicularia rickwesti* Bertani & Huff, 2013, a very distinct species endemic of the Dominican Republic (Bertani and Huff 2013).

The most recent diagnosis for the genus was proposed by Gallon (2008). He considered *Avicularia* close to the genera *Pachistopelma* Pocock, 1901 and *Iridopelma* Pocock, 1901 based on the presence of urticating setae type II on the abdomen. According to Gallon (2008), the males of *Avicularia* can be distinguished from males of *Iridopelma* by lacking tibial apophysis on leg II and the females by the absence of lobes on spermathecae. Besides, both sexes of *Avicularia* would have had leg IV longer than leg I, the opposite of *Iridopelma*; and both sexes of *Avicularia* differ from *Pachistopelma* by having anterior row of eyes strongly procurved, not straight. Also, according to Gallon (2008), only *Avicularia*, *Pachistopelma* and *Iridopelma* would belong to the subfamily Aviculariinae. This idea was contradicted by West et al. (2008) and Bertani (2012), who presented cladistics analyses containing diverse related genera and proposed that subfamily Aviculariinae is more inclusive than proposed by Gallon (2008).

*Avicularia* species have an arboreal habit, making their silk retreats on vegetation and human structures (Stradling 1994). Studies with *Avicularia avicularia* pointed out that despite being sedentary, this species has a well-defined pattern of daily activities. During the day, the specimens remain inside their retreats while during the night they emerge to catch their prey (Stradling 1994).

An interesting defense mechanism was observed by Bertani and Marques (1996). They observed that the abdominal urticating setae type II (Cooke et al. 1972) are transferred by direct contact when the spiders are disturbed, and are not expulsed in the air as happens in theraphosines. Type II setae of *Avicularia* species is stouter than the slender setae of theraphosines, preventing them from being carried by the air (Bertani and Marques 1996). However, some years later, an interesting exception was seen, constituting a case of convergence between aviculariines and theraphosines. Bertani et al. (2003) observed that *Avicularia versicolor* (Walckenaer, 1837) expulse the urticating setae in a similar way of theraphosines. The type II urticating setae of this particular species are very slender, therefore, they can be carried by air.

Detailed studies on habit, life cycle, and reproduction as well as biogeography and conservation are practically nonexistent. In one of the few ecological studies, Stradling (1994) analyzed the distribution and behavioral ecology of *Avicularia avicularia* in Trinidad. He carried out this study in the field for 18 months, in an abandoned tonka bean plantation, resulting in the largest ecological dataset on *Avicularia avicularia* and one of the most detailed studies of aviculariine ecology. Stradling (1994) presented important conclusions about distribution, dispersion, courtship and mating behavior of this species.

A more recent report on the ecology of an *Avicularia* species was conducted by Maréchal et al. (2009). The authors studied the behavioral ecology and population structure of *Avicularia versicolor* over several years and suggested that this endemic species from Martinique should be included in the CITES’s list (The Convention on International Trade in Endangered Species of Wild Fauna and Flora).

Desco-Derouet and Gros (1972) studied weight increase and linear growth of *Avicularia avicularia* and concluded that changes in these variables cannot be used to determine sex in juveniles. Dresco-Derouet (1970) published a short note about the development cycle of *Avicularia avicularia* in captivity. Stradling (1978) also studied the growth and maturation of *Avicularia avicularia*, but he did it in both laboratory and field conditions, resulting in a more extensive and accurate work.

Regarding applied studies using the *Avicularia* species as a model, an antifungal peptide from the venom of the *Avicularia rufa* (misidentified as *Avicularia juruensis*) was discovered (Ayroza et al. 2012). This peptide, named juruin, lacks haemolytic activity on human erythrocytes at the antimicrobial concentrations and it is very similar to peptides found in *Selenocosmia* Ausserer, 1871, *Chilobrachys* Karsch, 1892, and *Haplopelma* Simon, 1892 species (Ayroza et al. 2012). Juruin has very potent activity against the majority of the fungal and yeast strains, which can be used for development of new drugs and antibiotics (Ayroza et al. 2012).

To date, the genus *Avicularia* includes 47 species and two subspecies and is recorded from Costa Rica, Panama, Colombia, Venezuela, Guyana, Suriname, French Guiana, Brazil, Ecuador, Peru, Bolivia, and Chile as well across the Caribbean archipelago including Cuba, Dominican Republic, Puerto Rico, Martinique and Guadeloupe, and Trinidad and Tobago (World Spider Catalog 2016).

Even though the genus has a large number of species and has historical importance, it remains unrevised. To date, only short, taxonomic works were published, with the exception of Bertani and Fukushima (2009), who discussed some aspects of behavior, distribution, and conservation of the *Avicularia* species. With 31 *nomina dubia* and three *nomina nuda* (World Spider Catalog 2016), in addition to the many understudied valid species, it is an urgent task to revise the genus. Thus, the aim of this work is to revise *Avicularia* and propose a cladistic hypothesis for the genus and its close relatives. Taking into account the historical taxonomic difficulties, poor informative descriptions allied to old or lost type material and the extreme morphological homogeneity of the species, the present work does not intend to solve all the taxonomic problems related to the genus but rather to give a foundation for future and more detailed studies.

An additional difficulty of this work is the existence of relatively few specimens housed in scientific collections and available for taxonomic research. *Avicularia* is one of the most popular genera in the pet trade, with thousands of specimens spread out all over the world. However, only few specimens from the pet trade are deposited in collections and these are usually lacking locality or other data, therefore restricting their use in taxonomic studies.

## Material and methods

Specimens from the following institutions were examined:

American Museum of Natural History, New York (AMNH); The Natural History Museum, London (BMNH); California Academy of Sciences, San Francisco (CAS); Museu do Departamento de Zoologia da Universidade de Brasília, Brasília (DZUB); Instituto Butantan, São Paulo (IBSP); Universidad Nacional de Colombia, Bogota (ICN–AR); Museu do Instituto Nacional de Pesquisas da Amazônia, Manaus (INPA); Museu Argentino de Ciencias Naturales “Bernardino Rivadavia”, Buenos Aires (MACN–AR); Museu de Ciências e Tecnologia da Pontifícia Universidade Católica do Rio Grande do Sul, Porto Alegre (MCP); Museum of Comparative Zoology, Harvard University, Cambridge (MCZ); Instituto de Zoologia, Universidad Central de Venezuela, Maracay (MIZA); Muséum National d’histoire Naturelle, Paris (MNHN–AR); Museu Nacional, Rio de Janeiro (MNRJ); Museu Paraense Emílio Goeldi, Belém (MPEG); Universidad Nacional Mayor de San Marcos, Lima (MUSM–ENT); Museum Wiesbaden (Naturwissenschaftliche Sammlung), Wiesbaden (MWNH); Museu de Zoologia da Universidade de São Paulo, São Paulo (MZUSP); Forschungsinstitut und Naturmuseum Senckenberg, Frankfurt (SMF); Museo de Entomologia Klaus Raven Büller, Universidad Nacional Agraria La Molina, Lima (UA); Museum of Evolution, University of Uppsala, Uppsala (UUZM); Museum für Naturkunde der Humboldt-Universität, Berlin (ZMB); and Museu de Zoologia da Universidade Estadual de Campinas, Campinas (ZUEC).

The AMNH and CAS collections do not assign numbers to specimens or sets. Thus, in order to individualize and facilitate localization of the specimens by subsequent researchers, we gave informal codes and numbers after Museum acronyms.

The general description format follows Raven (2005), with modifications, mainly of setae and thrichobothria patterns, which were not, herein, studied in detail. All the measurements are in millimeters (mm). A stereomicroscope Leica M205C and a microscope Leica DM2500, both with a DFC 450 camera attached, combined with Leica LAS Montage and LAS 3D modules, were used to obtain images and measurements of small body parts and urticating setae, respectively. Large body parts as leg articles and carapace were measured with a digital Mitutoyo callipter with an error of 0.005, rounded up to two significant decimals. The measurements of legs and palps were taken on the dorsal aspect of the left side, unless appendages were lost, damaged, or obviously regenerated. Structures of the left side of the specimens were chosen for descriptions. When using structures of the right side, the figures were mirrored to show them as of the left side and allow for easy comparison. A Scanning Electron Microscope JEOL JSM840A from Laboratório de Microscopia Eletrônica of Instituto de Física of Universidade de São Paulo (USP) was used to obtain microphotographs. Male palpal bulb terminology follows Bertani (2000). Urticating setae terminology follows Cooke et al. (1972). Geographical coordinates: primary sources are between round brackets, and secondary sources obtained with Google Earth™, between square brackets. The coordinates from the secondary source were obtained from the center of the municipality cited in the specimen label and are in DMS (Degrees, Minutes, and Seconds), with format rounded off to minutes. Maps were made with SimpleMappr, an online tool to produce maps (Shorthouse 2010). Abbreviations: ALE = anterior lateral eye; AME = anterior median eye; CI = consistence index; ITC = inferior tarsal claw; L = length; PLE = posterior lateral eye; PLS = posterior lateral spinneret; PME = posterior median eye; PMS = posterior median spinneret; RI = retention index; and STC = superior tarsal claws. Missing leg articles are indicated by “–” in descriptions.

### Cladistic analysis

A data matrix with 46 taxa and 71 characters (Table 1) was analyzed with two programs using equal (Nona 2.0 for Windows (Goloboff 1998)) and implied character weighting (Piwe 3.0 for Windows (Goloboff 1997)). For Piwe, concavities from 1 to 6 were used. All characters were treated as unordered. Commands used to search for trees were h10000, h/1000, amb-, mult*500. Absolute and relative Bremer supports (Bremer 1994) were estimated in Piwe using the following command: h1000, find*, bsupport50, bsupport*.

**Table 1.** Data matrix showing the distribution of character states in cladistic analysis. (?= unknown, - = non-applicable; both codification treated as missing data).

**Taxon \ Character**	**0**	**1**	**2**	**3**	**4**	**5**	**6**	**7**	**8**	**9**	**10**	**11**	**12**	**13**	**14**	**15**	**16**	**17**	**18**	**19**	**20**	**21**	**22**	**23**	**24**	**25**	**26**	**27**	**28**	**29**	**30**	**31**	**32**	**33**	**34**	**35**
*Melloina santuario*	0	0	0	0	0	0	0	0	0	-	0	0	0	0	0	0	0	0	0	0	0	0	0	0	0	-	0	0	0	0	0	0	0	0	0	0
*Holothele rondoni*	0	0	0	0	0	0	0	0	1	0	0	0	0	0	0	0	0	0	0	0	0	0	0	0	0	-	0	0	0	0	0	0	0	1	0	0
*Pterinochilus* sp.	0	1	0	0	0	0	0	0	1	1	1	0	0	0	0	1	0	0	0	0	0	1	0	0	1	-	0	0	0	0	0	0	0	0	0	0
*Pelinobius muticus*	0	1	0	0	0	1	0	1	1	1	1	0	1	1	0	0	0	0	1	0	0	1	0	0	0	-	0	0	0	0	0	0	0	1	0	0
*Haplopelma* sp.	0	1	0	0	0	1	0	0	1	1	1	0	0	0	0	1	1	0	0	0	0	1	0	0	1	-	0	0	0	0	0	0	2	-	-	-
*Phlogiellus* sp.	1	0	0	2	0	1	0	0	1	1	0	0	1	0	0	0	0	1	0	0	0	1	0	0	0	-	0	0	0	0	0	0	0	0	0	0
*Lasiodora* sp.	0	1	0	0	0	0	0	0	1	1	1	0	0	0	1	0	0	0	1	0	0	1	0	0	0	-	0	0	0	0	0	0	1	0	-	-
*Phrixotrichus vulpinus*	0	0	0	1	0	0	0	0	1	1	1	0	0	0	0	0	0	0	0	0	0	1	0	0	3	-	0	0	0	0	0	0	0	1	-	-
*Poecilotheria* sp.	0	0	0	0	0	0	0	0	1	2	1	1	-	0	0	0	1	1	0	0	0	1	0	0	2	-	0	0	0	0	0	0	2	-	-	-
*Encyocratella olivacea*	0	0	1	1	0	0	0	0	1	1	1	0	0	1	0	0	0	0	0	0	0	0	0	0	1	-	0	0	0	0	0	1	-	-	-	-
*Stromatopelma* sp.	1	0	1	0	0	0	1	0	1	2	1	1	-	0	0	0	0	0	0	0	0	1	1	0	1	-	0	0	0	0	0	0	0	0	0	0
*Heteroscodra maculata*	1	0	1	0	0	0	1	0	1	2	1	1	-	0	0	0	0	0	0	0	0	1	1	0	1	-	0	0	0	0	0	0	0	0	0	0
*Psalmopoeus* sp.	1	0	0	0	0	0	1	0	1	2	1	0	1	0	0	0	0	1	0	0	0	1	0	1	4	0	0	1	0	0	0	0	0	1	0	0
*Tapinauchenius* sp.	1	0	0	0	0	0	1	0	1	2	1	0	1	0	0	0	0	0	0	0	0	1	0	1	4	0	0	1	0	0	0	0	0	0	0	0
*Ephebopus murinus*	1	0	0	0	0	0	0	0	1	2	1	0	1	0	0	0	0	0	0	0	1	1	0	1	0	-	0	1	0	0	0	0	0	0	0	0
*Ephebopus uatuman*	1	0	0	0	0	0	0	0	1	2	1	0	1	0	0	0	0	0	0	0	0	2	0	1	0	-	0	1	0	0	0	0	0	0	0	0
*Tapinauchenius seladonia*	0	0	0	0	1	0	0	0	1	2	1	0	1	0	0	0	0	0	0	1	0	0	0	0	4	0	1	0	0	0	0	0	0	0	0	1
*Typhochlaena amma*	0	0	0	0	1	0	1	0	1	2	1	1	-	0	0	0	0	0	0	1	0	1	0	?	?	?	?	?	0	0	0	0	0	0	1	0
*Typhochlaena costae*	0	0	0	0	1	0	0	0	1	2	1	1	-	0	0	0	0	0	0	1	0	1	0	0	4	0	0	0	0	0	0	0	0	0	0	0
*Typhochlaena curumim*	0	0	0	0	1	0	1	0	1	2	1	1	-	0	0	0	0	0	0	1	0	1	0	0	4	2	0	1	0	0	0	0	0	1	0	1
*Typhochlaena paschoali*	0	?	0	0	1	0	?	0	1	2	?	0	1	0	0	0	0	0	0	1	0	1	0	0	5	-	0	1	?	0	0	0	0	1	0	0
*Pachistopelma rufonigrum*	1	0	0	0	0	0	0	0	1	2	1	1	-	0	0	0	0	0	0	0	0	0	0	1	4	2	1	0	0	0	0	0	0	0	0	0
*Pachistopelma bromelicola*	1	0	0	0	0	0	0	0	1	2	1	1	-	0	0	0	0	0	0	0	0	0	0	1	4	0	1	0	0	0	0	0	0	0	0	0
*Iridopelma hirsutum*	0	0	0	0	0	0	0	0	1	2	1	1	-	0	0	0	0	0	0	0	0	1	0	1	5	-	1	0	1	0	0	0	0	1	0	1
*Iridopelma zorodes*	0	0	0	0	0	0	0	0	1	2	1	1	-	0	0	0	0	0	0	0	0	1	0	1	5	-	1	0	0	0	0	0	0	0	0	0
*Iridopelma vanini*	0	0	0	0	0	0	0	0	1	2	1	1	-	0	0	0	0	0	0	0	0	1	0	?	?	-	?	?	0	0	0	0	0	1	0	1
*Iridopelma katiae*	0	0	0	0	0	0	0	0	1	2	1	1	-	0	0	0	0	0	0	0	0	0	0	1	5	-	0	1	0	0	0	0	0	1	0	0
*Iridopelma oliveirai*	0	0	0	0	0	0	0	0	1	2	1	1	-	0	0	0	0	0	0	0	0	1	0	1	5	-	?	?	0	0	0	0	0	1	0	1
*Iridopelma marcoi*	0	?	0	0	0	0	?	0	1	2	?	1	-	0	0	0	0	0	0	0	0	1	0	?	?	-	?	?	?	0	0	0	0	1	0	1
*Caribena laeta* comb. n.	0	0	0	0	0	0	0	0	1	2	1	1	-	0	0	0	0	0	0	0	0	1	0	1	4	2	1	0	0	0	0	0	0	0	0	0
*Caribena versicolor* comb. n.	0	0	0	0	0	0	0	0	1	2	1	1	-	0	0	0	0	0	0	0	0	0	0	1	4	0	1	0	0	0	0	0	0	0	0	0
*Ybyrapora sooretama* comb. n.	0	0	0	0	0	?	0	0	1	2	1	1	-	0	0	0	0	0	0	0	0	1	0	1	6	-	1	0	1	0	0	0	0	1	0	0
*Ybyrapora gamba* comb. n.	0	0	0	0	0	?	0	0	1	2	1	1	-	0	0	0	0	0	0	0	0	1	0	1	6	-	1	0	1	0	0	0	0	0	0	0
*Ybyrapora diversipes* comb. n.	0	0	0	0	0	0	0	0	1	2	1	1	-	0	0	0	0	0	0	0	0	2	0	1	6	-	1	0	0	0	0	0	0	0	0	0
*Avicularia rickwesti* comb. n.	0	0	0	0	0	0	0	0	1	2	1	1	-	0	0	0	0	0	0	0	0	1	0	0	5	-	0	0	0	0	0	0	0	0	-	-
*Avicularia avicularia*	0	0	0	0	0	0	0	0	1	2	1	1	-	0	0	0	0	0	0	0	0	?	0	1	4	2	0	1	0	1	?	0	0	0	0	0
*Avicularia variegata* stat. n.	0	0	0	0	0	0	0	0	1	2	1	1	-	0	0	0	0	0	0	0	0	?	0	1	4	2	0	1	0	1	1	0	0	0	1	0
*Avicularia taunayi*	0	0	0	0	0	0	0	0	1	2	1	1	-	0	0	0	0	0	0	0	0	1	0	1	4	1	0	1	0	0	0	0	0	1	1	0
*Avicularia juruensis*	0	0	0	0	0	0	0	0	1	2	1	1	-	0	0	0	0	0	0	0	0	?	0	1	4	2	0	1	0	?	?	0	0	0	1	0
*Avicularia rufa*	0	0	0	0	0	0	0	0	1	2	1	1	-	0	0	0	0	0	0	0	0	2	0	1	4	2	0	1	0	1	1	0	0	0	0	0
*Avicularia purpurea*	0	0	0	0	0	0	0	0	1	2	1	1	-	0	0	0	0	0	0	0	0	1	0	1	4	0	1	0	0	0	0	0	0	0	0	0
*Avicularia merianae* sp. n.	0	0	0	0	0	0	0	0	1	2	1	1	-	0	0	0	0	0	0	0	0	1	0	1	4	1	0	1	0	0	0	0	0	0	0	0
*Avicularia hirschii*	0	0	0	0	0	0	0	0	1	2	1	1	-	0	0	0	0	0	0	0	0	1	0	1	7	-	0	1	?	0	0	0	0	0	0	1
*Avicularia minatrix*	0	0	0	0	0	0	0	0	1	2	1	1	-	0	0	0	0	0	0	0	0	1	0	0	4	0	0	1	0	0	0	0	0	0	0	0
*Avicularia lynnae* sp. n.	0	0	0	0	0	0	0	0	1	2	1	1	-	0	0	0	0	0	0	0	0	1	0	?	?	?	?	?	1	?	0	?	?	?	?	?
*Avicularia caei* sp. n.	0	0	0	0	0	0	0	0	1	2	1	1	-	0	0	0	0	0	0	0	0	1	0	?	?	?	?	?	0	?	0	?	?	?	?	?
**Taxon \ Character**	**36**	**37**	**38**	**39**	**40**	**41**	**42**	**43**	**44**	**45**	**46**	**47**	**48**	**49**	**50**	**51**	**52**	**53**	**54**	**55**	**56**	**57**	**58**	**59**	**60**	**61**	**62**	**63**	**64**	**65**	**66**	**67**	**68**	**69**	**70**	
*Melloina santuario*	?	0	0	-	-	0	0	0	0	-	2	0	0	0	0	-	0	0	0	0	0	0	-	0	0	0	-	-	0	0	0	0	0	0	-	
*Holothele rondoni*	0	0	0	-	-	0	1	0	1	0	0	0	0	0	0	-	0	0	0	0	0	0	-	0	0	0	-	-	0	0	0	0	0	3	-	
*Pterinochilus* sp.	0	0	0	-	-	1	0	0	0	-	0	0	0	0	0	-	0	0	0	0	0	1	-	0	0	0	-	-	0	0	0	2	1	3	-	
*Pelinobius muticus*	0	0	0	-	-	1	0	0	1	0	0	0	0	0	1	2	0	0	0	0	1	-	-	0	0	0	-	-	0	0	0	0	0	3	-	
*Haplopelma* sp.	0	-	0	-	-	1	1	1	0	-	0	1	2	0	1	0	1	1	1	1	0	2	1	0	0	0	-	-	0	0	0	1	1	3	-	
*Phlogiellus* sp.	0	0	0	-	-	1	0	0	1	1	0	0	0	0	0	-	0	0	0	0	1	-	-	0	0	0	-	-	0	0	0	1	1	3	-	
*Lasiodora* sp.	0	-	0	-	-	0	1	1	0	-	1	1	1	0	0	-	1	1	1	1	0	0	-	0	1	0	-	-	1	0	0	1	1	3	-	
*Phrixotrichus vulpinus*	0	-	0	-	-	0	1	1	0	-	0	0	0	0	0	-	1	1	0	0	0	0	-	0	0	0	-	-	1	1	0	2	2	3	-	
*Poecilotheria* sp.	0	-	0	-	-	1	0	0	0	-	1	1	0	0	0	-	1	1	1	0	1	-	-	0	0	0	-	-	0	0	0	1	1	1	0	
*Encyocratela olivacea*	-	-	0	-	-	1	0	0	?	?	0	1	2	0	0	-	1	1	0	0	1	-	-	0	0	0	-	-	0	0	0	1	1	1	?	
*Stromatopelma* sp.	0	0	0	-	-	1	0	0	0	-	0	0	0	0	0	-	0	0	0	0	1	-	-	0	0	0	-	-	0	0	0	1	1	1	0	
*Heteroscodra maculata*	0	0	0	-	-	1	0	0	0	-	0	0	0	0	0	-	0	0	0	0	1	-	-	0	0	0	-	-	0	0	0	1	0	1	0	
*Psalmopoeus* sp.	0	0	0	-	-	1	0	0	1	1	2	0	0	0	0	-	0	0	0	0	0	0	-	0	0	0	-	-	0	0	0	1	1	2	-	
*Tapinauchenius* sp.	0	0	0	-	-	1	0	0	0	-	2	0	0	0	0	-	0	0	0	0	0	0	-	0	0	0	-	-	0	0	0	1	1	2	-	
*Ephebopus murinus*	0	0	0	-	-	1	0	0	1	1	0	0	0	0	0	-	0	0	0	0	0	0	-	0	0	0	-	-	0	0	1	2	2	3	-	
*Ephebopus uatuman*	0	0	0	-	-	1	0	0	1	1	2	0	0	0	0	-	0	0	0	0	0	0	-	0	0	0	-	-	0	0	1	1	1	3	-	
*Typhochlaena seladonia*	?	0	0	-	-	1	0	0	1	0	3	0	0	0	2	1	0	0	0	0	1	-	-	0	0	1	0	0	0	0	0	1	1	1	2	
*Typhochlaena amma*	0	1	0	-	-	1	0	0	0	-	0	0	0	0	2	1	0	0	0	0	1	-	-	0	0	1	0	0	0	0	0	1	1	1	?	
*Typhochlaena costae*	0	0	0	-	-	1	1	0	0	-	0	0	0	0	1	2	0	0	0	0	1	-	-	0	0	1	0	0	0	0	0	1	1	1	?	
*Typhochlaena curumim*	0	0	0	-	-	1	0	0	0	-	0	0	0	0	1	2	0	0	0	0	1	-	-	0	0	1	0	0	0	0	0	1	0	1	2	
*Typhochlaena paschoali*	0	0	?	?	?	?	?	?	?	?	?	?	?	?	?	?	?	?	?	?	?	?	?	?	0	1	0	0	0	0	0	?	1	?	?	
*Pachistopelma rufonigrum*	0	0	1	0	0	1	0	0	0	-	2	0	0	0	2	0	0	0	0	0	0	2	0	0	0	1	1	0	0	0	0	0	0	1	3	
*Pachistopelma bromelicola*	0	0	1	0	0	1	0	0	0	-	2	0	0	0	2	0	0	0	0	0	0	2	0	0	0	1	1	0	0	0	0	0	0	1	3	
*Iridopelma hirsutum*	0	0	1	1	0	1	0	0	0	-	0	0	0	0	2	2	0	0	0	0	0	2	0	1	0	1	0	0	0	0	0	2	1	1	1	
*Iridopelma zorodes*	0	0	1	1	0	1	0	0	0	-	0	0	0	0	2	2	0	0	0	0	0	2	0	1	0	1	0	0	0	0	0	1	1	1	1	
*Iridopelma vanini*	0	0	1	1	0	1	0	0	0	-	2	0	0	0	2	2	0	0	0	0	0	2	0	1	0	1	0	0	0	0	0	1	1	1	?	
*Iridopelma katiae*	0	0	1	1	0	1	0	0	0	-	2	0	0	0	2	2	0	0	0	0	0	2	0	1	0	1	0	0	0	0	0	1	1	1	3	
*Iridopelma oliveirai*	0	0	1	1	0	1	0	0	0	-	0	0	0	0	2	2	0	0	0	0	0	2	0	1	0	1	0	0	0	0	0	2	1	1	?	
*Iridopelma marcoi*	0	0	?	?	?	?	?	?	?	?	?	?	?	?	?	?	?	?	?	?	?	?	?	?	0	?	1	-	0	0	0	?	1	1	?	
*Caribena laeta* comb. n.	0	0	1	0	1	1	0	0	0	-	2	0	0	0	1	2	0	0	0	0	0	2	0	0	0	1	0	1	0	0	0	1	1	1	0	
*Caribena versicolor* comb. n.	0	1	1	0	1	1	0	0	1	1	2	0	0	0	2	1	0	0	0	0	0	2	0	0	0	1	0	1	0	0	0	1	1	1	0	
*Ybyrapora sooretama* comb. n.	2	1	0	-	-	1	0	0	1	1	2	0	0	1	2	2	0	0	0	0	1	-	-	0	0	1	0	0	0	0	0	1	0	1	1	
*Ybyrapora gamba* comb. n.	2	1	0	-	-	1	0	0	0	-	2	0	0	0	1	2	0	0	0	0	1	-	-	0	0	1	0	0	0	0	0	1	1	1	1	
*Ybyrapora diversipes* comb. n.	2	1	1	1	0	1	0	0	0	-	3	0	0	0	2	0	0	0	0	0	1	-	-	0	0	1	0	0	0	0	0	1	1	1	1	
*Avicularia rickwesti* comb. n.	0	-	1	1	0	1	0	0	0	-	1	0	2	0	1	2	1	1	1	0	0	2	1	0	0	1	0	0	0	0	0	1	1	1	0	
*Avicularia avicularia*	0	1	1	1	0	1	0	0	1	1	2	0	0	1	2	1	0	0	0	0	0	2	1	0	0	1	0	0	0	0	0	0	0	1	0	
*Avicularia variegata* stat. n.	0	1	1	1	0	1	0	0	1	2	2	0	0	1	2	1	0	0	0	0	0	2	1	0	0	1	0	0	0	0	0	1	1	1	0	
*Avicularia taunayi*	0	1	1	1	0	1	0	0	1	1	2	0	0	1	2	1	0	0	0	0	0	2	1	0	0	1	0	0	0	0	0	1	1	1	0	
*Avicularia juruensis*	0	1	1	1	0	1	0	0	1	2	2	0	0	1	2	1	0	0	0	0	0	2	1	0	0	1	0	0	0	0	0	1	1	1	0	
*Avicularia rufa*	0	1	1	1	0	1	0	0	1	1	2	0	0	1	2	1	0	0	0	0	0	2	1	0	0	1	0	0	0	0	0	0	0	1	0	
**Taxon \ Character**	**36**	**37**	**38**	**39**	**40**	**41**	**42**	**43**	**44**	**45**	**46**	**47**	**48**	**49**	**50**	**51**	**52**	**53**	**54**	**55**	**56**	**57**	**58**	**59**	**60**	**61**	**62**	**63**	**64**	**65**	**66**	**67**	**68**	**69**	**70**	
*Avicularia purpurea*	1	1	0	-	-	1	0	0	1	1	2	0	0	0	2	1	0	0	0	0	0	2	1	0	0	1	0	0	0	0	0	1	1	1	0	
*Avicularia merianae* sp. n.	1	1	0	-	-	1	0	0	1	1	2	0	0	0	2	1	0	0	0	0	0	2	1	0	0	1	0	0	0	0	0	1	1	1	?	
*Avicularia hirschii*	0	1	1	0	0	1	0	0	1	1	2	0	0	0	2	1	0	0	0	0	1	-	-	0	0	1	0	0	0	0	0	1	0	1	0	
*Avicularia minatrix*	0	1	0	-	-	1	0	0	0	-	2	0	0	1	2	1	0	0	0	0	1	-	-	0	0	1	0	0	0	0	0	1	1	1	0	
*Avicularia lynnae* sp. n.	?	?	1	0	0	1	0	0	1	1	3	0	0	0	2	1	0	0	0	0	1	-	-	0	0	1	?	0	0	0	0	1	?	1	?	
*Avicularia caei* sp. n.	?	?	1	0	0	1	0	0	1	0	3	0	0	0	2	1	0	0	0	0	1	-	-	0	0	1	?	0	0	0	0	1	?	?	?	

In the cladistic analyses, representative species were included of seven of the eight theraphosid subfamilies recognized by Raven (1985), as well as species of all aviculariine genera and other available arboreal taxa with uncertain position, e.g., *Poecilotheria* Simon, 1885, *Heteroscodra* Pocock, 1899, and *Stromatopelma* Karsch, 1881, in order to test Aviculariinae monophyly. *Melloina santuario* Bertani, 2013 (Paratropididae) was used to root the cladogram. The choice of this genus was done based on two considerations: paratropidids were at the beginning of this study considered as the sister-group of Theraphosidae in morphological analysis (Raven 1985), and *Melloina* is the paratropidid genus which retains more plesiomorphic characters shared with the Theraphosidae (Raven 1985). However, recent molecular analyses indicate Barychelidae as the sister-group of Theraphosidae (Bond et al. 2012, Hamilton et al. 2016, Garrison et al. 2016, Wheeler et al. 2016), so future studies should aim to include more taxa based on molecular analyses. The ingroup specimens used in cladistics analysis are those used in the descriptions. Specimens used as outgroups are: **Paratropididae**, Glabropelmatinae: *Melloina santuario* Bertani, 2013, holotype male (MIZA 520) and paratype female (MNRJ 12965), both from Venezuela, Lara, Cueva El Santuario [9°49'N, 70°03'W], O. Villarreal col., 19 April 2000. **Theraphosidae**, Aviculariinae: *Ephebopus murinus* (Walckenaer, 1837), male (IBSP 9650) and female (IBSP 9658), Brazil, state of Pará, Tucuruí, U.H.E. Tucuruí, [4°20'S, 49°31'W], Equipe de Resgate de Fauna col., 1984; *Ephebopus uatuman* Lucas, Silva & Bertani, 1992, male holotype (IBSP 4939) and paratype female (IBSP 4940), Brazil, state of Amazonas, Presidente Figueiredo [2°02'S, 60°01'W], Uatuman River, Balbina Hydroelectric Power Station, M. Costa col., 19 February 1988; *Heteroscodra maculata* Pocock, 1899, male, Africa, pet trade (IBSP 9642); female, Guinea-Bissau, pet trade (IBSP 9644); *Iridopelma hirsutum* Pocock, 1901, male, Brazil, state of Paraíba, João Pessoa [7°07'S, 34°52'W], P. F. L. Duarte col., 30 November 1979 (IBSP 8078); female, Brazil, state of Alagoas, Murici, E. E. Murici, UFAL (09°14'1.73"S, 35°50'1.61"W), R. Bertani, D. R. M. Ortega and R. H. Nagahama col., 16 August 2006 (MNRJ 06252); *Iridopelma katiae* Bertani, 2012, holotype female, Brazil, state of Bahia, Mucugê, Parque Nacional da Chapada Diamantina (12°45'7.00"S, 41°30'2.18"W), 1399 m a.s.l., R. Bertani, C. S. Fukushima and R. H. Nagahama col., 18 February 2008 (MZUSP 36887) and paratype male, same data, (12°45'3.77"S, 41°30'4.01"W), 1377 m a.s.l., R. Bertani, C. S. Fukushima and R. H. Nagahama col., 17 February 2008 (MZUSP 36888); *Iridopelma marcoi* Bertani, 2012, holotype female, Brazil, state of Bahia, São Desidério (12°28'52"S, 45°09'10"W), 724 m a.s.l, M. A. Freitas col., October 2009, under tree bark, 1 m above the ground, area of carrasco vegetation (MZUSP 36891); *Iridopelma oliveirai* Bertani, 2012, holotype male, Brazil, state of Bahia, Central, Toca dos Pilões [11°08'S, 42°06'W], A. D. Brescovit col., July 2000 (IBSP 10100) and paratype female, at night in “Macambira” bromeliad (*Bromelia laciniosa*), same data (IBSP 8714); *Iridopelma vanini* Bertani, 2012, holotype female, Brazil, state of Piauí, Parnaíba [2°53'S, 41°41'W], 5 m a.s.l., R. Bertani col., November 1994 (IBSP Ref. 74.595) and paratype male, Brazil, state of Maranhão, Barreirinhas, Parque Nacional dos Lençóis Maranhenses [2°41'S, 42°55'W), 32 m a.s.l., Equipe Biota col., 12–18 October 2001 (IBSP 11328); *Iridopelma zorodes* (Mello-Leitão, 1926), male, Brazil, state of Bahia, Mata de São João, RPPN Camurujipe, in a leaf at night, R. Bertani, C. S. Fukushima and R. H. Nagahama col., 04 October 2007 (MNRJ 6254); female, Brazil, state of Sergipe, Areia Branca, Mosqueiro, I. Matos col., 01 November 1992, ref. 79901 (IBSP 11760); *Pachistopelma bromelicola* Bertani, 2012, holotype male, Brazil, state of Bahia, Elísio Medrado, RPPN Jequitibá (12°52'3.20"S, 39°28'9.09"W), R. Bertani, C. S. Fukushima and R. H. Nagahama col., 07 October 2007, collected at night, found immature inside bromeliads, matured in captivity in May 2010 (MNRJ 06241) and paratype female, same data (MNRJ 06242); *Pachistopelma rufonigrum* Pocock, 1901, male and female, Brazil, state of Alagoas, Murici, Estação Ecológica de Murici (9°14'9.52"S, 35°48'0.25"W), 245 m a.s.l., R. Bertani, R. H. Nagahama and D. R. M. Ortega col., 11 August 2006 (MNRJ 6246 AL1100); *Psalmopoeus cambridgei* Pocock, 1895, male, Trinidad-Tobago, pet trade (IBSP 9653); *Psalmopoeus* sp., female, Venezuela, pet trade (IBSP 9655); *Stromatopelma* sp., male, Sierra Leone, pet trade (IBSP 9665); female, Africa, pet trade (IBSP 11136); *Tapinauchenius violaceus* (Mello-Leitão, 1930), holotype female of *Tapinauchenius purpureus* Schmidt, 1995 (SMF 38042) and paratype male of *Tapinauchenius purpureus* Schmidt, 1995, French Guiana (SMF 38046); *Typhochlaena amma* Bertani, 2012, holotype female, Brazil, state of Espírito Santo, Santa Teresa, Estação Ecológica de Santa Lúcia [19°58'S, 40°32'W], 672 m a.s.l, A. P. L. Giupponi col., February 2008 (MNRJ 06239) and paratype male, same locality, no further data (MNRJ 12926); *Typhochlaena costae* Bertani, 2012, holotype female, Brazil, state of Tocantins, Palmas, U.H.E. Luís Eduardo Magalhães [10°12'S, 48°24'W], 211 m a.s.l, during faunal rescuing, M. Costa and D. Cândido col., 12 January 2002 (IBSP unnumbered) and paratype male, Brazil, Tocantins, Lajeado (9°46'4.85"S, 48°21'6.69"W), 226 m a.s.l, G. Puorto, R. Martins and I. Knysak col. (pitfall trap), April 2002 (IBSP unnumbered); *Typhochlaena curumim* Bertani, 2012, holotype female, Brazil, state of Paraíba, Areia, Reserva Ecológica Estadual Mata do Pau-Ferro [6°58'S, 35°42'W], 500 m a.s.l., under tree bark, A. D. Brescovit, R. Bertani, A. B. Bonaldo and S. C. Dias col., September 1999 (IBSP 8701) and paratype female (IBSP 8354), same data; male, Brazil, state of Rio Grande do Norte, Baía Formosa, lying over a leaf in a bush at night, S. N. Migliore col., 19 April 2014, S90 (MNRJ 06915); *Typhochlaena paschoali* Bertani, 2012, holotype female and immature paratype, Brazil, state of Bahia, Camacan [15°24'S, 39°30'W], no further data (MNRJ 13723); paratypes 1 female, 1 subadult male, 9 immatures, Brazil, state of Bahia, Jussari [15°10'S, 39°29'W], no further data (MNRJ 12928 – R2981); *Typhochlaena seladonia* (C. L. Koch, 1841), 1 male, 1 female exuvium, Brazil, state of Bahia, Camaçari, Jacuipe [12°42'S, 38°07'W], T. Brazil ded., August 1980 (IBSP 4551); 1 female, Brazil, state of Bahia, Salvador, Alphaville [12°56'S, 38°21'W], G. G. Montingelli col., 11 December 2001 (IBSP 109718); Eumenophorinae: *Pelinobius muticus* Karsch, 1885, male (IBSP 8530), female (IBSP 9643), both from Kenya, born in captivity; Harpactirinae: *Pterinochilus* sp., male, Angola, Biula-Dala [11°10'S, 20°12'E] (IBSP 9647); female, Africa, pet trade (IBSP 8765); Ischnocolinae: *Holothele rondoni* (Lucas & Bücherl, 1972), holotype male and paratype female, Brazil, state of Amazonas, Iauaretê [0°36'N, 69°11'W] (IBSP 4090); Ornithoctoninae: *Haplopelma longipes* von Wirth & Striffler, 2005, male, Cambodia, Skuon [1°33'N, 104°55'E], A. Anderson col., 10 July 2003 (MZUSP 28761); *Haplopelma minax* (Thorell, 1897), female, Thailand, 1 mi east of Bangkok [13°43'N, 100°31'E] (IBSP 9645); Selenocosmiinae: *Phlogiellus* sp, male, Vietnam, R. Blauman col., April 1989 (MZUSP 28762); female (AMNH), pet trade; *Poecilotheria* sp., male, India, pet trade (IBSP 9660); *Poecilotheria ornata* Pocock, 1899, female, Sri Lanka, pet trade (IBSP 8767); Theraphosinae: *Phrixotrichus vulpinus* (Karsch, 1880), 3 males, Chile (IBSP 3817–A); 1 female, Chile, Osorno [40°34'S, 73°09'W] (IBSP 3817–B); *Lasiodora* sp., male, Brazil, state of Paraíba, João Pessoa [7°07'S, 34°52'W] (IBSP 11143); female, Brazil, state of Pernambuco, Jaboatão dos Guararapes [8°06'S, 35°00'W], Conjunto Murebeca (IBSP 10293); Subfamily *Incertae sedis*: *Encyocratella olivacea* Strand, 1907, holotype female, Tanzania, Amani (05°09'S, 38°36'E), Vosseler leg., November 1903 (ZMB 10484); male, Tanzania, mountains near Arusha (03°23'S, 36°40'E) wild caught, reared to maturity, 2004 (BMNH); female holotype and 2 females paratypes of *Xenodendrophila gabrieli* Gallon, 2003, northern Tanzania, mountains near Arusha (03°23'S, 36°40'E), Joe Beraducci leg., circa April 1999 (BMNH 2005.123).

In the cladistic analyses we used the characters proposed by West et al. (2008), Bertani (2012), and 15 new characters. Forty characters (0–3, 5–18, 20–22, 31–33, 41, 43, 47–48, 52–57, 60–61, 64–66, and 69–70) are the same used by West et al. (2008) or were slightly modified here. Ten characters (4, 19, 23, 26, 35, 37–38, 59, 67 and 68) are strictly the same used by Bertani (2012). Characters 24, 44–46 and 50 were adapted from that work. The newly introduced characters are 25, 27–30, 34, 36, 39–40, 42, 49, 51, 58, 62–63. In characters marked with an asterisk (*, viz 7–8, 14, 20, 31, 60 and 65) fit and steps are not given as the character is not significant to the analyses but were kept to indicate autapomorphies for terminal taxa.

Character coding is always challenging, especially regarding problems concerning “part” coding (e.g., presence of tibial apophysis) versus “character-variable” coding (e.g., development of tibial apophysis). We decided to code “part” characters separately from its “character-variable” characters to preserve transformational independence between them, assuring that primary homology statements of both characters were included in the analyses (Lee and Bryant 1999). Fusing coding results in a loss of phylogenetic information and also in the determination of taxa synapomorphies by others characters (Lee and Bryant 1999). The characters that were splited are: 8/9, 11/12, 24/25, 31/32, 44/45, 56/57/58, 61/62, and 61/63.

Theraphosids are notorious for their morphological homogeneity (Bertani 2001). Due to this, it is extremely difficult to find characters suitable for taxonomy and cladistics. In order to find new characters, we have tried many approaches. We took measurements of leg and palp articles such as tibia, metatarsi and femora, and established ratios between them and ratios between one of these articles and the carapace. We also calculated the ratio between the length of tibial apophysis and tibia. Unfortunately, all results were not significant enough to be used in the analyses. Measurements of male palpal bulb, such as the ratio between the length of embolus and carapace, showed the same problems. Besides this, the morphology of the Aviculariinae male palpal bulb is a problem *per se*. It is very difficult to position the specimen in order to make reliable and replicable embolus measurements.

Another feature we have tried to use is the length of type II urticating setae. Some species, such as *Avicularia hirschii*, *Avicularia minatrix*, *Avicularia taunayi*, *Ybyrapora sooretama* comb. n. and *Ybyrapora gamba* comb. n., apparently have unusual urticating setae length. However, we could not confirm this suspicion since we have just one or two specimens of those species. Thus, it was impossible to determine the intraspecific length variation and establish a reliable length range for each species. Beside setae length, another urticating setae feature that could be used is the distribution of barbs along setae in females (Figs 16–17). Usually, females present barbs only in a short area near hair stalk (Stradling 1978). In contrast, morphotype 5 of *Avicularia avicularia* presents barbs along 1/3 of the seta length (Fig. 17). However, since we have only three immature females, it is not possible to state if that is a typical feature of the morphotype population.

Thus, after trying different attempts and approaches, we consider the following characters suitable to be used:

**0. Anterior row of eyes: (0) procurved (Fig. 13), (1) straight (Fig. 12).**

**1. Clypeus: (0) absent or narrow; (1) present, wide.**

West et al. (2008) and Bertani (2012) used three states for this character: “absent”, “narrow” and “wide”. Herein, we consider that the states “absent” and “narrow” should be merged due to difficulty in establishing limits between them. The state “wide”, on the other hand, is easily distinguishable.

**2. Fovea, closure: (0) slit like; (1) pit like.**

**3. Labial cuspules, number: (0) 30**–**300; (1) 0**–**20; (2) 350**–**450.**

**4. Sternum, shape: (0) longer than wide, not truncated behind (Fig. 9); (1) as long as wide, truncated behind (Fig. 7).**

**5. Sigilla, posterior pair, position: (0) marginal, less than 1.5 diameters from margin; (1) close to the center, more than 2 diameters from margin.**

**6. Setae on metatarsi and tibia I**–**IV, length, males: (0) same length as other articles (Fig. 299); (1) longer setae laterally projected, forming a brush (Fig. 301).**

7. Scopulae on metatarsi IV, division: (0) divided by setae or spiniform setae; (1) not divided.*

8. Tarsal scopulae, occurrence: (0) no true scopula; (1) true scopula.*

**9. Tarsal scopulae, setae development: (0) scopula composed of sparse setae; (1) dense scopula that does not extend much laterally; (2) scopula very extensive laterally, giving the tarsi and metatarsi I and II a spatulate appearance (Figs 299–301).**

**10. Tarsi IV, division, males: (0) cracked; (1) integral.**

**11. Leg spines, occurrence: (0) present; (1) absent.**

**12. Leg spines, distribution: (0) in whole tibiae and/or metatarsi; (1) only in ventral apical tibiae and/or metatarsi.**

**13. Palpal femora, scopula on retrolateral face, occurrence: (0) absent, (1) present.**

14. Femora IV, scopulae on retrolateral face, occurrence: (0) absent; (1) present.*

**15. Chelicerae, scopulae on retrolateral face, occurrence: (0) absent, (1) present.**

**16. Maxillae, spiniform setae on lower prolateral face: (0) absent, (1) present.**

**17. Stridulatory bristles form maxillae lyra: (0) absent, (1) present (Fig. 11).**

**18. Stridulatory bristles on coxae I, occurrence: (0) absent, (1) present.**

**19. Posterior lateral spinnerets, distal article, shape: (0) digitiform (Fig. 10); (1) domed (Fig. 8).**

20. Patellae and tibia, stripes, color: (0) same color of the rest of the segment, (1) white.*

**21. Leg rings on distal femora, tibiae and metatarsi, coloration: (0) same color of the rest of the segment, (1) white (Fig. 299), (2), yellow or orange (Fig. 300).**

*Avicularia avicularia* and *Avicularia variegata* stat. n. have morphotypes with different colors of leg rings. Due to this intraespecific variability, these two taxa were coded as “?” in the matrix.

**22. Tibiae, metatarsi and tarsi, dorsal coloration: (0) homogeneous color, (1) with black marking (Fig. 5).**

**23. Color pattern, ontogenetic change: (0) pattern remains practically the same during ontogeny; (1) pattern presents drastic changes during ontogeny.**

Most outgroup species have only slight variations in the general color and abdominal pattern during their ontogeny. We consider a drastic change when legs, carapace, and abdomen change their color during ontogeny. Most aviculariines have abdomen dorsum pattern with stripes in their early life stages, losing it when reaching maturity, which is, herein, considered as state 1. Other species, such as *Ephebopus murinus*, despite not having heterogeneous abdominal pattern during all ontogeny, have some conspicuous changes: immatures have light brown legs with black tarsi and black carapace, while adults have black legs with very conspicuous white stripes and light brown carapace. This condition is also considered as a drastic change and codified as “1”.

**24. Dorsal abdominal pattern in immatures (see figures in Bertani 2012): (0) homogeneous; (1) herringbone; (2) with a zigzag central longitudinal dark stripe over a clear spot, which is marginated in black and connects to five narrow transversal black stripes; (3) two median dorso-lateral spots; (4) central longitudinal black stripe with 5**–**6 lateral stripes, connecting or not with the central stripe (Fig. 37); (5) leaf pattern: black with a large central clear area having a longitudinal dark stripe, area closer to longitudinal dark stripe usually reddish; (6) central longitudinal reddish stripe inside a dark area with zigzag borders connected to transverse dark stripes (Fig. 295**); **(7) longitudinal central stripe of a different color of remaining abdomen (Fig. 184).**

Character modified from Bertani (2012). A new state was added (state 7), which was applied here only to *Avicularia hirschii*. State 4 is present in most *Avicularia* species. This abdominal stripped pattern is also spread over the subfamily. It can also be found in *Caribena* gen. n. species, in both *Pachistopelma* species, in *Typhochlaena curumim, Typhochlaena costae* and *Typhochlaena seladonia* as well as *Tapinauchenius* sp. and *Psalmopoeus* sp.

**25. Abdominal pattern, immatures, central longitudinal stripe, connection with lateral stripes: (0) connected with all lateral stripes (Fig. 167); (1) connected only with first and second lateral pair of stripes (Fig. 101); (2) disconnected from lateral stripes (Fig. 44).**

In species with abdominal pattern corresponding to state 4 of character 24, the central longitudinal black stripe can connect or not with the lateral stripes. State 0 is found in many Aviculariinae species such as *Avicularia purpurea* and *Avicularia minatrix* as well as in *Caribena versicolor* comb. n., *Typhochlaena costae*, *Typhochlaena seladonia*, *Pachistopelma bromelicola*, *Tapinauchenius* sp. and *Psalmopoeus* sp. State 1 is found only in two *Avicularia* species from the early stages: *Avicularia merianae* sp. n. and *Avicularia taunayi*. Central stripe disconnected from lateral stripes (state 2) can be found in *Avicularia rufa*, *Avicularia juruensis*, *Avicularia variegata* stat. n. and *Avicularia avicularia*, as well as in *Caribena laeta* comb. n., *Typhochlaena curumim* and in *Pachistopelma rufonigrum*.

**26. Body coloration, juveniles: (0) brownish or grayish (Fig. 44); (1) metallic green or blue (Fig. 239).**

**27. Tarsi, coloration, juveniles: (0) same color of other articles (Fig. 167); (1) black (Fig. 44).**

Black tarsi contrasting with other lighter articles in legs and palpi are present in juveniles of some *Tapinauchenius* and *Ephebopus* species. It also occurs in all *Avicularia* species with known immature stages (except *Avicularia purpurea*) as well as in *Iridopelma katiae* and *Typhochlaena paschoali*. Since *Psalmopoeus* sp. has black tarsi contrasting with clear metatarsi, it was codified as state 1 despite most articles having a dark color. *Typhochlaena curumim* has black tarsi and metatarsi; but as the other articles are lighter and there is a strong contrast between them, it is considered as having state 1.

**28. Dorsal abdominal pattern, single dorsal stripe, male, occurrence: (0) absent (Fig. 43); (1) present (Fig. 297).**

Males of *Ybyrapora sooretama* comb. n., *Ybyrapora gamba* comb. n.,

*Avicularia lynnae* sp. n., and *Iridopelma hirsutum* have a single dorsal central stripe on abdomen dorsum. This pattern is very distinct from those found in females of these same species and also very distinct from patterns commonly found in other aviculariine males. Patterns other than dorsal central stripe were all codified as state 0. *Avicularia hirschii* is coded as “?” since the holotype, the single known male, has the abdomen in poor condition. The description also does not allow us to recognize its abdominal pattern.

**29. Abdominal setae, distribution, females: (0) homogeneous (Fig. 304); (1) heterogeneous, with long guard-setae grouped on lateral and dorso anterior areas (Figs 302–303).**

Presence of long guard-setae grouped on lateral and dorsal anterior areas of the abdomen is a characteristic found only in females of *Avicularia avicularia*, *Avicularia rufa*, *Avicularia juruensis* and *Avicularia variegata* stat. n. These longer guard-setae have different coloration from the shorter body setae found on remaining abdominal areas. In males, the distribution pattern is homogeneous, with long guard-setae spread all over the dorsal abdomen.

This character is coded “?” in *Avicularia juruensis* since each morphotype has a different type of abdominal setae pattern.

**30. Legs and palp, long guard-setae, coloration: (0) setae with homogeneous coloration or gradually lightening along its length (Fig. 299); (1) setae with darker base and contrasting whitish apex (grizzled setae) (Fig. 300).**

Theraphosid spiders have two main covering setae types in legs and pedipalps; the short setae that densely covers most of the article area, providing a velvety aspect, is known as short body setae (Foelix 2010). The other type is the very long tactile setae, which presents insertion into a socket, allowing them to move, and is more spaced out among each other; this is known as guard-setae (Foelix 2010). These long setae are normally homogeneously dark or present a gradual lightening toward apex. But, in some species, these setae are dark and present a contrasting whitish apex.

All examined outgroup taxa have setae with homogeneous coloration along its length. In the ingroup, some species such as *Avicularia avicularia*, *Avicularia rufa*, *Avicularia juruensis* and *Avicularia variegata* stat. n. have the long setae with a contrasting whitish apex, giving a grizzled body aspect (Fig. 300). However, this character is coded “?” in *Avicularia juruensis* and *Avicularia avicularia* since some morphotypes (morphotypes 1 and 3 for *Avicularia juruensis* and morphotypes 1, 2, 4, and 5 for *Avicularia avicularia*) have homogeneous coloration along most of the guard-setae.

31. Spermathecae, occurrence: (0) present; (1) absent.*

**32. Spermathecae, number: (0) two, completely separated (Fig. 14), (1), two, fused at base; (2) one, totally fused.**

**33. Spermathecae, walls, shape: (0) without projections or lobes (Fig. 21); (1) with projections or lobes (Fig. 91).**

Of the several specimens of *Avicularia rufa*, *Avicularia juruensis*, *Avicularia avicularia*, and *Avicularia variegata* stat. n. examined, a few specimens have one or more discrete lobes in the spermathecae. We consider them an extreme morphological variation, as the typical spermathecae of those species lack any type of lobes.

*Caribena laeta* comb. n. spermathecae have a slight intumescence in their apex (Figs 227–229). It is not a well-defined lobe as in other taxa, such as *Ybyrapora sooretama* comb. n. (Fig. 257) or in *Avicularia taunayi* (Figs 91–92). Due to this, we considered *Caribena laeta* comb. n. as having state 0. State 1 is widespread among theraphosid genera.

**34. Spermathecae, midwidth: (0) as wide as its base, or midwidth more slender than the base width but wider than apex width (Fig. 21**); **(1) midwidth expanded, about 1.5 times its basal and apical portion widths, or wider (Fig. 54).**

We consider the spermathecae with midwidth expanded when this region is 1.5 times or more wider than both apex and base widths. Spermathecae of *Avicularia variegata* stat. n. (Fig. 54) and *Avicularia juruensis* (Fig. 106) clearly have state 1. In other aviculariine species, it is also possible to recognize this pattern despite their unusual spermathecae morphology, as in spermathecae with lobes in *Avicularia taunyai* (Fig. 91). Spermathecae midwidth of *Ybyrapora sooretama* comb. n., *Ybyrapora gamba* comb. n. and *Avicularia merianae* sp. n. are slightly wider than their bases’ width, but not enough to consider them as state 1.

In other taxa such as *Haplopelma* sp., *Poecilotheria* sp., *Lasiodora* sp., *Phrixotrichus vulpinus* and *Avicularia rickwesti* comb. n., spermathecae (Fig. 284) are very modified or completely fused. In these cases we consider the character inapplicable.

**35. Spermathecae, shape: (0) non-spiraled or not-twisted (Fig. 21); (1) twisted or spiraled (Fig. 183).**

**36. Spermathecae, weakly-sclerotized area, size: (0) weakly-developed or shorter than half the length of the well-sclerotized area (Fig. 21); (1) twice the length or the same length of the well-sclerotized area (Fig. 154); (2) spermatheca virtually non-sclerotized (Figs 257–259).**

In some species, the spermathecae have a weakly-sclerotized basal portion, lighter and softer than the more sclerotized distal area, and without large visible pores. As this weakly-sclerotized area is barely present in most outgroup species, we consider them as having state 0. State 1 is found only in *Avicularia purpurea* (Fig. 154) and *Avicularia merianae* sp. n. (Fig. 196). All three species of *Ybyrapora* gen. n. have spermathecae with a very small sclerotization degree, even in adult females (Figs 257–259), and they were coded as state 2. *Melloina santuario* and *Typhochlaena seladonia* have spermathecae virtually non-sclerotized, very similar to *Ybyrapora* gen. n. spermathecae. However, we know a single female of each species, both very small in size, in which the non-sclerotized spermathecae condition may be due to a not fully mature life stage. Thus, we decided to code both species in matrix as “?”.

**37. Spermathecae, curvature: (0) straight or almost so, curved inward (Fig. 14); (1) with an accentuated outwards curvature medially (Fig. 21).**

**38. Cymbium, process in retrolateral lobe, occurrence: (0) absent or very weakly-developed (Figs 162, 305); (1) present, well-developed (Figs 306–307).**

The presence of a process on the cymbium was considered by Raven (1985) a synapomorphy of Aviculariinae. However, more recent analyses showed that the structure is lacking in many Aviculariinae genera (West et al. 2008, Bertani 2012). Despite this, the character is informative and was studied in detail, herein. The process is a projection on the apical area of cymbium retrolateral lobes (Figs 306–307) and shows a considerable level of interspecific variation in size, shape, and associated setae. If on one hand this variation provides more information for cladistic analysis, on the other hand sometimes is difficult to establish limits for the states. We consider the process well-developed only when there is a clear and well-defined projection in the cymbium lobes (Figs 306–307). If there is a small bump without clear delimitation on cymbium retrolateral area, it was considered a very weakly-developed process, or state 0 (Figs 162, 305). We consider that *Pachistopelma* spp., *Iridopelma* spp., *Caribena* gen. n. spp., *Ybyrapora diversipes* comb. n., *Avicularia rickwesti* comb. n., *Avicularia avicularia, Avicularia rufa, Avicularia juruensis*, *Avicularia variegata* stat. n. and *Avicularia taunayi* have well-developed process (state 1).

**39. Cymbium, setae covering the retrolateral process, thickness: (0) thin (Fig. 306); (1) thick (Fig. 307).**

Thin setae covering the retrolateral process are found both in some *Avicularia* species as *Avicularia hirschii*, *Avicularia lynnae* sp. n. and *Avicularia caei* sp. n., as well as in species of *Caribena* gen. n. and in *Pachistopelma* species.

**40**. **Cymbium, process in retrolateral lobe, shape: (0) rounded (Fig. 307); (1) sharp (Fig. 306).**

The apex of the process on retrolateral lobe is usually rounded, except in both species of *Caribena* gen. n., in which the process apex is sharp.

**41. Cymbium, prolateral lobe, shape: (0) rounded; (1) subtriangular.**

**42. Tegulum, format: (0) globous (Fig. 29); (1) piriform (Fig. 320).**

We detected two distinct tegula shapes. The globous tegulum is short, narrowing abruptly and giving origin to a slender embolus since its beginning. The pyriform tegulum is longer and tapers to form the embolus.

All Aviculariinae possess globous tegulum, except *Typhochlaena costae*.

**43. Subtegulum, length: (0) small; (1) large, extending down the bulb for half of the tegulum.**

**44. Palpal bulb, prominence on tegulum (frontal view), occurrence: (0) absent (Fig. 312); (1) present (Figs 313–315).**

The tegula of some species have a groove in its prolateral side, which forms a prominence near it (Bertani 2012). This prominence can be better seen in frontal view (Figs 313–315). Presence of a prominence is shared by all *Avicularia* species except *Avicularia minatrix* and also in species of *Holothele*, *Pelinobius*, *Phlogiellus*, *Ephebopus*, and *Psalmopoeus*. In other aviculariine species this groove is lacking and the tegulum prolateral area is rounded. This state (0) is found in species of *Iridopelma, Pachistopelma*, *Typhochlaena* (except *Typhochlaena seladonia)*, *Ybyrapora* gen. n. (except in *Ybyrapora sooretama* comb. n.), *Caribena laeta* comb. n. and *Avicularia rickwesti* comb. n. *Encyocratela olivacea* is coded as “?” since this character was included after the specimen examination and we could not re-examine it in the proper position.

**45. Palpal bulb, prominence on tegulum, development: (0) weakly-developed (Fig. 313); (1) developed (Fig. 314); (2) well-developed (Fig. 315).**

We found that prominence on tegulum can have different development degrees. There are species in which prominence is well-developed (state 2), with a deep groove, as seen in *Avicularia variegata* stat. n. and in *Avicularia juruensis* (Fig. 315). When there is a distinct groove and it is clearly possible to determine a tegulum prominence, though it is not well-developed, it was coded as state 1. This state is found in most species analyzed (Fig. 314). However, if the groove is discrete, hard to see, and consequently the prominence is weakly-developed (Fig. 313), it was coded as state 0, as in *Holothele rondoni*, *Pelinobius muticus*, *Typhochlaena seladonia* and *Avicularia caei* sp. n.

**Figure 3.**
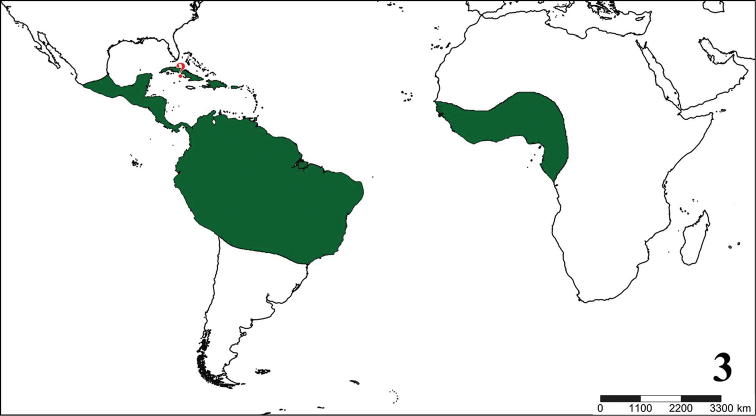
Approximate distribution area of Aviculariinae. ? = dubious record.

**46. Embolus, length, retrolateral view: (0) 1.5 to 2.5 times the tegulum’s length; (1) smaller than tegulum’s length (Fig. 320); (2) 3.0 to 3.5 times the tegulum’s length (Fig. 323); (3) more than 4 times the tegulum’s length (Fig. 277).**

Most Aviculariinae have emboli from 3 to 3.5 times the tegulum’s length (state 2), including all *Avicularia* species, except *Avicularia lynnae* sp. n. and *Avicularia caei* sp. n., which have emboli described as very long (state 3).

**47. Embolus, distal width: (0) thin, shorter than 1/5 tegulum’s length (Fig. 323); (1) thick, more than 1/3 tegulum’s length (Fig. 320).**

**48 Embolus, shape: (0) not flattened (Fig. 323); (1) slightly flattened (Fig. 320); (2) very flattened (Fig. 286).**

**49. Embolus, tip: (0) tapers (Figs 318–319); (1) narrows abruptly (Figs 316–317).**

In some Aviculariinae taxa such as *Avicularia minatrix*, *Avicularia taunayi*, *Avicularia rufa*, *Avicularia juruensis*, *Avicularia avicularia*, *Avicularia variegata* stat. n. and *Ybyrapora sooretama* comb. n., the embolus tip narrows abruptly near 1/5 distal portion (better visualized in dorsal view). The remaining taxa show a gradual embolus tapering.

**50. Embolus, proximal part in frontal view, shape: (0) straight; (1) slightly curved (Fig. 233); (2) very curved (Fig. 30).**

Most outgroups (except *Haplopelma* sp. and *Pelinobius muticus*) and some Aviculariinae, such as *Psalmopoeus* sp., *Tapinauchenius* sp., *Ephebopus* spp., *Stromatopelma* sp. and *Heteroscodra* sp., have straight proximal portion of embolus in frontal view. All the other Aviculariinae taxa have some curvature degree in proximal portion of embolus. *Caribena laeta* comb. n. (Fig. 233), *Ybyrapora gamba* comb. n., *Typhochlaena costae*, *Typhochlaena curumim* and *Avicularia rickwesti* comb. n. have slightly curved proximal embolus. The other Aviculariinae taxa have very curved proximal embolus (state 2) (Fig. 30).

**51. Embolus, curved, retrolateral view, angle between tegulum’s margin and embolus medial portion: (0) very acute angle (Fig. 321) (1) acute angle (Fig. 323) (2) right or obtuse angle (Fig. 322).**

We found that the angle between tegulum’s margin and embolus medial portion in retrolateral view can be informative for cladistic analysis. In *Haplopelma* sp., *Pachistopelma* spp. and *Ybyrapora diversipes* comb. n., the angle formed is very acute (state 0) (Fig. 321). In other taxa, such as *Typhochlaena costae*, *Typhochlaena curumim*, *Iridopelma* spp., *Ybyrapora* gen. n. (except *Ybyrapora diversipes* comb. n.), *Avicularia rickwesti* comb. n., *Caribena laeta* comb. n. and *Pelinobius muticus* sp., an obtuse angle is formed between embolus medial portion and tegulum’s margin (state 2) (Fig. 322). In the remaining aviculariine species, including all *Avicularia*, an acute angle is formed between these two structures (state 1) (Fig. 323).

**52. Bulb, prolateral inferior keel on embolus: (0) absent; (1) present.**

**53. Bulb, prolateral superior keel on embolus: (0) absent;(1) present.**

**54. Bulb, apical keel on embolus: (0) absent;(1) present**.

**55. Bulb, retrolateral keel on embolus: (0) absent; (1) present.**

**56. Tibial apophysis on leg I, occurrence: (0) present (Figs 310–311); (1) absent or weakly-developed (Figs 308–309).**

Specimens of *Ybyrapora sooretama* comb. n., *Ybyrapora gamba* comb. n., *Typhochlaena* spp., *Pelinobius muticus*, *Phlogiellus* sp., *Poecilotheria* sp., *Encyocratella olivacea*, *Heteroscodra* sp., and *Stromatopelma* sp. do not present any type of apophysis or tibial modification. Some aviculariine species have a discrete elevation on prolateral tibia that can be covered or not by a cluster of setae but clearly does not form a branch. This discrete elevation on apical prolateral tibia is found in *Ybyrapora diversipes* comb. n., *Avicularia hirschii*, *Avicularia minatrix*, *Avicularia lynnae* sp. n., and in *Avicularia caei* sp. n., and was coded as state 0 since it cannot be considered as a true apophysis.

**57. Tibial apophysis on leg I, shape: (0) 2 branches; (1) 1 branch with a megaspine; (2) 1 branch with setae (Figs 310–311).**

Males of many barychelid and theraphosid species have tibial apophysis composed of two branches on leg I (Raven 1985) (state 0). This type of apophysis should have been present on the ancestral Theraphosoidina, suffering modifications and losses in all derivate groups (Raven 1985). This condition is present in Theraphosinae (represented here in the analysis by *Lasiodora* sp. and *Phrixotrichus vulpinus*), in Ischnocolinae (here represented by *Holothele rondoni*) and in Paratropididae (represented by *Melloina santuario*). The presence of tibial apophysis composed of single branch with a megaspine (state 1) is found exclusively in Harpactirinae (*Pterinochilus* sp. in our analysis) (Gallon 2003).

The presence of a tibial apophysis with a single branch bearing spiniform setae (state 2, figs 310–311) is a condition widely distributed in Aviculariinae. All *Pachistopelma* spp., *Iridopelma* spp., *Caribena* gen. n. spp. as well as *Avicularia rickwesti* comb. n. and most *Avicularia* species have this condition. This condition also appears in outgroup taxon *Haplopelma minax* (Ornithoctoninae).

**58. Tibial apophysis on leg I, one branch with setae, spiniform setae on a branch, branch development; (0) weakly-developed branch (Fig. 310); (1) well-developed branch (Fig. 311).**

We detected different degrees of branch development in species that have a single and well-defined branch covered with spiniform setae (state 1 for character 57). The branch can be weakly-developed, not projecting far from tibia longitudinal axis. This is the condition of *Pachistopelma* spp., *Iridopelma* spp. and *Caribena* gen. n. A well-developed branch projected far from tibia longitudinal axis is present in most *Avicularia* species (*Avicularia avicularia*, *Avicularia rufa*, *Avicularia juruensis*, *Avicularia variegata* stat. n., *Avicularia taunayi*, *Avicularia purpurea* and *Avicularia merianae* sp. n.), in *Avicularia rickwesti* comb. n., and also in *Haplopelma* sp.

**59. Tibial apophysis in leg II, occurrence: (0) absent; (1) present.**

60. Type I urticating setae, occurrence: (0) absent; (1) present.*

**61. Type II urticating setae, any life stage, occurrence: (0) absent; (1) present.**

Despite females of *Pachistopelma* spp. lacking type II urticating setae, immature stages and males of both *Pachistopelma* species do have the type II urticating setae. As we do not know immature stages nor males of *Iridopelma marcoi*, this species was coded as “?”.

**62. Type II urticating setae, adult female, occurrence: (0) present; (1) absent.**

Mature females of *Pachistopelma* spp. and of *Iridopelma marcoi* lack type II urticating setae.

**63. Type II urticating setae, length, females: (0) up to 0.90mm (Figs 16–17); (1) more than 1 mm (Fig. 243).**

According to Cooke et al. (1972), urticating setae type II are approximately 0.5–1.5 mm in length. Bertani et al. (2003) observed that *Caribena versicolor* comb. n. has type II setae that is more slender and longer than the *Avicularia* species—up to 1.37 mm in females. In fact, this species as well as *Caribena laeta* comb. n. has longer and slender setae when compared with *Avicularia* spp., what was confirmed by comparison of scanning microscopy images of setae of both genera (Figs 17–18).

When urticating setae are analyzed, the gender of specimens examined should be considered because males have setae significantly longer than females (Stradling 1978, Bertani and Guadanucci 2013).

**64. Type III urticating setae, occurrence: (0) absent; (1) present.**

65. Type IV urticating setae, occurrence: (0) absent; (1) present.*

**66. Type V urticating setae, occurrence: (0) absent; (1) present.**

**67. Legs, ratio between length of leg IV and I, males: (0) leg IV more than 10% longer than leg I; (1) leg IV roughly the same length as leg I; (2) leg IV more than 10% shorter than leg I.**

**68. Legs, ratio between length of leg IV and I, females: (0) leg IV more than 10% longer than leg I; (1) leg IV roughly the same length as leg I; (2) leg IV more than 10% shorter than leg I.**

**69. Habits, females: (0) retreat within surface layers of soil; (1) arboreal; (2) opportunistic; (3) fossorial.**

**70. Arboreal retreat made by adults: (0) built on tree trunk or on palm tree leaf base; (1) built in leaves, normally with two or more leaves connected by silk; (2) built under loosened tree bark; (3) bromelicolous.**

## Taxonomy

### Family Theraphosidae Thorell, 1869

#### Subfamily Aviculariinae

Taxon classificationAnimaliaORDOFAMILIA

Simon, 1889

Aviculariae Simon, 1889: 213; 1891: 312.
Avicularieae Simon, 1892: 170; 1903: 918; Pocock 1895: 229; 1901: 547.
Aviculariieae Simon, 1903: 958.
Aviculariinae Petrunkevitch, 1928: 34, 81; 1929: 48; 1939: 152, 274; Schiapelli & Gerschman de Pikelin 1945: 209; Bonnet 1955: 833; Bücherl 1957: 380; Raven 1985: 119; West et al. 2008: 37, 53, 55, 56; Bertani 2012: 5, 73.
Avicularias Mello-Leitão, 1923: 314.

##### Diagnosis.

Aviculariines can be distinguished by the conjunction of the following characters: legs aspinose or with few apical spines on ventral tibiae and metatarsi; metatarsi and tarsi with scopulae very extended laterally, mainly on anterior legs, giving a spatulate appearance (Figs 299–301); two completely separated spermathecae (Fig. 21); absence of spiniform setae on lower prolateral maxillae.

Included genera: *Antillena* gen. n., *Avicularia* Lamarck, 1818, *Caribena* gen. n., *Ephebopus* Simon, 1892, *Heteroscodra* Pocock, 1899, *Iridopelma* Pocock, 1901, *Pachistopelma* Pocock, 1901, *Psalmopoeus* Pocock, 1895, *Stromatopelma* Karsch, 1881, *Tapinauchenius* Ausserer, 1871, *Typhochlaena* C. L. Koch, 1850, *Ybyrapora* gen. n.

Distribution (Fig. 3): from state of Vera Cruz in Mexico, throughout Central America and some Caribbean islands, northern and central South America to state of São Paulo, Brazil. They can be found from the Atlantic to Pacific coasts. Two genera (*Heteroscodra* and *Stromatopelma*) are found in West and Central Africa.

##### Key to Aviculariinae genera

(Females of *Avicularia lynnae* sp. n. and *Avicularia caei* sp. n. are unknown)

**Table:** 

1	Presence of a paddle of urticating setae (type V) on prolateral distal palpal femora (Fig. 4)	***Ephebopus***
–	Absence of a paddle of urticating setae (type V) on prolateral distal palpal femora	**2**
2	Presence of black marks on dorsal tibiae, metatarsi and tarsi (Africa) (Fig. 5)	**3**
–	Absence of black marks on dorsal tibiae, metatarsi and tarsi (New World)	**4**
3	Tibia IV incrassate (Fig. 6)	***Heteroscodra***
–	Tibia IV not incrassate	***Stromatopelma***
4	Sternum as long as wide, truncated behind (Fig. 7); posterior lateral spinnerets with domed, short distal article (Fig. 8)	***Typhochlaena***
–	Sternum longer than wide (Fig. 9), posterior lateral spinnerets with digitiform distal article (Fig. 10)	**5**
5	Spines present on ventral apical tibiae and/or metatarsi	**6**
–	Spines absent in all legs	**7**
6	Stridulatory setae forming a lyra on prolateral maxilla (Fig. 11)	***Psalmopoeus***
–	Stridulatory setae absent on maxilla	***Tapinauchenius***
7	Anterior row of eyes straight or very slightly procurve (Fig. 12); dorso-ventrally flattened abdomen in females	***Pachistopelma***
–	Anterior row of eyes strongly procurve (Fig. 13); abdomen not flattened	**8**
8	Spermathecae very short and broad, with distal half strongly sclerotized (Fig. 284); male palpal bulb with well-developed keels (Figs 285, 288) (Hispaniola Island)	***Antillena* gen. n.**
–	Spermatheca long, not strongly sclerotized; male palpal bulb lacking keels	**9**
9	Urticating setae type II very slender (Fig. 18) on a conspicuous patch on abdomen dorsum (Fig. 254) (Caribbean Islands: Martinique, Puerto Rico and U. S. Virgin Islands) (Fig. 226)	***Caribena* gen. n.**
–	Urticating setae type II stout (Figs 15–17) spread over most abdominal area (Figs 302–304)	**10**
10	Spermathecae lacking an accentuated outwards curvature medially, with distal portion far from base (Fig. 14); male with tibial apophyses on legs I and II	***Iridopelma***
–	Spermathecae with an accentuated outwards curvature medially (Fig. 21); male lacking tibial apophysis on leg II	**11**
11	Female	**12**
–	Male	**13**
12	Spermathecae virtually non-sclerotized (Figs 257–259) (southeastern and part of northeastern Brazil)	***Ybyrapora* gen. n.**
–	Spermathecae with a well-sclerotized area (Fig. 21)	***Avicularia***
13	Tibial apophysis on leg I absent (Fig. 308) or tibia with discrete elevation covered by a cluster of setae on apical portion (Fig. 309) **14**
–	Tibial apophysis on leg I present, well-developed (Fig. 311)	***Avicularia*** (part)
14	Cymbium with a weakly-developed process on retrolateral lobe or process lacking (Fig. 305)	**15**
–	Cymbium with a well-developed process on retrolateral lobe (Figs 177, 280)	**16**
15	Abdomen dorsum with a single stripe (Fig. 297) (southeastern and part of northeastern Brazil)	.***Ybyrapora* gen. n.** (part)
–	Abdomen dorsum with orange spots on its sides (Figs 89) (Venezuela)	***Avicularia*** (part)
16	Male palpal bulb embolus strongly curved (Figs 276–278); tarsi and metatarsi with dorso-medial conspicuous orange stripe and leg rings on femora, tibia and metatarsi orange (Bertani and Fukushima 2009, figs A1–A2) (southeastern and part of northeastern Brazil)	***Ybyrapora* gen. n.** (part)
–	Male palpal bulb embolus curved (Figs 203, 212); tarsi and metatarsi without orange stripes, and leg rings on femora, tibia and metatarsi whitish or yellow (Figs 209, 218)	***Avicularia*** (part)

#### Genus Avicularia

Taxon classificationAnimaliaORDOFAMILIA

Lamarck, 1818

Figs 1 , 9–10 , 13 , 15–17 , 19 , 20 , 21–27 , 28–35 , 36–43 , 44–49 , 50 , 51 , 52–53 , 54–57 , 58–65 , 66–73 , 74–75 , 76 , 77 , 78–79 , 80–87 , 88–89 , 90 , 91–92 , 93–100 , 101–104 , 105 , 106–108 , 109–116 , 117–122 , 123–126 , 127 , 128–130 , 131–138 , 139–141 , 142–147 , 148–153 , 154–157 , 158–165 , 166–171 , 172 , 173–180 , 181–183 , 184–187 , 188–195 , 196–198 , 199–200 , 201–208 , 209 , 210–217 , 218 , 299–300 , 302–304 , 307 , 309 , 311 , 312–315 , 316–317

Aranea Linnaeus, 1758: 622 (in part: Avicularia avicularia); Fabricius 1775: 438 (in part: Avicularia avicularia); 1793: 424 (in part: Avicularia avicularia); De Geer 1778: 313, pl. 38, fig. 8 (in part: Avicularia avicularia); 1783: 122, pl. 38, figs 8–10 (in part: Avicularia avicularia); Latreille 1802: 49 (in part: Avicularia avicularia). Name abandoned as generic name to be used for the Order name (Thorell 1870: 151).
Mygale Latreille, 1802: 49, 1804: 152, pl. LXII, fig.1 (in part: Mygale avicularia); 1806: 85 (in part: Mygale avicularia); Walckenaer 1805: 4 (in part: Mygale avicularia); Hahn 1820: 5 (in part: Mygale avicularia); Simon 1864: 70, figs 26–28 (in part: Mygale avicularia). Name preoccupied in Mammalia by Mygale Cuvier, 1800 (Thorell 1870: 162).
Avicularia Lamarck, 1818: 107 (type species Aranea avicularia Linnaeus, 1758 by subsequent designation in direction 67 of ICZN (1957: 116)); Thorell 1870: 168; Ausserer 1871: 128, 201; 1875: 138; Simon 1889: 213; 1892: 171; 1903: 958, 960; F. O. Pickard-Cambridge 1896: 740, 746; 1899: 42; Pocock 1901: 548; Mello-Leitão 1923: 320, 376; ICZN 1928: 395; Petrunkevitch 1928: 81; Roewer 1942: 253; Bonnet 1955: 826; Valerio 1979: 307; Raven 1985: 119; Gallon 2008: 243; World Spider Catalog 2016.
Eurypelma C. L. Koch, 1850: 73, 74 (in part); Simon 1864: 67 (in part); Roewer 1942: 238 (in part); Bonnet 1955: 1828; Raven 1985: 146, 153. Considered objective synonym of Avicularia by Raven (1985: 146).
Avicuscodra Strand, 1908: 771 (type species by original designation Avicuscodra arabica Strand, 1908, female, Egypt, El-Tor [28°14'N, 33°37'E], (Tor, Arabien [*sic*]) Rüppell, SMF 2660, examined); Petrunkevitch 1928: 81; Roewer 1942: 256; Gallon 2008: 243. First synonymized by Gallon (2008: 243).
Ancylochiros Mello-Leitão, 1920: 41 (type species by original designation Ancylochiros taunayi Mello-Leitão, 1920, immature male, Brazil, Minas Gerais, Mariana [20°22'S, 43°25'W], J. P. Fonseca leg., MZUSP 327, examined,); 1923: 318; 376; Roewer 1942: 225; Raven 1985: 149. First synonymized by Raven (1985).
Anchylochyrus Petrunkevitch, 1928: 83 (unjustified emendation per Bonnet 1955); Strand 1929: 12.

##### Type species.

*Aranea avicularia* Linnaeus, 1758, by subsequent designation (ICZN 1957).

##### Species included.

*Avicularia avicularia*, *Avicularia caei* sp. n., *Avicularia glauca, Avicularia hirschii*, *Avicularia juruensis*, *Avicularia lynnae* sp. n., *Avicularia merianae* sp. n., *Avicularia minatrix*, *Avicularia purpurea*, *Avicularia rufa*, *Avicularia taunayi* and *Avicularia variegata* stat. n.

##### Diagnosis.

*Avicularia* resembles *Caribena* gen. n., *Ybyrapora* gen. n., *Iridopelma* and *Typhochlaena* by the procurve anterior row of eyes (Fig. 13). It can be distinguished from *Typhochlaena* by the digitiform apical article of PLS (Fig. 10). It differs from *Iridopelma* by male lacking tibial apopysis on tibiae II and female by spermathecae long, with accentuated outwards curvature medially (Fig. 21). It differs from *Caribena* gen. n. by stout urticating setae on abdomen dorsum of male and female (Figs 15–17) and by rounded process on cymbium (Fig. 307). From *Ybyrapora* gen. n. it can be distinguished by sclerotized spermathecae in females (Fig. 21) and presence of developed tibial apophysis in males (Fig. 311), except *Avicularia minatrix*, *Avicularia lynnae* sp. n., *Avicularia caei* sp. n., and *A hirschii* that lack tibial apophysis. Males of these *Avicularia* species can be distinguished from *Ybyrapora diversipes* comb. n. by the embolus less curved in frontal view (Fig. 30). From *Ybyrapora sooretama* comb. n. and *Ybyrapora gamba* comb. n. they differ in terms of the presence of a well-developed process on cymbium (Fig. 307), except *Avicularia minatrix*. *Avicularia minatrix* can be distinguished from *Ybyrapora sooretama* comb. n. and *Ybyrapora gamba* comb. n. by the abdomen dorsum black having orange spots on its side (Fig. 89).

**Figures 4–14.**
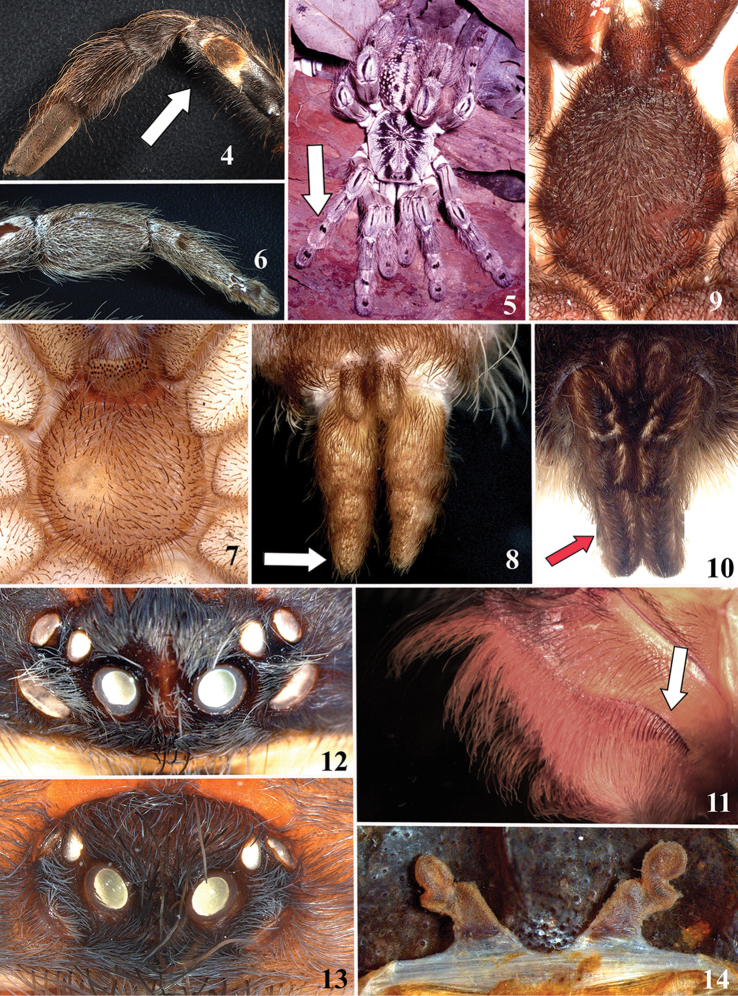
Aviculariinae characters. **4** *Ephebopus murinus*, palp with paddle of urticating setae type V (arrow) on prolateral side **5–6** *Heteroscodra* sp. **5** black marks (arrow) on dorsal tibiae, metatarsi and tarsi **6** tibia IV incrassate **7** *Typhochlaena curumim*, sternum as long as wide **8** *Typhoclaena seladonia*, posterior lateral spinnerets with domed article (arrow) **9–10** *Avicularia merianae* sp. n. **9** sternum longer than wide **10** posterior lateral spinnerets with digitiform distal article (arrow) **11** *Psalmopoeus* sp., stridulatory setae forming a lyra (arrow) on prolateral maxilla **12** *Tapinauchenius* sp., straight anterior row of eyes **13** *Avicularia avicularia*, anterior row of eyes strongly procurve **14** *Iridopelma hirsutum*, spermathecae.

##### Description.

**Figures 15–18.**
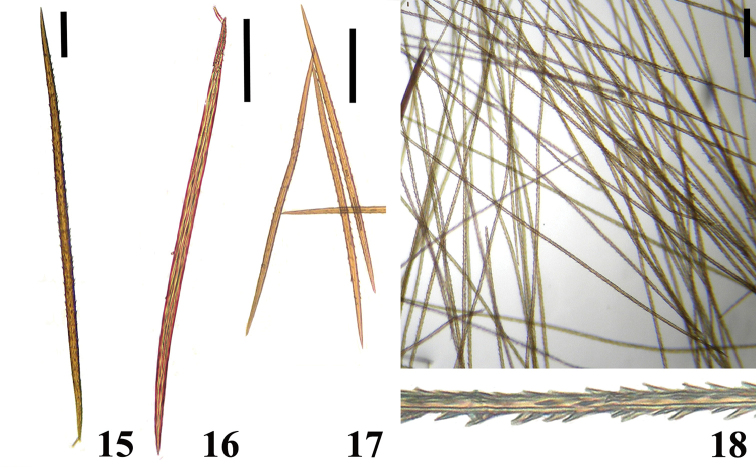
Urticating setae type II in Aviculariinae. **15** *Avicularia juruensis* male (CAS 4), showing well-developed barbs along all its length **16** *Avicularia variegata* stat. n. female (IBSP 7900), showing weakly-developed barbs only near the stalk **17** *Avicularia avicularia* female morphotype 5 (MNRJ 06916) from Pando, showing developed barbs along almost entire length **18** *Caribena versicolor* comb. n. male (MNHN−AR 4904), very slender setae with barbs along all length. Scale bars = 0.1 mm.

Carapace slightly longer than wide, cephalic region slightly raised. Cephalic and thoracic striae inconspicuous due to setae density. Fovea deep or shallow, slightly recurve (most species) or straight. Chelicera without rastelum. Eye tubercle distinct, raised or slightly raised, wider than long. Anterior row of eyes procurve (Fig. 13), posterior recurve or slightly recurve (most species). Clypeus narrow (most species) or absent. Labium subquadrate, longer than wide, with 50–133 cuspules spaced by one or two diameters from each other on the anterior third center. Maxillary lyra absent. Maxilla subretangular, anterior lobe distinctly produced into conical process, inner angle bearing 85–215 cuspules. Sternum longer than wide, posterior angle acute, not separating coxae IV. Three pairs of sigilla, some pairs sometimes not evident. Anterior ellipsoidal or rounded, middle rounded, posterior ellipsoidal (most species) or rounded. All positioned one diameter or less from margin. Leg formula: I=IV II III (most species) or IV I II III (*Avicularia avicularia*, *Avicularia rufa* and *Avicularia hirschii* female). Clavate trichobothria on distal 2/3 or 1/2 of tarsi I–IV. Tarsi I–IV fully scopulate, IV divided by a band of setae or divided by a bald stripe. Metatarsi I–II fully scopulate in most species, III 1/3 to 1/2 distal scopulate and IV 1/5 to 1/2 distal scopulate. Metatarsi IV divided by a row of setae. Scopulae of tarsi and metatarsi I–II very extended laterally giving them a spatulate appearance (Figs 299–300). Femora IV without retrolateral scopulae. Stridulatory setae absent. Legs aspinose. ITC absent; STC without denticles. Posterior lateral spinnerets digitiform (Fig. 10). Typical stout type II urticating setae on dorsal abdomen in females and males, 0.36 to 0.72 mm long, 0.11–0.019 mm wide in females and 0.78 to 1.11 mm long, 0.013–0.025 mm wide in males (Figs 15–17). Male tibiae I with apophysis (most species) (Fig. 311) or with discrete elevation covered by a cluster of setae on apical portion (Fig. 309), on prolateral side. Tibial apophysis with a single branch having well-developed base and grouped spiniform setae distally (Figs 33–35). Tibiae II lacking apophysis. Male metatarsus I touches retrolaterally tibial apophysis’ setae when folded. Globous bulb with small subtegulum; prominence on prolateral tegulum developed (most species) (Fig. 314), well-developed (*Avicularia variegata* stat. n. and *Avicularia juruensis*) (Fig. 315), weakly-developed (*Avicularia caei* sp. n.) (Fig. 313) or absent (*Avicularia minatrix*) (Fig. 312). Embolus not flattened, without keels, about 3.0 to 3.5 times tegulum’s length (most species) or more than 4 times tegulum’s length (*Avicularia lynnae* sp. n. and *Avicularia caei* sp. n.) in retrolateral view. Embolus medial portion and tegulum’s margin form an acute angle in retrolateral view (Fig. 323). Embolus with basal part very curved in frontal view (Figs 312–315), thin distal width and tip narrowing abruptly (Figs 316–317) or tapering (Figs 318–319). Cymbium subtriangular with subequal lobes, having a well-developed rounded process on retrolateral lobe (most species) (Fig. 307) or lacking any process (Fig. 162). Cymbium process bearing thick setae (most species) (Fig. 307) or thin setae (Fig. 205). Spermathecae with an accentuated outwards curvature medially, completely separated, and long (Fig. 21). Spermathecae not-twisted (except *Avicularia hirschii*) with walls lacking projections or lobes (except *Avicularia taunayi*) (Figs 21–27). Spermathecae midwidth as wide as its base width (most species) (Fig. 21) or midwidth expanded, about 1.5 times the apex width (*Avicularia variegata* stat. n., *Avicularia juruensis* and *Avicularia taunayi*) (Fig. 54). Spermathecae with weakly-sclerotized area shorter than half the length of well-sclerotized area (most species) (Fig. 21) or at least same size of well-sclerotized area (*Avicularia purpurea* and *Avicularia merianae* sp. n.) (Fig. 154). Abdomen dorsum of females with homogeneously distributed setae (most species) (Fig. 304) or with long guard-setae grouped on lateral and dorsal anterior areas (Figs 302–303). Legs and palps with long guard-setae having homogeneous coloration along its length (*Avicularia avicularia*, most morphotypes; *Avicularia juruensis* morphotype 3, *Avicularia purpurea*, *Avicularia taunayi* and *Avicularia merianae* sp. n.) (Fig. 299) or long guard-setae with darker base and contrasting whitish apex (*Avicularia avicularia* morphotypes 6 and 7; *Avicularia variegata* stat. n.; *Avicularia rufa*; and *Avicularia juruensis* morphotype 2) (Fig. 300). Leg rings on distal femora, tibiae and metatarsi whitish (most species) (Fig. 299) or yellowish (*Avicularia avicularia*, morphotype 5; *Avicularia variegata* stat. n., morphotype 2; *Avicularia juruensis* morphotype 2; and *Avicularia rufa*) (Fig. 300). All species except *Avicularia minatrix* showing drastic ontogenetic changes on abdominal color pattern. Brownish juveniles lacking metallic green or blue sheen, with black tarsi contrasting with other lighter articles (except *Avicularia purpurea*) (Fig. 44). Juveniles having dorsal reddish abdomen with a single central longitudinal stripe and some transversal black stripes on each side (except *Avicularia hirschii*). Central longitudinal stripe disconnected from transversal stripes (*Avicularia avicularia, Avicularia variegata* stat. n., *Avicularia juruensis* and *Avicularia rufa*) (Fig. 44), connected with all transversal stripes in each side of abdomen (*Avicularia purpurea* and *Avicularia minatrix*) (Fig. 167), connected only with the anterior pair of transversal stripes (*Avicularia merianae* sp. n.), or connected with first and second anterior pair of transversal stripes (*Avicularia taunyai*) (Fig. 101).

**Figure 19.**
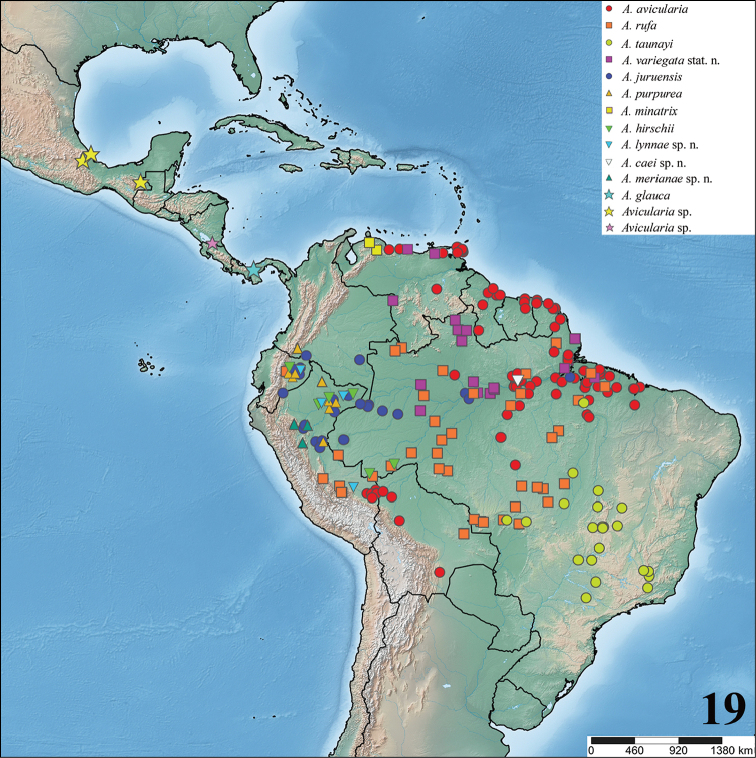
Map showing records of *Avicularia* Lamarck, 1818 species in Central and South America.

##### Distribution and habitat.

Costa Rica, Panama, Trinidad and Tobago, Venezuela, Guyana, Suriname, French Guiana, Colombia, Ecuador, Peru, Bolivia and Brazil. In Brazil, it occurs in the states of Roraima, Amapá, Amazonas, Pará, Maranhão, Tocantins, Acre, western Bahia, Rondônia, Mato Grosso, Goiás, Minas Gerais, São Paulo, and the Distrito Federal (Fig. 19). Valerio (1979) reports presence of *Avicularia* species in Costa Rica (see note on *Avicularia avicularia* redescription and on *Avicularia glauca* taxonomy). Beside this, there are reports in Lago Catemaco and other localities in the state of Veracruz, as well as in the state of Chiapas, Mexico (R. West and J. Mendoza, pers. comm.), but no Mexican specimens could be examined. Thus, despite the reliable records, it is not possible to know which species are in Mexico.

##### Remarks.

When erecting the genus *Avicularia*, Lamarck (1818) included in it three species described originally in *Aranea*, in this order: *Avicularia canceridea* (Lamarck, 1818), *Avicularia blondii* (Latreille, 1804), and *Avicularia fasciata* (Latreille, 1804). The first species, *Avicularia canceridea*, could be found in the Antilles in “Meridional America” (South America)(Lamarck 1818). As synonyms of *Avicularia canceridea*, Lamarck (1818) cited *Aranea avicularia* Linnaeus, 1758 (*sensu* Linnaeus 1758 and Fabricius 1793) and *Mygale avicularia* Latreille, 1804 (*sensu* Latreille 1804 and Walckenaer 1805). It is unclear why Lamarck (1818) did not use the pre-existent specific name *avicularia* instead of *canceridea*.

Thorell (1870) considered *Aranea avicularia* Linnaeus “as the type for *Avicularia*”, and *Avicularia canceridea* Lamarck and *Aranea vestiaria* De Geer as its synonyms. Apparently, this was the reason for the Lamarckian combination *Avicularia canceridea* being buried.

Ausserer (1871) considered *Avicularia canceridea* Lamarck, *Aranea avicularia* Linnaeus, *Aranea vestiaria* De Geer and *Mygale avicularia* Latreille as synonyms; however, he considered valid the binomy *Avicularia vestiaria* and stated it as the type species. F. O. Pickard-Cambridge (1899) also considered *Avicularia canceridea* Lamarck as a synonym of *Avicularia avicularia*, but stated this last one as the type species. Despite the specific epithet *avicularia* being the most used for the type species of *Avicularia* over the years, it was only officially considered as such in 1928 through Opinion 104 (ICZN 1928) and introduced in the official list of specific names in Zoology through Direction 67 of ICZN (ICZN 1957).

Herein, we tried to establish the real identity of *Avicularia avicularia* as the original description is uninformative and useless for species identification. The type locality, America, is also vague and the existence of types was controversial. Taking into consideration that *Aranea avicularia* is a very old species described by Linnaeus in the 10^th^ Edition of *Systema Naturae* (Linnaeus 1758), it would be a difficult task to track a type.

Apparently, Linnaeus (1758) did not designate holotypes. According to Papavero and Llorente-Bousquets (1995), the oldest citation regarding the type concept as we use, nowadays, is dated 1817 by the German dipterologist C. R. W. Wiedmann. In his publication, Wiedmann (1817 *apud* Papavero and Llorente-Bousquets 1995) approved the idea about designation of types in description of new species and creation of a *Normalmuseum* where all types should be deposited. Therefore, Linnaeus could not determine types since this concept was elaborated many years after publication of *Systema Naturae* (Papavero and Llorente-Bousquets 1995).

Linnean holotypes are also a problem for other specialists. Many botanists, for example, argue it is very difficult to know when Linnaeus used a well-defined specimen to describe a plant species. Thus, The Linnean Plant Name Typification Project was created to choose types from the specimens and illustrations that Linnaeus used in arriving at his concept of a species in question (The Linnean Society of London 2016). The creation of this project strengthens the ideia that Linnaeus did not designate holotypes. Specifically in *Avicularia*’s case, this idea is supported by the expressed citation by F. O. Pickard-Cambridge (1896) that the type is a figure of two specimens drawn by Merian (1726), and not a specimen.

Even with a doubtful existence of the *Avicularia avicularia* holotype, Gabriel et al. (2007) tried to locate it in The Linnean Society of London Collection. The authors found two specimens of *Aranea avicularia* considered by the curator as Linnean material (Gabriel et al. 2007) and observed they are two different species: a female of *Stromatopelma calceatum* Fabricius, 1793; and a male of *Harpactira atra* Latreille, 1832, both African species (Gabriel et al. 2007). They concluded that none of the African specimens in the Linnean collection could be considered types of *Avicularia avicularia* (Gabriel et al. 2007).

The ICZN states on the article 72.1.1 of its Code that “in the absence of holotype designation, or the designation of syntypes, or the subsequent designation of a lectotype, all [specimens] are syntypes and collectively they constitute the name-bearing type” (ICZN 1999). Additionally, the article 72.4.1. (ICZN 1999) states that “the type series of a nominal species-group taxon consists of all the specimens included by the author in the new nominal taxon (whether directly or by bibliographic reference)”. Thus, as there is no designation of a specific holotype in the original description, all the specimens used in the publications mentioned by Linnaeus in the description of *Aranea avicularia* are part of a syntypic series.

In that description, Linnaeus (1758) cited his own previous work (Linnaeus 1749), as well as Clusius (1611), Laet (1633), Piso and Marcgrave (1648), Worm (1655), Olearius (1666) and Merian (1705).

The spider drawn by Clusius (1611) seems, in fact, to be an aviculariine, since it has a hairy body and very developed subungueal tufts (Fig. 2). The spider was illustrated with low cephalic region and could be found in Baya de todos los Sanctos [*sic*] (Clusius 1611). The animal depicted resembles specimens of *Pachistopelma* spp., which can be found in Baía de Todos-os-Santos, a bay in the city of Salvador, state of Bahia, northeastern Brazil. Laet (1633) and Piso and Marcgrave (1648) also reproduced this illustration and indicated that this species is very common in Saint Aleixo Island (state of Pernambuco, Brazil), area where *Pachistopelma rufoniger* specimens are frequent and where also there is no record of *Avicularia* spp. Other authors (Olearius 1666, Worm 1655) used the same Clusius’ spider illustration to refer to this Brazilian species which we found to be a *Pachistopelma* species.

Merian (1705), on the other hand, illustrated two spiders with very developed and conspicuously colored subungueal tufts, unmistakably *Avicularia* sp. specimens (Fig. 1). One of them is leaving a typical retreat and feeding on ants and the other specimen is feeding on a small bird. Smith (2000b) stated that, probably because of this illustration, Linnaeus used the specific name “*avicularia*”, meaning “small bird” in Latin. Merian (1705) had drawn these spiders based on specimens observed during her expedition to Suriname. In the illustration’s comments, there is no indication of locality, nor any information that could lead to an inequivocal identification of the illustrated species.

Linnaeus (1758), in his *Aranea avicularia* description, also cited two illustrations of Seba (1734) of a spider from Ceylon, now Sri Lanka, which is probably a *Poecilotheria* specimen, and an unidentified specimen from America, which clearly is not an aviculariine.

Thus, among the syntypic *Avicularia avicularia* series are specimens of *Pachistopelma* spp. (Clusius 1611, Laet 1633, Piso and Margrave 1648, Worm 1655, Olearius 1666), *Avicularia* (Merian 1705) and unidentified specimens (Seba 1704). Apparently, the specimens used and/or illustrated by these authors are lost or have never been deposited in a zoological collection, but only observed in their natural habitat. Smith (2000a) believe that Linnaeus (1758) used the engraving done by Merian (1705) for describing *Aranea avicularia*, which was considered perfectly acceptable at that time. However, Smith (2000a) did not discard the possibility that Linnaeus had examined specimens collected by Merian, which could be deposited in particular collections, although the author states that much of Merian’s material is lost.

The ICZN Code article 72.4.1.1 states that “for a nominal species or subspecies established before 2000, any evidence, published or unpublished, may be taken into account to determine what specimens constitute the type series” (ICZN 1999). The Code also considered part of the syntypic series specimens of which there is evidence that they were known to the author and recognized by him when the nominal species was established (see example in article 72.4.1.1 of ICZN 1999). After searching some collections, we found three specimens of *Aranea avicularia* belonging to the Linnean collection at the Museum of Evolution of Uppsala University. Even though there is no irrefutable proof that these specimens once belonged to Linnaeus and were examined by him when describing the species (Dr. Mats Eriksson, pers. comm.), we think that two of three specimens found in the collection were used by Linnaeus in his work. The spiders’ donor was Adolf Fredrik, King of Sweden in the time Linnaeus was writing his *Systema Naturae* (Kullander 2001). The King had an extensive biological collection composed of plants and animals from all over the world, a common habit of wealthy and significant people of that time (Kullander 2001). During nine weeks between 1751 and 1754, Linnaeus, during that time a professor at Uppsala University, was housed in the Royal Castle to catalogue the royal collection; the result of this work was published in a book (Kullander 2001). It is probable that the King donated some material to Linnaeus, which were brought with him to Uppsala instead of going with most of the King’s material to the Swedish Museum of Natural History in Stockholm. Thus, those specimens of *Aranea avicularia* could have been really used by Linnaeus (1758) to describe the species and, therefore, they are part of the syntypic series.

**Figure 20.**
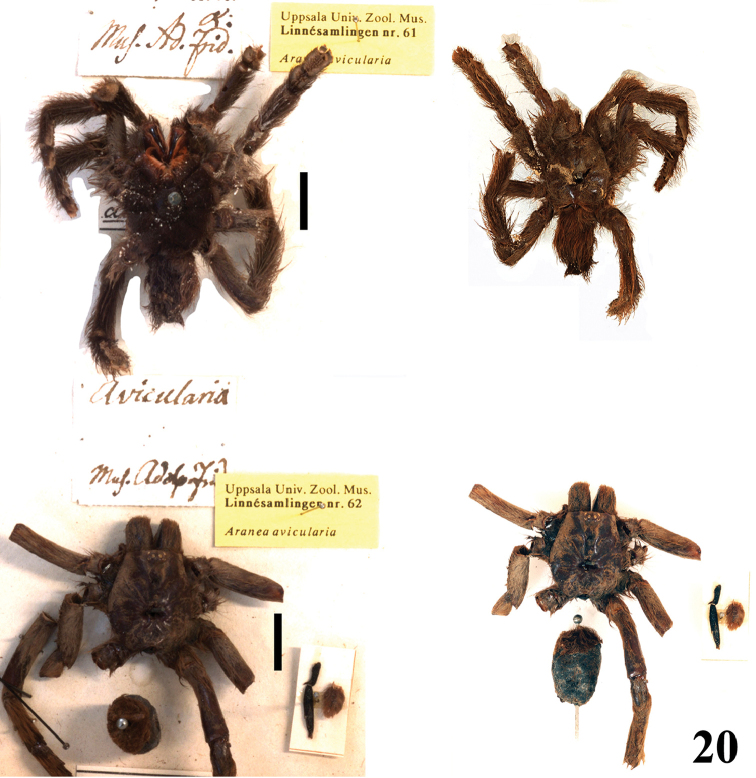
*Avicularia avicularia* (Linnaeus, 1758), types from Uppsala Collection (UUZM). Above, lectotype female (UUZM 61), below paralectotype (UUZM 62). Photo: Mats Eriksson, published with UUZM permission.

King Adolf’s specimens are in a dry, pinned collection, thus they cannot be properly examined and handle (Fig. 20). However, even by photography, it is clear that both are *Avicularia avicularia* specimens, probable from the morphotype found in the state of Pará, Brazil, since morphological features such as size and coloration match with those seen in specimens found in that area. Therefore, herein, we establish the specimen UUZM 61 from the Uppsala collection as lectotype and the specimen UUZM 62 as paralectotype, solving the oldest and one of the most vexing problems in mygalomorph taxonomy.

##### Key to *Avicularia* species

Male of *Avicularia glauca* and female of *Avicularia caei* sp.n. and *Avicularia lynnae* sp. n. are unknown.

**Female**

**Table:** 

1	Spermathecae long and twisted (Fig. 183)	***Avicularia hirschii***
–	Spermathecae not-twisted	**2**
2	Spermathecae with lobes from median to distal portions (Fig. 91)	***Avicularia taunayi***
–	Spemathecae lacking lobes	**3**
3	Spermathecae midwidth expanded, 1.5 times its base and apical widths (Fig. 54)	**4**
–	Spermathecae midwidth as wide as its base width (Fig. 21)	**5**
4	Legs with or without grizzled setae, brownish guard-setae on dark abdomen, and whitish or pale yellow rings on distal femora, tibiae and metatarsi (Figs 119, 122) (see morphotype characteristics and occurence areas)	***Avicularia juruensis***
–	All legs with grizzled setae, vivid reddish guard-setae on black abdomen, and whitish rings on distal femora, tibiae and metatarsi (Fig. 70); or all legs with grizzled setae, brownish guard-setae on black abdomen and pale yellow rings on distal femora, tibiae and metatarsi (Fig. 74)	***Avicularia variegata* stat. n.**
5	Leg IV longer (more than 10%) than leg I	**6**
–	Leg IV as long as leg I	**7**
6	All legs always with grizzled setae, vivid yellow rings on distal femora, tibiae and metatarsi, and abdomen with grey guard-setae grouped on lateral dorsal anterior areas and black short body setae (Fig. 145)	***Avicularia rufa***
–	Legs with or without grizzled setae, whitish or pale yellow rings on distal femora, tibiae and metatarsi and guard-setae grouped on lateral dorsal anterior area (Figs 36, 38–42, 45, 48) (see morphotype characteristics and occurence areas)	***Avicularia avicularia***
7	Abdomen dorsum black with lateral orange spots on its sides (Fig. 88), Venezuela	***Avicularia minatrix***
–	Abdomen dorsum with homogeneous color pattern, lacking orange spots	**8**
8	Carapace and legs with green sheen (Fig. 52), Panama	***Avicularia glauca***
–	Carapace and legs without green sheen, western South America	**9**
9	Carapace and legs with intense purple sheen (Figs 168, 170), abdomen black velvety (Fig. 304), Ecuador, Colombia and Peru	***Avicularia purpurea***
–	Carapace and legs with discrete pink sheen, abdomen with red brownish setae (Fig. 199), Peru	***Avicularia merianae* sp. n.**
**Males**
1	Embolus long, more than 4 times tegulum’s length (Figs 202, 211)	**2**
–	Embolus 2.5 to 3.5 times tegulum’s length (Fig. 29)	**3**
2	Abdomen dorsum with lateral stripes (Fig. 218) and tegulum with weakly-developed prominence (Fig. 313)	***Avicularia caei* sp. n.**
–	Abdomen dorsum with a single longitudinal stripe and tegulum with developed prominence (Fig. 209)	***Avicularia lynnae* sp. n.**
3	Tibia I with a discrete elevation covered by cluster of setae in apical portion, on prolateral side (Fig. 309)	**4**
–	Tibia I apophysis with well-developed branch bearing thick setae (Fig. 311)	**5**
4	Tegulum without prominence (Fig. 312), cymbium lacking process on retrolateral lobe (Fig. 84) and abdomen dorsum black with lateral orange spots on its sides (Fig. 89)	***Avicularia minatrix***
–	Tegulum with developed prominence (Fig. 175), cymbium with developed process on retrolateral lobe (Fig. 177)	***Avicularia hirschii***
5	Cymbium with retrolateral lobe lacking any process or with weakly-developed process (Fig. 162)	**6**
–	Cymbium with retrolateral lobe with well-developed process bearing thick setae (Fig. 307)	**7**
6	Carapace and legs with intense purple sheen (Figs 169, 171), abdomen black velvety, Ecuador, Colombia and Peru	***Avicularia purpurea***
–	Carapace and legs with discrete pink sheen, abdomen with red brownish guard-setae, Peru	***Avicularia merianae* sp. n.**
7	Leg IV longer (more than 10%) than leg I	**8**
–	Leg IV as long as leg I	**9**
8	All legs always with grizzled setae, distal femora, tibiae and metatarsi with vivid yellow rings and grey guard-setae on black abdomen (Fig. 300)	***Avicularia rufa***
–	Legs with or without grizzled setae, distal femora, tibiae and metatarsi with whitish or pale yellow rings (Figs 43, 46, 49) (see morphotype characteristics and occurrence areas)	***Avicularia avicularia***
9	Tegulum with developed prominence (Fig. 95), abdomen with three pairs of light brown spots extending from the dorsum to lateral region (Fig. 103)	***Avicularia taunayi***
–	Tegulum with well-developed prominence (Fig. 315), abdomen dorsum lacking spots	**10**
10	Legs with or without grizzled setae, abdomen dark with brownish guard-setae, and distal femora, tibia and metatarsi with whitish or pale yellow rings (Fig. 121) (see morphotype characteristics and occurrence areas)	***Avicularia juruensis***
–	Legs with grizzled setae, abdomen black with brownish guard-setae and distal femora, tibia and metatarsi with whitish or pale yellow rings (Figs 71, 75)	***Avicularia variegata* stat. n.**

#### Avicularia avicularia

Taxon classificationAnimaliaORDOFAMILIA

(Linnaeus, 1758)

Figs 1 , 13 , 17 , 19 , 20 , 21–27 , 28–35 , 36–43 , 44–49 , 50 , 51 , 299 , 303 , 311 , 314 , 316 , 323

Aranea avicularia Linnaeus, 1758: 622 (lectotype UUZM 61 and paralectotypes UUZM 62, UUZM 235 here designated, A. Fredrik leg., examined by photos); Fabricius 1775: 438; 1787: 346; 1793: 424; De Geer 1778: 313, pl. 38, fig. 8; 1783: 122, pl. 38, fig. 8; Latreille 1802: 49; ICZN 1957: 116.
Mygale avicularia : Latreille 1804: 152, pl. LXII, fig. 1; 1806: 32; Walckenaer 1805: 4; Hahn 1820: 5, t. 3; 1833: 101, tab. 25, fig. 75; C. L. Koch 1841: 73, tab. CCCXIII, fig. 737.
Mygale scoparia C. L. Koch, 1841: 54, fig. 725. First synonymized by F. O. Pickard-Cambridge 1896: 741.
Eurypelma avicularia : C. L. Koch 1850: 73; Simon 1864: 67. Considered objective synonym per Raven 1985: 146.
Avicularia velutina Simon, 1889: 213 (types not found; topotypes female and male, Venezuela, state of Carabobo, Puerto Cabello [10°27'N, 68°00'W], Collection E. Simon, MNHN–AR 4883, examined); Petrunkevitch 1911: 51; Mello-Leitão 1923: 377; Roewer 1942: 255; Schiapelli and Gerschman 1945: 191, pl. XVII; Bonnet 1955: 833. **Syn. n.**
Avicularia avicularia : Simon 1892: 171, fig. 120; F. O. Pickard-Cambridge 1896: 741, pl. 33, figs 10–11, pl. 34, fig. 19, pl. 35, fig. 13; Strand 1907a: 224; Strand 1907b: 89; Petrunkevitch 1911: 49; ICZN 1928: 395; Mello-Leitão 1923: 320, 324, 376; Bücherl 1957: 404, figs 91–91a; Valerio 1979: 307, figs 7–10; Roewer 1942: 253; Bonnet 1955: 827; 1957: 119; World Spider Catalog 2016.
Avicularia exilis Strand, 1907a: 220 (holotype, male, South America, perhaps Suriname, no further information, Coll. Kirschbaum, MWNH 418, examined by photo); Petrunkevitch 1911: 50; 1939: 286; Mello-Leitão 1923: 377; Roewer 1942: 254; Bonnet 1955: 830; World Spider Catalog 2016. **Syn. n.**
Avicularia ancylochyra Mello-Leitão, 1923: 330 (holotype, immature male, Brazil, state of Pará, Rio Tapajós [4°44"S, 56°36'W], E. Garbe col., E2977 C3141, examined); Petrunkevitch 1939: 284; Schiapelli & Gerschman de Pikelin 1945: 210, lam. XXIV; Bonnet 1955: 827. **Syn. n.**
Avicularia cuminami Mello-Leitão, 1930: 56, fig. 3 (holotype, immature, Brazil, Rio Cuminá [1°21'N, 56°04'W], G. Cruls col., MNRJ 21, examined); Petrunkevitch 1939: 286; Roewer 1942: 254; World Spider Catalog 2016. **Syn. n.**
Avicularia nigrotaeniata Mello-Leitão, 1940: 178, fig. 1 (holotype, immature male, Guyana, Kamara, Rio Cuyuni [6°24'N, 58°46'W], C. W. Richards col., Oxford University expedition, 22 November 1929, n°. 6290, BMNH 1930.12.14.168, examined); Brignoli 1983: 134; World Spider Catalog 2016. **Syn. n.**
Avicularia ancylochira : Roewer 1942: 253; World Spider Catalog 2016 (considered as the spelling apparently intended by Mello-Leitão, 1923).
Avicularia cuminamensis : Bonnet 1955: 830 (invalid emendation).

##### Remarks.

*Avicularia velutina* syntypes are two females from the forest of San Esteban, Venezuela, and a specimen from a mountain in North Venezuela (Simon 1889). After a search on Simon’s collection at MNHN, it was found 4 specimens from Venezuela labeled as *Avicularia velutina*. One is an immature from San Esteban (MNHN–AR4888), but its carapace measurement (4.6 mm) is not compatible with the female used on description (13.8 mm). Other two, one male and one female (MNHN–AR4883), are from Puerto Cabello, a town very close to San Esteban, locality mentioned on the description and where recently was created a National Park. Despite the female having the carapace measurement (13.25 mm) compatible with the female used in the description (13.8 mm), its collection data is not exactly the same of the one of the syntype series. Adding to this, its pair on the vial is a male, not other female as described by Simon (1889). The other female found in collection is from Caracas (MNHN–AR4894), on North Venezuela, and it is smaller than the Puerto Cabello’s female. Thus, herein, we consider the syntypes of *Avicularia velutina* Simon, 1889 lost. However, considering we examined topotypes and that their characteristics match with those found in *Avicularia avicularia*, only with slight differences on coloration, we decide to establish *Avicularia velutina* Simon, 1889 as a junior synonym of *Avicularia avicularia* (Linnaeus, 1758).

*Avicularia exilis* holotype is clearly an *Avicularia* since it has well-developed scopula, anterior row of eyes procurved and tibial apophysis with a single branch having well-developed base and grouped spiniform setae distally. The specimen has palpal bulb with developed prominence, leg IV longer than leg I, not grizzled guard-setae on palps and legs, and overall reddish brown coloration. These characteristics match *Avicularia avicularia*, which is found in Suriname, the probable type locality *Avicularia exilis*. Thus, we consider *Avicularia exilis* Strand, 1907 as junior synonym of *Avicularia avicularia* (Linnaeus, 1758).

*Avicularia ancylochyra* holotype has tarsi with well-developed scopula, anterior row of eyes procurved, no spines on legs, urticating setae of type II on abdomen dorsum, and abdominal pattern similar to *Avicularia avicularia*, *Avicularia rufa, Avicularia juruensis*, and *Avicularia variegata* stat. n. The leg IV is longer than leg I, it bears whitish legs rings (not vivid yellow) and type locality is in the state of Pará, Brazil. All these characters match *Avicularia avicularia*; therefore, we consider *Avicularia ancylochyra* Mello-Leitão, 1923 a junior synonym of *Avicularia avicularia* (Linnaeus, 1758).

*Avicularia cuminami* holotype has tarsal and metatarsal scopulae laterally expanded, anterior row of eyes procurve and type locality is state of Pará, Brazil. These characteristics match *Avicularia avicularia*; thus, we consider *Avicularia cuminami* Mello-Leitão, 1930 a junior synonym of *Avicularia avicularia* (Linnaeus, 1758).

*Avicularia nigrotaeniata* holotype is an immature male lacking spines on legs and with spatulated scopulae on tarsi and metatarsi. It has characteristics of *Avicularia* genus such as anterior row of eyes procurve and abdomen dorsum with lateral stripes and a black central longitudinal stripe. In Guyana, the type locality, *Avicularia avicularia* is the only species found. Thus, we consider *Avicularia nigrotaeniata* Mello-Leitão, 1940 a junior synonym of *Avicularia avicularia* (Linnaeus, 1758).

##### Diagnosis.

Male and female of *Avicularia avicularia* resemble *Avicularia rufa* and female of *Avicularia hirschii* by having leg IV longer than leg I. Females of *Avicularia avicularia* can be distinguished from those of *Avicularia hirschii* by the non-twisted spermathecae. Males and females of *Avicularia avicularia* can be distinguished from those of *Avicularia rufa* by having whitish rings on distal femora, tibiae and metatarsi (most morphotypes, Figs 36, 38–40) or pale yellow rings (morphotype 5, Fig. 41) combined with legs and palps with long guard-setae with homogeneous coloration (all morphotypes except 6 and 7, but these have intense green metallic sheen on carapace and legs, absent in *Avicularia rufa*).

##### Material examined.

1 female, Brazil, Pará, Altamira, Paratipuã, Ponto 5, A. P. L. Giupponi & D. Pedroso col., 12 April 2009 (MNRJ 13995); 2 males and 1 female, Amapá, Macapá, Parque Zoobotânico (IEPA), C. Costa, P. Magno & C. A. Júlio col., 20 August 1996 (MNRJ 13659A).

##### Additional material.

No further information: 1 juvenile female (MPEG 5140); 1 male, 1 female (IBSP 2419); 1 male, died in 27 October 1985 (IBSP IBA 414); 1 female (IBSP 8842); 1 female, IBAMA, died in January 2000, ref. 84526 (IBSP 8031); 1 immature male (IBSP 9916); 1 female (MNHN–AR Box 301); VENEZUELA: 1 juvenile female, no further information, Sullivan det. 2007 (AMNH Ve23); 1 female, no further information, Steve Brody coll. (AMNH Ve28); *Bolívar*: Hato La Vergareña (6°45'N, 63°30'W) 400–500 ft., 25 October 1954, J. J. Wurdack & N. G. L. Guppy col. (AMNH Ve22); *Carabobo*: Puerto Cabello [10°27'N, 68°00'W], 1 female, 1 male, no further information, Collection E. Simon (MNHN–AR 4883 Box 302); *Distrito Capital*: Caracas [10°29'N, 66°54'W], 1 female, no further information, Collection E. Simon (MNHN–AR 4894); *Monagas*: Caripito [10°06'N, 63°06'W], 1 female, 10 May 1942, Tropical Research Expedition, N. Y. Zoological Society, W. H. Beebe col. (AMNH Ve30); Trinidad and Tobago: no further information, 1 female (AMNH TR133); 1 female, M. Nieves col. (AMNH TR131); 1 male (AMNH TR129); probably from Port Spain sent in by T. H. G. Aigken [*sic*] (Aitken), 1 male, 1 immature male, Gertsch det. (AMNH TR130); 2 immatures, 3–4 instar group [*sic*], A. Bordes col., 1972 (AMNH TR tube); several spiderlings, A. Bordes col. (AMNH TR165); *Couva-Tabaquite-Talparo*: Freeport [10°26'N, 61°24'W], 1 female, E. N. Kjellesvig-Waering col., 1 May 1966 (AMNH TR181); *Diego Martin*: Fort George [10°41'N, 61°32'W], POS, 1 male, M. Nieves col., June 1957 (AMNH TR147); Goodwood Park [10°41'N, 61°34'W] (Goodward Park [*sic*]), 5 miles NW of Port of Spain, 300 m elevation, 1 female, 1 male, E. N. Kjellesvig-Waering col., 1 May 1965 (AMNH TR143); 1 immature, E. N. Kjellesvig-Waering col., 30 September 1965, (AMNH TR176); *Port of Spain* [10°39'N, 61°31'W]: 1 male, E. N. Kjellesvig-Waering col., 1 May 1954 (AMNH TR136); 1 female, E. N. Kjellesvig-Waering col., 18 September 1963 (AMNH TR126); 1 female, E. N. Kjellesvig-Waering col., 16 July 1965 (AMNH TR139); 1 male, 1 female, E. N. Kjellesvig-Waering col., 18 June 1968 (AMNH TR178); 1 male, R. S. Mathews col., April 1890 (AMNH 1.12); *Rio Claro-Mayaro*: La Horquette, Valley Road, 1 immature female, T. H. G. Aitken col., 6 April 1960, #78 (AMNH TR138); Maraval [10°41'N, 61°31'W], 3 males, 2 females, R. C. West col., 12 May 1981 (AMNH RW09, RW11, RW12, RW13, RW10, respectively); Mayaro Beach area [10°14'N, 61°00'W], 2 miles N of Plaisance, inside helicopter hangar, 2 juvenile females, E. N. Kjellesvig-Waering col., 20 December 1964 (AMNH TR182); under coconut husks, 2 males, E. N. Kjellesvig-Waering col., 22 March 1964 (AMNH TR157); 2 m elevation, 1 male, E. N. Kjellesvig-Waering col., 22 April 1964 (AMNH TR134); ex House, 2 m, 1 male, E. N. Kjellesvig-Waering col., 1 November 1964 (AMNH TR171); 1 juvenile female, E. N. Kjellesvig-Waering col., 5 September 1965 (AMNH TR167); 1 male, E. N. Kjellesvig-Waering col., 10 June 1966 (AMNH TR174); 3 m, 1 male, E. N. Kjellesvig-Waering col., 5 January 1966 (AMNH TR152); *Saint George*: Carenage [10°41'N, 61°35'W], 1 juvenile female, 29 April 1966, E. N. Kjellesvig-Waering col. (AMNH TR177); Nariva Swamp, bush bush forest [10°22'N, 61°02'W], Trinidad Regional Virus Lab., 1 juvenile female, T. H. G. Aitken col., 20 August 1962 (AMNH TR51); 1 male, T. H. G. Aitken coll., ex Boar, 12 February 1964 (AMNH TR156); 2 males, T. H. G. Aitken col., 29 September 1963 (AMNH TR132); Saddle Road (W61°, N10°), 1 juvenile T. H. G. Aitken col., May 1964 (AMNH TR166); *Sangre Grande*: Brigand Hill [10°29'N, 61°04'W], 1 juvenile female, L. N. Sorkin col., 21 July 1979, shed 31 July 79, 7 March 80, 10 February 81 and died 28 May 82 (AMNH TR141); Mount Harris [10°29'N, 61°06'W], 1 male, M. Nieves col., July 1959 (AMNH TR168); 1 male, M. Nieves col., June 1959 (AMNH TR151); 1 male, M. Nieves col., July 1959 (AMNH TR135); 1 female, 1 immature, M. Nieves col., July 1959 (AMNH TR145); *Siparia*: Fyzabad [10°10'N, 61°32'W] (Fyzbad [*sic*]), Bamboo stump, no. 226, 1 female, T. S. Jones col., 21 February 1945 (AMNH TR172); GUYANA: no further information, 1 female, 1914 (CAS13); 1 female, 2 immature males, 1914 (CAS12); *Berbice Oriental-Corentyne*: Canje (5°70'N, 57°50'W), Ikuruna River, forest savanna, 2 males, August−December 1961, G. Bentley col. (AMNH BG78, BG79); Lonsdale Village [6°11'N, 57°31'W] (Larsdale Village [*sic*]), 26 spiderlings, 19 juveniles, 2 females, E. B. Berbice col., 9 September 1946 (AMNH BG83); *Cuyuni-Mazaruni*: Bartica [6°24'N, 58°37'W], 15 milles above, Rio Moraballi and Rio Esequibo, 1 immature, R. W. G. Hingston col., labeled as *Avicularia nigrotaeniata* n. 43 (BMNH 1930.4.15.53); Kartabo [6°24'N, 58°37'W], 2 males, 1 female, captured in a small bush (AMNH BG42); 1 female, 1924 (AMNH BG73); 1 male, 1924 (AMNH BG93); 2 males, 1 female, Beebe col., “*Avicularia surinamensis*” det. Di Caporiacco (AMNH BG67); *Demerara-Mahaica*: Georgetown [6°48'N, 58°09'W], 1 small female, 7 immatures, J. Moral col., 1954 (AMNH BG82); 2 males, L. van Sertima col., 7 May 1991 (AMNH RW65, RW66); 2 immatures, A. McKee col., 19 April 1986 (AMNH RW64); *Potaro-Siparuni*: Tumatumari [5°15'N, 59°08'W], 1 male, 1 female, 5 August 1913, Acc 4435 (AMNH 1.9); 1 immature, August 1913, Acc: 4435 (AMNH 1.11); *Upper Takutu-Upper Essequibo Superior*: Dadanawa [2°50'N, 59°31'W], Ishertor, 5 females, 2 immatures, in webbed holes in ground, Terry-Holden Expedition, 7–9 November 1937 (AMNH BG61); 2 females, 2 immature males, 1 immature, 1 spiderling, 7–9 November 1937, Terry–Holden Expedition, Snedigar & Hassler col. [hand note: original label and part of material in another jar] (AMNH BG76); SURINAME: no further information, 1 female, Box 306 3739 (MNHN–AR 4887); in house, 1 female, Geiskes col. (AMNH Su62); 1 male, Box 306 3739 (MNHN–AR 4887); 1 female, C. Heller col., December 1908, jar no.1632/08 (ZMB); *Paramaribo*: Paramaribo [5°49'N, 55°10'W], 1 male, Reed col., 29 June 1910, Acc 3792 (AMNH Su60); 2 females, H. Heyde col., 30 July 1980 (AMNH RW61, RW62); 1 female, 1 immature, 7 March 1939, Geiskes col. (AMNH Su59); 1 female, C. Heller col., 16 June 1908, jar no.1136/08 (ZMB); Agronomic Station, 1 male, V. Doesburg col., August 1962 (AMNH Su101); *Marowijne*: Langamankondre [5°42'N, 54°01'W], 1 female, B. Malkin col., 15–30 August 1965 (MZUSP 10861); 7 females, 1 male, 5 immature males, 8 juveniles, 1 spiderling, B. Malkin col., 15–30 August 1965 (AMNH Su97); *Para*: no further information, 1 female, C. Heller col., 13 March 1909, jar no. 541/09 (ZMB); 1 female and 1 immature male, C. Heller col., 10 March 1909, jar no. 541/09 (ZMB); 1 female, C. Heller col., February 1908, jar no. 684/08 (ZMB); *Brokopondo*: Kabelstation [4°54'N, 55°08'W] Riheroever [?, unreadable], in bromeliad, 1 juvenile female, Gerskes col., 25 September 1938 (AMNH Su63); in a house, 1 female, Gerskes col., 26 September 1938 (AMNH Su62); FRENCH GUIANA: *Cayenne*: Camopi [3°09'N, 52°20'W], Yanioré, high Oyapock, 100 to 150 m, en amont de Saut, 1 male, Mission E. Aubert de La Rüe col., February 1949 (MNHN–AR 4886); Cayenne [4°55'N, 52°19'W], 1 female, “*Avicularia anthracina*” det. E. Simon, Milinon (MNHN–AR 155h-77); 1 male, “*Avicularia anthracina*” det. E. Simon (MNHN–AR 1719-79); 1 male, J. Moonen col., February 1992 (AMNH RW60); Table Du Mahury [4°52'N, 52°16'W], 1 female, J. Geay col.; 1902 (MHNP 3089); Montabo [4°56'N, 52°18'W], (Bordat Montabo [*sic*]) 1 immature male, Expedição Instituto Butantan col., 4 July 1955 (IBSP 3458); 1 female, Expedição Instituto Butantan col. (IBSP 3379); 1 female, Dr. Hoge col., 5 July 1951, in nest (IBSP 3377); Montsinery [4°53'N, 52°30'W], Emerald Jungle Village, 1 female, 3 males, J. Moonen col., 12 August 1998 (AMNH RW57, RW56, RW58, RW59, respectively), RW59 and RW58 on lodge roof beam; 1 female, R. C. West col., April 1999 (AMNH RW55); Saint-Georges-de-l’Oyapock [3°53'N, 51°48'W] (Oyapock [*sic*]), 1 male, F. Geay, 1900 (MNHN–AR 1293); Sinnamary [5°22'N, 52°57'W] (Linamany [*sic*]), 1 female, 1 male, 1 juvenile female, Expedição Instituto Butantan col. (IBSP 3396, IBSP 3398, IBSP 3397); *Saint Laurent du Maroni*: Saint Laurent du Maroni [5°29'N, 54°01'W] (du Maroni [*sic*], ?), 1 female, Gambey col. (MNHN–AR 4901); Saint Jean [5°24'N, 54°4'W] (Saint-Jean-du-Maroni [*sic*]), 1 male, 1 spiderling, R. Benoit col., March–April 1914 (MNHN–AR 4896); BRAZIL: *Amapá*: no further information, 1 male, F. Baia col., ref. 65469 (IBSP 8846); 2 males, 2 females, Dr. Hoge col., 4 August 1966 (IBSP 3760); Macapá [0°02'N, 51°04'W], Fazendinha, 1 male, 1 immature male, Dr. Hoge col., 4 August 1965 (IBSP 3736); 1 juvenile female, P. Magno col., October 1997 (MNRJ 12923); 1 male (MNRJ 12912); 6 males, C. Costa, P. Magno & C. A. Júlio col., 20 August 1996 (MNRJ 13659); Parque Zoobotânico (IEPA), 2 immatures, P. Magno, C. E. Alvarenga & C. Costa col., October 1995 (MNRJ 13653); Pacoval [0°33'N, 51°03'W], 1 male, Dr. Hoge col., 21 January 1966 (IBSP 3755); 1 female, 2 juvenile females, Dr. Hoge col., 26 September 1966 (IBSP 3795, IBSP 3797); Serra do Navio [1°38'S, 52°16'W], 1 male (IBSP 3685); 3 males, K. Lenko col., 8 November 1957 (IBSP 3490); Paredão Pacoval, 1 male, Dr. Hoge col., 4 March 1965 (IBSP 3704); 1 female, March 1985 (MNRJ 13526); 1 juvenile female, 7 immatures, 1 spiderling, March 1985 (MNRJ 13597); Laranjal do Jari [1°05'N, 53°13'W], Cachoeira do Santo Antônio, Rio Jari, 2 juvenile females, J. A. P. Barreiros col., 18–24 February 2003 (MPEG 0174); *Amazonas*: Fazenda São Francisco, 300 km subindo o Rio Negro, 1 male, G. Ilutseh col., 27 May 1982 (IBSP 4814); *Pará*: no further information, 1 female, J. C. Branner col., 1881 (CAS); Altamira [3°11'S, 52°12'W], trilha caverna clinas, Ponto 2, 1 female, A. P. L. Giupponi & D. Pedroso col., 10 April 2009 (MNRJ 13993); Ponto 2 , Cararaô, 2 females, A. P. L. Giupponi & D. Pedroso col., 10 April 2009 (MNRJ 13994, MNRJ 1400); Ponto 5, Paratipuã, 1 female, A. P. L. Giupponi & D. Pedroso col., 12 April 2009 (MNRJ 13995); Acampamento do Juruá, 2 females, 2 immatures, A. J. Cardoso, C. F. B. Haddad & M. Gordo col., 03–18 December 1986 (ZUEC); Ananindeua [1°21'S, 48°22'W], 1 female, R. F. da Silva col., 30 January 1975 (MPEG 5399); BR 316, Km 06, R. F. da Silva col., 1 male, 10 November 1996 (MPEG 5127); Socêgo [*sic*], Bacia do Sol, 1 female, 1 immature, March 1956 (IBSP 3781); Barcarena [1°34'S, 48°35'W], Ilha das Onças, Rio Piramanha, 2 males, E. Santos col., 19–20 April 2003 (MPEG 0172); Belém [1°27'S, 48°30'W], 3 females, 1 juvenile female, P. Cerveira col., refs 63979, 63999 (IBSP 8849, IBSP 8854, IBSP 8853, IBSP 8855, respectively); 1 juvenile female, Eq. Resgate de Fauna col., ref. 55334-3 (IBSP 8571); 1 female, 2 males (IBSP 3578); 1 immature male, 4 females (IBSP 3772); 1 male, 2 females and 1 immature (IBSP 3780); 1 male, 1 female, 15 June 1966 (IBSP 3770); 3 females, 1 immature, 15 June 1966 (IBSP 3771); 2 females, 15 June 1966 (IBSP 3779); 1 male, R. Cerqueira col., 1972 (ZUEC); 1 male, F. Lima col., 18 April 1989 (MCP 2702); 1 immature female, W. França col., 30 March 1987 (MCP 2697); 1 male, 2 January 2003 (MPEG 1108); C. Galeno col., 1 immature, June 1998 (AMNH RW14); Bairro de Souza, 1 male, L. M. Cunha col., 07 March 2005 (MPEG 5147); Base Aérea, 1 male, 1 female (IBSP 3687); Bragança, 1 immature, 15 June 1966 (IBSP 3777); *campus* de Pesquisa, 1 juvenile, J. Dias col., 30 March 1992 (MCP 2695); CPATU (em dendê), 2 males, Izaías col., 15 February 1984 (MPEG 135); Empresa Brasileira de Pesquisa Agropecuária (EMBRAPA), 1 female, R. F. da Silva col., 03 November 1977 (MPEG 5089); EMBRAPA in dendê area, 1 female, 3 juveniles, 1 spiderling, October–November 1981, H. N. da Cunha col. (MNRJ 13012); Instituto Agronômico do Norte (IAN), 1 immature male, 1 female, 4 immatures, A. R. Hoge col., 29 October to 5 December 1958 (IBSP 3672); IPEAN, 1 male, J. Bushell col., 23 May 1967, Exline–Peck collection (CAS); Mocambo, 1 female, R. F. da Silva col., 14 November 1977 (MEPG 3074); Mocambo, Parque Regional de Manutenção, Parque Ambiental de Belém, CPPTU, 1 male, M. A. S. de Azevedo & J. N. Ferreira col., 31 March 2005 (MPEG 5155); Serraria, IPAN, 1 male, P. Waldir col., 06 December 1966 (MPEG 5107); Universidade Federal Rural da Amazônia (UFRA), 1 female, W. C. Carrasco col., 08 July 2004, F18 A43 B3 (MPEG 5295); 1 juvenile female, Mara col., 15 June 2005, F4 A18 B3 (MPEG 14221); 1 female, 14 June 2004 (MPEG 5188); Vila Ajutei, 1 male, Augusto Côrrea col., 14 July 2002 (MPEG 0170); Museu Paraense Emílio Goeldi, *campus* de pesquisa [01°27'S, 48°26'W], 1 male, W. França col., 04 June 1984 (MPEG 5120); 1 male, Jarilson col., June 2009 (MPEG 5299); 1 male, J. Dias col., 16 June 1986 (MPEG 5126); 1 female, D. F. Candiani col., April 2006 (MPEG 5300); 1 female, M. C. Santos-Costa col., 02 March 2005 (MPEG 5221); 1 male, L. S. Carvalho col., 18 Abril 2006 (MPEG 5098); 1 male, A. B. Bonaldo col., 24 July 2002 (MPEG 227); 1 male, D. F. Candiani col., 22 Abril 2004 (MPEG 5094); 1 male, 08 April 2002 (MEPG 5117); 1 male, 31 March 2009 (MPEG 5632); 1 male, 11 April 2005 (MPEG 5141); 2 males, D. F. Candiani col., 12 July 2004 (MPEG 5281); 1 male, A. B. Bonaldo col., 12 July 2002 (MPEG 160); 2 immatures, 25 May 1912, Bluntschli-Peyer coll. (AMNH 1.27, AMNH 1.3); 1 immature, Bluntschli-Peyer col., 25 June 1912, (AMNH 1.18); Icoaraci [1°18'S, 48°28'W], 1 male, R. F. da Silva col., 10 March 1977 (MEPG 3082); 1 juvenile female, R. F. da Silva col., 05 July 1984, (MPEG 5131); 1 male, R. F. da Silva col., 8 July 1977 (AMNH RW15); 1 male, R. F. da Silva col., 18 May 1977 (AMNH RW18); 1 female, R. F. da Silva, 8 April 1977 (AMNH RW16); 1 male, R. F. da Silva col., 8 July 1977 (AMNH RW17); Outeiro [1°16'S, 48°28'W], 1 juvenile female, R. F. da Silva col., 13 July 1978 (MEPG 5096); Universidade Federal do Pará (01°28'S, 48°27'W), 1 female, C. Castro col., 24 June 2005 (MPEG 4820); 1 male, L. T. Miglio col., 15 September 2002 (MPEG 1109); Utinga [1°25'S, 48°24'W], 1 immature, Oliveira & Wygodzinsky col., 10–21 November 1963 (AMNH 1.2); Belém, Ilha de Cotijuba (01°14'S, 48°35'W), 1 female, 27 August 2003 (MPEG 4701); 1 female, Cotijuba 0011 (MPEG 5291); 1 female, R. F. da Silva col., 17 March 1977 (MPEG 3073); 1 female, R. F. da Silva col., 26 December 1977 (MEPG 3030); 1 male, R. F. da Silva col., 11 September 1977 (MPEG 3056); 1 male, R. F. da Silva col., 11 September 1977 (MPEG 3057); Icoaraci,1 female, 01 July 2002 (MPEG 1111); Belém, Ilha de Jutuba [1°14'S, 48°31'W], 2 males, 3 females, R. F. da Silva col., 30 June 1977 (MPEG 3047, MPEG 3046, MPEG 3045, MPEG 3034, MPEG 3033, respectively); Belém, Ilha do Mosqueiro [1°06'S, 48°23'W], 1 female, 1 immature male, P. Cerveira col., December 1990, ref. 63979 (IBSP 7884, IBSP 7883); 1 male, M. L. Mocambira col., 15 December 1989 (MCP 2699); 2 juvenile females, B. Mascarenhas & team col., 19–25 April 1998 (MPEG 5392, MPEG 5395); Belém, Ilha Nova [2°12'S, 49°27'W], 2 males, 2 females, R. F. da Silva col., 20 June 1977 (MPEG 3040, MPEG 3043, MPEG 3071, MPEG 5090); 1 male, R. F. da Silva col., 24 June 1977 (MPEG 3041); Belém, Ilha de Paquetá [1°16'S, 48°32'W], 2 females, R. F. da Silva col., 15 November 1977 (MPEG 3025, MPEG 3023); 1 juvenile female, R. F. da Silva col., 28 May 1977 (MPEG 5088); 1 male, R. F. da Silva col., 15 September 1977 (MPEG 3052); 1 male, 1 female, R. F. da Silva col., 19 June 1977 (MPEG 3078, MPEG 3062); 1 male, R. F. da Silva col., 15 May 1977 (AMNH RW21); 1 male, R. F. da Silva col., 20 May 1977 (AMNH RW22); 1 immature male, R. F. da Silva col., 15 November 1977 (AMNH RW20); 1 juvenile female, R. F. da Silva col., 26 November 1977 (AMNH RW19); Belém, Ilha de Tatuoca [1°12'S, 48°30'W], 1 female, 23 March 1978 (MPEG 5105); 2 males, EPA col., August 1969 (MZUSP 10849); Belém, Ilha de Urubuoca [1°19'S, 48°27'W], 1 female, 1 male, R. F. da Silva col., 28 July 1977 (MEPG 3036, MPEG 3313, respectively); Belterra [3°08'S, 55°03'W], 1 male, 18 December 2003 (MPEG 4703); Rio Assuá [?, unreadable], 1 female, H. Sioli col., May 1952 (IBSP 3130); Benevides [1°21'S, 48°14'W], Pratinha, Estrada do Açucareiro, 1 female, O. Cunha col., 19 February 1975 (MPEG 141); Breves [1°40'S, 50°28'W], Corcovado, 1 male, Expedição CDZ col., 18–19 October 1965 (IBSP 5688); Castanhal [1°17'S, 47°55'W], 1 female, O. Feassi col., Ref. 52599 (IBSP 8863); Ilha de Marajó [0°58'S, 49°35'W], 1 male, 1 female, 16 immatures, Instituto Agronômico col., 15 June 1966 (IBSP 3787, IBSP 3786, respectively); Santo André, 1 male, June–September 1912, Bluntschli-Peyer (AMNH 1.23); Cachoeira do Arari [1°0'S, 48°57'W], Jabuti, 1 female, L. Macambira col., 12 December 1990 (MPEG 0138); Itaituba [04°16'S, 55°59'W], 1 female (MZUSP 27659); Itupiranga [5°08'S, 49°19'W], campus avançado da UFPA, 3 females, 2 immatures, A. R. Hoge & P. Villela col., December 1971 (IBSP 4097); Jacareacanga [07°15'S, 57°26'W], 1 immature male (IBSP 3538); (Jacare-acanga [*sic*]), 1 spiderling, M. Alvarenga col., December 1968 (AMNH 1.20); Aproeste, km 350 da Transamazônica, 1 female, B. V. Rodrigues & N. Abrahim col., 27 October 2009 (MPEG 15640); Jatobal [5°08'S, 56°51'W], 3 males, January 1975 (IBSP 7896); Juruti [2°09'S, 56°05'W], Ferrovia Km 07, 1 immature female, A. C. Lima & F. E. Pimenta col., 17 Maio 2007, JURU 006 0286 (MPEG 15635); Ferrovia km 36, 1 female, A. Lima & F. Pimenta col., 24 May 2007, JURU 0060268 (MPEG 15636); Platô Capiranga, Linha 168E (02°28'22.1"S, 56°12'29.4"W), 1 male (MPEG 15639); Juruti, Acampamento Barroso (02°28'10.5"S, 56°00'3.5"W), 1 male, D. F. Candiani col., 07 August 2004, ref. JURU002 0044 (MPEG 2000); 2 males, 1 female, D. R. Santos-Souza col., 12 July 2002, ref. JURU 002 0084 (MPEG 2003, MPEG 2006, respectively); 1 female, D. F. Candiani col., 12 July 2002, ref. JURU 002 0083 (MPEG 2009); Juruti, Acampamento Mutum (01°36'44.77"S, 56°11'39.2"W), 1 juvenile female, 1 immature male, D. F. Candiani and D. R. Santos-Souza col., 03 August 2004, ref. Juruti 2004 (MPEG 15634); Marabá [5°22'S, 49°07'W], Transamazônica, 3 males, J. Navas col., 15 March 1975 (IBSP 4190); 1 female, M. F. Torres col., 14 September 1985 (MPEG 4231); 1 male, P. Tumma col., 8 October 1973 (IBSP 2495); Melgaço, Estação Científica Ferreira Penna, FLONA Caxiuanã [2°01'S, 51°39'W], 1 female, A. B. Bonaldo col., 21–30 November 2000 (MPEG 1923); Mocajuba [2°34'S, 49°30'W], Praia do Imbaubal, 1 male, 17 June 1984 (MPEG 5104); Monte Alegre [1°59'S, 54°04'W], Lagoa Grande, 1 immature, P. de Biasi col., 10 March 1979, ref. 24.434 (IBSP 4408); Óbidos [1°54'S, 55°31'W], 1 female, E. Garbe col., 1920 (MZUSP 10856, old collection number 556); Oriximiná [1°45'S, 55°51'W], 1 immature male, 1 March 1968, EPA col. (MZUSP 10850); 1 immature, in pineapple, EPA col., 13 January 1968 (MZUSP 10854); Porto Trombetas [1°28'S, 56°22'W], 1 male (IBSP 8847); Ourém [1°29'S, 47°10'W], Patauateua, 1 juvenile female, D. D. Guimarães col., 06 December 2002 (MPEG 213); Paragominas [3°00'S, 47°21'W], Fazenda Brejeiro, 1 male, C. Junqueira Netto col., 5 October 1970, ref. 5741 (IBSP 84); Canindé [2°33'S, 46°30'W] (Canindé, Maranhão [*sic*]), 1 immature female, May to August 1965, J. Carvalho col. (AMNH 1.16); Rio Cuminá Mirim [1°14'N, 55°52'W], As Pedras, 1 female, 1 immature, EPA col., 29 September to 4 October 1969 (MZUSP 1085); Rio Tocantins [02°04'S, 49°18'W], Ilhas das Cobras, Castanheira, 1 female, A. J. da Silva col. (MPEG 5100); Rio Trombetas [1°28'S, 56°22'W], 3 males, 2 females, 1 immature male, F. Palinger col., (IBSP 8762, IBSP 8761, IBSP 8759, respectively); Jacaré, 3 males, female, Expedição CDZ col., 20 September to 13 October 1965 (IBSP 5687); Santarém [2°26'S, 54°41'W], 1 female, E. Garbe col. (MZUSP 10864, old collection number 557); 1 male, G. Puorto col., December 1996 (IBSP 8578); Fazenda Taperinha, 1 female, EPA col., 1–11 February 1968 (MZUSP 10859); 1 female, 1–11 February 1968, E.P.A. col. [handwritten label], or Manaus, Lenko col., 12 September 1962, forest [museum label] (MZUSP 10858); Santo Antônio do Tauá [1°09'S, 48°07'W], Uxitena, 1 male, R. F. da Silva col., 07 April 1977 (MPEG 3076); Tomé-Açu [2°25'S, 48°09'W], Roda D’Água (capoeira), 1 female, J. Dias col., 19 June 1994 (MEPG 143); UHE Tucuruí [3°46'S, 49°40'W], Equipe de Resgate de Fauna col.: 2 males, 1 female, ERF col., 1984, IBA 243 (IBSP 7898); 1 female, ERF col., ref. 48286 (IBSP 8845); 1 female, ERF col., 1984, ref. IBA 383 (IBSP 7880); 1 male, ERF col.,1984, ref. IBA 1002 (IBSP 7879); 1 female, ERF col., 1984, ref. 47607 (IBSP 7882); 2 females, ERF col., 1984, ref. IBA 283 (IBSP 7887); 1 female, ERF col., ref. IBA 600 (IBSP 8851); 1 female, ERF col., ref. CB-1 (IBSP 8568); 1 female, ERF col., (IBSP IBA 380); 1 female, born in capitivity, died in 06 July 1993 (IBSP IBA 083-43); 1 female, ERF col., IBA 599 (IBSP 8850); 1 female, ERF col., 12 December 1984 (IBSP 4832); 1 female, ERF col., ref. 34C XXXV (IBSP 8857); 1 male, ERF col., ref. 48286 (IBSP 8848); 1 male, ERF col., ref. 47607 (IBSP 8573); 1 male, ERF col., 18 November 1986, IBA 860 (IBSP 4925-B); 2 males, ERF col., ref. CB-1 (IBSP 8579); 1 male, ERF col.(IBSP 8575); 2 females, ERF col. (IBSP 8843); 1 male, ERF col., ref. B5677 (IBSP 8580); 1 male, ERF col., 4 June 1987, IBA 277 (IBSP 8566); 1 female, ERF col., 1984, ref. IBA 283 (IBSP 7888); 1 female, ERF col., August 1984, ref. IBA 734 (IBSP 7877); 1 female, ERF col., ref. IBA 283 (IBSP 8862); 1 male, in bromeliad in a tree (21 m from the soil), C. Pantoja col., 28 June 1984 (IBSP 7916); 1 male, Equipe de Resgate de Fauna col., 1984, IBA 292 (IBSP 7885); 1 male, Equipe de Resgate de Fauna col., 1984, ref. 47607 (IBSP 7881); picada do Inajá, A3, lote 8, 1 female, B. Mascarenhas col., 20 June 1980 (MZUSP 9480); Base 4, Bravo, 1 female, Operação Resgate Faunístico col., 12 December 1984 (IBSP 4832); Vila Bravo, 1 female, Equipe de Resgate de Fauna col., ref. XXXVII-87 base 4 (IBSP 8844); Vila Cisipé, 1 male, Equipe Resgate de Fauna col., ref. 47607 (IBSP 8572); Ilha de Tocantins, Equipe de Resgate de Fauna col.: 1 female, ref. IBA 389 (IBSP 8861); 1 female, ERF col., ref. IBA 599 (IBSP 8569); 1 female, 2 June 1986, ERF col., ref. IBA 1013 (IBSP 8852); 1 female (IBSP ref. IBA 283); Breu Branco [3°45'S, 49°33'W], 1 male, died 20 February 1986 (IBSP CB1 - XV); 1 male, Equipe de Resgate de Fauna col., ref CBI V (IBSP 8565); Vigia [0°51'S, 48°08'W] (Vigia de Nazaré [*sic*]), Hospital UBSO IV, 1 male, 03 July 2005 (MPEG 5189); UBS V, 1 male, J. F. Maia col., 30 August 2005 (MPEG 5144); *Maranhão*: São Luís [2°31'S, 44°18'W], 1 female, G. R. Ristan Filho col., 29 February 1996, ref. 78226 (IBSP 8244); 1 female, ref. 78226 (IBSP unnumbered); Bacabal [4°13'S, 44°47'W], Lago Verde, 1 female, R. Neto col., 03 February 1983 (MPEG 5106); Palmeirândia [2°40'S, 44°54'W], 1 female, Dr. Hoge, Pedro and Joaquim col., 7–30 May 1962 (IBSP 3622); Fazenda São Luís, 1 female, 1 immature male, Dr. Hoge, Pedro & Joaquim col., 7–30 May 1962 (IBSP 3621); São Bento [2°41'S, 44°49'W], 1 immature male, 1 female, Dr. Hoge, Pedro & Joaquim col., 7–30 May 1962 (IBSP 3623, IBPS 3618); *Mato Grosso*: Alta Floresta [9°52'S, 56°05'W], 1 male, F. Palinger col. (IBSP 8768); PERU: *Madre de Dios*: Cuenca Rio Los Amigos [12°34'S, 70°06'W], 1 male (UA 003/2005); 1 female (UA 042/2006); 1 female (UA 007/2005); Aguajal [12°15'S, 69°16'S], CICRA, 1 female, 21 March 2006 (UA 041/2006); 1 male (UA 005/2005); 1 male (UA 002/2005); Puerto Maldonado [12°36'S, 69°41'W], 15 km East, Rio Madre de Dios, Albergue Lodge Cuzco Amazônico, 200 m, 1 female, G. C. Hunter col, 8 June 1983 (CAS10); La Cachuela (12°33'S, 69°11'W), 1 male and 2 females, H.–W. Auer col., September–October 2013 (MUSM–ENT 0506819); Tambopata [12°34'S, 69°11'W], 1 female, R. Bennett col., August 1994, in silk retreat on palm trees (AMNH RW47); Reserva Tambopata–Candamo [12°59'S, 69°36'W], Albergue Explorer’s Inn, 1 male and 1 female, K. J. N. Villa leg., 2005 (MZUSP 70948); BOLIVIA: *Beni*: Yucumo [15°08'S, 67°02'W], Aserradero Chaparina, 7 km from Yucumo, path to San Borja, prov. J. Ballivian, 1 male, J. Peñaranda col., 9 July 1991 (MNRJ 06918); 1 female, same data (MNRJ 06919); *Pando*: Manuripi, Camalho 15 km northwest Puesto Castañero, Chivé [12°23'S, 68°34'W], bosque alto, 1 female, F. Guerra col., 30 September 1991 (MNRJ 06916); *La Paz*: Iturralde [12°54'S, 67°45'W], Estância El Dorado, UMSA, Instituto de Ecologia, 1 immature male, S. Beck col., 28 February 1984 (MNRJ 06917); *Santa Cruz*: San Antonio del Parapeti [20°01'S, 63°13'W], 1 male, B. Malkin col., 24–25 May 1985 (AMNH Bo01).

##### Female.

**Redescription.** MNRJ 13995. Carapace: 19.39 long, 17.68 wide, 5.5 high. Chelicera: 8.40 long. Legs (femur, patella, tibia, metatarsus, tarsus, total): I: 14.66, 8.55, 11.65, 10.56, 8.25, 53.67. II: 14.04, 8.15, 10.56, 10.22, 3.55, 46.55. III: 13.55, 8.20, 10.55, 10.66, 3.11, 46.01. IV: 17.56, 8.65, 14.65, 13.66, 8.12, 62.64. Palp: 10.42, 8.65, 8.76, -, 8.62, 36.45. Midwidth: femora I–IV= 3.45, 3.21, 4.05, 3.50, palp= 2.56; patellae I–IV= 3.66, 3.55, 3.15, 3.50, palp= 3.05; tibiae I–IV= 3.37, 2.56, 3.26, 3.17, palp= 2.62; metatarsi I–IV= 2.45, 2.35, 2.40, 2.35; tarsi I–IV= 2.61, 2.65, 2.53, 2.30, palp= 2.55. Abdomen: 26.37 long, 17.56 wide. Spinnerets: PMS, 2.99 long, 1.62 wide, 0.1 apart; PLS, 2.04 basal, 1.71 middle, 4.07 distal; width 2.73, 2.3, 1.72, respectively.

Carapace: 1.10 times longer than wide; cephalic region slightly raised, thoracic striae inconspicuous.

Fovea: deep, straight, 2.27 wide.

Eyes: eye tubercle 1.30 high, 2.62 long, 3.52 wide. Clipeus: 0.53. Anterior eye row procurve, posterior slightly recurve. Eye size and interdistances: AME 0.73, ALE 0.85, PME 0.26, PLE 0.74, AME–AME 0.57, AME–ALE 0.48, AME–PME 0.27, ALE–ALE 2.35, ALE–PME 0.58, PME–PME 2.15, PME–PLE 0.14, PLE–PLE 2.86, ALE–PLE 0.38; AME–PLE 0.60.

Maxilla: length to width 1.86. Cuspules: 164 spread over ventral inner heel. Labium: 2.07 long, 3.46 wide, with 82 cuspules spaced by one diameter from each other on anterior third. Labio-sternal groove swallow, flat, with two slightly separate, large sigilla.

Chelicera: basal segment with 11 teeth in row and some small teeth on promargin. Sternum: 9.07 long, 8.08 wide. Sigilla: three pairs, posterior and middle rounded, anterior small, all less than one diameter from margin.

Legs: Formula: IV I II III. Length leg IV to leg I: 1.16. Clavate trichobothria: 2/3 distal tarsi I–IV. Scopulae: Tarsi I–IV fully scopulate; IV with a few sparse setae. Metatarsi I–II fully scopulate; III 2/3 distal; IV 1/3 distal. IV divided by rows of setae.

Type II urticating hairs: 0.62–0.69 long, 0.017–0.019 wide.

**Figures 21–27.**
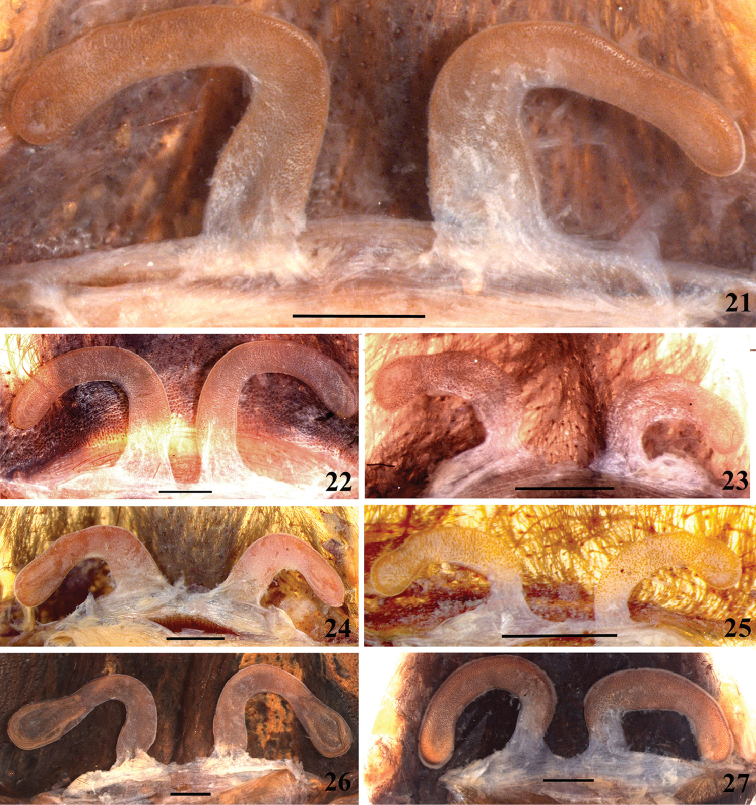
*Avicularia avicularia* (Linnaeus, 1758), spermathecae variation. **21** morphotype 1, Altamira, state of Pará, Brazil (MNRJ 13995) **22** morphotype 2, UHE Tucuruí, state of Pará, Brazil (IBSP 4885) **23** morphotype 3, Puerto Cabello, state of Carabobo, Venezuela (MNHN–AR 4883) **24** morphotype 4, Cuenca Rio Los Amigos, department of Madre de Dios, Peru (UA 0042/2006) **25** morphotype 5, Yucumo, department of Beni, Bolivia (MNRJ 06919) **26** morphotype 6, Paramaribo, district of Paramaribo, Suriname (AMNH Su59) **27** morphotype 7, Juruti, state of Pará, Brazil (MPEG 15640). Scale bars = 1 mm.

Spermathecae (Fig. 21): two completely separated, not-twisted and long spermathecae, with walls lacking projections or lobes and accentuated outwards curvature medially. Midwidth as wide as its base width and weakly-sclerotized area shorter than half the length of well-sclerotized area.

Color pattern (Fig. 36): carapace brown with brown short body setae with green sheen. Carapace border with long reddish brown setae with pink sheen. Coxae, labium, sternum and maxillae brown, slightly darker than ventral femora. Legs and palps with brown short body setae with green sheen and reddish brown guard-setae with homogeneous dark coloration on anterior legs and guard-setae with darker base and contrasting whitish apex on posterior legs. Leg rings on distal femora, tibiae and metatarsi whitish. Abdomen dorsum with long reddish brown guard-setae with pink sheen grouped on lateral dorsal anterior areas and dark short body setae. Ventral abdomen brown.

##### Male.

**Redescription**. MNRJ 13659A. Carapace: 17.23 long, 16.81 wide, 4.9 high. Chelicera: 7.2 long. Legs (femur, patella, tibia, metatarsus, tarsus, total): I: 15.4, 8.6, 11.8, 11.7, 6.9, 54.4. II: 14.8, 7.7, 11.7, 11.5, 6.7, 52.4. III: 13.2, 6.7, 10.2, 11.5, 6.6, 48.2. IV: 16.2, 7.9, 14.7, 15.6, 6.9, 61.3. Palp: 9.4, 5.3, 7.0, –, 2.9, 24.6. Midwidths: femora I–IV= 3.0, 2.8, 3.4, 3.1, palp= 2.2; patellae I–IV= 3.1, 3.1, 2.8, 3.2, palp= 2.3; tibiae I–IV= 2.3, 2.2, 2.2, 2.6, palp= 2.2; metatarsi I–IV=1.7, 1.6, 1.5, 1.8; tarsi I–IV= 2.0, 1.9, 1.8, 1.6, palp= 2.2. Abdomen: 19.83 long, 13.39 wide. Spinnerets: PMS, 1.75 long, 0.80 wide, 0.1 apart; PLS, 2.82 basal, 2.04 middle, 3.56 distal; width: 1.88, 1.55, 1.15, respectively.

As in female, except:

Carapace: 1.03 times longer than wide; cephalic region slightly raised, thoracic striae inconspicuous.

Fovea: deep, straight, 1.70 wide.

Eyes: eye tubercle 1.60 high, 2.23 long, 3.33 wide. Clypeus 0.22 wide. Eyes size and interdistances: AME 0.71, ALE 0.77, PME 0.27, PLE 0.700, AME–AME 0.62, AME–ALE 0.48, AME–PME 0.22, ALE–ALE 2.24, ALE–PME 0.78, PME–PME 1.99, PME–PLE 0.12, PLE–PLE 2.58, ALE–PLE 0.46, AME–PLE 0.51.

Maxilla: length to width: 2.13. Cuspules: 157 spread over ventral inner heel. Labium: 1.73 long, 2.40 wide, with 94 cuspules.

Chelicerae: basal segment with 10 teeth in row and some small teeth on promargin. Sternum: 8.58 long, 7.35 wide. Sigilla: three pairs, all rounded and large, less than one diameter from margin.

Legs: Length leg IV to leg I: 1.13. Metatarsi I–II fully scopulate, III scopulate in distal 2/3; IV, in distal 1/3. IV divided by wide row of setae.

Type II urticating hairs: 0.94–1.09 long, 0.020–0.025 wide.

**Figures 28–35.**
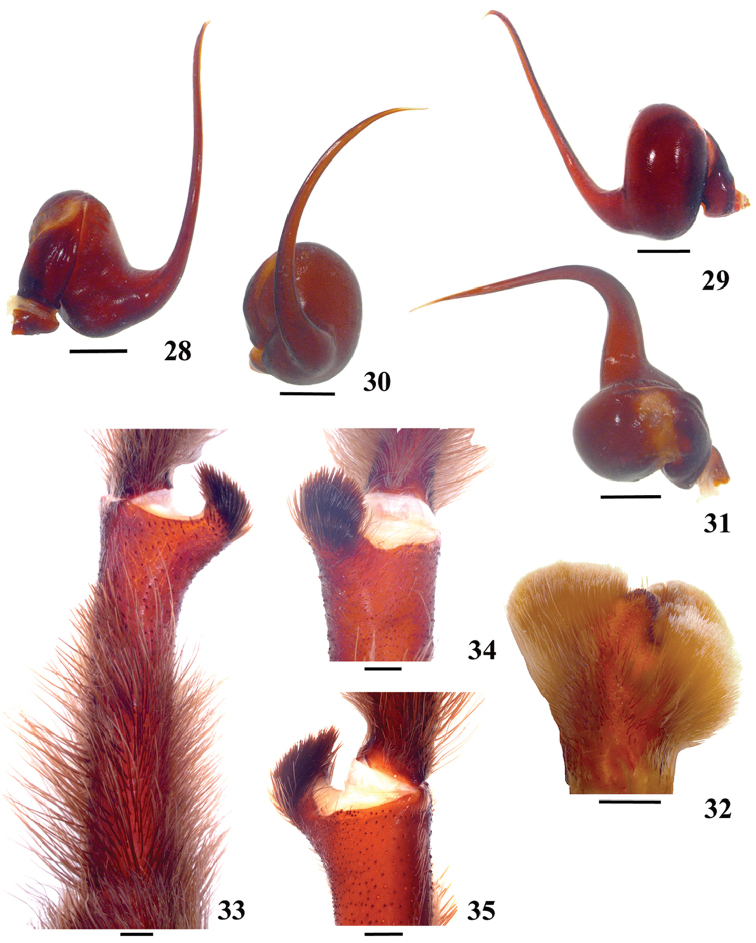
*Avicularia avicularia* (Linnaeus, 1758), male (MNRJ 13659A). **28–31** right palpal bulb (mirrored) **28** prolateral **29** retrolateral **30** frontal **31** dorsal **32** right cymbium, dorsal view (mirrored) **33–35** right tibial apophysis of leg I (mirrored) **33** prolateral **34** ventral **35** retrolateral. Scale bars = 1 mm.

Palp (Figs 28–31): globous bulb with small subtegulum and developed prominence on tegulum. Embolus: not flattened, lacking keels, 4.99 long in retrolateral view, about 3.0 times tegulum’s length. Medial portion and tegulum’s margin form an acute angle in retrolateral view (Fig. 323). Proximal part very curved in frontal view; thin distal width, abruptly narrowing distally; basal, middle, and distal width 0.49, 0.21, 0.07, respectively. Tegulum: 2.66 long, 1.72 high in retrolateral view. Cymbium subtriangular with subequal lobes, with well-developed rounded process on retrolateral lobe, bearing thick setae (Fig. 32).

Tibial apophysis (Figs 33–35): single branch on prolateral leg I, with well-developed base and grouped spiniform setae distally. Male metatarsus I touches retrolaterally tibial apophysis’ setae when folded.

Color pattern: carapace brown with brown short body setae with green and golden sheen. Carapace border with long setae the same color as dorsal carapace short body setae. Coxae, labium, sternum and maxillae brown, slightly darker than ventral femora. Legs and palps with brown short body setae with green sheen and reddish brown guard-setae with homogeneous dark coloration on anterior legs and guard-setae with darker base and contrasting whitish apex on posterior legs. Leg rings on distal femora, tibiae and metatarsi whitish. Abdomen dorsum with long reddish brown guard-setae with pink sheen and dark short body setae. Ventral abdomen brown.

**Figures 36–43.**
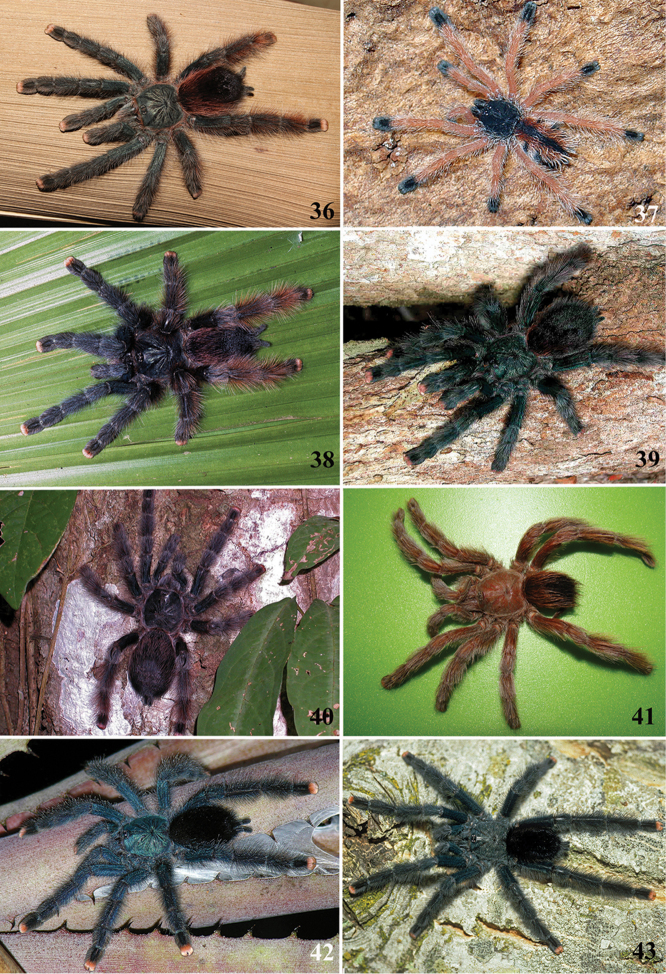
*Avicularia avicularia* (Linnaeus, 1758) morphotypes. **36–37** morphotype 1 **36** female from Castanhal, state of Pará, Brazil **37** immature from Santarém, state of Pará, Brazil **38** morphotype 2, female from Tucuruí, state of Pará, Brazil **39** morphotype 3, female from Caracas, Distrito Capital, Venezuela **40** morphotype 4, female from Tambopata, department of Madre de Dios, Peru **41** morphotype 5, preserved female from Manuripi, department of Pando, Bolivia **42–43** morphotype 6 **42** female from Montsinery, department of Cayenne, French Guiana **43** male from Georgetown, department of Demerara-Mahaica, Guyana. Photos: **36** R. Bertani; **37** M. Gamache, **38** W. Bokermann; **39–40, 42–43** R. C. West; **41** C. S. Fukushima.

##### Color pattern ontogeny.

**Figures 44–49.**
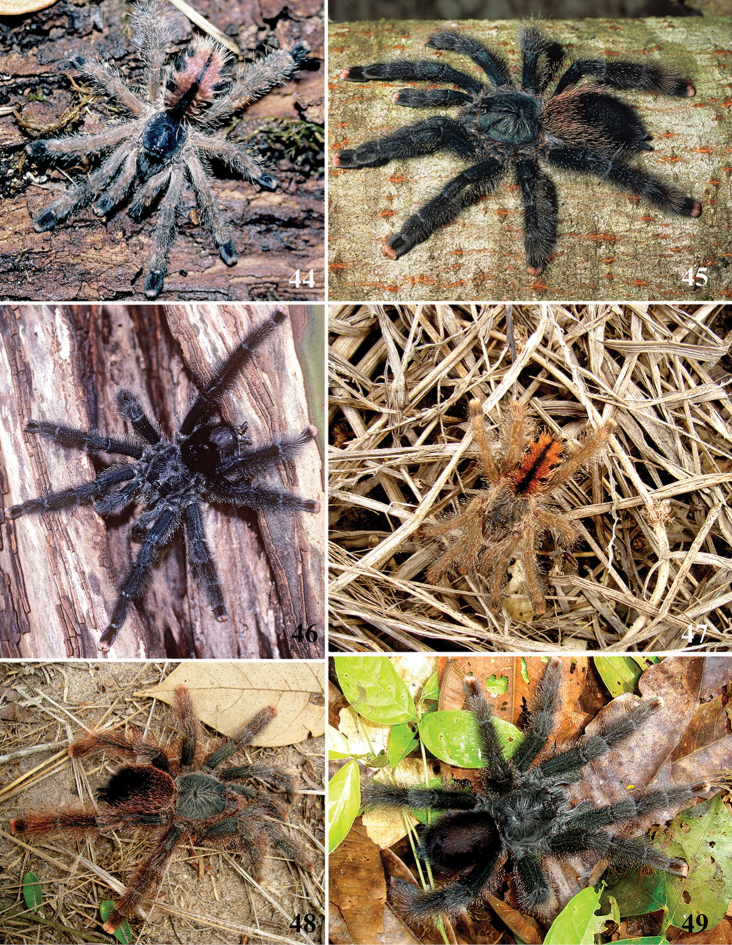
*Avicularia avicularia* (Linnaeus, 1758) morphotypes. **44–46** morphotype 6, from Trinidad and Tobago **44** immature **45** female **46** male **47–49** morphotype 7, from Juruti, state of Pará, Brazil **47** immature **48** female **49** male. Photos: **44–46** R. C. West; **47–49** F. E. Pimenta.

Brownish juveniles lacking metallic sheen, black tarsi contrasting with other lighter articles and abdomen dorsum reddish, with dorsal central longitudinal black stripe disconnected from transversal black stripes (Figs 37, 44, 47). When mature, both males and females lose this pattern.

##### Distribution.

**Figure 50.**
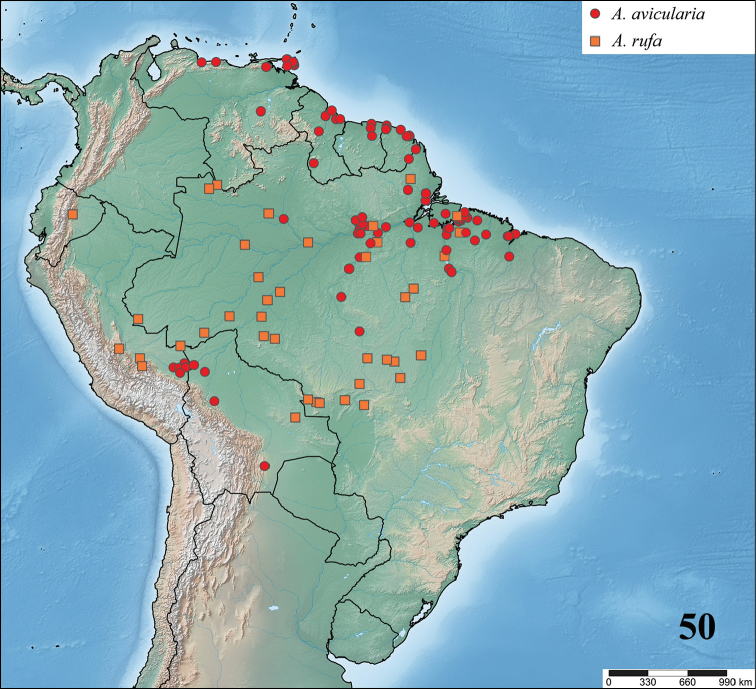
Map showing records of *Avicularia avicularia* (Linnaeus, 1758) and *Avicularia rufa* Schiapelli & Gerschman, 1945.

Venezuela, Trinidad and Tobago, Guyana, Suriname, French Guiana, Brazil (states of Amapá, Pará, Maranhão, Amazonas, Mato Grosso), and populations in Peru and Bolivia (Fig. 50). Possibly, on an opposite pattern to *Avicularia rufa*, *Avicularia avicularia* is rarer in western Brazil, the area connected to the region where occur *Avicularia avicularia* in Peru and Bolivia. This disjunct distribution pattern of *Avicularia avicularia* can be better understand only after sampling or having access to material from poorly sampled areas such as western Amazon (near Brazil-Peru-Colombia border), and Bolivia and Peru as a whole.

##### Natural history.

Stradling (1994) studied distribution and behavioral ecology of a population of *Avicularia avicularia* in Trinidad. The author reported that retreats were found in different situations on vegetation and in human constructions. He found first eight instars specimens predominantly between leaves of low-growing plants (especially in *Heliconia* Linnaeus), holding edges of leaves together with silk to create retreat. After this life stage, Stradling (1994) noted spiders tended to build their retreats in more elevated structures, not incorporating leaves in retreat. Stradling (1994) recorded capture of katydids, cockroaches, scarabs and lizards by *Avicularia avicularia* and also observed courtship behavior with males maturing in beginning of rainy season, in May and June. Females lay the eggsac in May, and after an incubation period ranging from 29 to 62 days, about 125 spiderlings hatch (Stradling 1994).

Bates (1863) observed a scene similar to the one depicted by Merian (1705) in Cametá, state of Pará, Brazil. He observed *Avicularia avicularia* specimen actively feeding on a small bird inside its webbed retreat on a tree, and also the existence of another small bird inside the spider’s retreat.

F. O. Pickard-Cambridge (1896) observed that adults constructed their retreat in “almost any locality which offered a more or less vacant cylindrical space”, including hollow stumps of Açai palm, folded leaves of bananas, in the hollow center of a pineapple plant and among the palm-leaf thatch of native houses.

Villa (2004) observed in areas at Tambopata district in Peru retreats of *Avicularia avicularia* built on trunks and in axils of palm tree *Attalea butyracea* (Mutis ex L.f.) Wess. Boer and also in *Heliconia psittacorum* L. plant. The reproduction period in that area is in September and October (Villa 2004).

**Figure 51.**
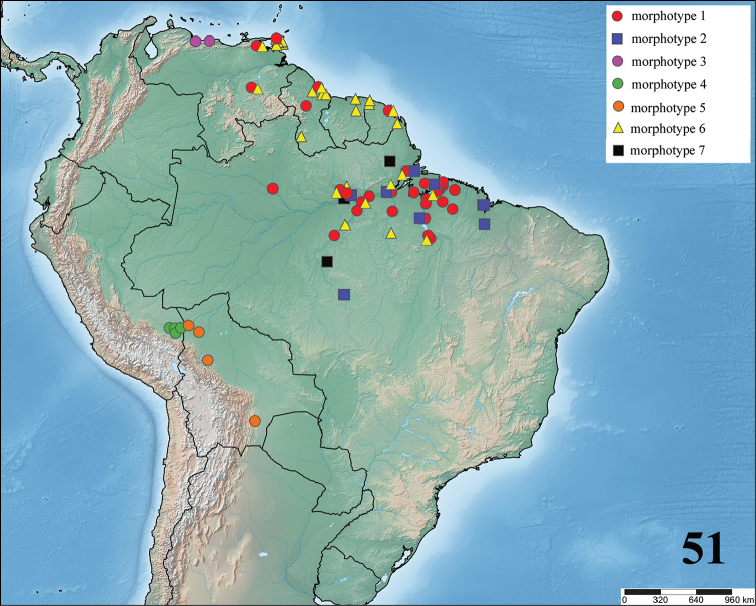
Map showing records of *Avicularia avicularia* (Linnaeus, 1758) morphotypes.

##### Variation.

Specimens of *Avicularia avicularia* show different patterns of coloration which seems to be correlated with their geographical distribution. However, genitalic and somatic characters are very homogeneous along the distribution of the species. Variations among different populations concerning color patterns are, herein, discussed. We do not consider the detected differences sufficient to elevate each morphotype to species status. Nonetheless, we do not discard the possibility of they corresponding to criptic species. In order to establish more accurate limits in *Avicularia avicularia*, it is necessary to employ multiple approaches, considering molecular, ecological, behavioral and geographic data.

Besides body coloration, there is urticating setae variation. Females of morphotype 5 have urticating setae with developed barbs along almost all lengths (Fig. 17), different from the other morphotypes (Fig. 16). However, as we had only access to three specimens it is not possible to make more considerations about it.

**Table 2.** *Avicularia avicularia* morphotypes: characteristics and geographical distribution.

	Morpho 1 (Fig. 36)	Morpho 2 (Fig. 38)	Morpho 3 (Fig. 39)	Morpho 4 (Fig. 40)	Morpho 5 (Fig. 41)	Morpho 6 (Figs 42–46)	Morpho 7 (Figs 47–49)
Occurrence area (Fig. 51)	Brazil, Guyana, French Guiana, Venezuela and Trinidad and Tobago	Brazil: states of Pará, Maranhão and Mato Grosso	Venezuela	Peru: department of Madre de Dios.	Bolivia: departments of Santa Cruz, La Paz and Beni.	Brazil, Guyana, French Guiana, Suriname, Venezuela and Trinidad and Tobago	Brazil: states of Pará and Amapá.
Overall aspect	brownish	brownish	brownish	brown reddish	greyish	greyish	light brownish
Legs and palps guard-setae	discrete grizzled or not grizzled	discrete grizzled or not grizzled	discrete grizzled or not grizzled	not grizzled	discrete grizzled or not grizzled	very grizzled	very grizzled
Legs and palps short body setae	brown with very intense green sheen	brown with discrete golden sheen	brown with golden sheen	brown with green and pink sheen	brown with very intense green sheen	brown with very intense green sheen	brown with discrete golden sheen
Leg rings	whitish	whitish	whitish	whitish	pale yellowish	whitish	whitish
Females: abdomen, guard-setae, color	reddish with pink sheen	reddish brown	reddish brown	reddish brown	reddish	brown, gradually lightening from base to tip	orange brownish, with whitish tip
Females: posterior legs, guard-setae color	vivid red	vivid red	reddish	vivid red	greyish	greyish	vivid orange
Males: abdomen, guard-setae, color	dark brownish	dark brownish	dark brownish	dark brownish	dark brownish	dark brownish, some with whitish tip	dark brownish, some with whitish tip

##### Remark.

Valerio (1979) reported the presence of *Avicularia avicularia* in Costa Rica (Fig. 19). In fact, the male palpal bulb and spermathecae illustrated by the author are compatible with *Avicularia* species. However, the specimens from Costa Rica reported by Valerio (1979) were examined and are more similar to *Avicularia purpurea* than to the larger species of *Avicularia*. See discussion in *Avicularia glauca* taxonomy.

#### Avicularia glauca

Taxon classificationAnimaliaORDOFAMILIA

Simon, 1891

Figs 19 , 52–53

Avicularia glauca Simon, 1891: 312 (holotype female, Panama, MNHN–AR 4897, examined); F. O. Pickard-Cambridge 1896: 744, 1899: 42; Petrunkevitch 1911: 50; Mello-Leitão 1923: 377; Roewer 1942: 254; Bonnet 1955: 831; World Spider Catalog 2016.

##### Remark.

The examined specimen is not labeled as holotype. However, it is the only specimen in Simon’s collection in which locality and size are compatible with the description (Simon 1891). Therefore, we consider it as the holoype.

**Figures 52–53.**
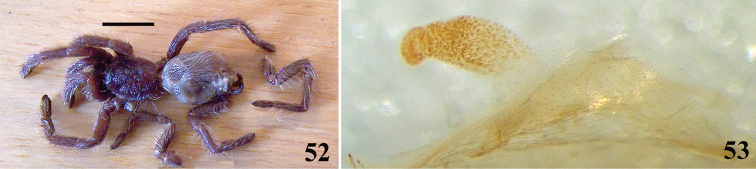
*Avicularia glauca* Simon, 1891, holotype female (MNHN–AR 4897). **52** habitus **53** spermathecae.

The small specimen (carapace length 9.7 mm) has tarsal and metatarsal scopulae expanded, giving a spatulated aspect, characteristic of Aviculariinae (Fig. 52). A single spermatheca is present: the other is supposed lost. It has an accentuated outwards curvature medially and lacks lobes, as most species of *Avicularia* (Fig. 53). Thus, it undoubtedly belongs to *Avicularia*. Its spermatheca has a short, well-sclerotized area that resembles the spermathecae of *Avicularia purpurea*, with which it seems to be very closely related. Furthermore, it has the overall metallic green color exhibited by young and small specimens of *Avicularia purpurea*. The specimens found in Costa Rica and reported by Valerio (1979) as *Avicularia avicularia* were examined by the second author (RB). They are, in fact, definitely not *Avicularia avicularia*. Despite many searches, we did not find any other specimen from Panama or Costa Rica in other arachnological collections, indicating *Avicularia* is a rare genus in southern Central America. Therefore, we conclude that *Avicularia glauca* can be a valid species from Panama and Costa Rica. The identity of Costa Rica’s *Avicularia* will be better discussed in another paper, in preparation with a Costa Rican colleague.

#### Avicularia variegata

Taxon classificationAnimaliaORDOFAMILIA

(F. O. Pickard-Cambridge, 1896) stat. n.

Figs 16 , 19 , 54–57 , 58–65 , 66–73 , 74–75 , 76 , 77 , 315 , 317

Avicularia avicularia variegata F. O. Pickard-Cambridge, 1896: 743, pl. 33, fig. 12 (lectotype female, here designated, Brazil, Amazonas, Itacoatiara [3°07'S, 58°26'W], Lower Amazon, January 1896, BMNH 1896.12.13.16; and paralectotype female, here designated, Brazil, Amazonas, Itacoatiara [3°07'S, 58°26'W], in banana tree, 7 February 1896, BMNH 1896.12.13.17; examined); Strand 1907b: 90; Petrunkevitch 1911: 49; Mello-Leitão 1923: 326, 376; Roewer 1942: 254; Bücherl 1957: 404; World Spider Catalog 2016.
Avicularia bicegoi Mello-Leitão, 1923: 329, figs 187, 189 (holotype subadult female, Brazil, Amazonas, Manaus [03°06'S, 60°01'W], Bicego col., MZUSP 133, examined); Roewer 1942: 254; Bonnet 1955: 830; Bücherl 1957: 404, figs 92–92a; World Spider Catalog 2016. **Syn. n.**

##### Remarks.

*Avicularia bicegoi* holotype has spermatheca midwidth expanded, about 1.5 times its basal and apical portion widths (Fig. 57); leg IV as long as leg I; whitish leg rings on distal femora, tibiae and metatarsi, and the type locality is Manaus, Amazonas. These characteristics match *Avicularia variegata* stat. n.; thus, we consider *Avicularia bicegoi* Mello-Leitão, 1923 junior synonym of *Avicularia variegata* (F. O. Pickard-Cambridge, 1896) stat. n.

##### Diagnosis.

**Figures 54–57.**
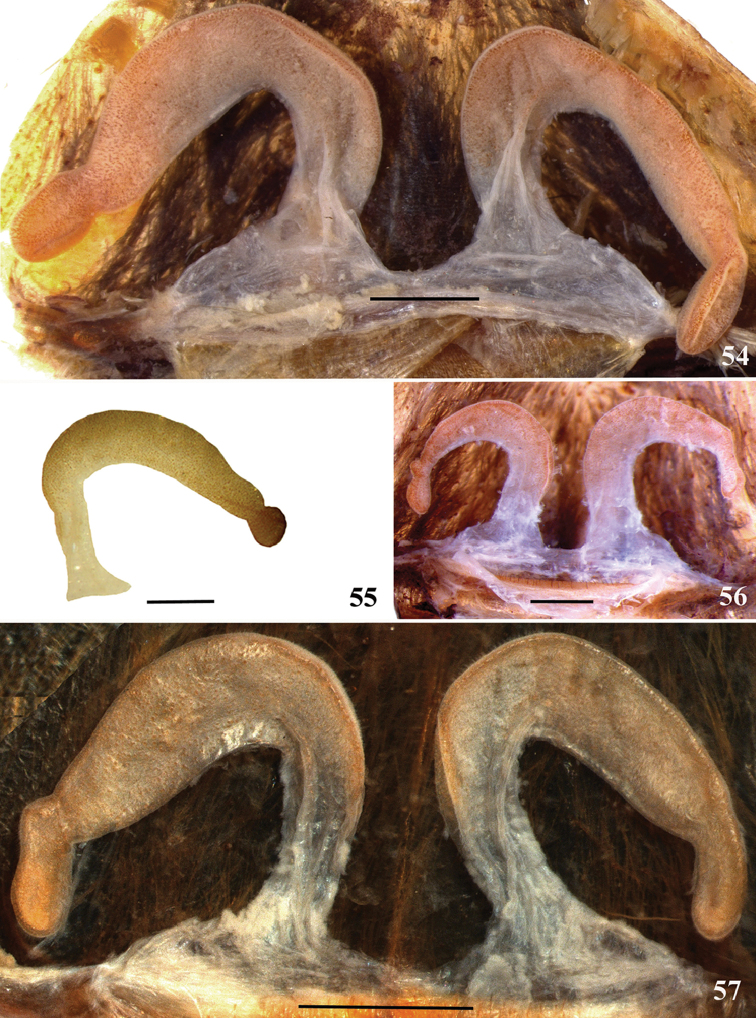
*Avicularia variegata* (F. O. Pickard-Cambridge, 1896) stat. n., spermathecae variation. **54** morphotype 1, Manaus, state of Amazonas, Brazil (INPA 4894) **55** holotype, Itacoatiara, state of Amazonas, Brazil (BMNH 1896.12.13.16) **56** morphotype 2, Alto Alegre, state of Roraima, Brazil (MZUSP 70946) **57** holotype of *Avicularia bicegoi* Mello-Leitão, 1923, Manaus, state of Amazonas, Brazil (MZUSP 133). Scale bars = 1 mm.

Females of *Avicularia variegata* stat. n. resemble *Avicularia juruensis* and *Avicularia taunayi* by the spermatheca midwidth expanded, about 1.5 times its basal and apical portion widths (Figs 54–57). They differ from females of *Avicularia taunayi* by lacking lobes on spermathecae. Females can be distinguished from those of *Avicularia juruensis* by the abdomen with vivid reddish brown guard-setae with pink sheen grouped on lateral and dorsal anterior areas, contrasting with the black short body setae and black venter (morphotype 1) (Fig. 70) or by legs and carapace with golden short body setae with green sheen, and pale yellow rings on distal femora, tibiae and metatarsi (morphotype 2) (Fig. 74). Males of *Avicularia variegata* stat. n. resemble those of *Avicularia avicularia*, *Avicularia rufa, Avicularia juruensis*, *Avicularia taunayi*, *Avicularia purpurea* and *Avicularia merianae* sp. n. by having tibial apophysis on leg I with well-developed base and grouped spiniform setae distally (Fig. 63). They differ from males of all these species except *Avicularia juruensis* by the well-developed prominence on tegulum (Fig. 60). Males and females of *Avicularia variegata* stat. n. can be distinguished from *Avicularia juruensis* by lacking intense purple sheen on carapace and legs (Figs 70, 71, 74, 75) or by occurrence area: Brazil, state of Amazonas (especially Manaus region) to state of Roraima, and Venezuela (Fig. 76).

##### Material examined.

1 female, Brazil, Amazonas, Manaus [03°06'S, 60°01'W], Parquejo, R. Oliveira-Filho col., 27 May 2008 (INPA 4894); 1 male, Brazil, Amazonas, Manaus [03°06'S, 60°01'W], Praça 14, M. P. Sena col., 27 March 1980 (INPA 4897).

**Figures 58–65.**
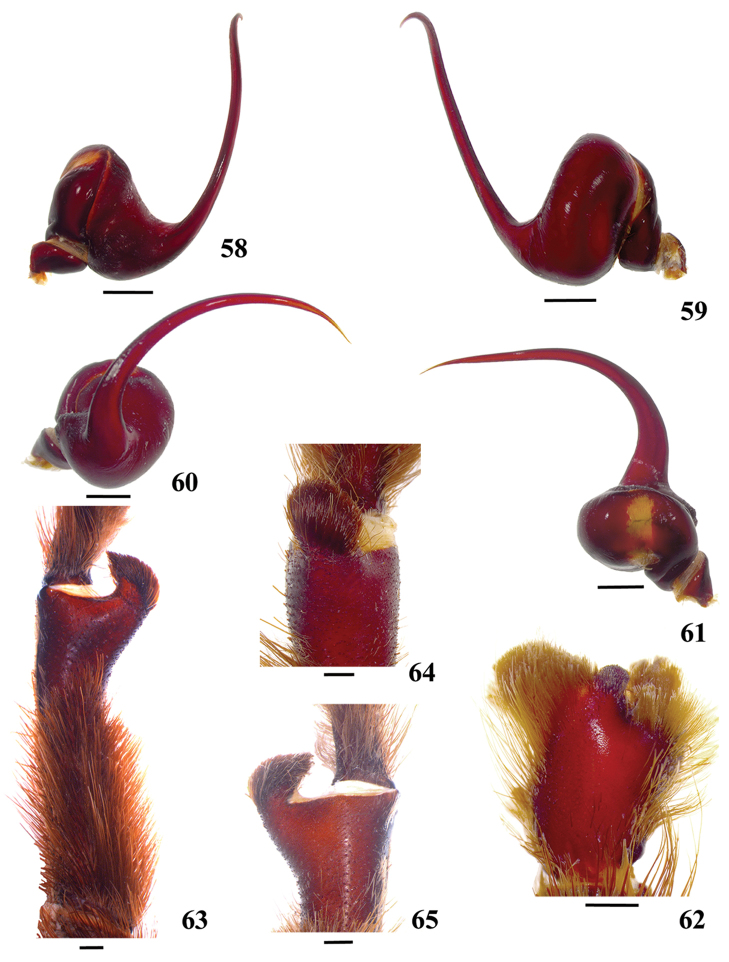
*Avicularia variegata* (F. O. Pickard-Cambridge, 1896) stat. n., male (INPA 4897). **58–61** left palpal bulb **58** prolateral **59** retrolateral **60** frontal **61** dorsal **62** left cymbium, dorsal **63–65** left tibial apophysis of leg I **63** prolateral **64** ventral **65** retrolateral. Scale bars = 1 mm.

##### Additional material.

VENEZUELA: 1 juvenile female, D. Grimaldi col., March 1989 (AMNH Ve21); *Distrito Capital*: Caracas [10°29'N, 66°54'W], Avila Mountains, 1 juvenile female and 1 immature, on trees, C. Siederman col., August 1991, (AMNH Ve18); *Monagas*: Caripito [10°06'N, 63°06'W], 1 male, 15–31 March 1942, Venezuela Expedition, Dept. Tropical Research, N. Y. Zool. Society, W. Beebe col., high and low jungle trails (AMNH Ve31); *Amazonas*: Puerto Ayacucho [5°39'N, 67°38'W], reg. 22, 2 males, in trees, C. Siederman col., May 1993 (AMNH Ve29); BRAZIL: *Roraima*: Alto Alegre [2°53'N, 61°29'W], 1 female, C. M. Moraes ded., April 2013, (MZUSP 70946); Amajari, Vila Tepequém (03°47'54"N, 61°44'57"W), 1 male, 17 November 2008, Yamaguti & Pinto da Rocha col. (MZUSP 70945); Boa Vista [2°49'N, 60°40'W], Balneário Água Boa, 1 male, 6h00, S. M. B. Lima col., 02 January 2002 (MNRJ 12968); Caracaraí, Estação Ecológica Niquiá [1°49'N, 61°07'W], Hotel Ecotur, 1 immature male, 07 October 2001 (IBSP 11270); Ilha de Maracá [3°25'N, 61°39'W], 2 males, A. B. Bonaldo col., 31 January to 14 February 1992 (MCP 1969); 2 males, A. Lise leg., 13 January to 14 February 1992 (MCP 1968); *Amapá*: Macapá, 4 km from Pacoval [0°02'N, 51°04'W], 1 female, Dr. Hoge col., 4 August 1965 (IBSP 3837); [0°03'S, 49°33'W], 1 female, 4 August 1965, Dr. Hoge col., died 26 June 1968 (IBSP 3873); *Amazonas*: Coari, Porto Urucu, Base de Operações Geólogo Pedro de Moura, (4°45'47"S, 65°02'41"W), 1 male, Dias et al. col., 2006 (MPEG 15633); (4°48'45"S, 65°02'01"W), 1 male, L. T. Miglio col., 09 July 2006 (MPEG 15641); Itacoatiara [3°07'S, 58°26'W], 1 female, Dirings col., March 1961 (IBSP 4265); Itapiranga [2°44'S, 58°01W], 1 immature male, EPA col., 11 September 1968 (MZUSP 10.860); Manaus [03°06'S, 60°01'W], 2 males, T. Gasnier col. (INPA 4952, INPA 4948); 1 male, H. Höfer leg., 28 March 1988 (INPA 4891); 1 female, 2 February 1948 (AMNH 1.13); 1 juvenile female, February 1943 (AMNH 1.26); Balneário do SESC, 1 female, R. Freitas col., 07 December 1975 (INPA 4896); conjunto Suframa, 1 juvenile female, Larissa col., 13 November 1997 (INPA 4893); conjunto Acariquara, 1 male, D. M. M. Mendes col., 18 March 2005 (INPA 4895); *campus* INPA, 1 male, Albuquerque col., May 1995 (INPA 4887); Estação Ecológica Experimental de Silvicultura Tropical, INPA, 1 female, A. L. R. Barreto col., 29 July 1980 (ZUEC 015); Estrada Manaus–Caracaraí, km 45, 2 immature males, J. Vasconcellos Neto col., July 1978 (ZUEC 019); INPA, Mata do Laguinho, 1 male and 1 female, 25 April 1959 (IBSP 3503); INPA, *campus* Peralta, 1 female, F. J. A. col., 13 January 1988 (INPA 4881); Manaus Airport, 1 male, A. Barros col., 27 April 1980 (INPA 4892); Peralta, 1 male, F. J. A. col., 05 June 1993 (INPA 4889); 1 female, F. J. A. col., 03 December 1990 (INPA 4883); Prédio do INPA, V8, 3°andar, Silvicultura, 1 male, P. A. Celeste col., 19 May 1986 (INPA 4888); Rio Tarumá, off Rio Negro, Manaus [03°06'S, 60°01'W], 1 juvenile female, N. Gordon col., June 1995, in house (AMNH RW32); Terra Nova [Manaus neighborhood or Amazonas’s town?], 1 male, Mancelu col., 07 September 1975 (INPA 4890); Vivenda Verde [Manaus neighborhood?], 1 male, H. Brandão col., 29 March 2002 (INPA 4885); Maraã [2°17'S, 65°00'W], Rio Japurá, Maguari, 1 male, R. Constantino col., 02 December 1998 (MPEG 5182); 1 juvenile female (MPEG 5504); Presidente Figueiredo [02°01'S, 60°01'W], UHE Balbina, 1 female, 2 juvenile females, faunal rescue team col., February 1988, ref. 56112 (IBSP 7872, IBSP 7871, IBSP 7875, respectively); 2 immatures, November 1987, collection Bokermann no. 886 (MZUSP 32173); 1 female, 1987, ref. 55569-12 (IBSP 7900); 2 juvenile females, 1 immature male, 1987, ref. 56681 (IBSP 7901); 1 juvenile female, November 1987, ref. IB34 (IBSP 7876); 1 male, 20 November 1987, ref. 55569-20 (IBSP 7873); 1 male, ref. 55854-2 (IBSP 7878); 3 females, 1 male, 2 immatures, 1987, ref. 56681 (IBSP 7902, IBSP 7903); 1 male, 1988, ref. 56572-7 (IBSP 7874); 3 juvenile females, 2 immature males, Eletronorte col., April 1988 (MNRJ 13818); Cachoeira do Tucumã, Rio Uatumã [2°36'S, 58°05'W], 1 immature (IBSP 7934); Margens do Rio Uatumã [2°36'S, 58°05'W], 1 female, 1 juvenile female, C. F. Alvarenga col., October 1987 (MNRJ 13652); *Pará*: Belém [1°26'S, 48°28'W] 1 female, Dr. Hoge & João col., September 1952 (IBSP 3119).

##### Female.

**Redescription.** INPA 4894. Carapace: 19.27 long, 19.06 wide, 4.20 high. Chelicera: 7.25 long. Legs (femur, patella, tibia, metatarsus, tarsus, total): I: 16.22, 10.06, 12.58, 10.94, 7.27, 57.07. II: 15.21, 9.21, 11.43, 10.37, 6.93, 53.15. III: 13.91, 8.39, 11.34, 10.72, 6.95, 51.31. IV: 16.89, 9.04, 14.17, 14.00, 6.94, 61.04. Palp: 11.19, 7.10, 7.84, –, 8.88, 35.01. Midwidths: femora I–IV= 3.40, 3.67, 3.63, 3.78, palp= 3.08; patellae I–IV= 3.68, 3.86, 4.03, 3.89, palp= 3.18; tibiae I–IV= 3.41, 3.21, 3.24, 3.38, palp= 2.98; metatarsi I–IV= 2.28, 2.52, 2.29, 2.58; tarsi I–IV= 3.20, 3.00, 2.97, 2.97, palp= 3.03. Abdomen: 24.77 long, 16.94 wide. Spinnerets: PMS, 2.34 long, 0.93 wide, 0.31 apart; PLS, 2.70 basal, 1.59 middle, 3.50 distal; midwidths 2.22, 1.83, 1.43, respectively.

Carapace: 1.01 times longer than wide; cephalic region not raised, thoracic striae inconspicuous.

Fovea: deep, slightly recurve, 2.35 wide.

Eyes: eye tubercle 0.95 high, 2.70 long, 3.71 wide. Clypeus 0.67. Anterior row of eyes procurve. Posterior row of eyes recurve. Eye size and interdistances: AME 0.86, ALE 0.89, PME 0.39, PLE 0.58, AME–AME 0.71, AME–ALE 0.64, AME–PME 0.22, ALE–ALE 2.35, ALE–PME 0.91, PME–PME 2.45, PME–PLE 0.08, PLE–PLE 3.10, ALE–PLE 0.55, AME–PLE 0.68.

Maxilla: length to width: 1.84. Cuspules: 100–200 spread over ventral inner heel. Labium: 2.68 long, 3.26 wide, with 103 cuspules spaced by one diameter in third distal area. Labium-sternal groove shallow, flattened, with two sigilla.

Chelicera: basal segment with 15 teeth and some small teeth on promargin. Sternum: 9.99 long, 8.39 wide. Sigilla: only posterior pair evident, rounded, less than one diameter from margin.

Legs: Formula: IV=I II III. Length leg IV to leg I: 1.07. Clavate trichobothria: distal 2/3 tarsi I–IV. Scopulae: Tarsi I–IV fully scopulate. Metatarsi I–II fully scopulate; III 2/3; IV 1/3 distal scopulate. IV divided by a wide row of setae.

Type II urticating setae (Fig. 16): 0.64–0.72 long, 0.016–0.019 wide.

Spermathecae (Fig. 54): two completely separated, not-twisted long spermathecae, with walls lacking projections or lobes and accentuated outwards curvature medially. Midwidth expanded, about 1.5 times its basal and apical portion widths, and weakly-sclerotized area shorter than half the length of well-sclerotized area.

Color pattern (Fig. 70): carapace brown with greyish short body setae with green sheen. Carapace border with long setae the same color as dorsal carapace short body setae. Coxae, labium, sternum and maxillae dark brown, darker than ventral femora. Legs and palps with greyish short body setae with green and pink sheen and brown guard-setae with darker base and contrasting whitish apex. Leg rings on distal femora, tibiae and metatarsi whitish. Abdomen dorsum with vivid reddish brown guard-setae with vivid pink sheen grouped on lateral and dorsal anterior areas and black short body setae. Abdomen venter velvety black.

**Figures 66–73.**
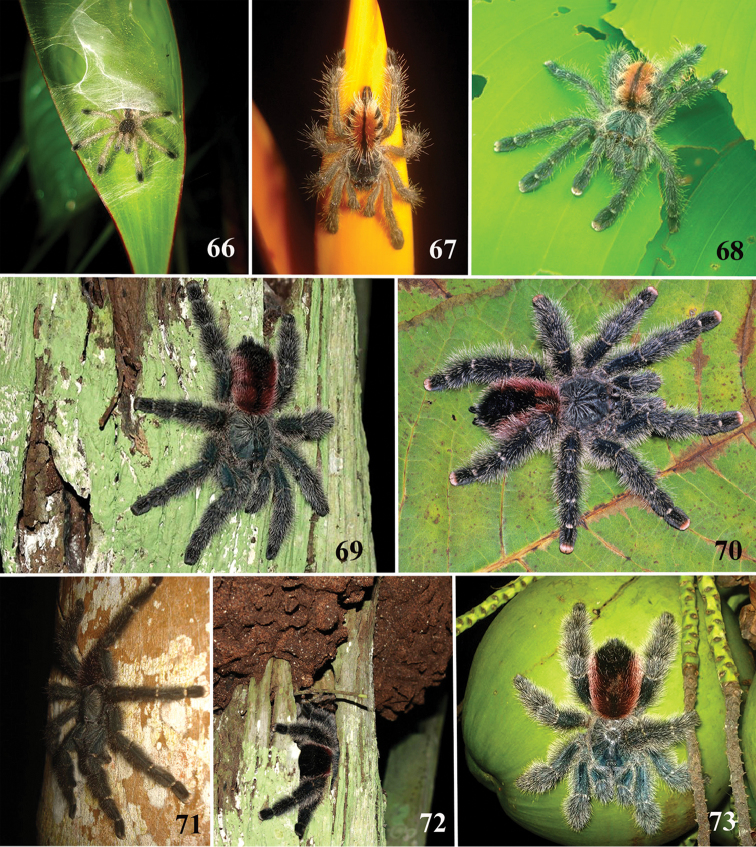
*Avicularia variegata* stat. n., morphotype 1, habitus **66** immature on leaf retreat **67** immature **68** juvenile **69** juvenile female **70** female **71** male **72** adult inside retreat on tree bark **73** female in coconut tree. Photos: **66–69, 71–73** Marlus Almeida; **70** R. C. West.

##### Male.

**Description.** INPA 4897. Carapace: 19.38 long, 19.08 wide, 5.27 high. Chelicera: 6.69 long. Legs (femur, patella, tibia, metatarsus, tarsus and total): I: 19.21, 10.36, 14.03, 14.46, 7.62, 65.68. II: 18.52, 9.55, 14.57, 14.15, 7.94, 64.73. III: 16.41, 8.40, 13.17, 13.82, 7.60, 59.40. IV: 19.64, 8.88, 16.32, 18.13, 7.72, 70.69. Palp: 11.53, 6.64, 9.10, –, 3.78, 31.05. Midwidths: femora I–IV= 3.92, 3.90, 4.09, 3.60, palp= 2.85; patellae I–IV= 3.87, 4.03, 3.86, 3.83, palp= 3.03; tibiae I–IV= 3.06, 3.31, 2.85, 3.18, palp= 2.71; metatarsi I–IV= 2.28, 2.12, 2.00, 1.95; tarsi I–IV= 2.49, 2.45, 2.25 2.31, palp= 2.61. Abdomen: 22.45 long, 14.42 wide. Spinnerets: PMS, 2.31 long, 0.79 wide, 0.16 apart; PLS, 2.35 basal, 1.20 middle, 3.56 distal; midwidths 1.95, 1.63, 1.25, respectively.

As in female, except:

Carapace: 1.02 times longer than wide.

Fovea: 2.59 wide.

Eyes: eye tubercle 1.59 high, 2.67 long, 3.48 wide. Clypeus 0.44. Eye size and interdistances: AME 0.84, ALE 0.79, PME 0.29, PLE 0.68, AME–AME 0.45, AME–ALE 0.52, AME–PME 0.27, ALE–ALE 2.53, ALE–PME 0.70, PME–PME 2.23, PME–PLE 0.20, PLE–PLE 2.68, ALE–PLE 0.52, AME–PLE 0.72.

Maxilla: length to width: 2.05. Labium: 2.19 long, 3.19 wide, with 100 cuspules spaced by one diameter in third area. Labio-sternal groove with no evident sigilla.

Chelicera: basal segment with 9 teeth and some small teeth on promargin. Sternum: 9.93 long, 7.45 wide. Sigilla: only posterior evident, rounded, less than one diameter from margin.

Legs: Length leg IV to leg I: 1.08. Scopula: Metatarsi III 1/2 distal scopulate.

Type II urticating setae: 0.91–1.00 long, 0.019–0.020 wide.

Palp (Figs 58–61): globous bulb with small subtegulum and well-developed prominence on tegulum. Embolus: not flattened, lacking keels, 5.65 long in retrolateral view, about 3.5 times tegulum’s length. Medial portion and tegulum’s margin form an acute angle in retrolateral view. Proximal part very curved in frontal view; thin distal width, abruptly narrowing distally; basal, middle, and distal width 0.91, 0.31, 0.05, respectively. Tegulum: 3.04 long, 1.65 high in retrolateral view. Cymbium subtriangular with subequal lobes, having a well-developed rounded process on retrolateral lobe, bearing thick setae (Fig. 62).

Tibial apophysis (Figs 63–65): single branch on prolateral leg I, with well-developed base and grouped spiniform setae distally. Male metatarsus I touches retrolaterally tibial apophysis’ setae when folded.

##### Color pattern ontogeny.

Brownish juveniles lacking metallic sheen, black tarsi contrasting with other lighter articles and abdomen dorsum reddish, with dorsal central longitudinal black stripe disconnected from transversal black stripes (Fig. 66). When mature, both males and females lose this pattern.

**Figures 74–75.**
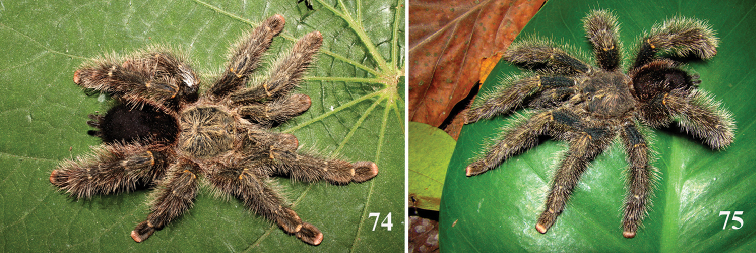
*Avicularia variegata* stat. n., morphotype 2, habitus **74** female **75** male. Photos: **74** C. S. Fukushima; **75** R. Bertani.

##### Distribution.

**Figure 76.**
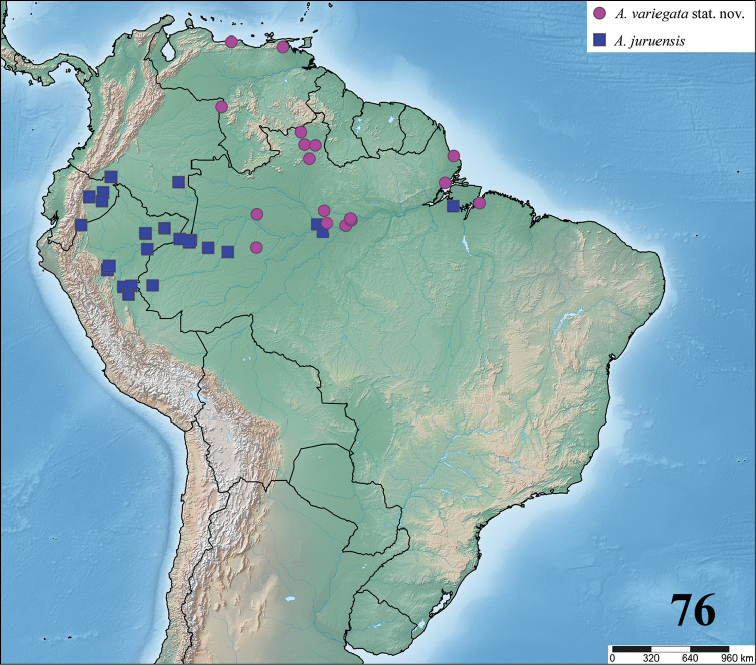
Map showing records of *Avicularia variegata* (F. O. Pickard-Cambridge, 1896) stat. n. and *Avicularia juruensis* Mello-Leitão, 1923.

Venezuela and Brazil (states of Roraima, Amapá, Amazonas and Pará) (Fig. 76).

##### Natural history.

Silva and Meirelles (2016) reported a predation on *Troglodytes musculus* (Naumann, 1823) bird by an specimen of *Avicularia variegata* stat. n. in a urban park at Manaus, state of Amazonas, Brazil.

##### Variation.

**Figures 77.**
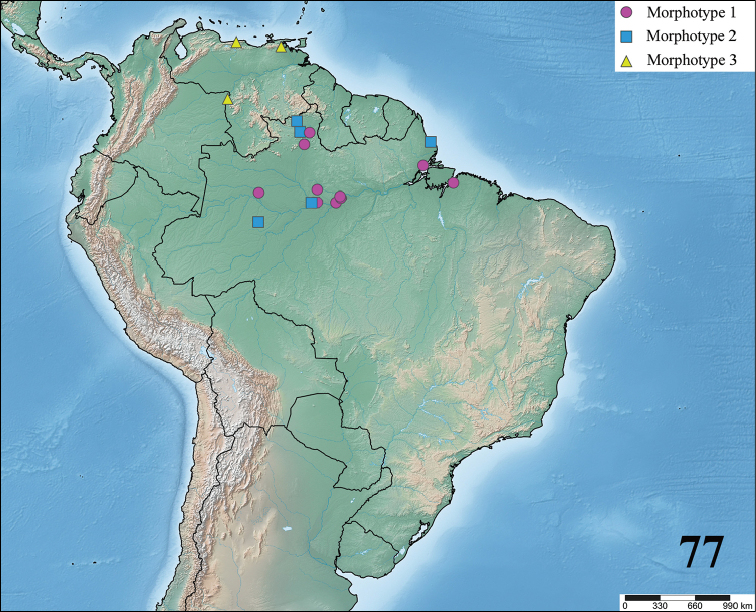
Map showing records of *Avicularia variegata* F. O. Pickard-Cambridge, 1896 stat. n. morphotypes.

All examined specimens of *Avicularia variegata* stat. n. have grizzled setae on palps and legs (except those found in Venezuela), but with slight differences in body coloration. We detected three morphotypes. Morphotype 1 is found especially near Manaus (Fig. 77) and females have carapace, legs, and palps with greyish short body setae with very intense green sheen and whitish leg rings (Fig. 70). Abdomen has vivid reddish guard-setae grouped on lateral and dorsal anterior areas, black short body setae and venter velvety black. Males have black abdomen with some whitish tipped setae homogeneously distributed (Fig. 71). Morphotype 2 is found in same area (Fig. 77), but has overall greyish coloration, with short body setae with discrete green sheen and yellowish leg rings. Females have abdomen with light brown guard-setae grouped on lateral and dorsal anterior areas, black short body setae, and venter dark brown (Fig. 74). Males are very similar to morphotype 1 but with yellowish rings (Fig. 75). Males from Puerto Ayacucho (AMNH Ve29) and from Caripito (AMNH Ve31), Venezuela, have genitalia and legs with proportions compatible with those from Brazil, as well as whitish leg rings. However, they do not have white tipped hairs on legs and abdomen as specimens of *Avicularia variegata* stat. n. from Manaus, Brazil (Fig. 77), and the prominence on palpal bulb is not well-developed as in specimens from Manaus (morphotype 1). The juvenile female AMNH Ve21, also from Venezuela, has short body setae with golden sheen and very discrete white tipped setae in legs and palps as well as whitish rings. We prefer not to consider these forms as separate species because these slight differences can be due to populational variation since the specimens were collected in the northern limit of the species distribution. It is here called morphotype 3. Despite being collected in Caracas, also in the northern limit of the species, the specimen AMNH Ve18 seems to be a typical morphotype 1. It is necessary to examine more material to understand morphological variation and species boundaries of Venezuelan *Avicularia*.

In his expedition on Lower Amazonas, F. O. Pickard-Cambridge (1896) made interesting observations about color variations along the river. According to him, there was a tendency in *Avicularia* spp. to have grizzled setae as he went further west (F. O. Pickard-Cambridge 1896). When F. O. Pickard-Cambridge (1896) described *Avicularia variegata* stat. n., he considered it as a subspecies of *Avicularia avicularia* and pointed out that some could consider *Avicularia variegata* stat. n. a species in the differentiation process—as further westwards it extended, the more evident the differential characters might be. He also observed the presence of intermediate forms at Santarém, state of Pará, which had the setae of the first two pairs of legs decidedly grizzled (F. O. Pickard-Cambridge 1896). We failed to find specimens with this pattern in our studied sample. Some specimens we examined had posterior legs with more grizzled setae than anterior, but none without any grizzled setae. Despite these two morphotypes being very distinct, F. O. Pickard-Cambridge (1896) stated a careful comparasion was needed of both forms to prove that there are more differences than just coloration. In fact, we found other differences. Leg IV of *Avicularia avicularia* is more than 10% longer than leg I and spermathecae midwidth is as wide as its base width; whereas leg IV of *Avicularia variegata* stat. n. has roughly the same length of leg I and spermathecae midwidth is expanded. Accordingly, we can consider them as separate taxa. Thus, we elevate the former subspecies *Avicularia avicularia variegata* F. O. Pickard-Cambridge, 1896 to species status, resulting in *Avicularia variegata* (F. O. Pickard-Cambridge, 1896) stat. n.

#### Avicularia minatrix

Taxon classificationAnimaliaORDOFAMILIA

Pocock, 1903

Figs 19 , 78–79 , 80–87 , 88–89 , 90 , 312

Avicularia minatrix Pocock, 1903: 81 (holotype female, Venezuela, Lara, Duaca [10°17'N, 69°09'W], BMNH 1903.7.1.120, examined); Petrunkevitch 1911: 50; Mello-Leitão 1923: 377; Roewer 1942: 255; Bonnet 1955: 832; Smith 1992: 22, figs 1–11; Tinter 1993: 10, figs 1–3, World Spider Catalog 2016.

##### Diagnosis.

**Figures 78–79.**
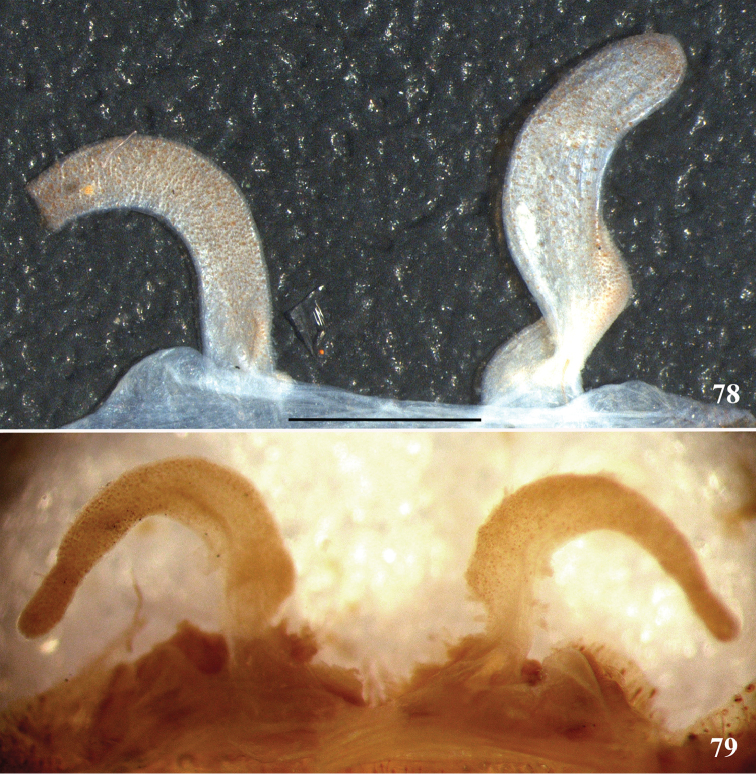
*Avicularia minatrix* Pocock, 1903, spermathecae variation. **78** Venezuela (IBSP 12886) **79** holotype, Duaca, state of Lara, Venezuela (BMNH 1903.7.1.120). Scale bars = 1 mm.

Females and males of *Avicularia minatrix* can be distinguished from all other *Avicularia* species by the abdominal pattern, vivid orange lateral spots over black background (Figs 88–89). Additionally, females of *Avicularia minatrix* resemble females of *Avicularia avicularia*, *Avicularia rufa*, *Avicularia purpurea*, *Avicularia hirschii* and *Avicularia merianae* sp. n. by the spermathecae midwidth as wide as its base (Fig. 78). They can be distinguished from *Avicularia avicularia* and *Avicularia rufa* by leg IV as long as leg I; from *Avicularia purpurea* and *Avicularia merianae* sp. n. by the spermathecae weakly-sclerotized area shorter than half the length of well-sclerotized area, and from *Avicularia hirschii* by the non-twisted spermathecae (Fig. 78). Males of *Avicularia minatrix* resemble those of *Avicularia hirschii*, *Avicularia lynnae* sp. n. and *Avicularia caei* sp. n. by the tibia I with a discrete elevation covered by a cluster of setae in apical portion, on prolateral side (Fig. 86), and can be distinguished from all of them by lacking process on retrolateral lobe of the cymbium.

##### Material examined.

VENEZUELA: 1 female, M. Baumgarten leg. 1994, ref. 73485 (IBSP 12886); 1 male, pet trade (MZUSP 70949); 1 male, L. Koschorreck col., 1991 (IBSP 11596).

##### Female.

**Redescription.** IBSP 12886. Carapace 12.85 long, 12.30 wide, 4.50 high. Chelicera: 4.09 long. Legs (femur, patella, tibia, metatarsus, tarsus, total): I: 9.38, 6.10, 6.91, 6.39, 4.24, 33.02. II: 8.84, 5.85, 6.56, 6.27, 4.26, 31.78. III: 7.82, 5.15, 5.23, 6.05, 3.49, 27.74. IV: 9.99, 5.07, 7.79, 7.42, 3.93, 34.20. Palp: 6.87, 4.31, 4.08, –, 5.00, 20.26. Midwidths: femora I–IV= 1.99, 2.00, 1.62, 2.13, palp= 2.11; patellae I–IV= 2.39, 2.24, 2.48, 2.59, palp= 2.26; tibiae I–IV= 2.01, 2.25, 2.01, 2.32, palp= 2.05; metatarsi I–IV= 1.95, 1.58, 1.59, 1.92; tarsi I–IV= 2.05, 2.02, 1.76, 2.02, palp= 2.12. Abdomen 14.10 long, 8.91 wide. Spinnerets: PMS, 1.72 long, 0.95 wide, 0.15 apart; PLS 2.57 basal, 1.68 middle, 2.34 distal; midwidths 1.57, 1.18, 0.93, respectively.

Carapace: 1.05 times longer than wide; cephalic region not raised, thoracic striae inconspicuous. Carapace covered by long setae.

Fovea: deep, slightly recurve, 2.46 wide.

Eyes: eye tubercle: 0.90 high, 1.97 long, 2.77 wide. Clypeus 0.46. Anterior row of eyes procurve, posterior row of eyes slightly recurve. Eye size and interdistances: AME 0.64, ALE 0.59, PME 0.24, PLE 0.58, AME–AME 0.27, AME–ALE 0.36, AME–PME 0.14, ALE–ALE 1.70, ALE–PME 0.55, PME–PME 1.58, PME–PLE 0.15, PLE–PLE 2.02, ALE–PLE 0.40, AME–PLE 0.47.

Maxilla: length to width: 1.48. Cuspules: 190 spread over inner heel. Labium: 1.87 long, 2.31 wide, with about 50 cuspules spaced by one diamenter of each other on anterior half (malformed). Labium sternal groove shallow, flat, with two separate, large sigilla.

Chelicera: basal segment with 8 teeth and some small teeth on promargin.

Sternum: 7.27 long, 4.32 wide. Sigilla: only posterior evident, rounded, very close to margin.

Legs: Formula: IV=I II III. Length leg IV to leg I: 1.04. Clavate trichobothria: distal 2/3 of tarsi I–II; distal 1/2 of tarsi II–IV. Scopula: Tarsi I–IV fully scopulate. Metatarsi I–II fully scopulate, III 2/3, IV 1/3 distal scopulate. IV divided by row of setae.

Type II urticating setae: 0.38–0.51 long and 0.011–0.013 wide (measured in holotype BMNH 1903.7.1.120).

Spermathecae (Fig. 78): two completely separated, not-twisted long spermathecae, with walls lacking projections or lobes and accentuated outwards curvature medially. Midwidth as wide as its base width and weakly-sclerotized area shorter than half the length of well-sclerotized area.

Color pattern (Fig. 88): carapace orange brown with golden short body setae with pink sheen. Carapace border with long setae the same color as dorsal carapace short body setae. Coxae, labium, sternum and maxillae orange brown, same color of ventral femora. Legs and palps with brown short body setae with pink sheen and long orange guard-setae. Leg rings on distal femora, tibiae and metatarsi whitish. Abdomen dorsum with orange guard-setae homogeneously distributed, vivid orange short body setae forming lateral spots and black short body setae forming dark background. Ventral abdomen brown.

**Figures 80–87.**
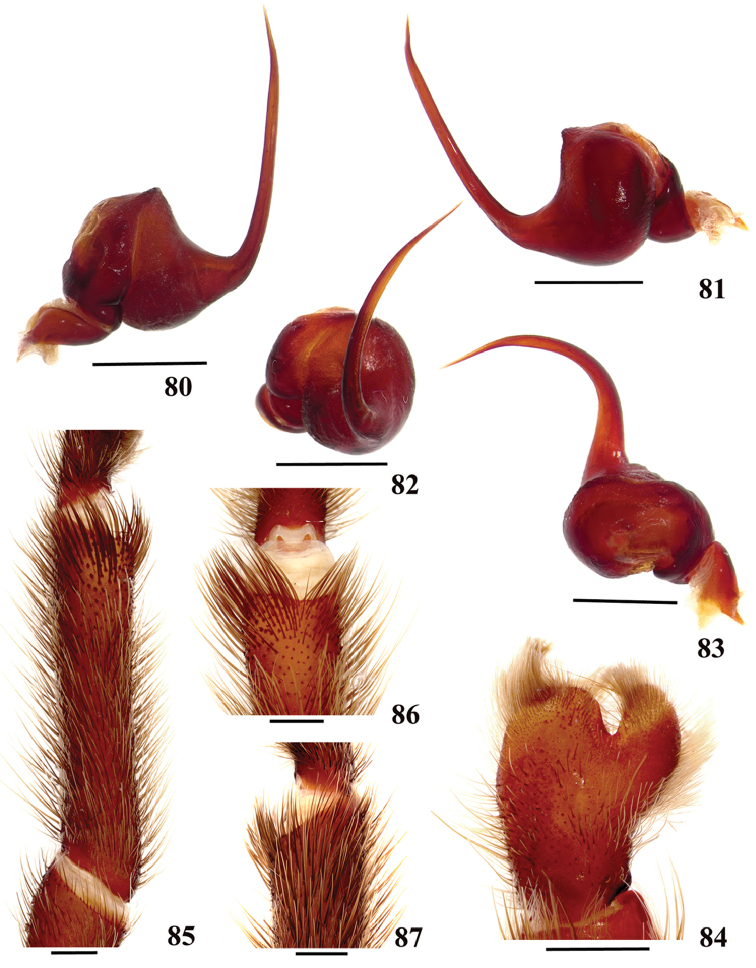
*Avicularia minatrix* Pocock, 1903, male (MZUSP 70949). **80–83** right palpal bulb (mirrored) **80** prolateral **81** retrolateral **82** frontal **83** dorsal **84** right cymbium, dorsal (mirrored) **85–87** left tibia I **85** prolateral **86** ventral **87** retrolateral. Scale bars = 1 mm.

**Figures 88–89.**
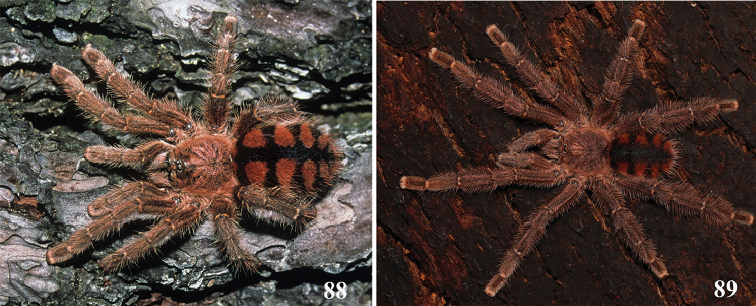
*Avicularia minatrix* Pocock, 1903, habitus. **88** female, state of Falcón, Venezuela **89** male. Photos: **88** J. Huff; **89** J. Newland.

##### Male.

**Redescription.** IBSP 11596. Carapace 10.08 long, 9.49 wide, 2.32 high. Chelicera 2.84 long. Legs (femur, patella, tibia, metatarsus, tarsus, total): I: 9.40, 5.29, 7.46, 6.76, 3.97, 32.88. II: 8.97, 4.76, 5.73, 5.92, 3.55, 28.93. III: 7.30, 3.89, 5.08, 5.92, 3.58, 25.77. IV: 9.76, 4.66, 7.98, 8.61, 4.00, 35.01. Palp: 5.83, 3.43, 4.17, –, 2.47, 15.9. Midwidths: femora I–IV= 2.02, 1.77, 2.04, 1.60, palp= 1.61; patellae I–IV= 1.85, 1.76, 1.82, 1.87, palp= 1.48; tibiae I–IV= 1.73, 1.65, 1.72, 1.70, palp= 1.48; metatarsi I–IV= 1.25, 1.19, 1.19, 1.18; tarsi I–IV= 1.54, 1.33, 1.42, 1.21, palp= 1.75. Abdomen 9.78 long, 5.73 wide. Spinnerets: PMS, 1.06 long, 0.59 wide, 0.18 apart; PLS, 1.66 basal, 0.69 middle, 1.58 distal; midwidths 0.73, 0.59, 0.49, respectively.

As in female, except:

Carapace: 1.06 times longer than wide.

Fovea: 1.56 wide.

Eye: eye tubercle: 1.51 long, 2.24 wide, 0.71 high. Clypeus: 0.20 wide. Eye size and interdistances: AME 0.58, ALE 0.60, PME 0.26, PLE 0.54, AME–AME 0.25, AME–ALE 0.18, AME–PME 0.16, ALE–ALE 1.25, ALE–PME 0.45, PME–PME 1.33, PME–PLE 0.13, PLE–PLE 1.69, ALE–PLE 0.10, AME–PLE 0.42.

Maxilla: length to width: 1.98. Cuspules: 100–115 spread over inner heel. Labium: 1.04 long, 1.58 wide, with about 80 cuspules spaced by two diameters from each other on anterior half.

Chelicera: basal segment with 10 teeth decreasing in size from distal area. Sternum: 4.95 long, 3.72 wide. Sigilla: three pairs, posterior oval.

Legs: Length leg IV to leg I: 1.07. Scopula: tarsi IV with some sparse setae.

Type II urticating setae: not seen due to abdomen in poor conditions.

Palp (Figs 80–83, 312): globous bulb with a small subtegulum, lacking prominence on tegulum. Embolus: not flattened, lacking keels, 2.66 long in retrolateral view, about 3.5 times tegulum’s length. Medial portion and tegulum’s margin form an acute angle in retrolateral view. Proximal part very curved in frontal view; thin distal width, abruptly narrowing distally; basal, middle, and distal width of 0.48, 0.15, 0.04, respectively. Tegulum: 1.25 long, 0.88 high in retrolateral view. Cymbium subtriangular with subequal lobes, lacking process on retrolateral lobe (Fig. 84).

Tibia I with discrete elevation covered by a cluster of setae in apical portion, on prolateral side (Figs 85–87).

Color pattern (Fig. 89): same as female, but the dorsal central black area is wider than in female.

**Figure 90.**
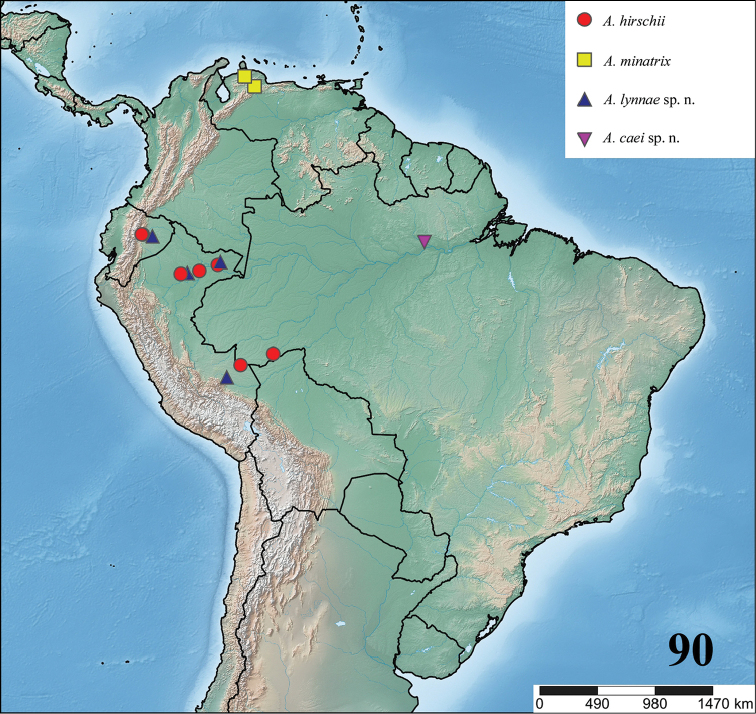
Map showing records of *Avicularia minatrix* Pocock, 1903, *Avicularia hirschii* Bullmer et al. 2006, *Avicularia lynnae* sp. n. and *Avicularia caei* sp. n.

##### Color pattern ontogeny.

Adults maintain the same coloration pattern of immatures. There is no drastic ontogenetic changes in this species.

##### Distribution.

Known only from Venezuela (Fig. 90).

##### Natural history.

Silken tubes are built by specimens inside tree bark and hollow branches and in the center of large bromeliads, which are in xenophyte bush grassland clearings of tropical forest (Smith 1992).

**Figures 91–92.**
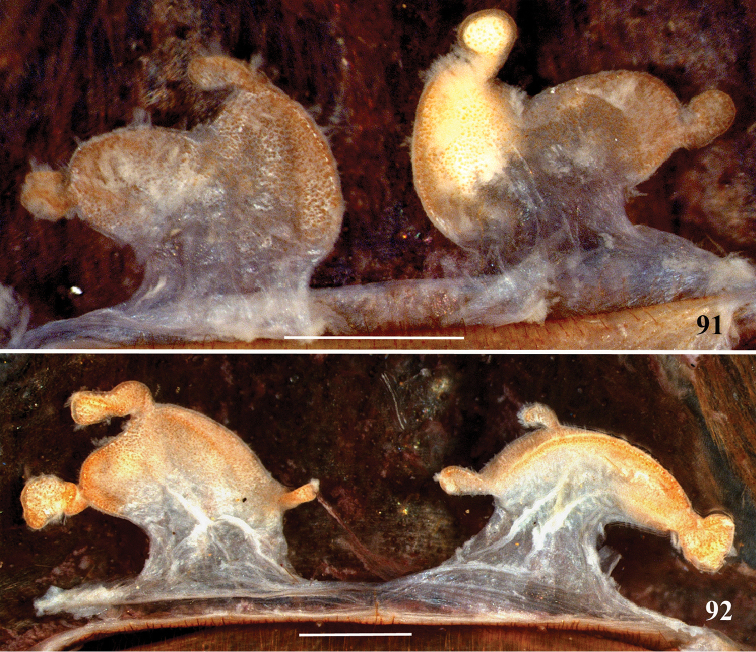
*Avicularia taunayi* (Mello-Leitão, 1920), spermathecae variation. **91** Sobradinho, Brasília, Distrito Federal, Brazil (DZUB 352) **92** Brasíla, Distrito Federal, Brazil (DZUB 1979). Scale bars = 1 mm.

#### Avicularia taunayi

Taxon classificationAnimaliaORDOFAMILIA

(Mello-Leitão, 1920)

Figs 19 , 91–92 , 93–100 , 101–104 , 105

Ancylochiros taunayi Mello-Leitão, 1920: 142 (holotype immature male, Brazil, Minas Gerais, Mariana [20°22'S, 43°25'W], J. P. Fonseca leg., MZUSP 327, examined); 1923: 319, 376, figs 41–44, 160; Roewer 1942: 225; Bonnet 1955: 316.
Anchylochiros taunayi : Petrunkevitch 1939: 291.
Avicularia taunayi : Raven 1985: 149; Bertani 2012: 78, fig. 176; Bertani and Motta 2013: 108, figs 1–13; World Spider Catalog 2016.

##### Diagnosis.

Females of *Avicularia taunayi* resemble those of *Avicularia juruensis* and *Avicularia variegata* stat. n. by the spermathecae with midwidth expanded; about 1.5 times its basal and apical portions widths (Fig. 91). They differ from all these species by the spermathecae with lobes from median to distal portions (Fig. 91). Males of *Avicularia taunayi* resemble *Avicularia avicularia*, *Avicularia variegata* stat. n., *Avicularia juruensis*, *Avicularia rufa*, *Avicularia purpurea*, and *Avicularia merianae* sp. n. by tibial apophysis on leg I with well-developed base and grouped spiniform setae distally (Fig. 98). They differ from *Avicularia purpurea* and *Avicularia merianae* sp. n. by well-developed process on cymbium retrolateral lobe (Fig. 97); from *Avicularia avicularia* and *Avicularia rufa* by leg IV as long as leg I and from *Avicularia variegata* stat. n. and *Avicularia juruensis* by developed prominence on tegulum (Fig. 95). Additionally, they can be distinguished from all these species by light brown spots extending from the dorsum to lateral region of abdomen (Fig. 103).

**Figures 93–100.**
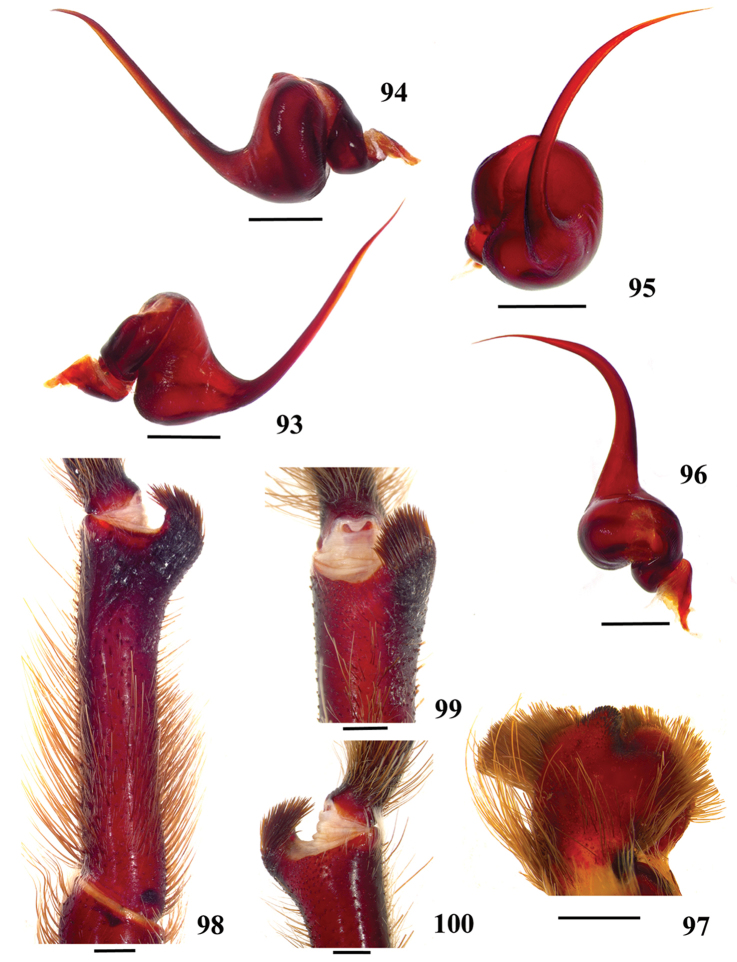
*Avicularia taunayi* (Mello-Leitão, 1920), male (DZUB 1675; except cymbium, DZUB 4542). **93–96** left palpal bulb **93** prolateral **94** retrolateral **95** frontal **96** dorsal **97** left cymbium, dorsal **98–100** right tibial apophysis of leg I (mirrored) **98** prolateral **99** ventral **100** retrolateral. Scale bars = 1 mm.

##### Material examined.

Female, Brazil, Minas Gerais, Barão de Cocais [19°56'S, 43°28'W], J. P. Couto col., 5 February 1971, ref. 4336 (IBSP 199); male, Brazil, Brasília D.F. [15°46'S, 47°55'W], Gláucia & Reuber col., 14 May 2002 (DZUB 1675).

**Figures 101–104.**
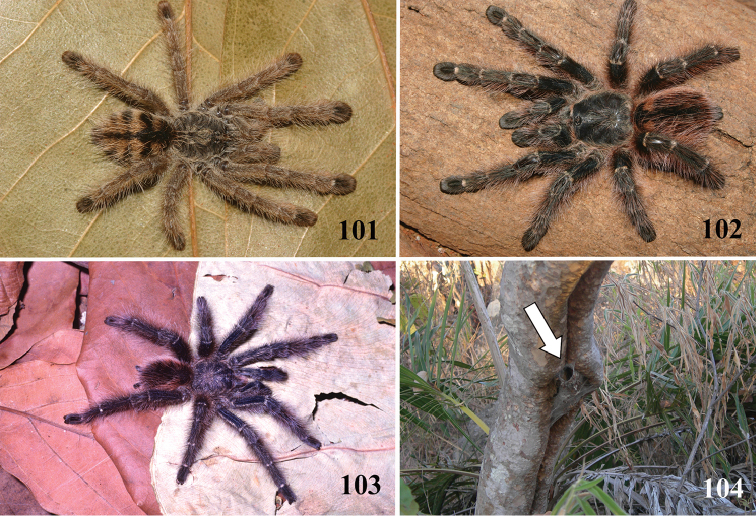
*Avicularia taunayi* (Mello-Leitão, 1920), habitus and retreat. **101** immature **102** female **103** male **104** retreat inside trunk of *Myrcia tormentosa* (Aubl.). Arrow indicating retreat opening. Photos: **101–103** R. Bertani; **104** C. S. Fukushima.

##### Additional material.

BRAZIL: *Pará*: Tucuruí [3°46'S, 49°40'W], 1 male, Equipe de Resgate de Fauna col., IBA 285 (IBSP 8570); 1 male, 3 immatures, 27 January 1986 (IBSP ref. 48014); *Mato Grosso*: Barra do Bugres [15°03'S, 57°10'W], Serra das Araras, 1 juvenile female, C. Strusman col., September 1992 (MCP 2293); Chapada dos Guimarães [15°27'S, 55°45'W], 1 female, M. Acuarensa col., November 1963 (AMNH 1.7); Barra do Tapirapé [10°38'S, 50°36'W] (Barro do Tapirapé [*sic*]), 2 females, B. Malkin col., 1962 (AMNH 1.5); *Goiás*: Ipameri [17°43'S, 48°09'W], 1 male, 1 female, F. R. Alves col., February 1996, ref. 78206 (IBSP 14397); *Distrito Federal*: Brasília [15°47'S, 47°53'W], 1 female, 15 November 2002, F. Brasil leg. (DZUB 1979); Paranoá [15°43'S, 47°44'W], 1 female, 1 immature, R. Bertani, C. S. Fukushima, R. H. Nagahama, P. C. Motta, P. Jotta, 12 July 2007 (DZUB 4707); Sobradinho [Colorado, Córrego do Urubu, 15°42'S, 47°51'W], 1 female, 1 January 1999, J. Marinho-Filho leg. (DZUB 352); *Minas Gerais*: Santa Vitória [18°51'S, 50°07'W], 1 female, M. Rosa col., 28 July 1981 (ZUEC 018); Monte Alegre de Minas [18°52'S, 48°52'W] (Monte Alegre [*sic*]), 1 male, A. Lourenço col., June 2005, ref. 95480 (IBSP 12780).

**Figure 105.**
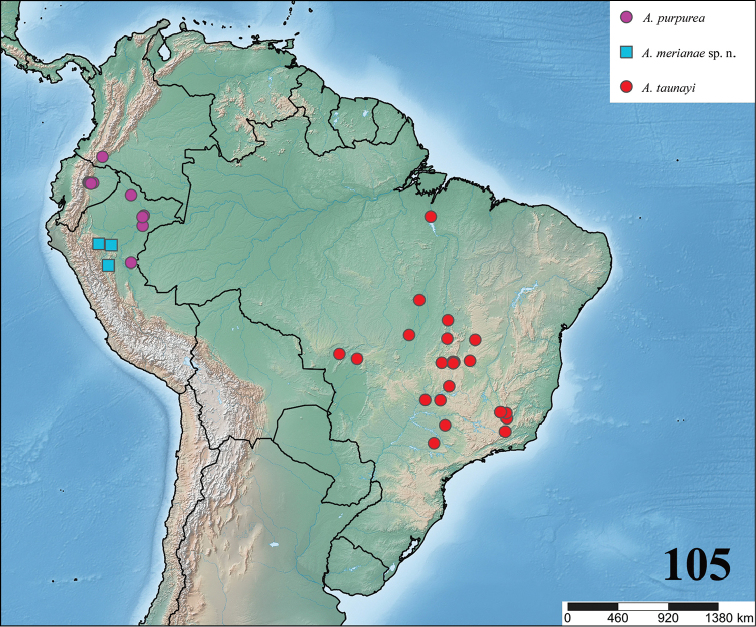
Map showing records of *Avicularia taunayi* (Melo-Leitão, 1920), *Avicularia purpurea* Kirk, 1990 and *Avicularia merianae* sp. n.

##### Description, color pattern ontogeny and distribution.

See Bertani and Motta (2013).

##### Complementary description.

Spermathecae (Figs 91–92): two completely separated not-twisted long spermathecae, with walls having lobes from median to distal portions and accentuaded outwards curvature medially. Midwidth expanded, about 1.5 times its basal and apical portion widths and weakly-sclerotized area shorter than half the length of well-sclerotized area.

Palp (Fig. 93–96): globous bulb with small subtegulum and developed prominence on tegulum. Embolus: not flattened, lacking keels, 2.62 long in retrolateral view, about 3.0 times tegulum’s length. Medial portion and tegulum’s margin form an acute angle in retrolateral view. Proximal part very curved in frontal view; thin distal width, abruptly narrowing distally; basal, middle, and distal widths 0.42, 0.24, 0.03, respectively. Tegulum: 1.76 long, 0.96 high in retrolateral view. Cymbium subtriangular with subequal lobes, having well-developed rounded process on retrolateral lobe, bearing thick setae (Fig. 97).

Tibial apophysis (Figs 98–100): single branch on prolateral leg I, with well-developed base and grouped spiniform setae distally. Male metatarsus I touches retrolaterally tibial apophysis’ setae when folded.

Type II urticating setae: 0.362–0.407 long, 0.009–0.013 wide in females; 0.840–0.968 long, 0.014–0.020 wide in males.

Color pattern (Figs 102–103): both male and female have long guard-setae on legs and palps not grizzled.

##### Color pattern ontogeny.

Brownish juveniles lacking metallic sheen, black tarsi contrasting with other lighter articles, and abdomen dorsum reddish, with dorsal central longitudinal black stripe connected with first two pairs of transversal black stripes (Fig. 101). When mature, part of the pattern remains (Figs 102–103). Adult females have abdomen with reddish brown guard-setae homogeneously distributed and black short body setae with spots of reddish brown short body setae (Fig. 102). Males have same abdominal pattern as females but reddish brown spots of short body setae are ill-defined (Fig. 103).

##### Distribution.

Brazil, states of Tocantins, Goiás, Pará, São Paulo, Mato Grosso, west of Bahia, Minas Gerais and in Brasília (Distrito Federal), in savannah areas (Fig. 105).

##### Natural history.

A small population of *Avicularia taunayi* was found at Distrito Federal, in a mountain area that had savannah areas mixed with anthropized areas with houses and farms (Bertani and Motta 2013). Specimens were found in tree holes within retreats similar to the ones made by other species of *Avicularia* (Fig. 104). This is a unique species of this genus in the bioma Cerrado (savannah) (Bertani and Motta 2013).

#### Avicularia juruensis

Taxon classificationAnimaliaORDOFAMILIA

Mello-Leitão, 1923

Figs 15 , 19 , 76 , 106–108 , 109–116 , 117–122 , 123–126 , 127 , 307

Avicularia juruensis Mello-Leitão, 1923: 321, 377, figs 156, 188 (syntypes 4 females and 1 male, Brazil, Amazonas, Juruá [4°47'S, 68°38'W], Garbe col., 1902, MZUSP 125A–D, examined; lectotype male (MZUSP 125C) and paralectotype female (MZUSP 125B), here designated); Roewer 1942: 255; Bücherl 1953: 128, figs 9–11 (misidentification, probably a Tapinauchenius); Bonnet 1955: 831; World Spider Catalog 2016.
Avicularia urticans Schmidt, 1994: 5, figs 1–2 (holotype female, from Peru, Krasa leg., 1989, SMF 38035 and spermathecae in microslides, SMF 58243-84 21/11, SMF 58243-84, examined), 1995c: 2, figs 1–2; World Spider Catalog 2016. **Syn. n.**

##### Remarks.

*Avicularia urticans* holotype is in poor conditions since the specimen died during moulting process. Its spermatheca is preserved in slides, but unfortunately it lost most of its natural shape. Despite this, it was possible to observe that spermatheca have midwidth expanded, about 1.5 times its basal and apical portion widths. Spermatheca morphology and overall body coloration match with large specimens found in Peru and Ecuador. Well-preserved material were examined and despite some differences in color features, *Avicularia urticans* is indistinguishable from *Avicularia juruensis*. Thus, we consider *Avicularia urticans* Schmidt, 1994 as junior synonym of *Avicularia juruensis* Mello-Leitão, 1923.

**Figures 106–108.**
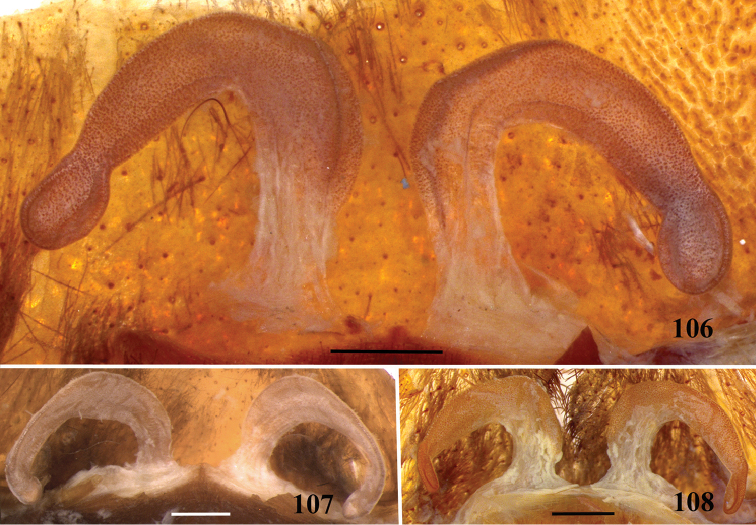
*Avicularia juruensis* Mello-Leitão, 1923, spermathecae variation. **106** paralectotype, Rio Juruá, state of Amazonas, Brazil (MZUSP 125C) **107** paralectotype, Rio Juruá, state of Amazonas, Brazil (MZUSP 125A) **108** morphotype 3, Pebas, department of Loreto, Peru (MNHN–AR 4902). Scale bars = 1 mm.

##### Diagnosis.

Females of *Avicularia juruensis* resemble those of *Avicularia variegata* stat. n. and *Avicularia taunayi* by the spermathecae having midwidth expanded, about 1.5 times its basal and apical portion widths (Fig. 106). They can be distinguished from *Avicularia taunayi* by the spermathecae lacking lobes (Fig. 106). Males of *Avicularia juruensis* resemble those of *Avicularia avicularia*, *Avicularia rufa, Avicularia variegata* stat. n., *Avicularia taunayi*, *Avicularia purpurea*, and *Avicularia merianae* sp. n. by the tibial apophysis on leg I with well-developed base and grouped spiniform setae distally (Fig. 114). They differ from males of all these species except *Avicularia variegata* stat. n. by its well-developed prominence on tegulum (Fig. 111). Males and females of *Avicularia juruensis* can be distinguished from *Avicularia variegata* stat. n. by intense purple sheen on carapace and legs (morphotype 2, Figs 120–121) or intense golden sheen on carapace and legs (morphotype 1, Fig. 122). They can also be distinguished from *Avicularia variegata* stat. n. by the occurrence area: western part of South America, in Brazil, Colombia, Ecuador, and Peru (Fig. 76).

##### Additional material.

COLOMBIA: *Vaupés*: Vaupés [0°5'N, 70°48'W], low Río Apaporis, Lago Toraima, Estación Biológica Caparu, 200 m asl, 1 male, Col. Jaime Pinzól (AP3-5) (ICN–Ar-2006); *Putumayo*: Puerto Leguízamo, Parque Nacional Natural La Paya [0°28'N, 75°49'W], Mamansoyá (Mamangaya [*sic*]), 1 male, 21 September 2001, D. Campos col. (ICN–Ar-1972); *Amazonas*: Letícia, km 2 Via Taparaca (trapaca [*sic*]), (4°12'19.25'S, 69°55'58.07"W), 100 m asl, 1 male, Col. Est. Sist. Anim. I-2002, 25 April 2002 (13001) (ICN–Ar-1970); [4°12'S, 69°56'W], km 11, Carretera a Tarapaca, bosque en interior de hija enrollada con casulo de seda a 50 m del suelo, coleta manual, 100 m asl, 1 immature, E. Flórez col., 27 October 1997 (ICN–Ar-1978); km 10, via Terapacos, Finca La Arerosa, 95 m asl, 1 immature, Col. Est. Sist. Animal II.03, 06 November 2003 (ICN–Ar-2369); La Pedrera, Resguardo Indígena Curaril–Los Ingleses, colectada em el interior de una vivenda, em horas nocturnas, 1 male, Z. Cordero col., 24 April 2004 (ICN–Ar-6819); cerca de Letícia [4°12'S, 69°56'W], 100 m asl, D. Campos col., August 1997 (ICN–Ar-5002); BRAZIL: *Amazonas*: between Benjamin Constant [4°22'S, 70°01'W] and São Paulo de Olivença [3°22'S, 68°52'W], 1 male, P. L. Conti col., 3 August 1972, ref. 9971 (IBSP 2829); Alto Solimões, 2 males, 31 August 1972 (IBSP 3389); Igarapé Belém, near confluence with Rio Solimões [3°05'S, 60°08'W], 1 male, B. Malkin col., 5–30 April 1966 (AMNH 1.29); Rio Negro [3°09'S, 59°57'W], 1 female, J. Coffey col., September 1994 (AMNH RW31); Carauari, left margin of Rio Juruá, Comunidade Esperança, RESEX Médio Juruá (05°05'31"S, 67°10'03"W), 1 male, F. F. Xavier Filho & A. L. Henriques col., 27 June to 16 July 2005 (INPA 4886); *Pará*: Breves [1°40'S, 50°28'W], margem W, Área 2, 1 male, J. Dias col., 2 February 1988 (MPEG 5398); *Acre*: Cruzeiro do Sul [7°37'S, 72°40'W], 1 female, S. Albuquerque (by photo); ECUADOR: 1 female, M. Baumgarten leg., 1994 (IBSP 12887); *Napo*: Parque Nacional Yasuní, Catholica Field Station (0°40'54"S, 76°23'9.33"W), 2 males, A. I. Ognato col., 15 July 1996 (CAS 6, CAS 4); Puerto Napo, 20 km east, Aliñahuí (1°0'S, 77°25'W), 450 m, 1 immature male, V. D. & B. Roth col., January 1994 (CAS 11A); 1 immature male, *Avicularia urticans* det. R. West in August 94, V. D. & B. Roth col. (CAS 8); 1 male, *Avicularia urticans* det. R. West in August 94, V. D. & B. Roth col., June 1994 (CAS 5); Puerto Napo, 25 km East, Selva Aliñahuí [1°0'S, 77°25'W], 450 m, 2 immature males, E. Ross col., January–February 1991; Avicularia sp. near juruensis det. J. Ledford 1997 (CAS); *Pastaza*: Tiguino [1°12'S, 7°51'W], 1 female, W. Lamar col., September 1990 (AMNH RW53); *Morona-Santiago*: Los Tayos, (3°05'S, 78°02'W), 1 female, in grass area near mil. camp, in afternoon, 5 July 1976 (IBSP 12884); same locality, in hole in tree trunk live but part rotten c.1 m from base, 1 male (IBSP 12888); PERU: spermathecae in microslides, no further information (SMF 58257-84); Rio Bombo, alto Tapiche, 1 female, 2 immatures, H. Bassler col, January 1928 (AMNH Pe55); R. Marañon [6°24'S, 76°05'W], 1 female, Bristol [col.?], October 1927 (AMNH Pe5); Marañón (Marauon [*sic*]),1 female,1 immature male, Bristol [col.?], October 1927 (AMNH Pe96); no data, probably Loreto, INRENA confiscation, 1 female (UA 088/2004); Peruvian jungle, confiscation, 1 male (UA 098/2004); *Loreto*: no further information, 1 female, Collection Bluntschili-Peyes, 1912 (AMNH Pe115); Cashiboya [7°39'S, 74°55'W], 1 female, February 1927 (AMNH Pe111); Estirón [4°07'S, 70°43'W], Rio Ampiacu, 3 females, 1 male, 2 juveniles, B. Malkin col., 15–22 May 1966 (AMNH Pe58); Iquitos [3°44'S, 73°15'W], 1 female, J. Huff col., November 1995 (AMNH RW51); Iquitos, Rio Momon, Amazon Camp (3°41'13.00"S, 73°16'48'00"W), 2 females, 1 male, R. C. West col., 9 November 1993 (AMNH RW35, AMNH RW36, AMNH RW37, respectively); 1 male, T. Mason col., March 1993 (AMNH RW39); 2 females, R. C. West col., 6 November 1993 (AMNH RW40, AMNH RW41); 1 male, E. Cooper col., May 1993 (AMNH RW43); Pebas [3°19'S, 71°51'W], 1 female, De Mathan col. (MNHN–AR 4902); Rio Tahuayo [4°53'S, 73°08'W], 1 male, W. Lamar col., September 1990, found parasite on forest floor (AMNH RW46); *San Martín*: Tarapoto [06°07'S, 75°57'W], 1 female, (UA 668/2005); Valley of Cainarachi, 40 miles east of Tarapoto, 1 female, 700–1500 m a.s.l., December 1925 (AMNH Pe54); *Ucayali*: Pampa Hermosa, Rio Ucayali [7°34'S, 74°19'W], 1 male, 1 female, January or June 1927 (AMNH Pe34); (Pompa Hermosa [*sic*]; Ucayoli [*sic*]), 8 females, February 1927 (AMNH Pe116); Rio Utiquinea [8°131'S, 74°32'W] (Upper Utoguinia [*sic*]), La frontera, 1 juvenile female, H. Bassler col., 1928 (AMNH Pe113); (Rio Utuguinea [*sic*]), Peru–Brazil frontier, 1 immature, August 1927 (AMNH Pe109); Crenze Zwischen Peru und Brazil, sud Crenze, Beim Uberer Utoquinia, 1 immature male, “Scolopenda”, 10 February 1928 (AMNH 1.10); Río Ucayali [7°34'S, 74°19'W], Suhuaya + Rean Rean [?], 2 juvenile females, 1 immature male, 12–16 December 1926 (AMNH Pe122).

**Figures 109–116.**
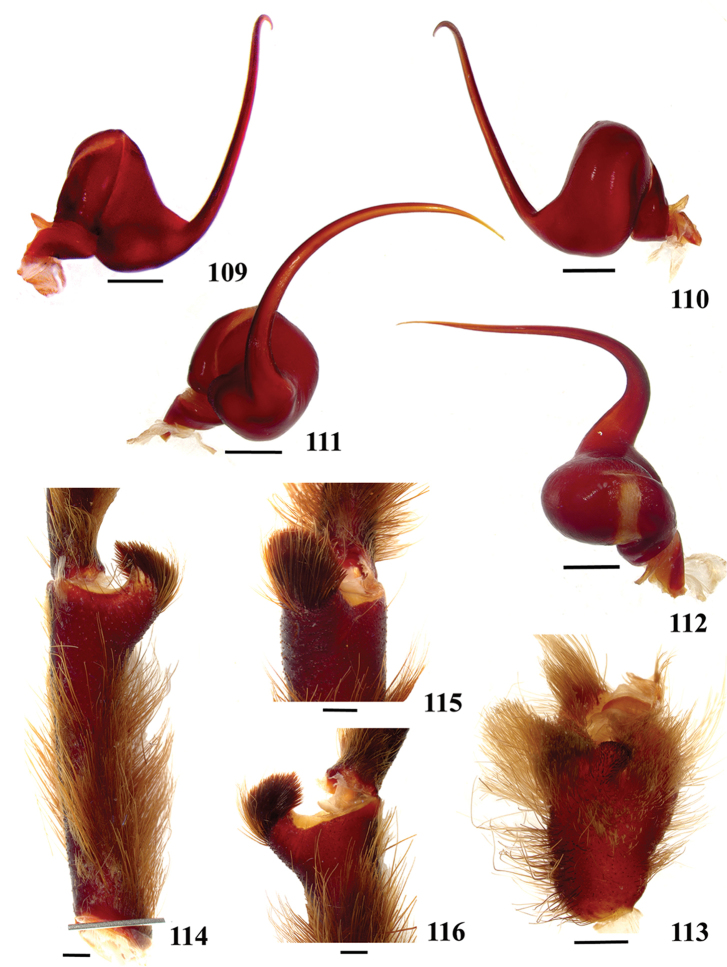
*Avicularia juruensis* Mello-Leitão, 1923, male lectotype (MZUSP 125B). **109–112** left palpal bulb **109** prolateral **110** retrolateral **111** frontal **112** dorsal **113** left cymbium, dorsal **114–116** right tibial apophysis of leg I (mirrored) **114** prolateral **115** ventral **116** retrolateral. Scale bars = 1 mm.

**Figures 117–122.**
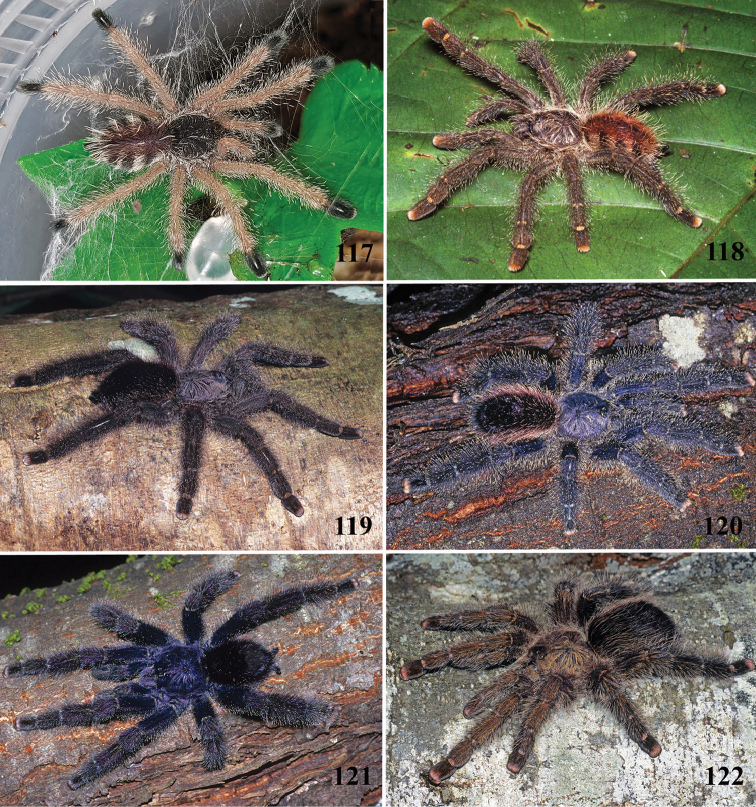
*Avicularia juruensis* Mello-Leitão, 1923, habitus. **117–118** morphotype 2 **117** immature **118** juvenile **119** female with yellowish leg ring, department of Napo, Ecuador **120** female with whitish leg ring, department of Loreto, Peru **121** male, department of Loreto, Peru **122** morphotype 1, female, Peru. Photos: **117** Tanya Stewart; **118** W. Lamar; **119–122** R. C. West.

##### Male.

**Description.** MZUSP 125C. Carapace: 15.23 long, 15.31 wide, 4.67 high. Chelicera: 5.60 long. Legs (femur, patella, tibia, metatarsus, tarsus, total): I: 15.92, 8.47, 12.61, 11.81, 7.31, 56.12. II: lost. III: lost. IV: 16.63, 6.87, 14.78, 15.72, 6.16, 60.16. Palp: 9.11, 6.03, 8.09, –, 3.51, 26.74. Midwidths: femora I–IV= 3.21, –, –, 3.08, palp= 2.23; patellae I–IV= 2.74, –, –, 2.60, palp= 1.91; tibiae I–IV= 2.34, –, –, 2.43, palp= 2.18; metatarsi I–IV= 1.54, –, –, 1.70; tarsi I–IV= 1.67, –, –, 1.93, palp= 2.06. Abdomen: 16.66 long, 12.25 wide. Spinnerets: PMS, 1.20 long, 0.54 wide, 0.23 apart; PLS, 1.83 basal, 1.79 middle, 2.96 distal; midwidths 1.41, 1.41, 0.93, respectively.

Carapace: as long as wide; cephalic region slightly raised, thoracic striae conspicuous.

Fovea: deep, slightly recurve, 2.27 wide.

Eyes: ocular tubercle 1.05 high, 2.19 long, 3.22 wide. Clypeus 0.56. Anterior eye row procurve. Posterior slightly recurve. Eye size and interdistances: AME 0.69, ALE 0.77, PME 0.25, PLE 0.65, AME–AME 0.59, AME–ALE 0.51, AME–PME 0.22, ALE–ALE 2.14, ALE–PME 0.95, PME–PME 1.97, PME–PLE 0.21, PLE–PLE 2.47, ALE–PLE 0.54, AME–PLE 0.56.

Maxilla: length to width: 2.31. Cuspules: 100–200 spread over ventral inner heel. Labium: 2.51 long, 2.75 wide, with 87 cuspules spaced by one diameter from each other on anterior third. Labio-sternal groove shallow, flat, with no visible sigilla.

Sternum: 7.18 long, 6.71 wide. Sigilla: not evident.

Legs: Formula: IV=I – –. Length leg IV to leg I: 1.07. Clavate trichobothria: 2/3 distal on tarsi I, IV. Scopulae: Tarsi I and IV fully scopulate. IV lacking sparse setae. Metatarsi I fully scopulate, II–III ?; IV on distal 1/3. IV divided by a row of setae.

Type II urticating setae: 0.94–1.01 long, 0.017–0.021 wide.

Palp (Figs 109–112): globous bulb with small subtegulum and well-developed prominence on tegulum. Embolus: not flattened, without keels, 5.02 long in retrolateral view, about 3.0 times tegulum’s length. Medial portion and tegulum’s margin form an acute angle in retrolateral view. Proximal part very curved in frontal view; thin distal width, abruptly narrowing distally; basal, middle, and distal width of 0.89, 0.17, 0.03, respectively. Tegulum: 1.62 long, 2.72 high in retrolateral view. Cymbium subtriangular with subequal lobes, and well-developed rounded process on retrolateral lobe, bearing thick setae (Figs 113, 307).

Tibial apophysis (Figs 114–116): single branch on prolateral leg I, with well-developed base and grouped spiniform setae distally. Male metatarsus I touches retrolaterally tibial apophysis’ setae when folded.

Color pattern: carapace brown with golden short body setae. Carapace border with long setae the same color as dorsal carapace short body setae. Coxae, labium, sternum and maxillae brown, slightly darker than ventral femora. Legs and palps with golden brown short body setae and brown long dark guard-setae. Leg rings on distal femora, tibiae and metatarsi whitish. Abdomen dorsum with reddish brown guard-setae and black short body setae. Ventral abdomen brown.

##### Female.

**Redescription.** MZUSP 125B. Carapace: 19.26 long, 16.94 wide, 5.22 high. Chelicera: 8.77 long. Legs (femur, patella, tibia, metatarsus, tarsus, total): I: 15.30, 9.13, 11.53, 9.92, 6.66, 52.54. II: 14.21, 8.26, 10.39, 10.37, 6.25, 49.48. III: 12.99, 7.49, 9.86, 9.68, 6.43, 46.45. IV: 15.75, 8.48, 13.59, 13.19, 6.62, 57.63. Palp: 10.61, 6.74, 7.07, –, 8.34, 32.76. Midwidths: femora I–IV= 3.64, 3.71, 3.84, 3.45, palp= 2.84; patellae I–IV= 3.55, 3.68, 3.69, 3.62, palp= 2.90; tibiae I–IV= 2.97, 3.13, 3.00, 2.72, palp= 2.88; metatarsi I–IV= 2.34, 2.67, 2.21, 2.20; tarsi I–IV= 2.89, 2.44, 2.80, 2.62, palp= 2.78. Abdomen: 24.29 long, 16.59 wide. Spinnerets: PMS, 2.32 long, 1.54 wide, 0.1 apart; PLS, 3.43 basal, 2.14 middle, 2.86 distal; widths 1.82, 1.45, 1.28, respectively.

As in male, except:

Carapace: 1.14 times longer than wide.

Fovea: 2.19 wide.

Eyes: eye tubercle 1.15 high, 2.76 long, 3.56 wide. Clypeus 0.48. Eye size and interdistances: AME 0.78, ALE 0.80, PME 0.34, PLE 0.75, AME–AME 0.64, AME–ALE 0.56, AME–PME 0.29, ALE–ALE 2.53, ALE–PME 0.91, PME–PME 2.13, PME–PLE 0.14, PLE–PLE 2.95 ALE–PLE 0.66, AME–PLE 0.66.

Maxilla: length to width: 1.70. Cuspules: 100–200 spread over ventral inner heel. Labium: 2.00 long, 2.71 wide, with 88 cuspules spaced by one diameter on anterior third.

Chelicera: basal segment with 10 teeth. Sternum: 8.69 long, 7.79 wide.

Legs: Formula: IV=I II III. Length leg IV to leg I: 1.10. Clavate trichobothria: 2/3 distal on tarsi I–IV. Scopulae: Tarsi I and IV fully scopulate. IV lacking sparse setae. Metatarsi I–II fully scopulate, III on distal 2/3; IV on distal 1/3. III divided by a bald area, IV divided by a row of setae.

Type II urticating setae: 0.54–0.66 long, 0.014–0.018 wide (measured MZUSP 125A).

Spermathecae (Fig. 106): two completely separated, not-twisted very long spermathecae, with walls lacking projections or lobes and accentuated outwards curvature medially. Midwidth expanded, about 1.5 times its basal and apical portion widths and weakly-sclerotized area shorter than half the length of well-sclerotized area.

Color pattern: dorsal abdomen with long brown guard-setae grouped on lateral and dorsal anterior areas, and dark short body setae.

##### Variation.

We found two different morphotypes. Morphotype 1 is found near Rio Juruá, state of Amazonas and Acre, Brazil, and in some areas of Peru (Fig. 127). Females have discrete grizzled setae in palp and legs, whitish leg rings and carapace, legs and palps with short body setae with golden and pink sheen, and abdomen with long light brown guard-setae homogeneously distributed over dark brown body short setae (Fig. 124). Males examined are not in good conditions but they do not seem to have white tipped setae on dorsal abdomen. Despite having yellowish leg rings, a female from Iquitos, Peru (Fig. 122) is considered as morphotype 1 since it has discrete grizzled guard-setae and short body setae with intense golden sheen on legs and carapace. Morphotype 2 is the most common morphotype found in the material examined and it was formerly known as *Avicularia urticans*. Both sexes have very grizzled setae on palps and legs, and carapace, legs and palps with dark short body setae with intense purple sheen, and yellowish leg rings (Figs 119–121). Females have dorsal abdomen with long reddish brown guard-setae grouped on lateral and dorsal anterior areas, and dark short body setae (Fig. 120). Males have white tipped setae homogenously distributed on dorsal abdomen (Fig. 121). They can be found mostly in Ecuador and Peru, but a specimen with the same characteristic pattern was found in Breves, state of Pará, Brazil.

**Figures 123–126.**
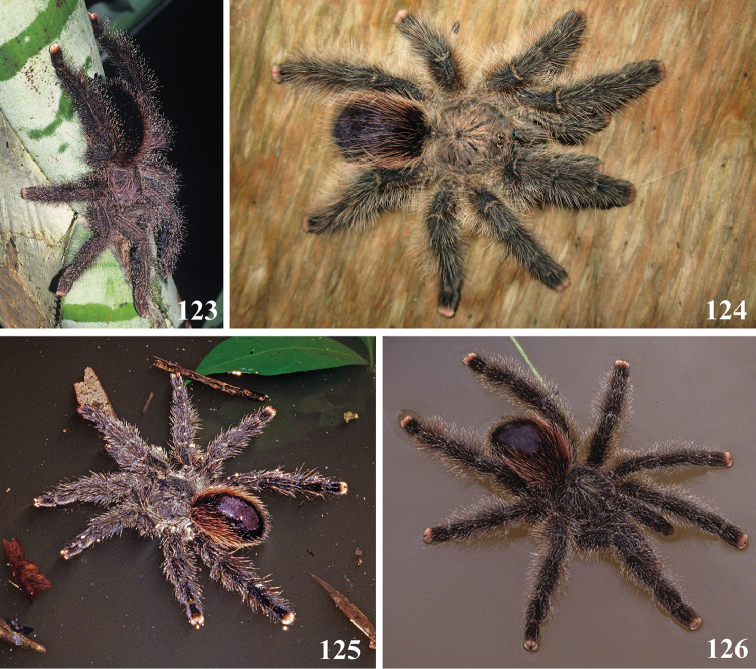
*Avicularia juruensis* Mello-Leitão, 1923, retreat, habitus and behavior. **123** morphotype 2, retreat **124** morphotype 1, female from Cruzeiro do Sul, state of Acre, Brazil, habitus **125** female swimming on Rio Momon, Peru **126** female swimming on Rio Marañon, Peru. Photos: **123, 125–126** R. C. West; **124** S. Albuquerque.

##### Remarks.

For many years the name *Avicularia juruensis* has been applied to specimens that have vivid yellow leg rings and grizzled setae on legs and palps, that are commonly found in the states of Mato Grosso and Rondônia, Brazil. They have spermathecae with midwidth not expanded, developed prominence on palpal bulb and leg IV longer than leg I. However, *Avicularia juruenesis* syntypes have whitish leg rings and lack setae with conspicuous whitish apex on legs, spermathecae with midwidth expanded, palpal bulb with well-developed prominence, and leg IV as long as leg I. The characters found in these specimens formerly known as *Avicularia juruensis* match, in fact, with those of *Avicularia rufa*. Thus, we conclude the name *Avicularia juruensis* is being mistakenly applied to specimens of *Avicularia rufa*.

**Figure 127.**
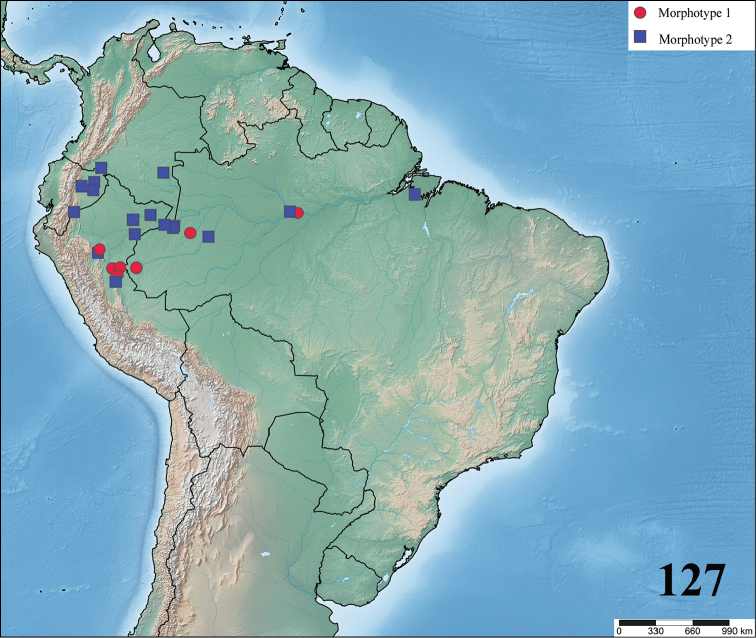
Map showing records of *Avicularia juruensis* Mello-Leitão, 1923 morphotypes.

##### Color pattern ontogeny.

Brownish juveniles lacking metallic sheen, black tarsi contrasting with other lighter articles and abdomen dorsum reddish, with dorsal central longitudinal black stripe disconnected from transversal black stripes (Fig. 117). When mature, both males and females lose this pattern.

##### Distribution.

Brazil (states of Amazonas, Acre and Pará), Colombia, Ecuador and Peru (Fig. 76).

##### Natural history.

Silken retreats of *Avicularia juruensis* are similar to other *Avicularia* species (Fig. 123). An adult female *Avicularia juruensis* was reported feeding on a greater sac-winged bat (*Saccopteryx bilineata* (Temminck, 1838)) on the side of a palm tree near Rio Yarapa, Peru (Nyffeler and Knörnschild 2013). Another interesting behavior was the ability to swim through large rivers such as Marañon and Momon rivers in Peru (Figs 125–126) (R. C. West, pers. comm.), which can explain why rivers do not seem to act as natural barriers to *Avicularia*.

#### Avicularia rufa

Taxon classificationAnimaliaORDOFAMILIA

Schiapelli & Gerschman, 1945

Figs 19 , 50 , 128–130 , 131–138 , 139–141 , 142–147 , 148–153 , 300 , 302

Avicularia rufa Schiapelli & Gerschman, 1945: 190, pls. XV–XVI, XXV (holotype female, Brazil, Mato Grosso, Alto Ji Paraná [10°52'N, 61°55'W] (Gy Paraná [*sic*]), Dr. Vellard col., December 1938, MACN–Ar 845, examined,); Brignoli 1983: 134; World Spider Catalog 2016.
Avicularia juruensis (misidentification): West et al. 2008: 37, 52–54; Ayroza et al. 2012: 2; Bertani 2012: 5, 79–80.

##### Diagnosis.

males and females of *Avicularia rufa* resemble *Avicularia avicularia* and female of *Avicularia hirschii* by the leg IV longer than leg I. Females of *Avicularia rufa* can be distinguished from those of *Avicularia hirschii* by the non-twisted spermathecae. Females and males of *Avicularia rufa* differ from those of *Avicularia avicularia* by having vivid yellow rings on distal femora, tibiae and metatarsi combined with legs and palps with very grizzled guard-setae (Fig. 145) (from all morphotypes of *Avicularia avicularia* except 6 and 7, but these have intense green metallic sheen on carapace and legs, absent in *Avicularia rufa*).

##### Material examined.

1 female, Brazil, Mato Grosso, between Vale de São Domingos [15°17'S, 59°03'W] (Vale de São Lourenço [*sic*]) and Pontes & Lacerda [15°12'S, 59°19'W], Operação Coatá, I. Knysak col., 14 December 2002, RGI 1963 (IBSP 10264) and 2 males and 1 immature female, Brazil, Mato Grosso, Operação Coatá col., 1–20 July 2002 (MCP 13592).

**Figures 128–130.**
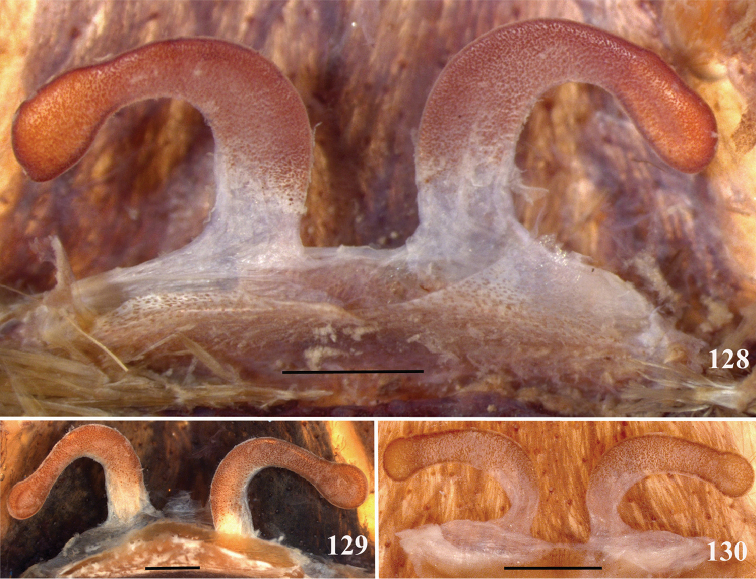
*Avicularia rufa* Schiapelli & Gerschman, 1945, spermathecae variation. **128** between vale de São Domingos and Pontes & Lacerda, state of Mato Grosso, Brazil (IBSP 10264) **129** Rio Negro, state of Amazonas, Brazil (AMNH RW29) **130** Tomé-Assu, state of Pará, Brazil (IBSP 3105). Scale bars = 1 mm.

##### Additional material.

BRAZIL: no further data: 1 female, 1 immature (IBSP 2157); 1 female (IBSP 3170); 7 immatures, G. Raidar col., 14 May 1951 (IBSP 2620); 1 female (IBSP 4368); 2 males (MZUSP 22501); 2 females (IBSP 1393); *Amapá*: Serra do Navio [1°38'N, 52°12'W], 2 immature males (IBSP 3703); *Amazonas*: Rodovia Transamazônica, km 530, 1 male, 23 October 1975 (IBSP 7895); Transamazônica Humaitá–Porto Velho, 1 immature, Dr. A. H. Hoge col., ref. 4102, July 1972 (IBSP 4102); Barcelos [0°57'S, 62°55'W], Parque Nacional do Jaú, Lago do Miratuca, 1 female, R. Andriazze, W. L. S. Costa & L. Aquino col., 14 July 1993 (INPA 4884); Boca do Jacaré [5°45'S, 63°41'W], Rio Solimões, 1 female (IBSP 3110); Boca do Tefé [3°19'S, 64°43'W], Rio Solimões, 1 female (IBSP 3104); 2 females, 1 immature male (IBSP 3099); 1 female, 3 immatures (IBSP ref.189); 1 male (IBSP 3101); 1 female (IBSP 3103); 1 immature male (IBSP 3102); 2 immatures (IBSP 3100); Boca do Tefé [3°19'S, 64°43'W], Rio Solimões, 5 females, 1 male, 2 immatures males, 5 immatures (IBSP 3104, IBSP 3099, IBSP ref. 189, IBSP 3103, IBSP 3101, IBSP 3102, IBSP 3100); Cucuí [1°11'N, 66°49'W], 1 juvenile female, P. Serveira col., November 1975, ref.17.595 (IBSP 2569); Humaitá [7°30'S, 63°01'W], 1 female, in silk retreat in a palm tree (IBSP 7932); 1 male, 4 females, L. R. Latorre col., November 1981, ref. 40348 (IBSP 4686, IBSP 4687); pottery and pineapple crop, 1 male, 1 juvenile female, 1 immature male, 2 immatures, Dr. A. R. Hoge col., July 1972, IB 24.8.72 (IBSP 4099); Transamazônica, km 86, 1 male, 23 July 1972 (IBSP 4100); Puruzinho, Rio Madeira [6°52'S, 62°05'W], 1 female, EPA col., 4 December 1975 (MZUSP 27610); Rio Negro [3°09'S, 59°57'W], 2 males, 1 female, A. McKee col., 19 April 1989 (AMNH RW67, AMNH RW25, AMNH RW27); 2 females, A. McKee col., 29 June 1988 (AMNH RW29, AMNH RW28); 1 immature male, R. Mascarino col., 18 September 1996 (AMNH RW30); Tapera [0°55'N, 67°26'W], Rio Negro, 1 immature male, EPA col., 4 November 1972, P. E. Vanzolini col., 722574 (MZUSP unnumbered); Tefé [3°20'S, 64°43'W], 1 male, 1 female, De Mathan col. (MNHN–AR 4903); 3 females, 1 immature (IBSP 193); 2 immature females, 2 immatures (IBSP 184); *Pará*: Belém [1°27'S, 48°30'W], IAN, 1 immature male (IBSP 3123); Belterra [3°07'S, 55°03'W], 1 female, 15 March 2003 (MPEG 4800); Itaituba [4°16'S, 55°59'W], Rio Maropa, 1 male, W. G. Ravem col., ref. 41844 (IBSP 8576); 1 female, dead 25 April 1990 (IBSP ref. 41.844); Óbidos [1°54'S, 55°31'W], Igarapé Jaramacaru, Campos do Ariramba, 1 immature male, EPA col., 24 January 1960 (MZUSP 14873); São Félix do Xingu [6°37'S, 51°58'W], 1 male, 1 female, G. Whitaker col., July 1980, ref. 28300 (IBSP 4562); Tomé-Assu [2°25'S, 48°09'W] (Thomé Assu [*sic*]), Rio Acará Mirim, 1 female (IBSP 3105); Tucuruí [3°46'S, 49°40'W], 1 female, Equipe de Resgate de Fauna col., Ref. IBA 218 (IBSP 8574); Vila Bravo, 1 female, Equipe de Resgate de Fauna col., ref. B410-12C (IBSP 8577); *Acre*: Rio Branco [9°58'S, 67°48'W], Catuaba, 1 immature male, A03, April 1996 (IBSP unnumbered); Marechal Thaumaturgo, Terra Indígena Jaminawa-Arara, Rio Bagé, Aldeia Buritizal, [8°56'S, 72°47'W], 1 immature, 02 September 2000, 9AM, Coleção de Simone Ladeia Andrade (MZUSP 70947); *Rondônia*: Candeias do Jamari, UHE Samuel [8°45'S, 63°27'W], 1 female, W. Bokerman col., June 1989 (AMNH RW24); Jaru [10°26'S, 62°28'W], Santa Cruz da Serra, 2 females, Expedição Polo Noroeste col., 23–27 December 1964 (MZUSP 11065); Monte Negro [10°14'S, 63°17'W], 1 immature male, 1 female, 1 juvenile female, R. Bertani & P. I. da Silva Junior col., 22 July to 03 August 2002 (IBSP 10042, IBSP 10218, IBSP 10945, respectively); Núcleo Avançado de Pesquisa de Monte Negro, 1 male, L. M. A. Camargo col., June 2002, ref. 89431 (IBSP 1028); 1 juvenile female, L. M. A. Camargo col., 06 April 2002, ref. 89430 (IBSP 10205); Porto Velho [8°43'S, 63°53'W], 1 male, G. Insley col., 31 August 1973, ref. 12732 (IBSP 2454); 1 male, G. Insley col., 9 October 1973, ref. 12922 (IBSP 2483); Santa Luzia d’Oeste [11°54'S, 61°46'W], 1 immature female, R. Moterani col., July 1999 (IBSP 9538); *Mato Grosso*: Alto Xingu [7°16'S, 52°36'W], 1 male, H. Schulze col., 21 October 1964 (IBSP 3693); Barra do Bugres [15°03'S, 57°10'W], Cia Paulista de Ferro Ligas, 1 immature, 26 July 1971, ref. 7624 (IBSP 4103); 1 immature, A. Cerrutti col., November 1984 (MNRJ 12942); Chapada dos Guimarães [15°26'S, 55°44'W], UHE Rio Manso, 1 female, Faunal Rescue Team of Furnas col., 2000 (IBSP 9087); 1 female, H. N. da Cunha col., December 1976 (IBSP 13805); Rio Kuluene (Koluene [*sic*]) [13°22'S, 52°59'W], 1 immature male, J. M. C. Carvalho col., 1947 (MNRJ 13.773); Lagoa Ipavu [12°7'S, 53°26'W] (Lagoa Ipavi [*sic*]), 1 immature male, 1 juvenile female, P. Vanzolini col., 1965 (MZUSP E3474 C3918); Nova Mutum [13°49'S, 56°05'W], 1 female, R. K. Ribeiro col., 06–15 July 2001 (IBSP 10929); Parque Nacional Xingu [11°59'S, 54°00'W], 1 male, C. Andreatta col., March 1968 (IBSP 39A); 2 immatures, Alvarenga & Werner col., November 1961 (AMNH 1.19); São José do Rio Claro [13°25'S, 56°42'W], 2 males, M. Caleffo col., June 1997 (IBSP 10309); Sinop [11°52'S, 55°29'W], 1 immature, 4 March 1977, ref. 20109 (IBSP 4455); Vila Bela da Santissima Trindade [15°00'S, 59°57'W] (Villebela [*sic*]), Rio Serra Azul, 1 male, Dr. Hoge col., 27 June 1962 (IBSP 3614); Maloca Feia, 1 female, Dr. Hoge, Pedro & Joaquim col., 27 June 1962 (IBSP 3615); Between Vale de São Domingos [15°17'S, 59°03'W] ([*sic*] Vale de São Lourenço) and Pontes & Lacerda [15°14'S, 59°19'W], UHE Guaporé, Operação Coatá, I. Knysak col.: 1 juvenile female, 17 May 2002, RGI 074 (IBSP 10274); 1 juvenile female, Operação Coatá, I. Knysak col., 18 May 2002, RGI 086 (IBSP 10253); 1 juvenile female, Operação Coatá, I. Knysak col., 14 September 2002, RGI 1871 (IBSP 10262); 2 females, Operação Coatá, I. Knysak col.,1 October 2002, RGI 2527, RGI 2528 (IBSP 10241, IBSP10242, respectively); 2 females, 5 juvenile females, 3 immature males, Operação Coatá, I. Knysak col., 03 October 2002, RGI 2299, RGI 2516, RGI 2313, RGI 2518, RGI 2300, RGI 2314, RGI 2517, RGI 2315, RGI 2316, RGI 2302 (IBSP 10232, IBSP 10224, IBSP 10246, IBSP 10226, IBSP 10233, IBSP 10247, IBSP 10225, IBSP 10248, IBSP 10249, IBSP 10235, respectively); 1 female, Operação Coatá, I. Knysak col., 05 October 2002, RGI 1871 (IBSP 10220); 1 immature female, Operação Coatá, I. Knysak col., 7 October 2002, RGI 1871 (IBSP 10230); 1 female, 2 juvenile females, Operação Coatá, I. Knysak col., 08 October 2002, RGI 1773, RGI 1774, RGI 1775 (IBSP 10275, IBSP 10279, IBSP 10278, respectively); 1 immature, S2, Operação Coatá, I. Knysak col., 8 October 2002 (IBSP unnumbered); 2 females, 1 juvenile female, 1 immature male, Operação Coatá, I. Knysak col., 14 October 2002, RGI-, RGI 1962, RGI 1964, RGI 1966 (IBSP 10256, IBSP 10263, IBSP 10255, IBSP 10257, respectively); 1 female, Operação Coatá, I. Knysak col., 01 December 2002, RGI 2526 (IBSP 10240); 1 immature male, 03 December 2002, RGI 2301 (IBSP 10234); U.H.E. Guaporé [15°16'S, 59°04'W], Operação Coatá, 2 females, 1 juvenile female, 4–14 October 2002 (MCP 13595); 1 immature male, 2 immatures, Operação Coatá, 14 October 2002 (MCP 13598); 4 immatures, Operação Coatá, 10 June 2002 (MCP 13593); 3 females,1 immature female, Operação Coatá, 14 February 2002 (MCP 13596, MCP 13599); 1 immature, Operação Coatá, 01–07 September 2002 (MCP 13555); 2 immature males, 1 female, Operação Coatá, 4–14 December 2002 (MCP 13594); 1 female, 1 immature, Operação Coatá, 14 December 2002 (MCP 13597); Xingu, Suia-Missu [11°39'S, 51°25'W] (Tuiã Missu [*sic*]), 5 immatures, Whytaker col., 05 February 1980, ref. 27055 (IBSP 4523); ECUADOR: *Napo*: Puerto Napo [1°01'S, 77°43'W], 25 km east, Selva Aliñahuí, 450 m, 1 juvenile female, E. S. Ross col., March 1992 (CAS 9); PERU: *Madre de Dios*: Iñapari [10°57'S, 69°34'W], Esperanza, 2 females, 4 immature males, 245 m a.s.l., average temperature 32.4°C, J. Morant Araque col., 22 September 1992 (MUSM); *Junin*: Rio Tambo [11°10'S, 74°14'W], Shevaja, in short palm trees, 3 females, P. Hocking col., 12 October 2011 (MUSM); 300 m a.s.l., in palm trees, 1 female, P. Hocking col., 23 October 2010 (MUSM); 1 female, P. Hocking col., October 2010 (MUSM–ENTO 504260); 1 female, H. Bassler col., February 1928 (AMNH Pe121); *Cusco*: Cashiriari (11°52'S, 72°39'W), 1 female, S. Córdova col., 25 November 1997 (MUSM–ENTO); Cusco [department or city?], Timpia Rio Urubamba [12°28'S, 72°29'W], 1 female, P. Hocking & L. Campos col., March 2009 (MUSM–ENTO 500676); BOLIVIA: *Santa Cruz*: San Ignácio de Velasco [16°22'S, 60°55'W], 1 female (IBSP 3552).

**Figures 131–138.**
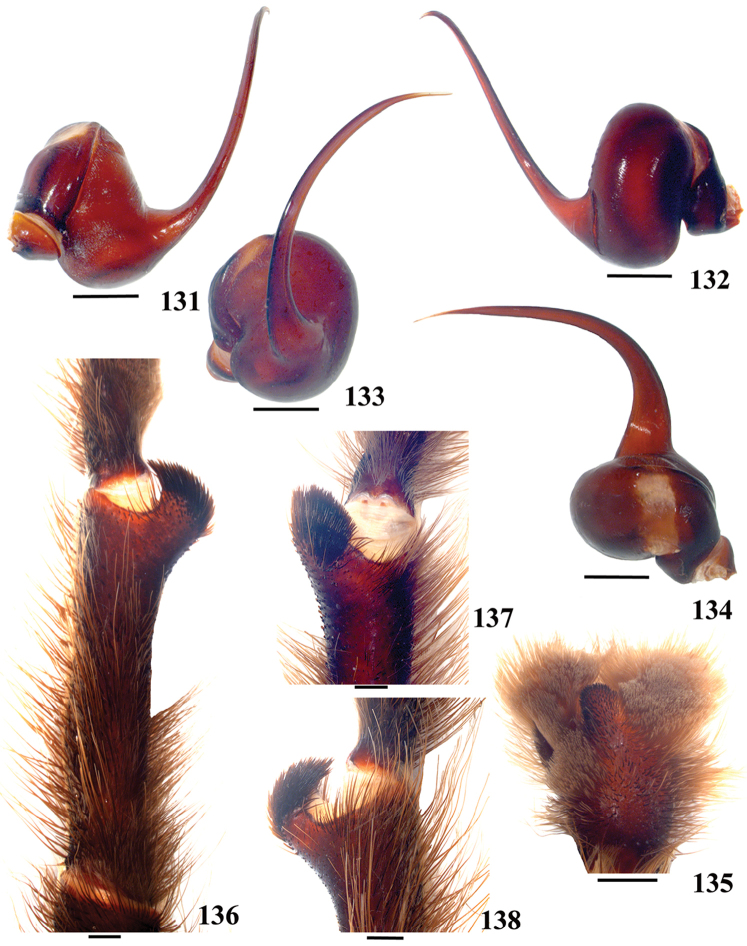
*Avicularia rufa* Schiapelli & Gerschman, 1945, male (MCP 13592). **131–134** right palpal bulb (mirrored) **131** prolateral **132** retrolateral **133** frontal **134** dorsal **135** right cymbium, dorsal (mirrored) **136–138** right tibial apophysis of leg I (mirrored) **136** prolateral **137** ventral **138** retrolateral. Scale bars = 1 mm.

**Figures 139–141.**
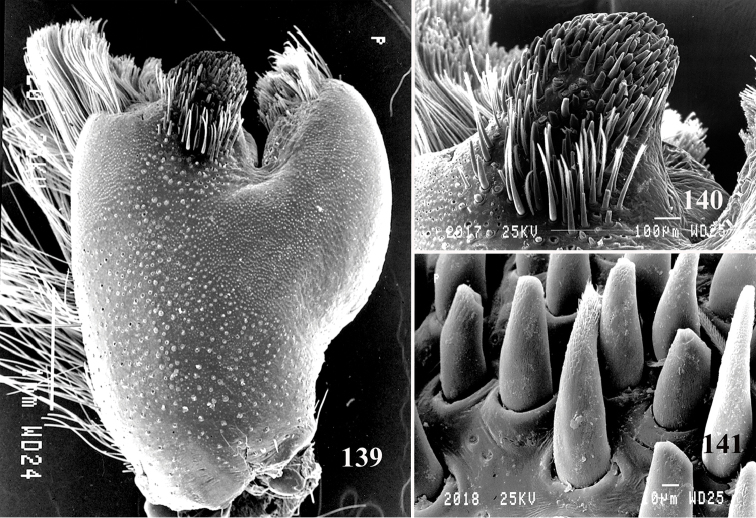
SEM microphotographs of cymbium process in male of *Avicularia rufa*. **139** dorsal view **140** process, detail, dorsal **141** well-developed setae of process.

##### Female.

**Redescription.** IBSP 10264. Carapace: 17.53 long, 17.69 wide, 4.26 high. Chelicera: 7.70 long. Legs (femur, patella, tibia, metatarsus, tarsus, total): I: 15.59, 8.80, 11.42, 9.78, 6.41, 51.63. II: 14.52, 8.76, 10.23, 9.58, 6.86, 49.95. III: 13.61, 7.99, 10.21, 10.37, 6.14, 48.32. IV: 17.59, 8.68, 15.93, 14.81, 6.67, 63.68. Palp: 10.20, 6.51, 7.18, –, 7.48. Midwidths: femora I–IV= 3.69, 3.18, 3.42, 3.68, palp= 2.85; patellae I–IV= 3.44, 3.41, 3.36, 3.78, palp= 2.96; tibiae I–IV= 2.89, 3.18, 3.02, 3.24, palp= 2.80; metatarsi I–IV= 2.61, 2.51, 2.82, 2.73; tarsi I–IV= 2.89, 2.93, 2.95, 3.05, palp= 2.95. Abdomen: 24.70 long, 17.11 wide. Spinnerets: PMS, 2.73 long, 1.44 wide, 0.4 apart; PLS, 3.31 basal, 1.56 middle, 3.78 distal; midwidths 2.44, 2.06, 1.61, respectively.

Carapace: 0.99 times longer than wide; cephalic region not raised, thoracic striae inconspicuous.

Fovea: deep, slightly recurved, 3.52 wide.

Eyes: eye tubercle 1.61 high, 2.71 long, 3.39 wide. Clypeus 0.18. Anteriore eye row procurve. Posterior eye row slightly recurve. Eye sizes and interdistances: AME 0.78, ALE 0.87, PME 0.32, PLE 0.76, AME–AME 0.56, AME–ALE 0.42, AME–PME 0.22, ALE–ALE 2.13, ALE–PME 0.56, PME–PME 2.15, PME–PLE 0.18, PLE–PLE 2.90, ALE–PLE 0.28, AME–PLE 0.64.

Maxilla: length to width: 1.69 longer than wide. Cuspules: 100–200 spread over ventral inner heel. Labium: 2.02 long, 3.41 wide, with 133 cuspules spaced by one diameter from each other on anterior third. Labio-sternal groove shallow, flattened, without evident sigilla.

Chelicera: basal segment with 10 teeth in a row and some small teeth on promargin. Sternum: 7.75 long, 7.45 wide. Sigilla: rounded posterior, less than one diameter from margin; other sigilla not evident.

Leg: Formula: IV I II III. Length leg IV to leg I: 1.23. Clavate trichobothria: distal 2/3 tarsi I–IV. Scopula: Tarsi I–IV fully scopulate. Metatarsi I fully scopulate; II 4/5; III 1/3 and IV 1/2 distal scopulate. IV divided by a row of setae.

Type II urticating setae: 0.50–0.58 long, 0.013–0.019 wide (measured from IBSP 10233).

Spermathecae (Fig. 128): two completely separated, not-twisted long spermathecae, with walls lacking projections or lobes and accentuated outwards curvature medially. Midwidth as wide as its base width and weakly-sclerotized area shorter than half the length of well-sclerotized area.

Color pattern (Fig. 145): carapace brown with golden short body setae with sligthtly purple sheen. Carapace border with long setae the same color as dorsal carapace short body setae. Coxae, labium, sternum and maxillae darker than ventral femora. Legs and palps with brown short body setae having pink sheen and brown long guard-setae with darker base and contrasting whitish apex. Leg rings on distal femora, tibiae and and metatarsi vivid yellow (Fig. 300). Abdomen with long greyish brown guard-setae grouped on lateral and dorsal anterior areas and dark short body setae (Fig. 302). Abdomen venter brown.

##### Male.

**Description.**

MCP 13592 (larger specimen). Carapace: 14.15 long, 14.45 wide, 3.3 high. Chelicera: 6.6 long. Legs (femur, patella, tibia, metatarsus, tarsus, total): I: 13.56, 7.50, 11.67, 11.46, 7.55, 51.74. II: 13.02, 7.16, 11.14, 11.15, 6.45, 48.92. III: 13.44, 6.45, 10.55, 11.46, 6.66, 48.59. IV: 18.14, 7.35, 14.16, 13.55, 8.15, 61.65. Palp: 3.62, 3.45, 8.51, –, 3.54, 19.12. Midwidths: femora I–IV= 2.62, 3.07, 2.65, 5.56; palp= 2.16; patellae I–IV= 3.50, 3.15, 2.56, 2.66, palp= 2.42; tibiae I–IV= 2.66, 2.25, 2.26, 2.46, palp= 2.15; metatarsi I–IV= 1.67, 1.75, 1.70, 1.13; tarsi I–IV= 2.12, 2.15, 2.26, 2.33. palp= 2.16. Abdomen: 19.07 long, 13.05 wide. Spinnerets: PMS, 1.90 long, 0.98 wide, 0.2 apart; PLS, 2.70 basal, 1.40 middle, 2.95 distal; midwidths 1.50, 1.40, 1.16 respectively.

As in female, except:

Carapace: 0.98 times longer than wide.

Fovea: 2.55 wide.

Eyes: eye tubercle 1.3 high, 2.56 long, 3.00 wide. Clypeus 0.34. Eye size and interdistances: AME 0.75, ALE 0.80, PME 0.28, PLE 0.50, AME–AME 0.58, AME–ALE 0.37, AME–PME 0.19, ALE–ALE 1.92, ALE–PME 0.63, PME–PME 1.92, PME–PLE 0.11, PLE–PLE 2.39, ALE–PLE 0.31, AME–PLE 0.52. Ratio of the eye group width to length 1.84.

Maxilla: length to width: 1.94. Labium: 1.79 long, 2.45 wide, with 109 cuspules spaced by one diameter from each other on anterior third.

Chelicera: basal segment with 12 teeth in a row and some small teeth on promargin. Sternum: 7.72 long, 6.44 wide. Sigilla: anterior rounded, middle fusiform, both less than one diameter from margin.

Legs: Length leg IV to leg I: 1.19. Scopula: Tarsi I–IV fully scopulate. Metatarsi I–II 3/4; III 1/2, IV 1/3 distal scopulate. IV divided by a bald area.

Type II urticating setae: 1.01–1.11 long, 0.021–0.023 wide.

Palp (Figs 131–134): globous bulb with small subtegulum and developed prominence on tegulum. Embolus: not flattened, lacking keels, 4.38 long in retrolateral view, about 3 times tegulum’s length. Medial portion and tegulum’s margin form an acute angle in retrolateral view. Proximal part very curved in frontal view; thin distal width, abruptly narrowing distally; basal, middle, and distal width, 0.45, 0.24, 0.12, respectively. Tegulum: 2.68 long, 1.57 high in retrolateral view. Cymbium subtriangular with subequal lobes, having a well-developed rounded process on retrolateral lobe, bearing thick setae (Fig. 135, 139–141).

Tibial apophysis (Figs 136–138): single branch on prolateral leg I, with well-developed base and grouped spiniform setae distally. Male metatarsus I touches retrolaterally tibial apophysis’ setae when folded.

Coloration (Fig. 146): abdomen with long greyish brown guard-setae homogeneously distributed and dark short body setae. Abdomen venter brown.

**Figures 142–147.**
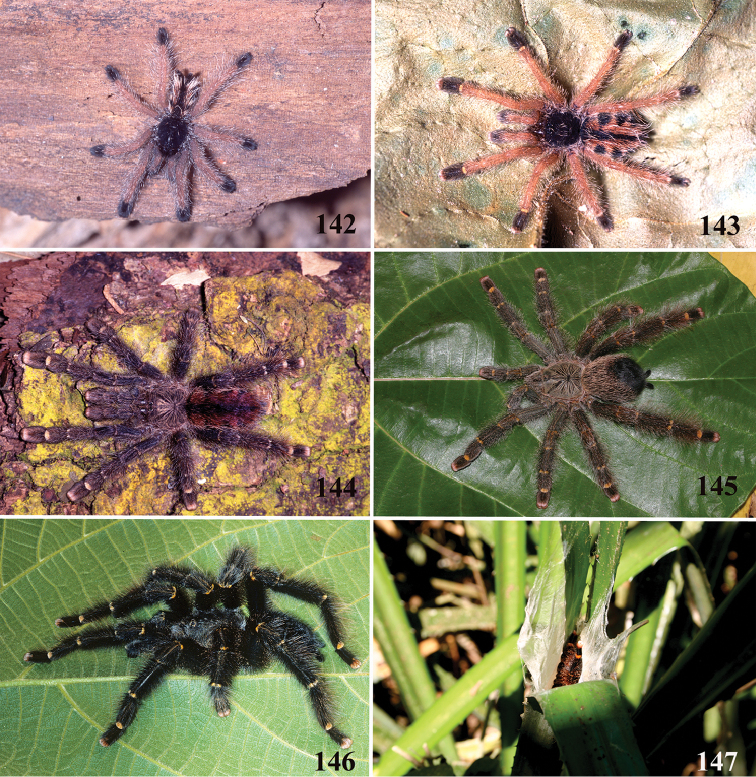
*Avicularia rufa* Schiapelli & Gerschman, 1945, habitus and retreat. **142** immature **143** juvenile **144** old juvenile **145** female **146** male **147** retreat on bromelid. Photos: **142–145, 147** R. Bertani; **146** W. Bokermann.

##### Color pattern ontogeny.

Brownish juveniles lacking metallic sheen, black tarsi contrasting with other lighter articles (Fig. 142) and abdomen dorsum reddish, with dorsal central longitudinal black stripe disconnected from transversal black stripes (Fig. 143). When mature, both males and females lose this pattern (Figs 145–146).

##### Distribution.

Brazil (states of Amazonas, Amapá, Pará, Acre, Rondônia and Mato Grosso), Ecuador, Peru and Bolivia (Fig. 50).

##### Natural history.

**Figures 148–153.**
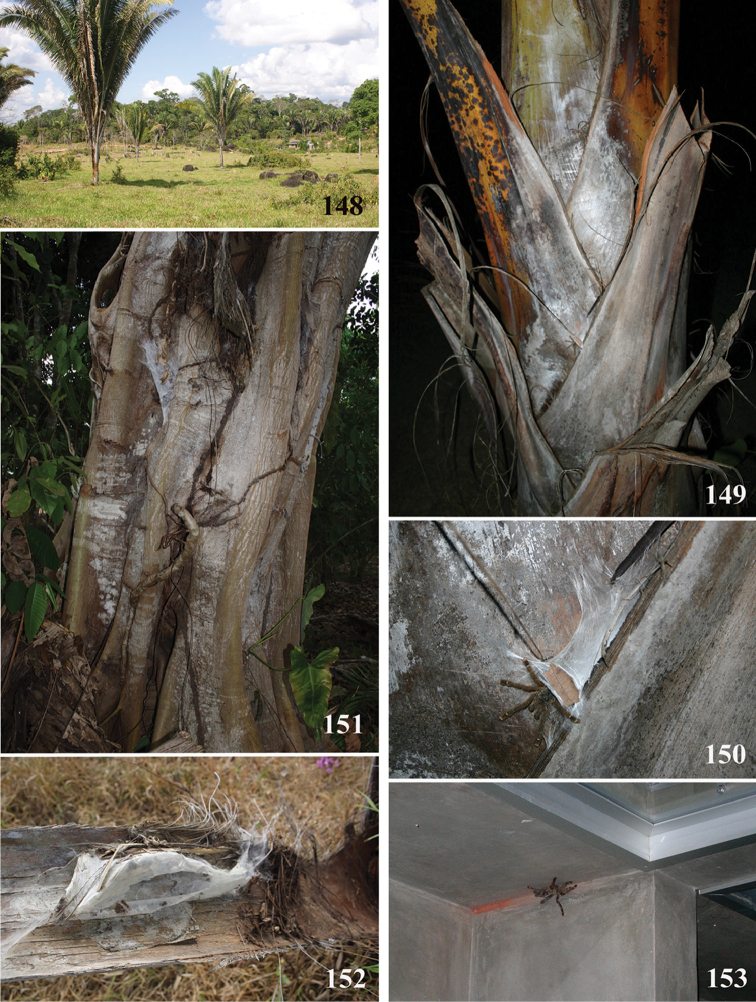
*Avicularia rufa* Schiapelli & Gerschman, 1945, habitat and retreats. **148** Babaçu palms, a common habitat of *Avicularia rufa* in state of Rondônia, Brazil **149** retreat of immature on a palm tree **150** detail of retreat **151** retreat over a tree bark **152** use of intern side of babaçu palm to build retreat **153** in human buildings. Photos: **148–152** R. Bertani; **153** C. S. Fukushima.

In northern region of Brazil, such as in state of Rondônia, specimens are commonly found in babaçu palms (Fig. 148). They build their retreats in palm axis or using underside of the palms leaves (Figs 149–150, 152). They can also build retreats over larger tree bark (Fig. 151) or inside bromeliads (Fig. 147). Specimens are usually found on human-made structures, such as on houses and other buildings (Fig. 153).

#### Avicularia purpurea

Taxon classificationAnimaliaORDOFAMILIA

Kirk, 1990

Figs 19 , 105 , 154–157 , 158–165 , 166–171 , 172 , 304

Avicularia purpurea Kirk, 1990: 15, figs 1–5 (holotype female, Ecuador, Tena [0°58'S, 77°48'W], approximately 500 m a.s.l., H. Hirschi col., 1989, BMNH 1990.5.22.1 and paratype female, Peru, H. Hirschi col., 1990, BMNH 1990.5.22.2; examined); Bullmer et al. 2006: 7, figs 16–19, 21; World Spider Catalog 2016.

##### Diagnosis.

**Figures 154–157.**
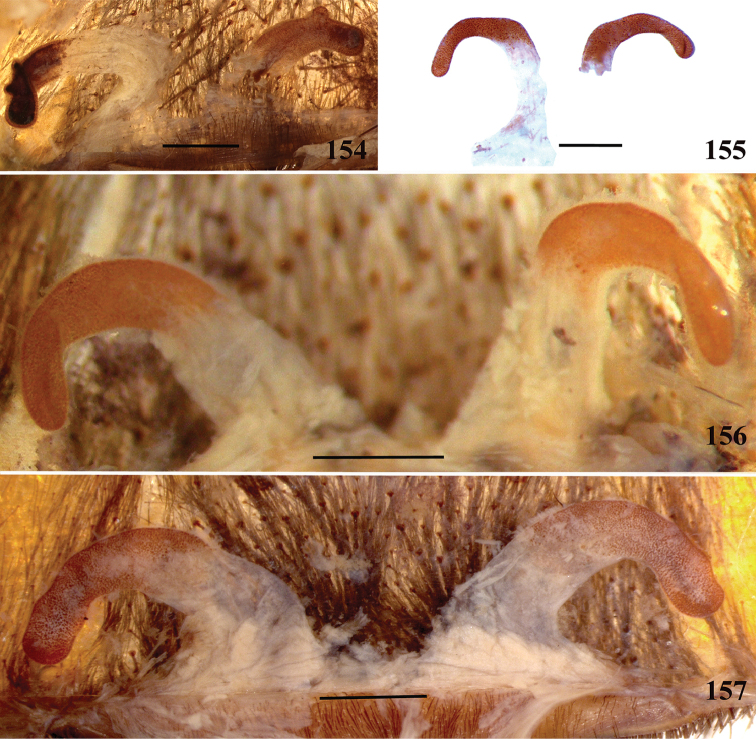
*Avicularia purpurea* Kirk, 1990, spermathecae variation. **154** Puerto Napo, department of Napo, Ecuador (CAS 11) **155** Tena, department of Napo, Ecuador (AMNH RW54) **156** holotype, Tena, department of Napo, Ecuador (BMNH 1990.5.22.1) **157** Iquitos, department of Loreto, Peru (AMNH RW33). Scale bars = 1 mm.

Females of *Avicularia purpurea* resemble those of *Avicularia merianae* sp. n. by the spermathecae with weakly-sclerotized area at least same length of well-sclerotized area (Figs 154–157). They differ from *Avicularia merianae* sp. n. by the velvety black abdomen as well as legs and palps with dark short body setae with very intense purple sheen (Figs 168, 170). Males of *Avicularia purpurea* resemble those of *Avicularia avicularia*, *Avicularia rufa. Avicularia juruensis*, *Avicularia variegata* stat. n., *Avicularia taunayi*, and *Avicularia merianae* sp. n. by tibial apophysis on leg I with well-developed base and grouped spiniform setae distally (Fig. 163). They can be distinguished from all these species except *Avicularia merianae* sp. n. by cymbium lacking well-developed process on retrolateral lobe (Fig. 162). It differs from *Avicularia merianae* sp. n. by velvety black abdomen, and legs and palps with dark short body setae with very intense purple sheen (Figs 169, 171).

##### Material examinad.

1 female, Ecuador, Napo, 20 km East of Puerto Napo, Aliñahuí (1°0'S, 77°25'O), 450 m, V. D. & B. Roth col., January 1994 (CAS 11); 1 male, Ecuador, Napo, 25 km East of Puerto Napo [1°01'S, 77°43'W], jungle Aliñahuí, 450 m, E. S. Ross col, January–February 1991 (CAS 3).

##### Additional material.

COLOMBIA: *Putumayo*: Mocoa [1°09'N, 76°39'W], Vda. Pepino, 500 m asl, 1 female, T. Sanjuan col., 30 April 1997 (ICN–Ar-1990); ECUADOR: *Napo*: Puerto Napo, 20 km East, Aliñahuí (1°0'S, 77°25'W), 450 m, 1 male, V. D. & B. Roth col., June 1994 (CAS 7); [1°01'S, 77°43'W], 1 male, V. Roth col., June 1994 (AMNH RW52); Río Napo [2°00'S, 74°20'W], 1 female, Gerhard col., 1994 (IBSP 11597); Tena, Cabañas Aliñahui (1°02'54.00"S, 77°36'05.00"W), 1 female, R. Baxter col., August 1994, in silk retreat on side of tree (AMNH RW54); PERU: *Loreto*: Iquitos, Rio Momon, Amazon Camp (3°41'16.00"S, 73°16'48"W), T. Mason col., May 1994 (AMNH RW33); 1 male, W. Lamar col., 20 August 1997 (MNRJ 06913); Río Yarapa [4°31'S, 73°22'W], 1 female, R. C. West col., 16 November 1993, in a palm tree at night (AMNH RW44); Río Nanay [3°48'S, 73°23'W], 1 female, R. C. West col., 5 November 1993 (AMNH RW45); *Ucayali* Río Ucayali [7°34'S, 74°20'W], 1 female, R. C. West col., 14 November 1993, in silk retreat on a citrus tree (AMNH RW48).

##### Female.

**Redescription.** CAS. Carapace: 15.4 long, 14.02 wide, 3.98 high. Chelicera: 5.38 long. Legs (femur, patella, tibia, metatarsus, tarsus, total): I: 11.69, 7.20, 8.54, 7.25, 5.15, 39.83. II: 10.74, 6.84, 7.99, 7.13, 4.89, 37.59. III: 9.75, 5.93, 7.33, 7.15, 4.42, 34.58. IV: 12.08, 6.47, 10.14, 9.51, 4.31, 42.51. Palp: 8.10, 5.10, 4.72, –, 5.41, 23.33. Midwidths: femora I–IV= 2.81, 3.08, 3.07, 2.90, palp= 2.38; patellae I–IV= 2.95, 2.70, 2.87, 2.98, palp= 2.57; tibiae I–IV= 2.39, 2.30, 2.56, 2.82, palp= 2.49; metatarsi I–IV= 1.92, 1.72, 1.86, 1.71; tarsi I–IV= 2.18, 2.19, 2.01, 2.13, palp= 2.36. Abdomen: 19.26 long, 13.62 wide. Spinnerets: PMS, 1.81 long, 1.19 wide, 1.07 apart; PLS, 2.28 basal, 1.09 middle, 2.74 distal; midwidths 2.38, 1.78, 1.41, respectively.

Carapace: 1.10 times longer than wide; cephalic region moderately raised, thoracic striae conspicuous.

Fovea: deep, recurve, 2.03 wide.

Eyes: eye tubercle 1.17 high, 2.21 long, 3.43 wide. Clypeus 0.12. Anterior row of eye procurve. Posterior row of eyes slightly recurve. Eye sizes and interdistances: AME 0.75, ALE 0.72, PME 0.27, PLE 0.62, AME–AME 0.6, AME–ALE 0.47, AME–PME 0.25, ALE–ALE 2.19, ALE–PME 0.63, PME–PME 2.06, PME–PLE 0.23, PLE–PLE 2.67, ALE–PLE 0.48, AME–PLE 0.67.

Maxilla length to width: 1.89. Cuspules: 100–200 spread over ventral inner heel. Labium: 2.03 long, 2.46 wide, with 108 cuspules spaced by one diameter from each other on anterior half. Labio-sternal groove shallow and flattened, sigilla not evident.

Chelicera: basal segment with 14 teeth and some small teeth on promargin. Sternum: 7.56 long, 6.27 wide. Sigilla: three pairs, elipsoidal posterior, in 45°angle, less than one diameter from margin; fusiform median, less than one diameter from margin; anterior not evident.

Legs: Formula: IV=I II III. Length leg IV to leg I: 1.07. Clavate trichobothria: distal 1/2 of tarsi I–IV. Scopula: Tarsi I–IV fully scopulate, IV divided by a wide band of setae. Metatarsi I–II fully scopulate; III on distal 2/3; IV, on distal 1/3. IV divided by wide band of setae.

Type II urticating setae: 0.53–0.58 long, 0.011–0.014 wide.

Spermathecae (Fig. 154): two completely separated, not-twisted long spermathecae, with walls lacking projections or lobes and accentuated outwards curvature medially. Midwidth as wide as its base width and weakly-sclerotized area at least same length of well-sclerotized area.

Color pattern (Figs 168): carapace brown with golden short body setae with very intense purple sheen. Carapace border with long setae the same color as dorsal carapace short body setae. Coxae, labium, sternum and maxillae brown, with the same color of ventral femora. All ventral parts, specially sternum, covered by longer setae. Legs and palps with brown short body setae with very intense purple sheen and brown long guard-setae. Leg rings on distal femora, tibiae and metatarsi whitish. Abdomen dorsum with long brown guard-setae homogeneously distributed and black short body setae, with velvety aspect (Fig. 304). Abdomen venter brown.

**Figures 158–165.**
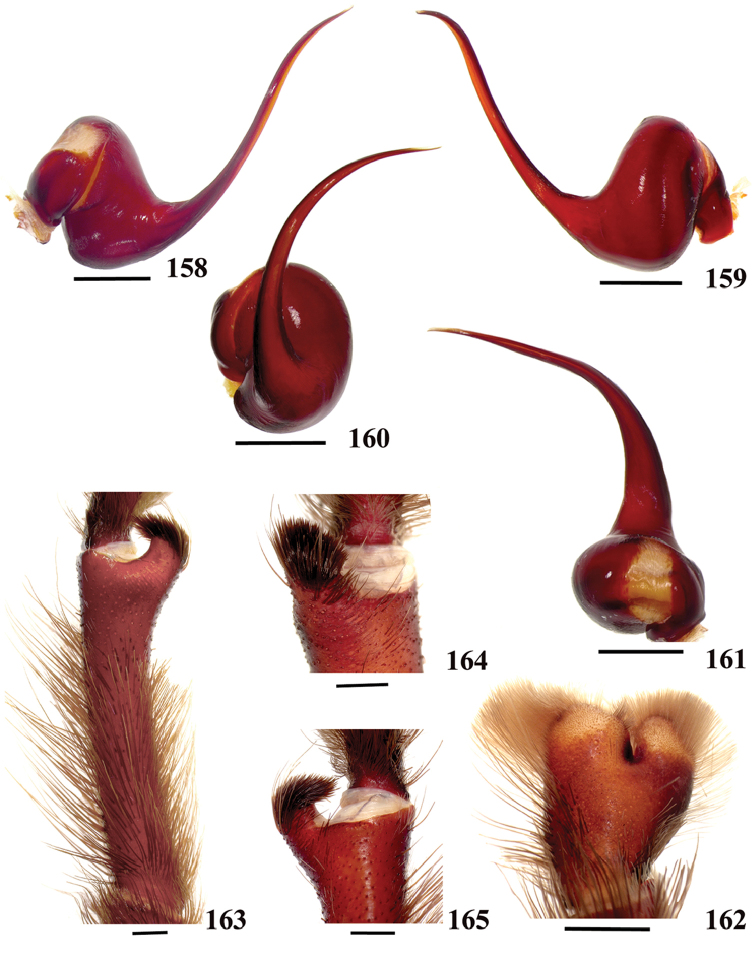
*Avicularia purpurea* Kirk, 1990, male (CAS 3; except palpal bulb in prolateral view, CAS 7). **158–161** left palpal bulb **158** prolateral **159** retrolateral **160** frontal **161** dorsal **162** left cymbium, dorsal **163–165** left tibial apophysis of leg I **163** prolateral **164** ventral **165** retrolateral. Scale bars = 1 mm.

##### Male.

**Description.**

CAS. Carapace: 11.98 long, 10.74 wide, 2.85 high. Chelicera: 3.79 long. Legs (femur, patella, tibia, metatarsus, tarsus, total): I: 12.59, 6.43, 9.70, 9.13, 5.68, 43.53. II: 11.82, 5.86, 9.35, 8.78, 5.18, 40.99. III: 10.47, 5.26, 8.36, 8.54, 4.77, 37.40. IV: 13.16, 5.27, 11.04, 11.25, 4.68, 45.40. Palp: 6.97, 4.35, 5.55, –, 2.32, 19.15. Midwidths: femora I–IV= 2.08, 2.49, 2.18, 2.20, palp= 1.71; patellae I–IV= 2.22, 2.21, 2.15, 2.28, palp= 1.68; tibiae I–IV= 1.64, 1.73, 1.86, 1.89, palp= 1.65; metatarsi I–IV= 1.35, 1.31, 1.25, 1.23; tarsi I–IV= 1.63, 1.58, 1.65, 1.53, palp= 1.69. Abdomen: 13.09 long, 7.88 wide. Spinnerets: PMS, 1.57 long, 0.79 wide, 0.29 apart; PLS, 2.00 basal, 1.12 middle, 2.78 distal; midwidths 1.42, 1.29, 0.92, respectively.

As in female, except:

Carapace: 1.12 times longer than wide; cephalic region not raised, thoracic striae inconspicuous.

Fovea: straight, 1.14 wide.

Eyes: eye tubercle 0.60 high, 1.73 long, 2.54 wide. Clypeus 0.30. Eye size and interdistances: AME 0.58, ALE 0.67, PME 0.22, PLE 0.42, AME–AME 0.48, AME–ALE 0.36, AME–PME 0.20, ALE–ALE 1.66, ALE–PME 0.74, PME–PME 1.52, PME–PLE 0.07, PLE–PLE 1.95, ALE–PLE 0.40, AME–PLE 0.43.

Maxilla: length to width: 2.07. Labium: 1.33 long, 1.92 wide, with 120 cuspules spaced by one diameter from each other on the anterior half.

Chelicera: basal segment with 11 teeth and some small teeth on promargin. Sternum: 5.78 long, 4.81 wide. Sigilla: three pairs, posterior and median rounded, large, less than one diameter from margin; anterior not evident.

Legs: Length leg IV to leg I: 1.04. Scopula: Metatarsi IV scopulate in distal 1/4. IV divided by band of setae.

Type II urticating setae: 0.78–0.83 long, 0.015–0.017 wide.

Palp (Figs 158–161): globous bulb with small subtegulum and developed prominence on tegulum. Embolus: not flattened, lacking keels, 3.5 long in retrolateral view, about 3.0 times tegulum’s length. Medial portion and tegulum’s margin form an acute angle in retrolateral view. Proximal part very curved in frontal view; thin distal width, tapering distally; basal, middle, and distal width 0.68, 0.18, 0.05, respectively. Tegulum: 1.96 long, 1.66 high in retrolateral view. Cymbium subtriangular with subequal lobes, lacking well-developed process on retrolateral lobe (Fig. 162).

Tibial apophysis (Figs 163–165): single branch on prolateral leg I, with well-developed base and grouped spiniform setae distally. Male metatarsus I touches retrolaterally tibial apophysis’ setae when folded.

**Figures 166–171.**
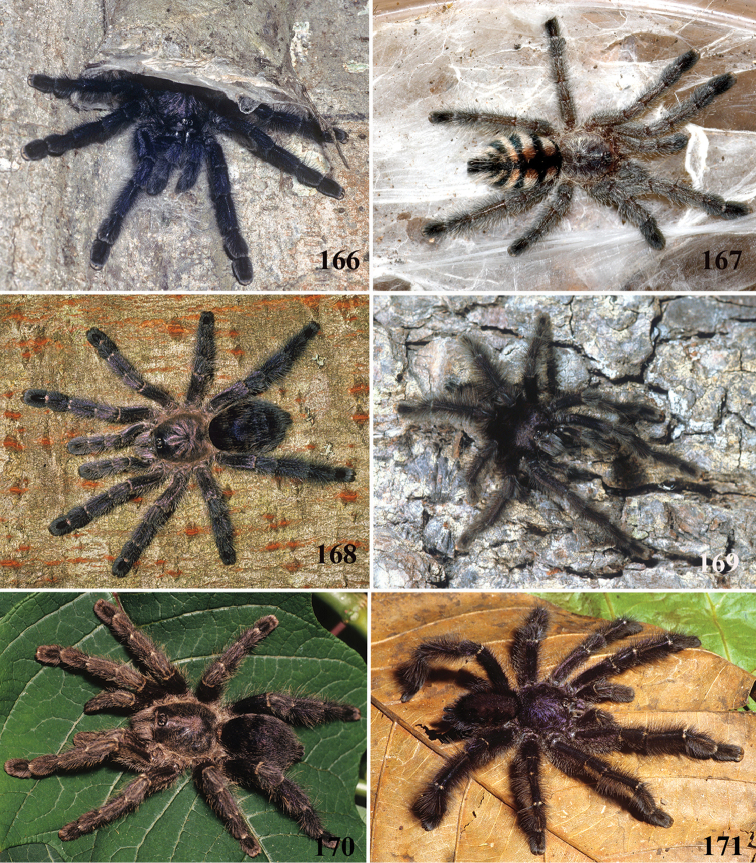
*Avicularia purpurea* Kirk, 1990, habitus and retreat. **166** silken retreat over tree bark **167** immature **168–169** northern form, from Tena, Ecuador **168** female **169** male **170–171** southern form, from Rio Momon, Peru **170** female **171** male. Photos: **166, 168, 170** R. C. West; **167** R. Bertani; **169** R. Baxter; **171** W. Lamar.

##### Color pattern ontogeny.

Juveniles present green metallic sheen, all articles with blackish color and abdomen dorsum with central longitudinal black stripe connected with all transversal black stripes of each side (Fig. 167). When mature, both males and females lose this pattern (Figs 168–171).

##### Variation.

**Figure 172.**
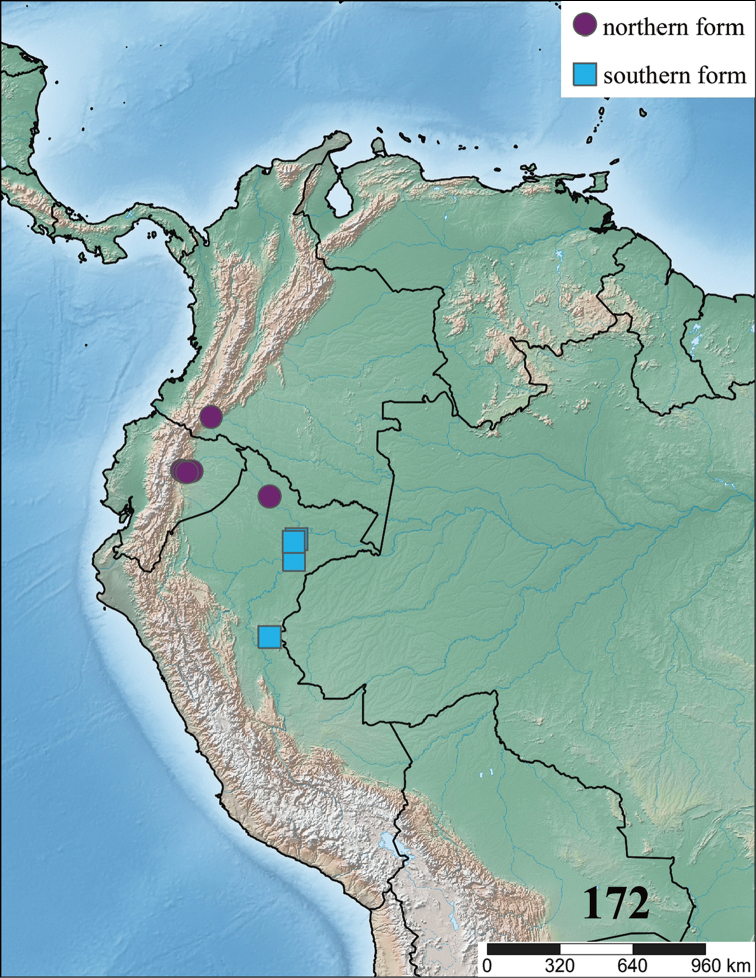
Map showing records of *Avicularia purpurea* Kirk, 1990 morphotypes.

We found two different morphotypes among the examined material (Fig. 172). The northern form is the same holotype’s morphotype (Figs 168–169). The southern form has very discrete grizzled setae in palps and legs, and less intense purple sheen in short body setae of carapace, legs, and palps (Figs 170–171). Females of the southern form have abdomen with light brown guard-setae homogeneously distributed, with a couple of reddish brown lateral stripes until penultimate or ultimate molt, distinct from the velvety black dorsal abdomen of the northern morphotype. Males from southern and northern form showed no morphological difference among them (Figs 169, 171).

##### Distribution.

Colombia, Ecuador, and Peru (Fig. 105).

##### Natural history.

Specimens were found in anthrophized areas, such as cultivated areas and cattle pastures with dispersed trees, near Tena, Ecuador (Kirk 1990). They are arboreals, making their retreats in tree holes, epiphytes, and inside houses and other human constructions (Kirk 1990). Specimens of the southern form were found in natural cavities of living palms or citrus-like trees in open forested fringes or in gallery forest areas (Rick West, pers. comm.). They use bark and organic debris to camouflage the partially exposed webbed retreat, which lies about 25 cm from soil (Rick West, pers. comm.).

#### Avicularia hirschii

Taxon classificationAnimaliaORDOFAMILIA

Bullmer et al. 2006

Figs 19 , 90 , 173–180 , 181–183 , 184–187 , 309

Avicularia hirschii Bullmer et al. 2006: 3, figs 1–10, 20, 22 (holotype male, Ecuador, Oriente, nahe Misahualli [1°02'S, 77°41'W], Bullmer leg., August 1997, SMF 57125 and paratype female, Ecuador, Oriente, nahe Misahualli [1°02'S, 77°41'W], Bullmer leg., August 1997, SMF 57126, examined); World Spider Catalog 2016.

##### Diagnosis.

Females of *Avicularia hirschii* resemble those of *Avicularia avicularia* and *Avicularia rufa* by the leg IV longer than leg I. They can be distinguished from them by twisted spermatheca (Fig. 181). Males of *Avicularia hirschii* resemble those of *Avicularia minatrix*, *Avicularia lynnae* sp. n. and *Avicularia caei* sp. n. by tibia I with discrete elevation covered by a cluster of setae in apical portion, on prolateral side (Fig. 179). They can be distinguished from males of *Avicularia lynnae* sp. n. and *Avicularia caei* sp. n. by shorter embolus, about 3.0 to 3.5 times tegulum’s width in retrolateral view (Fig. 174) and from *Avicularia minatrix* by having prominece on tegulum (Fig. 175) and cymbium with process on retrolateral lobe (Fig. 177).

**Figures 173–180.**
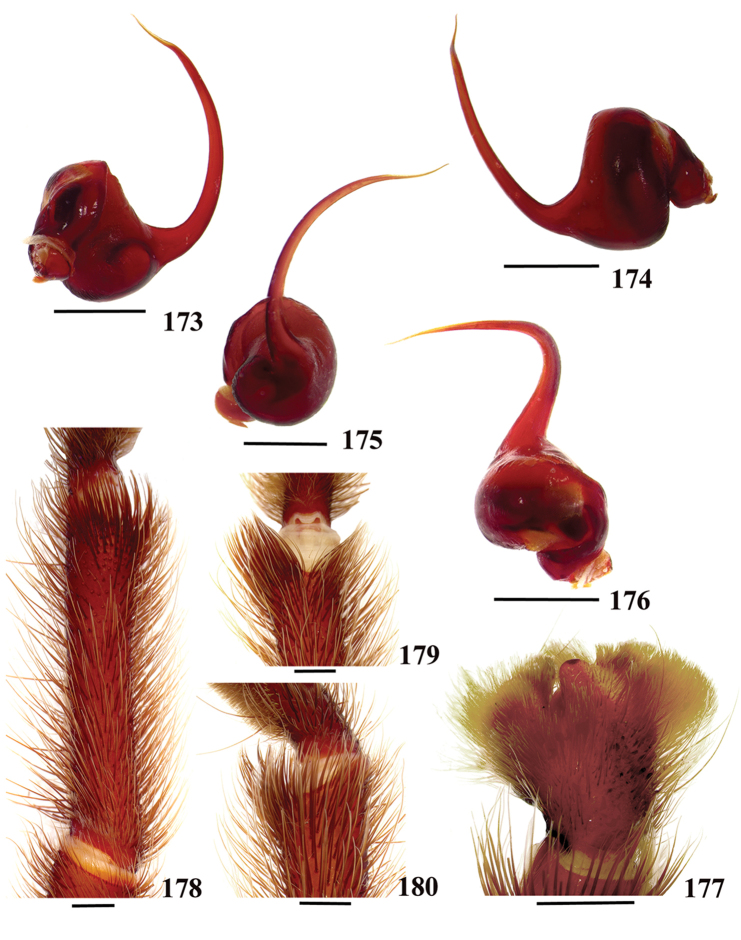
*Avicularia hirschii* Bullmer et al. 2006, male holotype (SMF 57125). **173–176** right palpal bulb (mirrored) **173** prolateral **174** retrolateral **175** frontal **176** dorsal **177** right cymbium, dorsal (mirrored) **178–180** left tibia I **178** prolateral **179** ventral **180** retrolateral. Scale bars = 1 mm.

##### Additional material.

BRAZIL: *Acre*: Senador Guiomard [10°08'S, 67°44'W], 1 female, C. Alexandre col., 12–17 July 2013 (MNRJ 06912); Estação Ecológica Rio Acre [10°59'S, 70°13'W], M. A. de Freitas col., 4–18 February 2016 (MNRJ 06911); ECUADOR: *Pastaza*: 25 km east of Puerto Napo [1°01'S, 77°43'W], selva Aliñahuí, 450 m, I–II, 1 female, 1 immature, E. S. Ross col., 1991 (CAS 1, CAS 2); PERU: *Loreto*: confluence of Rio Zumun and Rio Yaguasyacu [3°21'S, 71°58'W] (Rio Yahnasyacu [*sic*]), 2 juvenile females, J. Becker col. (MNRJ 13759); Iquitos, Rio Nanay [3°48'S, 73°23'W], 1 juvenile female, R. C. West col., 5 November 1993 (AMNH RW50); 1 juvenile female, Rio Tigre, Cristo Rey village [3°58'S, 74°16'W], W. Lamar col., 19 July 1998, in light silken retreat between broad leaves 1.5 m above ground in forest behind village (MNRJ 06910).

##### Male.

**Redescription**. SMF 57125. Carapace: 10.83 long, 9.32 wide, 2.50 high. Chelicera: 2.65 long. Legs (femur, patella, tibia, metatarsus, tarsus, total): I: 10.02, 5.63, 7.80, 7.72, 4.51, 35.68. II: 9.66, 4.99, 7.44, 6.88, 4.48, 33.45. III: 8.44, 4.27, 6.17, 6.94, 3.66, 29.48. IV: 10.20, 4.64, 8.79, 9.30, 3.68, 36.61. Palp: 6.08, 3.63, 4.57, –, 1.78, 16.06. Midwidths: femora I–IV= 1.94, 1.77, 2.14, 2.15, palp= 1.45; patellae I–IV= 2.9, 1.87, 1.97, 1.76, palp= 1.56; tibiae I–IV= 1.53, 1.66, 1.53, 1.60, palp= 1.67; metatarsi I–IV= 1.02, 1.22, 1.04, 1.17; tarsi I–IV= 1.24, 1.47, 1.38, 1.50, palp= 1.57. Abdomen: 10.95 long, 6.04 wide. Spinnerets: PMS, 1.02 long, 0.46 wide, 0.1 apart; PLS, 1.82 basal, 0.93 middle, 2.21 distal; midwidths 1.04, 0.82, 0.60, respectively.

Carapace: 1.16 times longer than wide; cephalic region not raised.

Fovea: deep, straight, 1.29 wide.

Eyes: eye tubercle 0.66 high, 1.59 long, 2.30 wide. Clypeus absent. Anterior row of eyes procurve, posterior slightly recurve. Eye size and interdistances: AME 0.69, ALE 0.72, PME 0.30, PLE 0.44, AME–AME 0.34, AME–ALE 0.16, AME–PME 0.10, ALE–ALE 1.45, ALE–PME 0.55, PME–PME 1.50, PME–PLE 0.03, PLE–PLE 1.75, ALE–PLE 0.23, AME–PLE 0.43.

Maxilla: length to width: 2.53. Cuspules: 112 spread over ventral inner heel. Labium: 1.00 long, 1.64 wide, with 54 cuspules spaced by one diameter from each other on anterior third. Labium sternal groove shallow, flat, sigilla not evident.

Chelicera: basal segment with 9 teeth and some small teeth on promargin. Sternum: 5.01 long, 3.93 wide. Sigilla: only posterior pair evident, rounded, less than one diameter from margin.

Legs: Formula: IV=I II III. Length leg IV to leg I: 1.03. Clavate trichobothria: 2/3 distal tarsi I–IV. Scopula: Tarsi I–IV fully scopulate. IV with some sparse setae. Metatarsi I–II fully scopulate; III 2/3; IV 1/2 distal scopulate. IV divided by a wide row of setae.

Type II urticating setae: 0.10–0.11 long (according to original description, since holotype abdomen is bald).

Palp (Figs 173–176): globous bulb with small subtegulum and developed prominence on tegulum. Embolus: not flattened, lacking keels, 2.7 long in retrolateral view, about 3.5 times tegulum’s length. Medial portion and tegulum’s margin form an acute angle in retrolateral view. Proximal part very curved in frontal view; thin distal width, tapering distally; basal, middle, and distal width of 0.35, 0.11, 0.03, respectively. Tegulum: 1.51 long, 0.93 wide in retrolateral view. Cymbium subtriangular with subequal lobes. Cymbium with well-developed rounded process on retrolateral lobe, bearing thin setae (Fig. 177).

Tibia I with discrete elevation covered by cluster of setae in apical portion, on prolateral side (Figs 178–180, 309).

Color pattern: carapace brown with golden short body setae with pink sheen. Carapace border with long setae the same color as dorsal carapace short body setae. Coxae, labium, sternum and maxillae light brown, same color of ventral femora. Legs and palps with brown short body setae with golden sheen and reddish brown long guard-setae. Posterior legs darker, blackish. Tarsi III and IV with reddish central well-developed tufts (not detected, but informed on original description). Tarsi with “U” shaped orange stripe. Leg rings on distal femora, tibiae and metatarsi whitish. Abdomen dorsum with long reddish guard-setae and dark short body setae. Ventral abdomen light brown.

**Figures 181–183.**
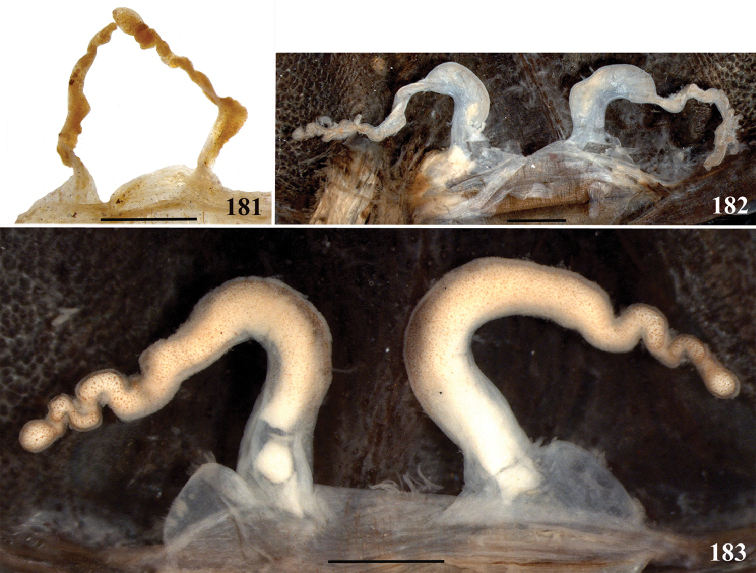
*Avicularia hirschii* Bullmer et al. 2006, spermathecae variation. **181** paratype, Oriente, department of Napo, Ecuador (SMF 57126) **182** Estação Ecológica Rio Acre, Assis Brasil, state of Acre, Brazil (MNRJ 06911) **183** Senador Guiomard, state of Acre, Brazil (MNRJ 06912). Scale bars = 1 mm.

**Figures 184–187.**
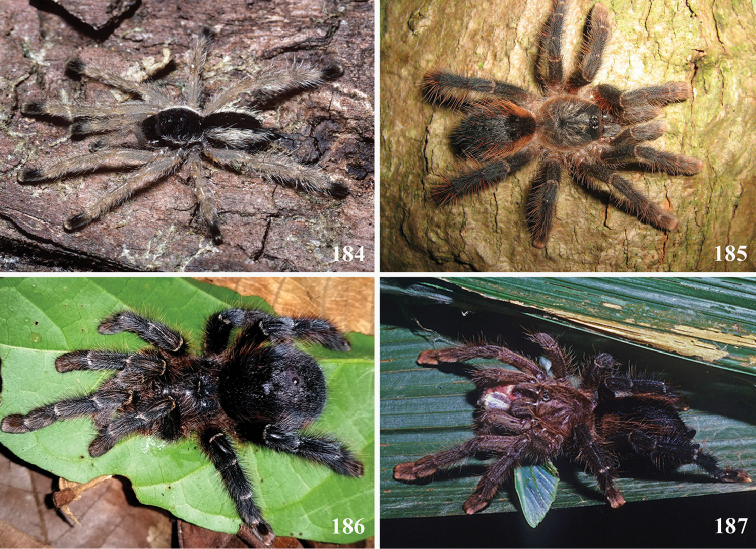
*Avicularia hirschii* Bullmer et al. 2006, habitus. **184** immature, Tiguino, department of Pastaza, Ecuador **185** juvenile, Senador Guiomard, state of Acre, Brazil **186** female, Estação Ecológica Rio Acre, Assis Brasil, state of Acre, Brazil **187** female feeding on an insect, Rio Momón, department of Loreto, Peru. Photos: **184** W. Lamar; **185** S. Albuquerque, **186** M. A. de Freitas; **187** R. C. West.

##### Female.

**Redescription.** SMF 57126. Carapace: 11.92 long, 11.37 wide, 3.46 high. Chelicera: 3.84 long. Legs (femur, patella, tibia, metatarsus, tarsus and total): I: 9.69, 5.45, 6.78, 6.43, 4.81, 33.16. II: 9.11, 5.39, 6.69, 6.05, 4.38, 31.62. III: 8.23, 4.90, 5.49, 6.53, 3.94, 29.09. IV: 10.19, 5.64, 8.73, 9.22, 4.57, 38.35. Palp: 6.99, 4.14, 4.40, –, 5.08, 20.61. Midwidths: femora I–IV= 2.00, 1.97, 2.49, 2.21, palp= 1.93; patellae I–IV= 2.10, 1.92, 2.27, 2.47, palp= 1.74; tibiae I–IV= 1.90, 1.83, 2.09, 2.27, palp= 1.73; metatarsi I–IV= 1.60, 1.39, 1.58, 1.82; tarsi I–IV= 1.91, 1.71, 1.81, 1.96, palp= 1.79. Abdomen: 12.61 long, 10.24 wide. Spinnerets: PMS, 1.38 long, 1.07 wide, 0.07 apart; PLS damaged.

As in male, except:

Carapace: 1.05 times longer than wide; thoracic striae conspicuous.

Fovae: recurve, 1.46 wide.

Eyes: eye tubercle 0.94 high, 2.02 long, 2.88 wide. Clypeus 0.08. Eye size and interdistances: AME 0.65, ALE 0.67, PME 0.22, PLE 0 .68, AME–AME 0.41, AME–ALE 0.40, AME–PME 0.14, ALE–ALE 1.74, ALE–PME 0.75, PME–PME 1.57, PME–PLE 0.03, PLE–PLE 1.99, ALE–PLE 0.24, AME–PLE 0.50.

Maxilla: length to width: 2.03. Cuspules: 146. Labium: 1.50 long, 2.40 wide, with 64 cuspules spaced by one diameter. Labio-sternal groove with two separate, large sigilla.

Chelicera: basal segmente with 12 teeth and some small teeth on promargin. Sternum: 6.66 long, 4.30 wide. Sigilla: only posterior pair evident, elipsoidal, less than one diameter from margin.

Legs: Formula: IV I II III. Length leg IV to leg I: 1.16. Scopula: Tarsi IV divided by some setae on base; metatarsi IV scopulate on distal 1/4.

Type II urticating setae: 0.38–0.50 long, 0.011–0.014 wide

Spermathecae (Fig. 181): two completely separated, twisted long spermatheca, with walls lacking projections or lobes and accentuated outwards curvature medially. Midwidth as wide as its base width and weakly-sclerotized area shorter than half the length of well-sclerotized area.

Color pattern: dorsal abdomen with vivid reddish guard-setae grouped on lateral area and black short body setae (Fig. 186).

##### Color pattern ontogeny.

Brownish juveniles lacking metallic sheen, black tarsi contrasting with other lighter articles and black central longitudinal stripe on abdomen dorsum (Fig. 184).

##### Distribution.

Brazil (state of Acre), Ecuador and Peru (Fig. 90).

##### Natural history.

Types were collected in trees in an old pasture, in different trees about 5 m from each other, surrounding by grass (Bullmer et al. 2006). Immature male was in a tree in a retreat about 1.6 m above the ground and female was also in a tree; both retreats were made by web, soil and moss, 60 cm from ground (Bullmer et al. 2006). A found eggsac hatched in captivity and contained 38 specimens (Bullmer et al. 2006). In the state of Acre, Brazil, specimens were found in silken retreats constructed inside tree trunks (C. Alexandre, pers. comm.; M. A. Freitas and A. Zanotti, pers. comm.).

#### Avicularia merianae sp. n.

Taxon classificationAnimaliaORDOFAMILIA

http://zoobank.org/C2A7B023-1FA4-4C74-B205-0549E650C4DA

Figs 9–10 , 19 , 105 , 188–195 , 196–198 , 199–200

##### Diagnosis.

Females of *Avicularia merianae* sp. n. resemble those of *Avicularia purpurea* by the spermathecae with weakly-sclerotized area at least same length of well-sclerotized area (Fig. 196). They differ from *Avicularia purpurea* by dark brown dorsal abdomen, and by lacking dark short body setae with very intense purple sheen on legs and palps. Males of *Avicularia merianae* sp. n. resemble those of *Avicularia avicularia*, *Avicularia rufa. Avicularia juruensis*, *Avicularia variegata* stat. n., *Avicularia taunayi* and *Avicularia purpurea* by tibial apophysis on leg I with well-developed base and grouped spiniform setae distally (Fig. 194). They can be distinguished from all these species except *Avicularia purpurea* by cymbium lacking well-developed process on retrolateral lobe (Fig. 192). It differs from *Avicularia purpurea* by lacking velvety black abdomen and dark short body setae with very intense purple sheen on legs and palps.

##### Etymology.

It was named after Maria Sybilla Merian, the German-born naturalist who drew the famous engraving of a specimen of *Avicularia* eating a bird, in recognition to her importance for Natural Sciences. This extraordinary woman was one of the pioneering female scientists and a remarkable artist. This name is considered feminine in gender.

**Figures 188–195.**
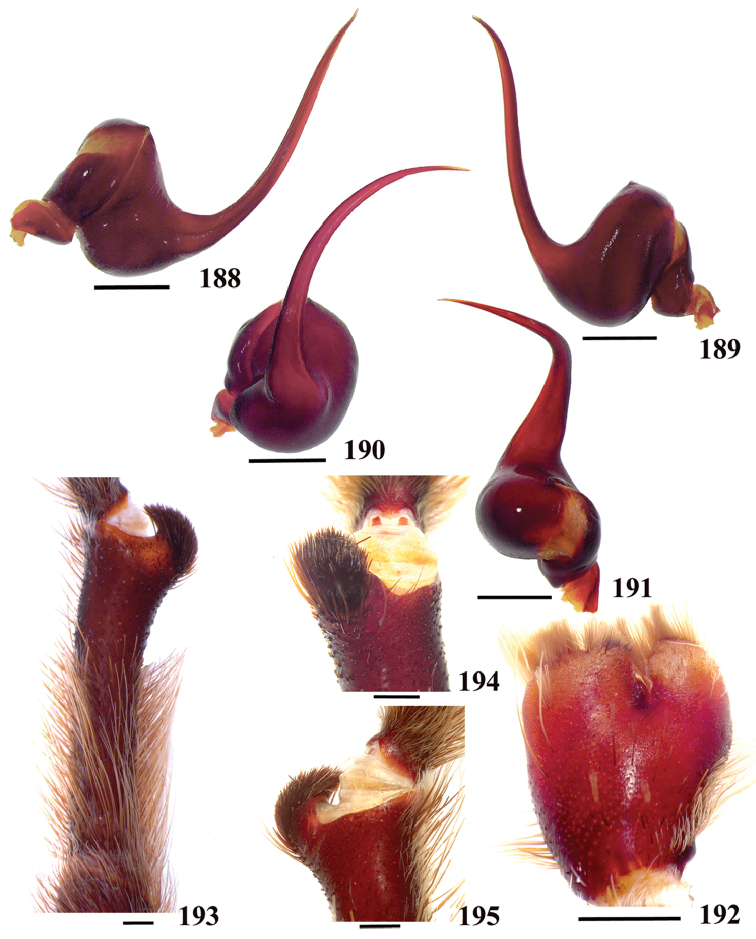
*Avicularia merianae* sp. n., male holotype (AMNH Pe102). **188–191** left palpal bulb **188** prolateral **189** retrolateral **190** frontal **191** dorsal **192** left cymbium, dorsal **193–195** left tibial apophysis of leg I **193** prolateral **194** ventral **195** retrolateral. Scale bars = 1 mm.

##### Material examined.

Holotype male, 1 female and 3 immatures paratypes, Peru, department of San Martín, Hara, 20 miles SE from Moyobamba [6°01'S, 76°58'W], F. Woytkowski col., 1–30 June 1947 (AMNH Pe102).

##### Additional material.

PERU: *San Martín*: Tarapoto [6°07'S, 75°57'W], 830 m a.s.l., 7 females, 1 juvenile female, 2 immature males, 15 immatures, 1 spiderling, F. Woytkowski col., 10–18 February 1947 (AMNH Pe106, Pe105, Pe108); Ekin, west of Tarapoto, 1 young female, under fallen tree, 890 m a.s.l., F. Woytkowski col., 9–21 March 1947 (AMNH Pe104); 1 immature male, under fallen bark, same collector and date (AMNH Pe107); Moyobamba [6°01'S, 76°58'W], Hara, 20 miles southeast of Moyobamba, 4 males, 12 females, 3 juvenile females, 22 immatures, 1 spiderling, F. Woytkowski, 1–30 June 1947 (AMNH Pe98, Pe103, Pe100, Pe53, Pe99, Pe101, AMNH tube, AMNH tube); Rio Huallaga [7°47'S, 76°11'W], 1 immature male, J. C. Pallister col., 15 December 1946 (AMNH Pe112).

**Figures 196–198.**
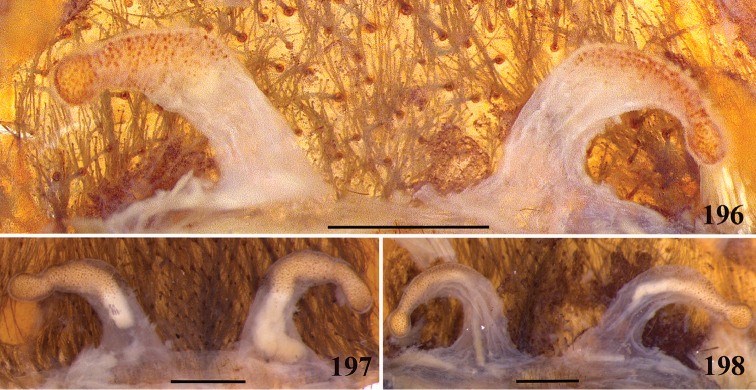
*Avicularia merianae* sp. n., spermathecae variation. **196** paratype, Moyobamba, department of San Martín, Peru (AMNH Pe102) **197** Tarapoto, department of San Martín, Peru (AMNH Pe106) **198** Moyobamba, department of San Martín, Peru (AMNH Pe100). Scale bars = 1 mm.

##### Male.

**Description.**

AMNH Pe102. Carapace: 13.49 long, 12.80 wide, 3.48 high. Chelicera: 3.46 long. Legs (femur, patella, tibia, metatarsus, tarsus and total): I: 13.41, 7.59, 10.39, 9.96, 6.21, 47.56. II: 12.75, 7.14, 9.89, 9.44, 5.48, 44.70. III: 11.01, 5.86, 8.57, 9.13, 5.46, 40.03. IV: 14.15, 7.03, 12.05, 11.85, 5.54, 50.62. Palp: 7.98, 4.78, 5.87, –, 2.45, 21.08. Midwidths: femora I–IV=2.45, 2.51, 2.39, 2.55, palp= 1.85; patellae I–IV= 2.58, 2.90, 2.51, 2.89, palp= 1.99; tibiae I–IV= 2.09, 2.05, 2.06, 2.16, palp= 1.69; metatarsi I–IV= 1.68, 1.57, 1.53, 1.33; tarsi I–IV= 1.56, 1.70, 1.67, 1.51, palp= 1.88. Abdomen: 16.35 long, 10.45 wide. Spinnerets: PMS, 2.15 long, 0.94 wide, 0.44 apart; PLS, 2.38 basal, 1.88 middle, 3.00 distal; midwidths 1.30, 1.26, 1.17, respectively.

Carapace: 1.06 times longer than wide; cephalic region not raised, thoracic striae inconspicuous.

Fovea: deep, recurve, 1.78 wide.

Eyes: eye tubercle 1.16 high, 1.97 long, 2.61 wide. Clypeus absent. Anterior eye row procurve. Posterior eye row slightly recurve. Eye size and interdistances: AME 0.72, ALE 0.72, PME 0.21, PLE 0.54, AME–AME 0.32, AME–ALE 0.37, AME–PME 0.12, ALE–ALE 1.78, ALE–PME 0.90, PME–PME 1.71, PME–PLE 0.07, PLE–PLE 2.07, ALE–PLE 0.39, AME–PLE 0.40.

Maxilla: length to width: 1.96. Cuspules: 236 spread over ventral inner heel. Labium: 1.62 long, 1.89 wide, with 101 cuspules spaced by one diameter from each other, on anterior half. Labio-sternal groove shallow, flat, with no evident sigilla.

Chelicera: basal segment with 12 teeth and some small teeth on promargin. Sternum: 7.04 long, 6.04 wide. Sigilla: anterior pair not evident, middle fusiform, posterior ellipsoidal, in a 45°angle, both less than one diameter from margin.

Legs: Formula: IV=I II III. Length leg IV to leg I: 1.06. Clavate trichobothria: distal 2/3 of tarsi I–IV. Scopula: Tarsi I–IV fully scopulate. Metatarsi I fully scopulate, II–III 2/3; IV 1/4 distal scopulate. IV divided by a row of setae.

Type II urticating setae: 0.82–0.92 long, 0.016–0.019 wide.

Palp (Figs 188–191): globous bulb with small subtegulum and developed prominence on tegulum. Embolus: not flattened, lacking keels, 3.83 long in retrolateral view, about 3.0 times tegulum’s length. Medial portion and tegulum’s margin form an acute angle in retrolateral view. Proximal part very curved in frontal view; thin distal width, tapering distally; basal, middle, and distal width 0.81, 0.21, 0.03, respectively. Tegulum: 2.07 long, 1.20 high in retrolateral view. Cymbium subtriangular with subequal lobes, lacking process on retrolateral lobe (Fig. 192).

Tibial apophysis (Figs 193–195): a single branch on prolateral leg I, with well-developed base and grouped spiniform setae distally. Male metatarsus I touches retrolaterally tibial apophysis’ setae when folded.

Color pattern: carapace brown with golden short body setae with pink sheen. Carapace border with long setae the same color as dorsal carapace short body setae. Coxae, labium, sternum and maxillae light brown, same color of ventral femora. Legs and palps with golden brown short body setae with pink sheen and brown long guard-setae. Leg rings on distal femora, tibiae and metatarsi whitish. Abdomen with orange brown guard-setae homogeneously distributed and dark brown body setae. Abdomen venter brown.

**Figures 199–200.**
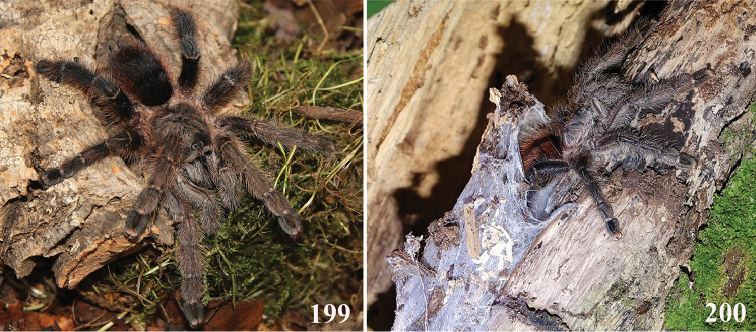
*Avicularia merianae* sp. n., habitus and retreat. **199** female **200** silken retreat over tree bark. Photos: H.-W. Auer.

##### Female.

**Description.**

AMNH Pe102. Carapace: 14.57 long, 13.15 wide, 3.83 high. Chelicera: 6.14 long. Legs (femur, patella, tibia, metatarsus, tarsus, total): I: 10.75, 6.90, 7.99, 6.72, 5.15, 37.51. II: 9.99, 6.52, 7.31, 6.41, 4.67, 34.90. III: 9.04, 5.59, 6.88, 6.54, 4.88, 32.93. IV: 11.37, 6.50, 9.66, 8.82, 4.38, 40.73. Palp: 7.95, 4.77, 4.70, –, 5.35, 22.77. Midwidths: femora I–IV= 2.68, 3.02, 3.05, 2.88, palp= 2.09; patellae I–IV= 2.82, 2.79, 2.77, 2.90, palp= 2.34; tibiae I–IV= 2.29, 2.35, 2.59, 3.11, palp= 2.29; metatarsi I–IV= 2.07, 2.05, 1.90, 2.05; tarsi I–IV= 2.24, 2.27, 2.09, 2.12, palp= 2.37. Abdomen: 17.49 long, 11.96 wide. Spinnerets: PMS, 2.08 long, 1.01 wide, 0.16 apart; PLS, 2.37 basal, 1.11 middle, 2.65 distal; midwidths 1.78, 1.49, 1.21, respectively.

As in male, except:

Carapace: 1.11 times longer than wide.

Fovea: slightly recurve, 1.77 wide.

Eyes: eye tubercle 0.70 high, 2.07 long, 2.91 wide. Clypeus 0.30. Eye size and interdistances: AME 0.72, ALE 0.70, PME 0.28, PLE 0.55, AME–AME 0.50, AME–ALE 0.47, AME–PME 0.19, ALE–ALE 2.03, ALE–PME 0.71, PME–PME 1.64, PME–PLE 0.16, PLE–PLE 2.11, ALE–PLE 0.49, AME–PLE 0.52.

Maxilla: length to width: 2.02. Labium: 1.77 long, 2.41 wide, with 96 cuspules spaced by one diameter from each other, on anterior half.

Chelicera: basal segment with 9 teeth and some small teeth on promargin. Sternum: 7.17 long, 5.92 wide.

Legs: Length leg IV to leg I: 1.09 Clavate trichobothria: on distal 1/2 tarsi I–IV. Scopula: Tarsus IV with sparse setae. Metatarsi II fully scopulate; III 1/2, IV 1/3 distal scopulate.

Type II urticating setae: 0.49–0.60 long, 0.014–0.017 wide.

Spermathecae (Fig. 196): two completely separated, not-twisted long spermathecae, with walls lacking projections or lobes and accentuaded outwards curvature medially. Midwidth expanded, about 1.5 times its basal and apical portion widths and weakly-sclerotized area at least same length of well-sclerotized area.

Color pattern (Fig. 199): as in male.

##### Color pattern ontogeny.

Brownish juveniles lacking metallic sheen, black tarsi contrasting with other lighter articles and abdomen dorsum reddish, with dorsal central longitudinal black stripe connected only with anterior pair of transversal black stripes. When mature, both males and females lose this pattern.

##### Distribution.

Peru, department of San Martín (Fig. 105).

##### Natural history.

Unknown.

#### Avicularia lynnae sp. n.

Taxon classificationAnimaliaORDOFAMILIA

http://zoobank.org/4087E14F-2413-4DF6-84CE-F7D0C7B710E5

Figs 19 , 90 , 201–208 , 209

##### Diagnosis.

Males of *Avicularia lynnae* sp. n. resemble those of *Avicularia minatrix*, *Avicularia hirschii* and *Avicularia caei* sp. n. by tibia I with discrete elevation covered by a cluster of setae in apical portion, on prolateral side (Fig. 207). They can be distinguished from all species except *Avicularia caei* sp. n. by very long embolus, more than 4 times tegulum’s width in retrolateral view (Fig. 202). Males of *Avicularia lynnae* sp. n. differ from male *Avicularia caei* sp. n. by having developed prominence on tegulum (Fig. 203) and by abdomen dorsum with single central longitudinal dark stripe (Fig. 209). Female unknown.

**Figures 201–208.**
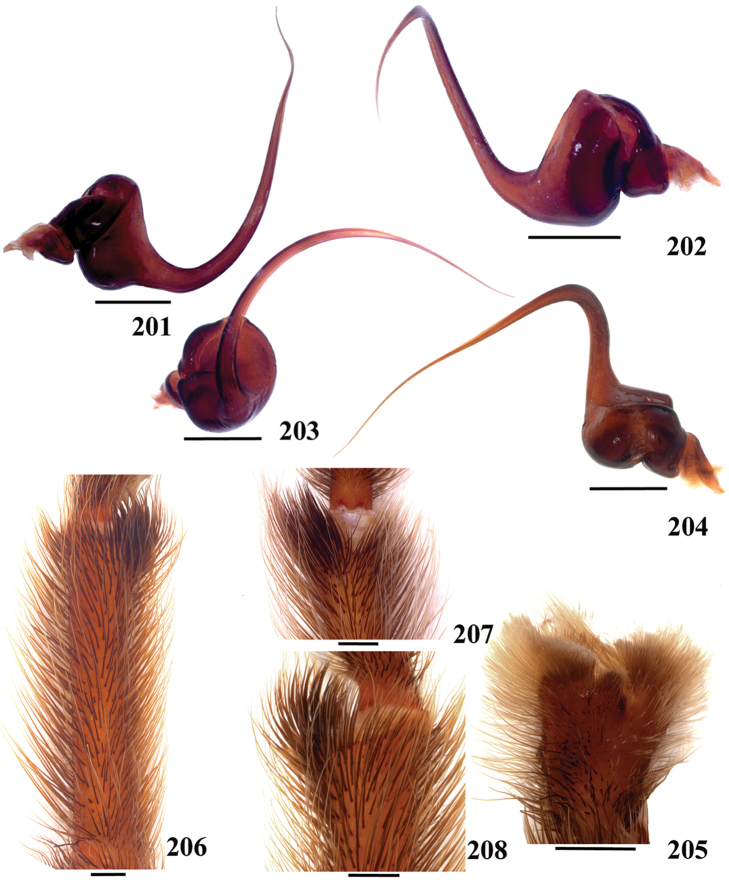
*Avicularia lynnae* sp. n., male holotype (AMNH RW49). **201–204** left palpal bulb **201** prolateral **202** retrolateral **203** frontal **204** dorsal **205** left cymbium, dorsal **206–208** left tibia I **206** prolateral **207** ventral **208** retrolateral. Scale bars = 1 mm.

##### Etymology.

It was named after Lynn West, wife of mygalomorph expert Rick West. This name is considered feminine in gender.

##### Material examined.

Holotype male, Peru, Loreto, Rio Tigre, Cristo Rey village [3°58'S, 74°16'W] near Iquitos, R. C. West col., 21 November 1993, crossing trail by day (AMNH RW49); paratype male, Peru, Loreto, Brillo Nuevo [3°09'S, 71°46'W] (Brillo Neuvo [*sic*]), Rio Yaguasyacu, B. Lamar col. (AMNH–RCW).

##### Additional material.

ECUADOR: *Pastaza*: Tigüino [1°10'S, 76°57'W], 1 male, B. Lamar col., September 1990, found in a bird capture net (AMNH–RCW); PERU: Marañón (Marauon [*sic*] [river or province?]), 1 male, Bristol, October 1927 (AMNH Pe96); *Madre de Dios*: Zona Reservada Pakitza [11°56'S, 71°17'W], 356 m asl, 1 male, Igidio & D. Silva col., 13 August 1992 (MUSM-ENTO 500685).

**Figure 209.**
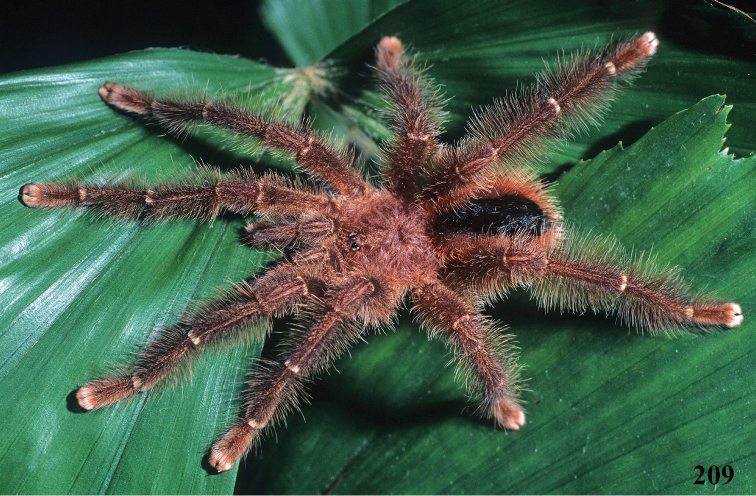
*Avicularia lynnae* sp. n., habitus, male from department of Loreto, Peru. Photo: R. C. West.

##### Male.

**Description.** AMNH RW49. Carapace: 10.87 long, 10.25 wide, 2.40 high. Chelicera: 3.07 long. Legs (femur, patella, tibia, metatarsus, tarsus, total): I: 11.9, 5.4, 9.2, 9.3, 5.0, 40.8. II: 11.1, 5.2, 8.9, 8.6, 4.7, 38.5. III: 10.0, 4.6, 8.0, 8.6, 4.6, 35.8. IV: 12.2, 4.9, 10.6, 12.6, 4.0, 44.3; Palp: 6.8, 3.7, 5.5, –, 2.1, 18.1; Midwidths: femora I–IV= 1.18, 1.7, 2.14, 1.9, palp= 1.6; patellae I–IV= 1.9, 2.2, 2.0, 2.1, palp= 1.8; tibiae I–IV= 1.7, 1.6, 1.4, 1.5, palp= 1.4; metatarsi I–IV= 1.1, 1.2, 1.1, 1.2; tarsi I–IV= 1.2, 1.3, 1.4, 1.2, palp= 1.3. Abdomen: 10.77 long, 8.49 wide. Spinnerets: PMS, 1.05 long, 0.46 wide, 0.13 apart; PLS, 1.50 basal, 0.85 middle, 2.17 distal; midwidths 0.96, 0.76, 0.68, respectively.

Carapace: 1.06 times longer than wide; cephalic region not raised, thoracic striae inconspicuous.

Fovea: shallow, straight, 0.64 wide.

Eyes: eye tubercle 0.91 high, 1.80 long, 2.41 wide. Clypeus absent. Anterior row of eyes slightly procurve, posterior slightly recurve. Eye size and interdistances: AME 0.62, ALE 0.61, PME 0.19, PLE 0.55, AME–AME 0.41, AME–ALE 0.27, AME–PME 0.14, ALE–ALE 1.54, ALE–PME 0.39, PME–PME 1.50, PME–PLE 0.04, PLE–PLE 1.96, ALE–PLE 0.19, AME–PLE 0.34.

Maxilla: length to width: 2.57. Cuspules: about 85 spread over ventral inner heel. Labium: 1.08 long, 1.72 wide, with 54 cuspules spaced by more than one diameter from each other, on anterior half. Labio-sternal groove shallow, flat, two slightly separate, large sigilla.

Chelicera: basal segment with 12 teeth and some small teeth on promargin. Sternum: 6.0 long, 3.91 wide. Sigilla: three pairs, anterior rounded, middle fusiform, posterior rounded, set at 45°angle, all close to margin.

Legs: Formula: IV=I II III. Length leg IV to leg I: 1.09. Clavate trichobothria: distal 2/3 tarsi I–IV. Scopula: Tarsi I–IV fully scopulate. Metatarsi I–II fully scopulate; III 1/2, IV 1/5 distal scopulate. IV divided by a bald area.

Type II urticating setae: 0.73–0.87 long, 0.013–0.019 wide.

Palp (Figs 201–204): globous bulb with small subtegulum and developed prominence on tegulum. Embolus: not flattened, lacking keels, 3.91 long in retrolateral view, about 4.5 times tegulum’s length. Medial portion and tegulum’s margin form an acute angle in retrolateral view. Proximal part very curved in frontal view; thin distal width, tapering distally; basal, middle and distal width of 0.26, 0.18, 0.03, respectively. Tegulum: 1.51 long, 0.89 high in retrolateral view. Cymbium subtriangular with subequal lobes, with well-developed process bearing thin setae on retrolateral lobe (Fig. 205).

Tibial I with a discrete elevation covered by a cluster of setae in apical portion, on prolateral side (Figs 206–208).

Color pattern (Fig. 209): carapace brown with golden short body setae and thick dark longer setae spread over the carapace. Carapace border long setae the same color as dorsal carapace short body setae. Coxae, labium, sternum and maxillae light brown, same color as ventral femora. Legs and palps with brown short body setae and orange brown long guard-setae. Leg rings on distal femora, tibiae and metatarsi whitish. Abdomen dorsum with long guard-setae homogeneously distributed, lateral orange short body setae and black short body setae forming a central longitudinal stripe. Ventral abdomen light brown.

##### Color pattern ontogeny.

Immatures are unknown.

##### Distribution.

Ecuador and Peru (Fig. 90).

##### Natural history.

Specimens were found in a silken retreat in a curled living leaf (W. Lamar, pers. comm. to R. C. West).

Female unknown.

##### Remarks.

Aviculariinae diversity in Ecuador, Peru and Colombia is poorly known, and certainly underestimated; specimens are rare in arachnological collections. Thus, the identity of some specimens collected in these countries should be analyzed carefully. *Avicularia lynnae* sp. n. specimens were collected in Peru and Ecuador, and its female is unknown. The species is sympatric with *Avicularia hirschii* and resembles it by having tibia I with discrete elevation covered by a cluster of setae in apical portion on prolateral side, and by having cymbium with thin setae covering the process on retrolateral lobe. The difference lies in embolus length, much greater in *Avicularia lynnae* sp. n. (Fig. 204) than in *Avicularia hirschii* (Fig. 176). Pairing female and immature to males of each one of these sympatric species is a problem. Paratype female of *Avicularia hirschii* has very long twisted spermatheca, which is morphologically more compatible with the very long embolus of males of *Avicularia lynnae* sp. n. Immatures of *Avicularia hirschii* were described as having single dorsal black stripe on abdomen (Fig. 184). However, this immature pattern could fit to adults of either *Avicularia hirschii* and *Avicularia lynnae* sp. n. Thus, it is necessary to further collect specimens to solve this query.

#### Avicularia caei

Taxon classificationAnimaliaORDOFAMILIA

sp. n. 

http://zoobank.org/AE612016-7D21-48F6-8366-B289560ED3BC

Figs 19 , 90 , 210–217 , 218 , 313

##### Diagnosis.

Males of *Avicularia caei* sp. n. resemble those of *Avicularia minatrix*, *Avicularia hirschii* and *Avicularia lynnae* sp. n. by tibia I with discrete elevation covered by a cluster of setae in apical portion, on prolateral side (Fig. 216). They can be distinguished from all species except *Avicularia lynnae* sp. n. by very long embolus, more than 4 times tegulum’s width in retrolateral view (Fig. 211). Males of *Avicularia caei* sp. n. can be distinguished from males of *Avicularia lynnae* sp. n. by weakly-developed prominence on tegulum (Fig. 212) and abdomen with lateral dark stripes (Fig. 218). Female is unknown.

**Figures 210–217.**
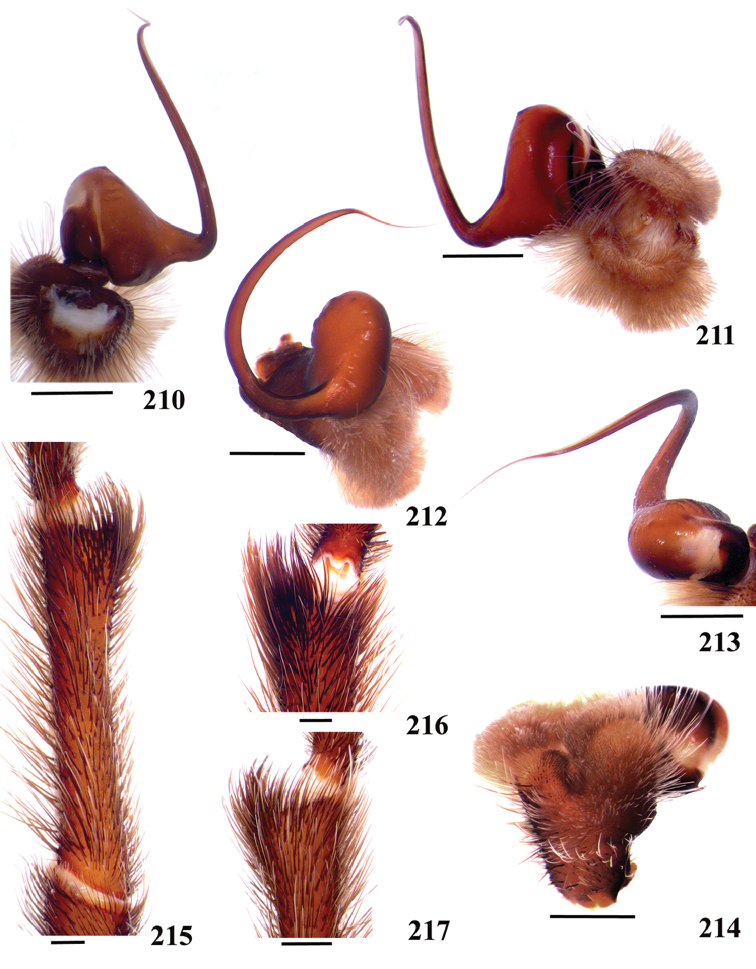
*Avicularia caei* sp. n., male holotype (MPEG 015637). **210–213** right palpal bulb (mirrored) **210** prolateral **211** retrolateral **212** frontal **213** dorsal **214** right cymbium, dorsal (mirrored) **215–217** right tibia I (mirrored) **215** prolateral **216** ventral **217** retrolateral. Scale bars = 1 mm.

##### Etymology.

This species is named after Carlos Eduardo Gurgel Paiola, aka Caê, in honor to his continuous support to one of the authors (CSF). This name is masculine in gender.

##### Material examined.

Holotype male, Brazil, Pará, Juruti, Acampamento Mutum (01°36'44.7"S, 56°11'39.2"W), L. T. Miglio col., 08 August 2008, ref. JURU 009 0043 (MPEG 15637).

**Figure 218.**
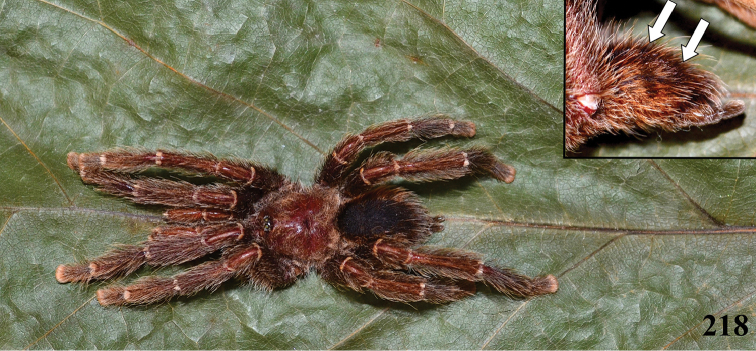
*Avicularia caei* sp. n, holotype, habitus, preserved male (MPEG 015637). Detail: lateral abdomen. Arrow indicating stripes. Photo: C. S. Fukushima.

##### Male.

**Description.**

MPEG 15637. Carapace: 10.44 long, 9.96 wide, 3.08 high. Chelicera: 3.45 long. Legs (femur, patella, tibia, metatarsus, tarsus, total): I: 11.01, 5.79, 8.44, 8.21, 4.47, 37.92. II: 10.37, 5.18, 8.05, 8.30, 4.35, 36.25. III: 9.19, 4.34, 7.17, 7.92, 4.02, 32.64. IV: 10.85, 5.26, 10.26, 10.66, 4.59, 41.62. Palp: 6.39, 3.96, 5.29, –, 2.62, 18.26. Midwidths: femora I–IV= 2.08, 2.08, 2.44, 1.89, palp= 1.44; patellae I–IV= 1.98, 2.10, 2.17, 1.90, palp= 1.48; tibiae I–IV= 1.25, 1.50, 1.47, 1.45, palp= 1.57; metatarsi I–IV= 1.10, 1.46, 1.02, 1.01; tarsi I–IV= 1.15, 1.37, 1.33, 1.15, palp= 1.64. Abdomen: 12.51 long, 7.59 wide. Spinnerets: PMS, 1.32 long, 0.63 wide, 0.19 apart; PLS, 2.31 basal, 1.16 middle, 2.04 distal; midwidths 1.17, 0.93, 0.76, respectively.

Carapace: 1.05 times longer than wide; cephalic region not raised, thoracic striae inconspicuous.

Fovea: shallow, recurve, 1.14 wide.

Eyes: eye tubercle 0.76 high, 1.72 long, 2.16 wide. Clypeus absent. Anterior row of eyes procurve, posterior slightly recurve. Eye size and interdistances: AME 0.50, ALE 0.57, PME 0.20, PLE 0.49, AME–AME 0.37, AME–ALE 0.20, AME–PME 0.07, ALE–ALE 1.41, ALE–PME 0.34, PME–PME 1.35, PME–PLE 0.02, PLE–PLE 1.70, ALE–PLE 0.29, AME–PLE 0.28.

Maxilla: length to width: 2.14. Cuspules: about 101 spread over ventral inner heel. Labium: 1.23 long, 1.65 wide, with 64 cuspules spaced by one diameter from each other on anterior half. Labium sternal groove shallow, flat, with two slightly separate, slim sigilla.

Chelicera: basal segment with 9 teeth and some small teeth on promargin. Sternum: 5.57 long, 4.49 wide. Sigilla: only posterior pair evident, oval, set at 45°angle, one diameter from margin.

Legs: Formula: IV=I II III. Length leg IV to leg I: 1.10. Clavate trichobothria: distal 1/2 tarsi I–II; 2/3 tarsus III, 1/2 tarsus IV. Scopula: Tarsi I–IV fully scopulate, IV with few sparse setae. Metatarsi I–II scopulate in distal 3/4; III 1/2; IV 1/4 distal scopulate. IV divided by row of setae.

Type II urticating setae: 0.99–1.05 long, 0.018–0.024 wide.

Palp (Figs 210–213, 313): globous bulb with small subtegulum and weakly-developed prominence on tegulum. Embolus: not flattened, lacking keels, 3.78 long in retrolateral view, about 4 times tegulum’s length. Medial portion and tegulum’s margin form an acute angle in retrolateral view. Proximal part very curved in frontal view; thin distal width, tapering distally; basal, middle, and distal width of 0.28, 0.15, 0.03, respectively. Tegulum: 1.62 long, 0.96 high in retrolateral view. Cymbium subtriangular with subequal lobes having well-developed rounded process bearing thin setae on retrolateral lobe (Fig. 214).

Tibia I with discrete elevation covered by cluster of setae in apical portion, on prolateral side (Figs 215–217).

Color pattern (Fig. 218): carapace orange with golden brown short body setae, thick longer setae spread over the carapace. Carapace border with long setae the same color as dorsal carapace short body setae. Coxae, labium, sternum and maxillae light brown, same color as ventral femora. Legs and palps with dark brown long guard-setae. Leg rings on distal femora, tibiae and metatarsi whitish. Abdomen dorsum with light brown guard-setae, light brown short body setae forming lateral stripes, and black short body setae forming a dark background. Ventral abdomen dark brown.

Female is unknown.

##### Color pattern ontogeny.

Immatures are unknown.

##### Distribution.

Brazil, known only from type locality (Fig. 90).

##### Natural history.

Unknown. A single specimen was found walking on forest ground (L. Miglio, pers. comm.).

### Taxonomic remarks

#### Avicularia vestiaria

Taxon classificationAnimaliaORDOFAMILIA

(De Geer, 1778), nomen nudum

Aranea vestiaria De Geer, 1778: 313; F. O. Pickard-Cambridge 1896: 741 (under Avicularia avicularia); Petrunkevitch 1911: 49 (under Avicularia avicularia); Roewer 1942: 253 (under Avicularia avicularia); Bonnet 1955: 828 (under Avicularia avicularia); World Spider Catalog 2016 (under Avicularia avicularia).
Avicularia vestiaria : Ausserer 1871: 201 (as senior synonym of Aranea avicularia), 1875: 184; Hasselt 1888: 166 (as senior synonym of Mygale avicularia*sensu* Latreille).

##### Remarks.

As stated by F. O. Pickard-Cambridge (1896), the name “*vestiaria*” is solely a word used for De Geer (1778) in his redescription of *Aranea avicularia* Linnaeus (1758) (Simon 1892: 172; F. O. Pickard-Cambridge 1896: 741), *in litt.* “*Aranea vestiaria hirsutissima nigro-fusca S. rufescens, plantis amplis tomentosis*” (De Geer 1778: 313). It is not a specific name as considered by Ausserer (1871). Even though other authors such as Ausserer (1875) considered it a valid name, we understand a formal description was not presented or intended and, therefore, *Avicularia vestiaria* (De Geer, 1778) is, herein, considered a *nomen nudum*.

#### Iridopelma leporina

Taxon classificationAnimaliaORDOFAMILIA

(C. L. Koch, 1841), comb. n., nomen dubium

Fig. 219

Mygale leporina C. L. Koch, 1841: 55 tab. CCCVI, fig. 726 (syntypes 4 dry, pinned immatures, Bahia, Brazil, Freir leg., ZMB 2028, examined,); Ausserer 1871: 217; F. O. Pickard-Cambridge 1896: 744. **Syn. n.**
Eurypelma leporina : C. L. Koch 1850: 74.
Avicularia leporina : Simon 1892: 172; Roewer 1942: 255; Bonnet 1955: 832; World Spider Catalog 2016.

##### Remarks.

**Figures 219–224.**
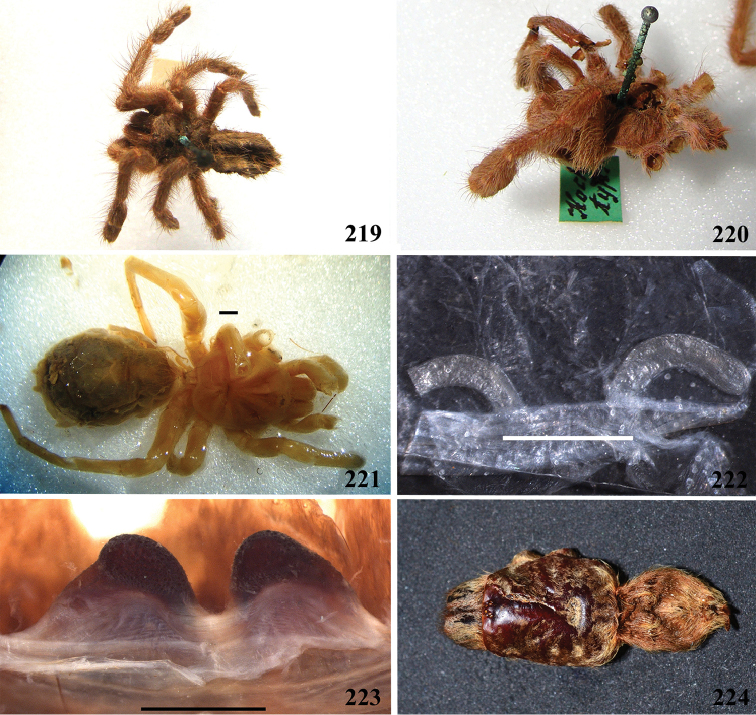
Species formerly included in *Avicularia*. **219** syntype of *Mygale leporina* C. L. Koch, 1841, immature, from state of Bahia, Brazil, (ZMB 2028), habitus **220** syntype of *Mygale plantaris* C. L. Koch, 1842, immature, from Brazil (ZMB 2026), habitus **221** *Euathlus affinis* (Nicolet, 1849) comb. n., lectotype female, from Santiago, Chile (MNHN–AR 4871B), habitus **222** *Avicularia arabica* (Strand, 1908) *nomen dubium*, holotype juvenile female, from El-Tor, Egypt (SMF 2660), spermathecae **223–224** *Thrixopelma aymara* (Chamberlin, 1916) comb. n., holotype female, from Aymara, Peru (MCZ 145) **223** spermatheca **224** habitus.

The syntypes are specimens of subfamily Aviculariinae because they have tarsi with well-developed scopulae, with a spatulated aspect (Fig. 219). They have anterior row of eyes procurved, spinnerets with the last article digitiform and no spines on legs. The color pattern of the immatures and the type locality, Bahia (Brazil), match with *Iridopelma* species. Thus, we decided to transfer *Mygale leporina* to *Iridopelma*, making the new combination *Iridopelma leporina* (C. L. Koch, 1841) comb. n. However, as the specimens are immatures and in poor conditions we consider *Iridopelma leporina* (C. L. Koch, 1841) comb. n. *nomen dubium*.

#### Iridopelma plantaris

Taxon classificationAnimaliaORDOFAMILIA

(C. L. Koch, 1842), comb. n., nomen dubium

Fig. 220

Mygale plantaris C. L. Koch, 1842; 71 tab. CCCXII, fig. 736 (syntypes 2 immatures, Brazil, ZMB 2026, examined). **Syn. n.**
Eurypelma plantaris : C. L. Koch 1850: 74.
Avicularia plantaris : Simon 1892: 172; World Spider Catalog 2016.

##### Remarks.

C. L. Koch (1842) published the description of *Mygale plantaris* in the same year of *Mygale leporina*. As *Mygale leporina*, the procurve anterior row of eyes, the digitiform posterior article of spinnerets, the color pattern and type locality (Brazil) of the syntypes match with *Iridopelma* (Fig. 220). Therefore, we transfer *Mygale plantaris* to *Iridopelma*, making the new combination *Iridopelma plantaris* (C. L. Koch, 1842) comb. n. Like *Mygale leporina*, the syntypes of *Mygale plantaris* are immature specimens. *Iridopelma* species present very intense ontogenetic changes in color pattern, thus different life stages present very distinct abdominal color pattern. This could be the reason why C. L. Koch (1842) described both forms as two different species when they could be the same. As we can’t assuredly affirm they are the same species, since the types are not adults or even in good condition, we decided to keep them as separate species and to consider *Iridopelma plantaris* (C. L. Koch, 1842) comb. n. *nomen dubium*.

#### Euathlus affinis

Taxon classificationAnimaliaORDOFAMILIA

(Nicolet, 1849) comb. n.

Figs 221 , 225

Mygale affinis Nicolet, 1849: 333, pl. 1, fig. 6 (female lectotype and immature paralectotype designated, herein, from Chile, Santiago [33°28'S, 70°38'W]), MNHN–AR 4871B, examined).
Brachypelma affinis : Mello-Leitão 1936d: 118; Bonnet 1955: 2989.
Eurypelma affine : Roewer 1942: 238.
Avicularia affinis : World Spider Catalog 2016.

##### Remarks.

**Figure 225.**
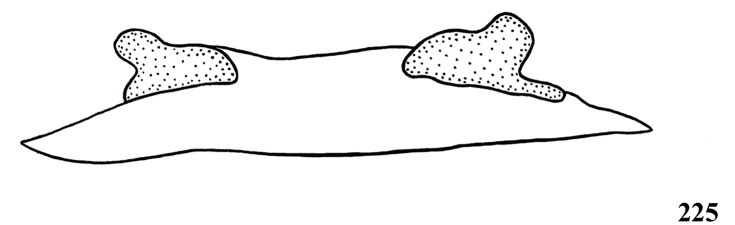
*Euathlus affinis* (Nicolet, 1849) comb. n., lectotype (MNHN–AR 4871B), spermathecae.

The lectotype female designated, herein, is a small specimen, with carapace 5.3 mm long (Fig. 221). It does not belong to any Aviculariinae species since it has spines on the legs and lacks the characteristic developed scopula on tarsi. Its spermathecae (Fig. 225) are compatible with *Euathlus* Ausserer, 1875 species (Perafán and Pérez-Miles 2014), which have species recorded for the area where the specimen was collected (Santiago, Chile). Furthermore, the original illustration published by Nicolet (1849, plate 1) shows a spider with reddish coloration and lacking two urticating setae patches, which is also compatible with *Euathlus* species, such as *Encyocratella parvulus* (Pocock, 1903) and *Euathlus condorito* Perafán & Pérez-Miles, 2014. Therefore, we transfer *Avicularia affinis* (Nicolet, 1849) to *Euathlus*, resulting in *Euathlus affinis* (Nicolet, 1849) comb. n.

#### Ischnocolus hirsutum

Taxon classificationAnimaliaORDOFAMILIA

Ausserer, 1875, nomen dubium

Ischnocolus hirsutus Ausserer, 1875: 170 (syntypes 11 small specimens from Cuba, BMNH 1890.7.1.347, and 2 small specimens from Bogota, Colombia [4°35'N, 74°04'W], in another vial, BMNH 1890.7.1.339+347, examined).
Eurypelma hirsutum : Roewer 1942: 240.
Avicularia hirsuta : World Spider Catalog 2016.

##### Remarks.

Ausserer (1875) mentioned several females from Cuba and two specimens from “Santa Fé de Bogotá” (known, nowadays, as Bogota, capital of Colombia) in Keyserling collection (housed at the BMNH). All examined specimens from Cuba have urticating setae type III on abdomen dorsum, and all specimens from Bogota have setae type I. Therefore, they belong to subfamily Theraphosinae and it is, herein, removed from the genus *Avicularia*. All syntypes are immatures lacking diagnostic characters and, therefore, we consider *Ischnocolus hirsutum* Ausserer, 1875 a *nomen dubium*.

#### Avicularia metallica

Taxon classificationAnimaliaORDOFAMILIA

Ausserer, 1875, nomen dubium

Avicularia metallica Ausserer, 1875: 185; Simon 1892: 171; Strand 1907b: 92; Mello-Leitão 1923: 377; Roewer 1942: 255; Bonnet 1955: 832; Petrunkevitch 1911: 50; 1939: 287; World Spider Catalog 2016.

##### Remarks.

The type was not found in Vienna collection where it should be deposited according to description. Thus, we consider *Avicularia metallica* Ausserer, 1875 as *nomen dubium*.

#### Ischnocolus gracilis

Taxon classificationAnimaliaORDOFAMILIA

Keyserling, 1891, nomen dubium

Ischnocolus gracilis Keyserling, 1891: 11 (holotype immature male, Brazil, Rio de Janeiro, Monte Verde [21°27'S, 41°55'W], BMNH 90.7.1.346, examined).
Eurypelma gracile : Roewer 1942: 239.
Avicularia gracilis : World Spider Catalog 2016.

##### Remarks.

The specimen is very small, the epigastric area is dissected, but there are no spermathecae. Abdomen dorsum is bald. Since the description mentions spines on posterior articles, we, herein, remove the species from the genus *Avicularia*. As the specimen is immature, damaged, and lacking diagnostic characters, therefore we consider, herein, *Ischnocolus gracilis* Keyserling, 1891 a *nomen dubium*.

#### Grammostola subvulpina

Taxon classificationAnimaliaORDOFAMILIA

(Strand, 1906) comb. n.

Avicularia subvulpina Strand, 1906a: 22 (holotype male, no further information, MWNH 362, examined by photo); 1907j: 233; Mello-Leitão 1923: 377; Petrunkevitch 1939: 288; Roewer 1942: 254; Bonnet 1955: 833; World Spider Catalog 2016.

##### Remarks.

A specimen that fits Strand’s (1906a) description was found in Wiesbaden collection and is, herein, considered as the holotype. The male resembles a characteristic *Grammostola* Simon, 1892 species from Brazil. Thus, we transfer *Avicularia subvulpina* to *Grammostola*, making the new combination *Grammostola subvulpina* (Strand, 1906) comb. n.

#### Avicularia arabica

Taxon classificationAnimaliaORDOFAMILIA

(Strand, 1908), nomen dubium

Fig. 222

Avicuscodra arabica Strand, 1908: 771 (holotype female, Egypt, El-Tor [28°14'N, 33°37'E] (Tor, Arabien [*sic*]), Rüppell, SMF 2660, examined); 1916: 20; Roewer 1942: 256.
Chaetopelma arabica : Raven 1985: 149.
Avicularia arabica : Gallon 2008: 243, figs 39–41; World Spider Catalog 2016.

##### Remarks.

The holotype of *Avicuscodra arabica* was recently rediscovered by Gallon (2008) in SMF. The specimen, supposedly from Tor, Egypt, is clearly an *Avicularia* as proposed by Gallon (2008). It has two spermathecae lacking lobes and with accentuated outwards curvature medially. Urticating setae of type II are present on abdomen dorsum. These characters are present in *Avicularia* species, which are restricted to Central and South America. As Gallon (2008) stated, the presence of a tropical American specimen together with Arabian material is most likely a mistake made by a nonspecialist before Strand’s examination.

Even though it is possible to identify the specimen as belonging to the genus *Avicularia*, it is a small female in poor condition, without evident color pattern and no other diagnostic characteristic. It is not possible to assure the specimen identity since spermathecae morphology is similar in most *Avicularia* species (Fig. 222). Furthermore, there is no indication of where the specimen came from. Thus, *Avicularia arabica* (Strand, 1908) is, herein, considered *nomen dubium*.

#### Thrixopelma aymara

Taxon classificationAnimaliaORDOFAMILIA

(Chamberlin, 1916) comb. n.

Figs 223–224

Eurypelma aymara Chamberlin, 1916: 201 (holotype female, Peru, Aymara, Dr. W. H. Jones col., MCZ 145, examined); Bonnet 1955: 1831.
Eurypelma aymarum : Roewer 1942: 238.
Avicularia aymara : World Spider Catalog 2016.

##### Remarks.

The specimen is not an aviculariine since it has no spatulate scopulae, bears two rounded spermathecae, and has no type II urticating setae (Fig. 224). The type locality, spermathecae morphology (Fig. 223) and presence of urticating setae type III match with the diagnostic characteristics of *Thrixopelma* species. Thus, we transfer *Eurypelma aymara* to *Thrixopelma*, making the new combination *Thrixopelma aymara* (Chamberlin, 1916) comb. n.

#### Avicularia aurantiaca

Taxon classificationAnimaliaORDOFAMILIA

Bauer, 1996, nomen dubium

Avicularia aurantiaca Bauer, 1996: 2, figs 1–4; World Spider Catalog 2016.

##### Material examined.

Female spermathecae exuvia in microscope slide, Peru, G. Schmidt ded. (SMF 58271-84).

##### Remarks.

The original description (Bauer 1996) stated he intended to deposit a female specimen at SMF as holotype, a slide prepared with spemathecae from an exuvia and a paratype male. The holotype and paratype specimen were never deposited in SMF collection. The description states female has leg IV longer than leg I, spermathecae with midwidth not expanded and orange leg rings (explaining why it was called “aurantiaca”). Thus, *Avicularia aurantiaca* is likely to be a junior synonym of *Avicularia rufa*. However, as there is no holotype and the description does not allow for a precise identification and characterization of the species, the name *Avicularia aurantiaca* Bauer, 1996 is considered, herein, as *nomen dubium*.

##### Types not found.

The following types have been unsuccessfully searched for within many arachnological collections and their descriptions do not allow for reliable identification. Thus, we consider the following species all *nomina dubia*: *Araneus hirtipes* Fabricius, 1793 *nomen dubium*; *Avicularia testacea* (C. L. Koch, 1841) *nomen dubium*; *Avicularia detrita* (C. L. Koch, 1842) *nomen dubium*; *Avicularia hirsutissima* (C. L. Koch, 1842) *nomen dubium*; *Avicularia holmbergi* Thorell, 1890 *nomen dubium*; *Ischnocolus doleschalli* Ausserer, 1871 *nomen dubium*; *Avicularia rapax* (Ausserer 1875) *nomen dubium*; *Avicularia ochracea* (Perty, 1833) *nomen dubium*; and *Avicularia walckenaerii* (Perty, 1833) *nomen dubium*.

#### Araneus hirtipes

Taxon classificationAnimaliaORDOFAMILIA

(Fabricius, 1787), nomen dubium

Aranea hirtipes Fabricius, 1787: 346, 1793: 428; Latreille 1806: 83 (under Mygale avicularia); Hahn 1833: 10 (under Mygale avicularia); Walckenaer 1837: 212 (under Mygale versicolor); World Spider Catalog 2016 (under Avicularia versicolor).
Mygale hirtipes : C. L. Koch 1836: 38.
Eurypelma hirtipes : C. L. Koch 1850: 74.
Avicularia hirtipes : Bonnet 1955: 831.

##### Remarks.

Considered synonym of *Mygale avicularia* by Latreille (1806) and Hahn (1833) as well as of *Mygale versicolor* by Walckenaer (1837). Although very uninformative, the description shows an eye disposition that suggests this species is not a mygalomorph spider. Thus, we considered *Araneus hirtipes* (Fabricius, 1787) *nomen dubium*.

#### Avicularia testacea

Taxon classificationAnimaliaORDOFAMILIA

(C. L. Koch, 1841), nomen dubium

Mygale testacea C. L. Koch, 1841: 45, Tab. CCCIII, fig. 719; F. O. Pickard-Cambridge 1896: 741 (under Avicularia avicularia); World Spider Catalog 2016 (under Avicularia avicularia).

##### Remarks.

F. O. Pickard-Cambridge (1896) considered it as a probable synonym of *Avicularia avicularia*. The specimen drawned by C. L. Koch (1841) resembles a specimen of *Avicularia* since it has well-developed scopula, anterior row of eyes procurve and long and curved palpal embolus. However, it is not possible to establish its identity without analyze the type. Therefore, herein, we removed it from the synonymy with *Avicularia avicularia* and consider *Avicularia testacea* (C. L. Koch, 1841) *nomen dubium*.

#### Avicularia detrita

Taxon classificationAnimaliaORDOFAMILIA

(C. L. Koch, 1842), nomen dubium

Mygale detrita C. L. Koch, 1842: 86, Tab. CCCXVIII, fig. 746; F. O. Pickard-Cambridge 1896: 744; Petrunkevitch 1911: 49; 1939: 286; Mello-Leitão 1923: 377; Roewer 1942: 254.
Avicularia detrita : Simon 1903: 958; Bonnet 1955: 830; World Spider Catalog 2016.

##### Remarks.

Analyzing Koch’s drawing, it is possible to note the absence of spatulated scopula, typical of Aviculariinae. Male palpal bulb also seems different from Aviculariinae species. Thus, it is probable that this species belongs to another subfamily, such as Theraphosinae.

#### Avicularia hirsutissima

Taxon classificationAnimaliaORDOFAMILIA

(C. L. Koch, 1842), nomen dubium

Mygale hirsutissima C. L. Koch, 1842: 76, fig. 738; Petrunkevitch 1911: 49 (under Avicularia avicularia); Roewer 1942: 254 (under Avicularia avicularia); Bonnet 1955: 829 (under Avicularia avicularia); World Spider Catalog 2016 (under Avicularia avicularia).

##### Remarks.

The drawn probably represents a specimen of *Avicularia* since it has well-developed scopula and the anterior row of eyes procurve. However, it is not possible to establish its identity without examining the type. Herein, we removed it from the synonymy with *Avicularia avicularia* established by Petrunkevitch (1911) and we consider *Avicularia hirsutissima* (C. L. Koch, 1842) *nomen dubium*.

#### Avicularia holmbergi

Taxon classificationAnimaliaORDOFAMILIA

Thorell, 1890, nomen dubium

Avicularia holmbergi Thorell, 1890: 399; F. O. Pickard-Cambridge 1896: 744; Simon 1892: 170; Petrunkevitch 1911: 50; 1939: 287; Mello-Leitão 1923: 377; Roewer 1942: 254; Bonnet 1955: 831; World Spider Catalog 2016.

##### Remarks.

This species was described in a work on the Indonesian fauna, but type locality was considered uncertain (Thorell 1890).

We consulted the curator of Arachnoidea Collection in the Natural History Museum of Vienna, where Ausserer’s specimens below were likely deposited. Unfortunately, the types are not there and descriptions do not allow reliable identification of the species.

#### Ischnocolus doleschalli

Taxon classificationAnimaliaORDOFAMILIA

Ausserer, 1871, nomen dubium

Ischnocolus doleschalli Ausserer, 1871: 189.
Eurypelma doleschalli Roewer 1942: 239.
Avicularia doleschalli : World Spider Catalog 2016.

##### Remarks.

Ausserer (1871) renamed as *Ischnocolus doleschalli* the species *Theraphosa bicolor* Doleschal (probably from Brazil), and added to Doleschal’s description that the specimen has spines on tibiae and metatarsi. Thus, it is not an Aviculariinae and, herein, we consider *Ischnocolus doleschalli* Ausserer, 1871 a *nomen dubium*.

#### Avicularia rapax

Taxon classificationAnimaliaORDOFAMILIA

(Ausserer, 1875), nomen dubium

Eurypelma rapax Ausserer, 1875: 200, pl. 7, fig. 45; Petrunkevitch 1911: 63; 1939: 264; Roewer 1942: 241; Bonnet 1955: 1833.
Avicularia rapax : World Spider Catalog 2016.

##### Remarks.

Ausserer (1875) described a male with two-branched tibial apophysis, and having setae on palpal tibia. It resembles a theraphosine specimen, but Ausserer did not give information on diagnostic characters. Thus, we consider *Avicularia rapax* (Ausserer, 1875) a *nomen dubium*.

We failed to find holotypes of both Perty’s species below. However, Perty (1833) drew in detail the legs of these species, showing well-developed scopulae on tarsi and metatarsi, with the spatulated aspect characteristic of aviculariines.

#### Avicularia ochracea

Taxon classificationAnimaliaORDOFAMILIA

(Perty, 1833), nomen dubium

Mygale ochracea Perty, 1833: 191, pl. XXXVIII, fig. 2; Walckenaer 1837: 215; C. L. Koch 1841: 48, pl. CCCIV, fig. 721.
Eurypelma ochracea : Ausserer 1871: 215; C. L. Koch: 1842: 48; 1850: 74; Petrunkevitch 1911: 63; 1939: 264.
Eurypelma ochraceum : Roewer 1942: 240; Bonnet 1955: 1832.
Avicularia ochracea : World Spider Catalog 2016.

##### Remarks.

*Mygale ochracea* was described as having an overall ocher coloration and a reddish abdomen. The specimen was found around Rio Negro River, in the province of the same name. At this time, the Rio Negro Province comprised the cities Barcelos and Vila da Barra do Rio Negro, now the city of Manaus, capital of the state of Amazonas (Instituto Histório e Geográfico Brasileiro *in* Spix and Martius 1823). The aviculariine species that fit Perty’s illustration and that occur in that area are *Avicularia avicularia* and *Avicularia juruensis*. However, based in Perty’s description it is not possible to determine *Avicularia ochracea* identity. Thus, we considered *Avicularia ochracea* (Perty, 1833) *nomen dubium*.

#### Avicularia walckenaerii

Taxon classificationAnimaliaORDOFAMILIA

(Perty, 1833), nomen dubium

Mygale walckenaerii Perty, 1833: 191, pl. XXXVIII, fig. 3; Walckenaer 1837: 217; C. L. Koch 1841: 46, pl. CCCIV, fig. 720.
Avicularia walckenaeri : Simon 1892: 171; Petrunkevitch 1911: 766; 1939: 288; Mello-Leitão 1923: 377; World Spider Catalog 2016.

##### Remarks.

*Mygale walckenaerii* was found in equatorial Brazil by Spix and Martius. The equatorial part of Brazil they explored were the states of Amazonas, Maranhão and Pará. *Mygale walckenaerii* was described as having an overall dark coloration, with greyish setae covering its body and “scarlet feet” (Perty 1833), in reference to vivid reddish rings on tarsi, metatarsi and tibiae. Based on the description (Perty 1833) and considering the probable occurrence area, the specimen depicted by Perty could be an *Avicularia rufa* or *Avicularia variegata* stat. n. As it is not possible to positively identify the species described by Perty (1833), we considered *Avicularia walckenaerii* (Perty, 1833) *nomen dubium*.

The holotypes of the species described by Tesmoingt (1996abc; 1999ab) are not at the Musée d’Histoire Naturelle de Lille, where they should be deposited according to descriptions. We contacted the author and he said the types have never been deposited in any collection. As it is not possible to be sure about the identity of this species only by the information contained in the descriptions, we consider *Avicularia azuraklaasi* Tesmoingt, 1996 *nomen dubium*, *Avicularia braunshauseni* Tesmoingt, 1999 *nomen dubium*, *Avicularia geroldi* Tesmoingt, 1999 *nomen dubium*, *Avicularia huriana* Tesmoingt, 1996 *nomen dubium*, and *Avicularia ulrichea* Tesmoingt, 1996 *nomen dubium*.

#### Avicularia azuraklaasi

Taxon classificationAnimaliaORDOFAMILIA

Tesmoingt, 1996, nomen dubium

Avicularia azuraklaasi Tesmoingt, 1996a: 8, figs 1–8; World Spider Catalog 2016.

#### Avicularia braunshauseni

Taxon classificationAnimaliaORDOFAMILIA

Tesmoingt, 1999, nomen dubium

Avicularia braunshauseni Tesmoingt, 1999a: 14, figs 1–5; World Spider Catalog 2016.

#### Avicularia geroldi

Taxon classificationAnimaliaORDOFAMILIA

Tesmoingt, 1999, nomen dubium

Avicularia geroldi Tesmoingt, 1999b: 17, figs 1–8; World Spider Catalog 2016.

#### Avicularia huriana

Taxon classificationAnimaliaORDOFAMILIA

Tesmoingt, 1996, nomen dubium

Avicularia huriana Tesmoingt, 1996b: 6, figs 1–18; 1996c: 2, figs 1A, 2–7, Bullmer et al. 2006: 7, figs 11–14; World Spider Catalog 2016.

#### Avicularia ulrichea

Taxon classificationAnimaliaORDOFAMILIA

Tesmoingt, 1996, nomen dubium

Avicularia sp. Tesmoingt, 1996b: 6, fig. unnumbered.
Avicularia ulrichea Tesmoingt, 1996c: 2, fig. 1B; World Spider Catalog 2016.

##### Types considered lost, herein.

#### Avicularia soratae

Taxon classificationAnimaliaORDOFAMILIA

Strand, 1907, nomen dubium

Avicularia soratae Strand, 1907d: 556; 1907e: 260; Petrunkevitch 1911: 50; 1939: 288; Mello-Leitão 1923: 377; Roewer 1942: 255; World Spider Catalog 2016.
Avicularia soratensis : Bonnet 1955: 832.

##### Remarks.

Strand (1907e) indicated the type is deposited in Lübeck Museum. However, the former collections of the old museum were destroyed in 1942 during World War II (S. Füting, pers. comm.). Thus, the type is lost and *Avicularia soratae* Strand, 1907 is considered, herein, as *nomen dubium*.

The three species below were deposited in Staatliches Museum für Naturkunde Stuttgart (SMF). Its arachnological collection was destroyed in World War II and contained at least 169 types of Strand, 9 types of C. Koch and 3 types of Keyserling (Renner 1988). The names *Avicularia fasciculata* Strand, 1907, *Avicularia fasciculata clara* Strand, 1907 and *Avicularia surinamensis* Strand, 1907 are, herein, considered as *nomina dubia*.

#### Avicularia fasciculata

Taxon classificationAnimaliaORDOFAMILIA

Strand, 1907, nomen dubium

Avicularia fasciculata Strand, 1907b: 92; 1907c: 465; Mello-Leitão 1923: 377; Petrunkevitch 1939: 286; Roewer 1942: 254; Bonnet 1955: 831; World Spider Catalog 2016.

#### Avicularia fasciculata clara

Taxon classificationAnimaliaORDOFAMILIA

Strand, 1907, nomen dubium

Avicularia fasciculata clara Strand, 1907c: 467; Roewer 1942: 254; World Spider Catalog 2016.

#### Avicularia surinamensis

Taxon classificationAnimaliaORDOFAMILIA

Strand, 1907, nomen dubium

Avicularia surinamensis Strand, 1907b: 90; 1907b: 465; Mello-Leitão 1923: 377; Petrunkevitch 1939: 288; Roewer 1942: 255; Bonnet 1955: 833, World Spider Catalog 2016.

##### Note.

The *Avicularia* species considered *nomina dubia* and *nomina nuda* by World Spider Catalog (2016) do not have their status changed.

#### Caribena gen. n.

Taxon classificationAnimaliaORDOFAMILIA

http://zoobank.org/45A06052-FAED-48FB-8307-72E3A74048ED

##### Diagnosis.

Females and males differ from those of all Aviculariinae genera by having type II urticating setae longer (more than 1 mm long) and slender (less than 0.009 mm wide) (Figs 18, 243) on a conspicuous patch on abdomen dorsum (Figs 241, 254). Additionally, males have a sharp spiniform process in the retrolateral lobe of cymbium (Fig. 306).

##### Etymology.

From the Spanish word “caribeña”, meaning “from Caribbean”, the occurrence area of this genus, and is considered feminine in gender.

##### Description.

Carapace longer than wide, cephalic region slightly raised. Cephalic and thoracic striae inconspicuous or evident. Fovea deep, straight or recurve. Chelicera without rastelum. Eye tubercle distinct, slightly raised to raised, wider than long. Anterior row of eyes procurve, posterior slightly recurve. Clypeus narrow or absent. Labium subquadrate, longer than wide, with 95–115 cuspules spaced by one diameter from each other on anterior third center. Maxillary lyra absent. Maxilla subretangular, anterior lobe distinctly produced into conical process, inner angle bearing 100–200 cuspules. Sternum longer than wide, with posterior angle acute, not separating coxae IV. Anterior pair of sigilla not evident, middle fusiform, posterior rounded or ellipsoidal; all less than one diameter from margin. Leg formula: I=IV II III. Clavate trichobothria on distal 2/3 or 1/2 of tarsi I–IV. Tarsi I–IV fully scopulate, IV divided or not by a band of setae. Metatarsi I–II fully scopulate, III 1/2 to 2/3 distal scopulate, IV 1/2 to 1/3 distal scopulate. Metatarsi IV divided by row of setae. Scopulae of tarsi and metatarsi I–II extended very laterally giving them spatulate appearance. Femora IV without retrolateral scopulae. Stridulatory setae absent. Leg aspinose. ITC absent; STC without denticles. Posterior lateral spinneret distally elongating, digitiform. Type II urticating setae very slender on dorsal abdomen, with barbs along entire length in both sexes (Figs 18, 243). Tibial apophysis single branch on prolateral leg I, with weakly-developed base and grouped spiniform setae distally (Fig. 310). Tibial apophysis on leg II absent. Male metatarsus I touches retrolaterally tibial apophysis’ setae when folded. Globous bulb with small subtegulum, lacking prominence (*Caribena laeta* comb. n., Fig. 233) or with developed prominence on tegulum (*Caribena versicolor* comb. nov., Fig. 246). Embolus not flattened, lacking keels, about 3.0 to 3.5 times tegulum’s length. Embolus medial portion and tegulum’s margin forming an obtuse (*Caribena laeta* comb. n., Fig. 232) or an acute angle (*Caribena versicolor* comb. n., Fig. 245) in retrolateral view. Embolus proximal part slightly curved (*Caribena laeta* comb. n., Fig. 233) or very curved (*Caribena versicolor* comb. n., Fig. 246) in frontal view; thin distal width, tapering distally (Figs 234; 318). Cymbium subtriangular with subequal lobes, having well-developed sharp process on retrolateral lobe, bearing thin setae (Figs 235, 306). Two completely separated, not-twisted, short (*Caribena laeta* comb. n., Fig. 227) or long spermathecae (*Caribena versicolor* comb. n., Fig. 230); with apex with intumescence (*Caribena laeta* comb. n., Fig. 227) or lacking it (*Caribena versicolor* comb. n., Fig. 230). Spermathecae almost straight (*Caribena laeta* comb. n., Fig. 227) or with an accentuated outwards curvature medially (*Caribena versicolor* comb. n., Fig. 230). Spermatheca midwidth as wide as its base width and weakly-sclerotized area shorter than half the length of well-sclerotized area. Drastic ontogenetic changes on abdominal color pattern. Juveniles have green metallic sheen, no black tarsi (Figs 240, 252) and abdomen with dorsal central longitudinal black stripe connected (*Caribena versicolor* comb. n., Fig. 252) or not (*Caribena laeta* comb. n., Fig. 240) with conspicuous transversal black stripes. When mature, they lose this pattern.

##### Type species.

*Mygale laeta* C. L. Koch, 1842, herein, designated.

##### Species included.

*Caribena laeta* (C. L. Koch, 1842) comb. n. and *Caribena versicolor* (Walckenaer, 1837) comb. n.

##### Distribution.

**Figure 226.**
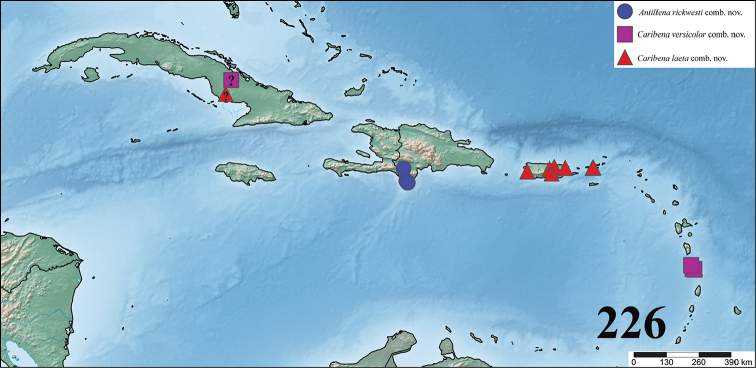
Map showing records of *Caribena* gen. n. and *Antillena* gen. n. ? = uncertain record.

Puerto Rico, the U. S. Virgin Island and Martinique (Fig. 226).

##### Key to males

**Table:** 

1	Embolus with proximal part slightly curved in frontal view (Fig. 233), medial portion and tegulum’s margin form an obtuse angle in retrolateral view (Fig. 232); tegulum lacking prominence (Fig. 233)	***Caribena laeta* comb. n.**
–	Embolus with proximal part very curved in frontal view (Fig. 246), medial portion and tegulum’s margin form an acute angle in retrolateral view (Fig. 245); tegulum with developed prominence (Fig. 246)	***Caribena versicolor* comb. n.**

##### Key to females

**Table:** 

1	Spermathecae almost straight, with intumescence on its apex (Fig. 227)	***Caribena laeta* comb. n.**
–	Spermathecae with accentuated outwards curvature medially, lacking intumescence on its apex (Fig. 230)	***Caribena versicolor* comb. n.**

#### Caribena laeta

Taxon classificationAnimaliaORDOFAMILIA

(C. L. Koch, 1842) comb. n.

Figs 226 , 227–229 , 231–238 , 239–242 , 243 , 310

Mygale laeta C. L. Koch, 1842: 66, pl. CCCX, fig. 732 (lectotype female and paralectotype immature, here designated, Puerto Rico, Moritz leg., ZMB 2045, examined); Ausserer 1871: 217; F. O. Pickard-Cambridge 1896: 744.
Mygale caesia C. L. Koch, 1842: 83, tab. CCCXVII, fig. 744 (syntypes 4 immatures, Puerto Rico, ZMB 2034, examined); F. O. Pickard-Cambridge 1896: 744. **Syn. n.**
Avicularia caesia : Simon 1892: 171; Petrunkevitch 1911: 49, 1929: 20, 51, figs 32–35, 1939: 285; Roewer 1942: 254; Bonnet 1955: 830; World Spider Catalog 2016.
Avicularia laeta : F. O. Pickard-Cambridge 1896: 744; Petrunkevitch 1911: 50, 1929: 20, 31, 48, fig. 31, 1939: 287; Mello-Leitão 1923: 377; Roewer 1942: 255; Bonnet 1955: 832; World Spider Catalog 2016.

##### Diagnosis.

**Figures 227–230.**
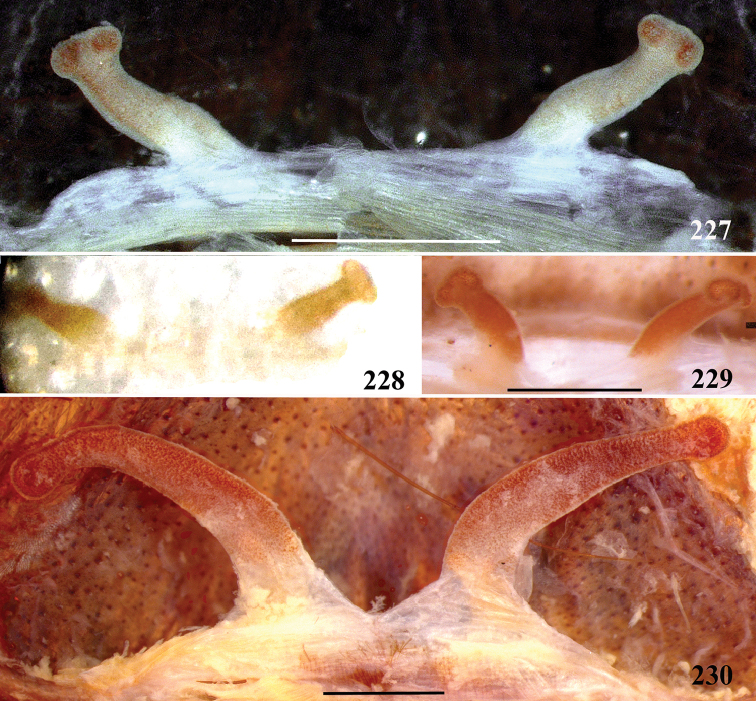
*Caribena* gen. n., spermathecae **227–229** *Caribena laeta* (C. L. Koch, 1842) comb. n., spermathecae variation. **227** Corozal, Puerto Rico (MNRJ 06909) **228** syntype, Puerto Rico (ZMB 2045) **229** Saint John, Virgin Islands (AMNH VI) **230** *Caribena versicolor* (Walckenaer, 1837) comb. n., Martinique (MNHN–AR 4904). Scale bars = 1 mm.

Females can be distinguished from females of *Caribena versicolor* comb. n. by almost straight spermathecae with intumescence on their apex (Figs 227–229). Males differ from males of *Caribena versicolor* comb. n. by lacking prominence on tegulum, by having embolus medial portion and tegulum’s margin forming an obtuse angle in retrolateral view and by slightly curved shape of basal part of embolus in frontal view (Fig. 233).

##### Material examined.

Puerto Rico, Corozal [18°18'N, 66°19'W], 1 female, M. Martinez col., 19 September 1994 (MNRJ 06909); Puerto Rico. Bayamon [18°22'N, 66°09'W], 1 male, 19 November 1932 (AMNH PR).

##### Additional material.

CUBA: 1 female (ZMB 30732); PUERTO RICO: 1 female (ZMB 30757); Road 10, km 21, 1 female, V. Roth col., 12 July 1977, in foliage of *Cecropia* tree (AMNH RW05); Aibonito [18°08'N, 66°15'W], 1 juvenile female, 1–3 June 1915, ref. 3553 (AMNH PR); 1 immature male (AMNH 1.15); Bayamon [18°22'N, 66°09'W], 1 male, T. Fuentes col., 12 September 1989 (AMNH RW04); Corozal [18°18'N, 66°19'W], 1 male, M. Martinez col., 26 September 1994 (MNRJ 06908); 1 male, same collector, 17 September 1991 (MNRJ 06906); 1 male, same collector, 21 September 1991 (MNRJ 06907); Mayaguez [18°12'N, 67°08'W], Tela: Ninguna, 1 female, A. Ruiz col., 15 February 1962 (AMNH PR); Rio Grande, El Yunque [18°20'N, 65°45'W], 1 female, J. Coffey col., 11 October 1993, in silk retreat in bromeliad (AMNH RW03); 1 male, north slope of Turquillo mountains near El Yunque National Forest [18°17'N, 65°48'W], A. Sanchez col., 29 September 1997 (MNRJ 06905); VIRGIN ISLANDS: Saint John [18°20'N, 64°47'W], 1 female, A. Sanchez col., April 1999, in silken retreat in bromeliad (AMNH RW02); Cinnamon Bay [18°21'N, 64°45'W] trail, about 700 m, 1 immature male, R. Zueifel col., in forest, in a bark, during night, 9 July 1980 (AMNH VI); 1 male, 1 female, Island Project staff col., 18 December 1965 (AMNH VI).

##### Remarks.

The syntypic series of *Mygale laeta* is constituted by specimens poorly conserved: a specimen without spermatheca (thereafter considered immature) and female with characteristic spermathecae, as reason why it was chosen to be lectotype.

The syntypic series of *Mygale caesia* is composed of four very small specimens, with central dark longitudinal stripe and dark transversal stripes on each side of abdomen. They retain the green-blue sheen illustrated on the drawings of C. L. Koch (1842), a pattern characteristic of juveniles from many aviculariines. C. L. Koch (1842) described these specimens as being a second species of *Mygale* in Puerto Rico (the first one was *Mygale laeta*). However, it is very probable that both are the same species as it was not found any other aviculariine species in Puerto Rico and these immatures are very similar to *Caribena laeta* comb. n. immatures. Thus, we consider *Mygale caesia* as the immature stage of *Mygale laeta*. Since both species were described in the same paper, by page priority *Mygale caesia* C. L. Koch, 1842 is, herein, considered junior synonym of *Mygale laeta* C. L. Koch, 1842 new synonymy.

##### Female.

**Redescription.** AMNH RCW. Carapace: 16.98 long, 14.82 wide, 5.01 high. Chelicera: 7.55 long. Legs (femur, patella, tibia, metatarsus, tarsus, total): I: 13.84, 8.48, 11.16, 9.24, 5.44, 48.16. II: 12.68, 7.52, 9.63, 8.80, 4.84, 43.47. III: 11.87, 6.85, 8.75, 8.73, 4.51, 40.71. IV: 13.88, 7.54, 11.18, 10.89, 3.92, 47.41. Palp: 9.55, 5.71, 6.27, –, 5.97, 27.50. Midwidths: femora I–IV= 3.13, 2.62, 3.08, 3.08, palp= 2.58; patellae I–IV= 3.16, 2.99, 3.18, 3.01, palp= 2.50; tibiae I–IV= 2.45, 2.56, 2.43, 2.73, palp= 2.01; metatarsi I–IV= 1.98, 1.94, 1.89, 1.87; tarsi I–IV= 2.24, 2.07, 2.14, 2.15, palp= 2.28. Abdomen: 23.50 long, 17.64 wide. Spinnerets: PMS, 1.73 long, 0.90 wide, 0.43 apart; PLS, 1.87 basal, 1.67 middle, 2.80 distal; midwidths 1.30, 1.17, 0.76, respectively.

Carapace: 1.15 times longer than wide; cephalic region not raised, thoracic striae inconspicuous.

Fovea: deep, straight, 2.06 wide.

Eyes: eye tubercle 1.07 high, 2.43 long, 3.51 wide. Clypeus 0.20. Anterior row of eyes procurve, posterior row slightly recurve. Eye size and interdistances: AME 0.80, ALE 0.86, PME 0.39, PLE 0.65, AME–AME 0.60, AME–ALE 0.51, AME–PME 0.17, ALE–ALE 2.00, ALE–PME 0.96, PME–PME 1.86, PME–PLE 0.28, PLE–PLE 2.38, ALE–PLE 0.49, AME–PLE 0.57.

Maxilla: length to width: 1.68. Cuspules: 100–200 spread over ventral inner heel. Labium: 2.28 long, 2.73 wide, with 97 cuspules spaced by one diameter from each other on anterior third. Labio-sternal groove shallow, flat, sigilla not evident.

Chelicera: basal segment with 12 teeth and some small teeth on promargin. Sternum: 8.34 long, 6.71 wide. Sigilla: anterior pair not evident, median fusiform, posterior elipsoidal, both less than one diameter from margin.

Legs: Formula: IV=I II III. Length leg IV to leg I: 0.98. Clavate trichobothria: distal 2/3 tarsi I–IV. Scopula: Tarsi I–IV fully scopulate. IV divided by row of setae. Metatarsi I–II fully scopulate; III–IV 1/2 distal scopulate. IV divided by row of setae.

Type II urticating setae (Fig. 243): 1.02–1.33 long, 0.006–0.009 wide.

Spermathecae (Fig. 227): two short, almost straight spermathecae bearing intumescence (not a well-defined lobe) in their apex. Midwidth as wide as its base width and weakly-sclerotized area shorter than half the length of well-sclerotized area.

Color pattern (Fig. 241): carapace brown with golden short body setae. Carapace border long setae the same color as dorsal carapace short body setae. Coxae, labium, sternum and maxillae brown, slightly darker than ventral femora. Legs and palps with golden short body setae with green sheen and dark brown long guard-setae, gradually lightening from base to tip. Leg rings on distal femora, tibiae and metatarsi whitish. Tarsi with an “U” shaped orange stripe. Abdomen with golden brown guard-setae homogeneously distributed and golden short body setae. Ventral abdomen brown. Urticating setae form very distinctive small golden patch on dorso posterior area of abdomen.

**Figures 231–238.**
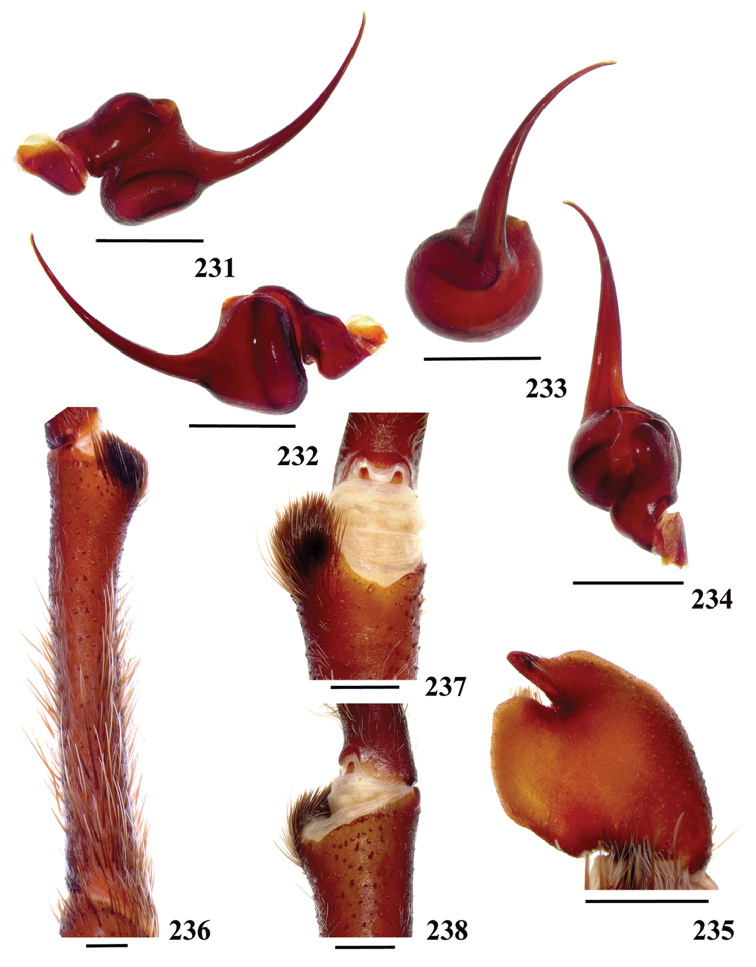
*Caribena laeta* (C. L. Koch, 1842) comb. n., male (AMNH PR). **231–234** left palpal bulb **231** prolateral **232** retrolateral **233** frontal **234** dorsal **235** left cymbium, dorsal **236–238** left tibial apophysis of leg I **236** prolateral **237** ventral **238** retrolateral. Scale bars = 1 mm.

##### Male.

**Description.** AMNH. Carapace: 10.22 long, 8.86 wide, 3.05 high. Chelicera: 2.96 long. Legs (femur, patella, tibia, metatarsus, tarsus, total): I: 11.12, 5.80, 9.30, 8.75, 4.43, 39.40. II: 10.20, 5.03, 8.86, 8.01, 4.77, 36.87. III: 9.20, 4.14, 7.14, 7.91, 4.06, 32.45. IV: 10.57, 4.85, 9.54, 9.48, 4.30, 38.74. Palp: 6.68, 3.90, 5.01, –, 1.76, 17.35. Midwidths: femora I–IV= 1.94, 1.80, 1.77, 1.90, palp= 1.53; patellae I–IV= 1.69, 1.75, 1.72, 1.70, palp= 1.38; tibiae I–IV= 1.53, 1.35, 1.41, 1.40, palp= 1.42; metatarsi I–IV= 1.16, 1.01, 1.02, 1.08; tarsi I–IV= 1.16, 1.07, 1.27, 1.27, palp= 1.29. Abdomen: 9.79 long, 6.92 wide. Spinnerets: PMS, 0.92 long, 0.41 wide, 0.18 apart; PLS, 1.47 basal, 0.80 middle, 1.56 distal; midwidths 0.78, 0.68, 0.48, respectively.

As in female, except:

Carapace: 1.15 times longer than wide.

Fovea: 1.02 wide.

Eyes: eye tubercle 0.73 high, 1.65 long, 2.16 wide. Clypeus absent. Eye size and interdistances: AME 0.61, ALE 0.61, PME 0.27, PLE 0.48, AME–AME 0.31, AME–ALE 0.25, AME–PME 0.11, ALE–ALE 1.27, ALE–PME 0.72, PME–PME 1.17, PME–PLE 0.07, PLE–PLE 1.67, ALE–PLE 0.24, AME–PLE 0.37.

Maxilla: length to width: 1.90. Cuspules: 174. Labium: 1.22 long, 1.45 wide, with 112 cuspules spaced by one diameter from each other on anterior half.

Chelicera: basal segment with 8 teeth and some small teeth on promargin. Sternum: 4.94 long, 3.73 wide. Sigilla: three pairs, posterior rounded, less than one diameter from margin.

Legs: Formula: IV=I II III. Length leg IV to leg I: 0.98. Metatarsus III scopulate in distal 2/3; IV, in distal 1/2.

Type II urticating setae: 1.41–1.71 long and 0.007–0.009 wide.

Palp (Figs 231–234): globous bulb with small subtegulum, lacking prominence on tegulum. Embolus: not flattened, lacking keels, 2.32 long in retrolateral view, about 3 times tegulum’s length. Medial portion and tegulum’s margin form an obtuse angle in retrolateral view. Proximal part slightly curved in frontal view; thin distal width, tapering distally; basal, middle, and distal width of 0.44, 0.12, 0.04, respectively. Tegulum: 1.25 long, 0.74 high in retrolateral view. Cymbium subtriangular with subequal lobes, having well-developed sharp process on retrolateral lobe, bearing thin setae (Fig. 235).

Tibial apophysis (Figs 236–238, 310): single branch on prolateral leg I, with weakly-developed base and grouped spiniform setae distally. Male metatarsus I touches retrolaterally tibial apophysis’ setae when folded.

**Figures 239–242.**
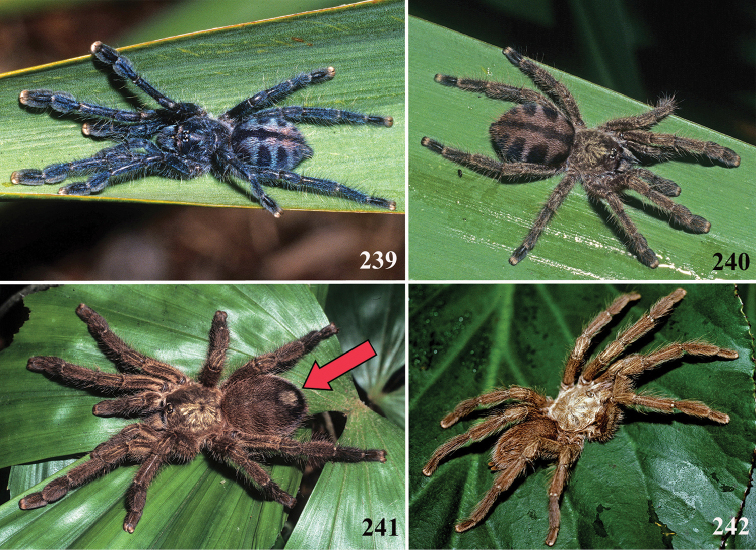
*Caribena laeta* (C. L. Koch, 1842) comb. n., Puerto Rico, habitus. **239** immature **240** juvenile **241** female **242** male. Arrow: Urticating setae type II on a conspicuous patch on dorsal abdomen. Photos: R. C. West.

##### Color pattern ontogeny.

Juveniles with metallic sheen, all articles with same blackish color (Fig. 240), and abdomen dorsum with dorsal central longitudinal black stripe disconnected from transversal black stripes. When mature, they lose this pattern (Figs 241–242).

**Figure 243.**
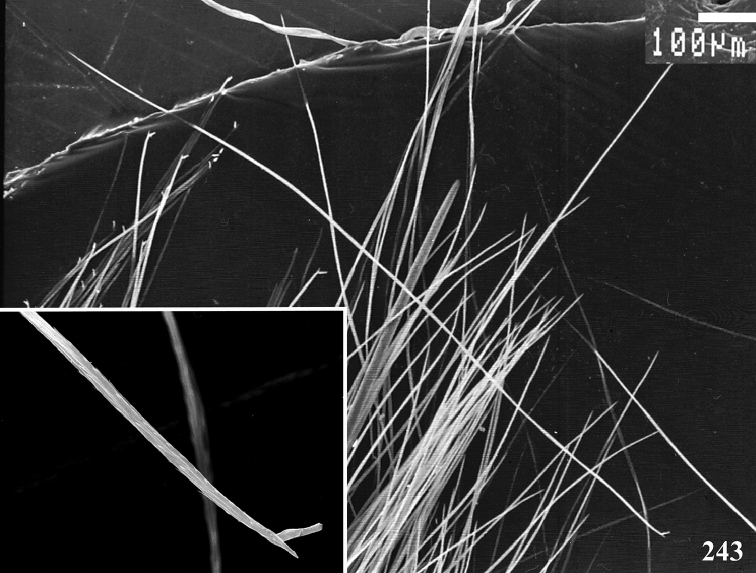
*Caribena laeta* (C. L. Koch, 1842) comb. n., SEM microphotographs of slender urticating setae type II, with barbs along all its length. Detail: region near stalk, female (MNRJ 06909). Scale bar = 100 μm.

##### Distribution.

Puerto Rico and the U. S. Virgin Island (Lesser Antilles) (Fig. 226). There is a dubious record of a single specimen from Cuba lacking any information on collectors and date.

##### Natural history.

Petrunkevitch (1929) mention that three females captured in Puerto Rico were found on retreats inside bromeliad leaves. The author also mentioned retreats of this species are similar to retreats of *Avicularia* species, consisting of a dense silk tube found in leaves and even in human constructions (Petrunkevitch 1929).

#### Caribena versicolor

Taxon classificationAnimaliaORDOFAMILIA

(Walckenaer, 1837) comb. n.

Figs 18 , 226 , 230 , 244–251 , 252–255 , 306

Mygale versicolor Walckenaer, 1837: 211 (holotype considered lost).
Avicularia rutilans Ausserer, 1875: 184, t. 7, fig. 34 (holotype male, N. Granada, Keyserling collection, BMNH 1890.7.1.359, examined); F. O. Pickard-Cambridge 1896: 744; Petrunkevitch 1911: 50, 1939: 28; Mello-Leitão 1923: 377; Roewer 1942: 255; Bonnet 1955: 832; World Spider Catalog 2016. **Syn. n.**
Avicularia versicolor : Simon 1892: 171, 172; Petrunkevitch 1911: 51, 766, 1939: 288; Mello-Leitão 1923: 377; Roewer 1942: 255; World Spider Catalog 2016.

##### Remarks.

*Avicularia rutilans* holotype has same morphology of palpal bulb, tibial apophysis and urticating setae II and the characteristic intense green sheen found in specimens from Martinique. Despite labeled as coming from N. Granada (Colombia, see discussion below), it is undoubtedly a specimen of *Caribena versicolor* comb. n. Thus, we consider *Avicularia rutilans* Ausserer, 1875 as junior synonym of *Caribena versicolor* (Walckenaer, 1837) comb. nov.

**Figures 244–251.**
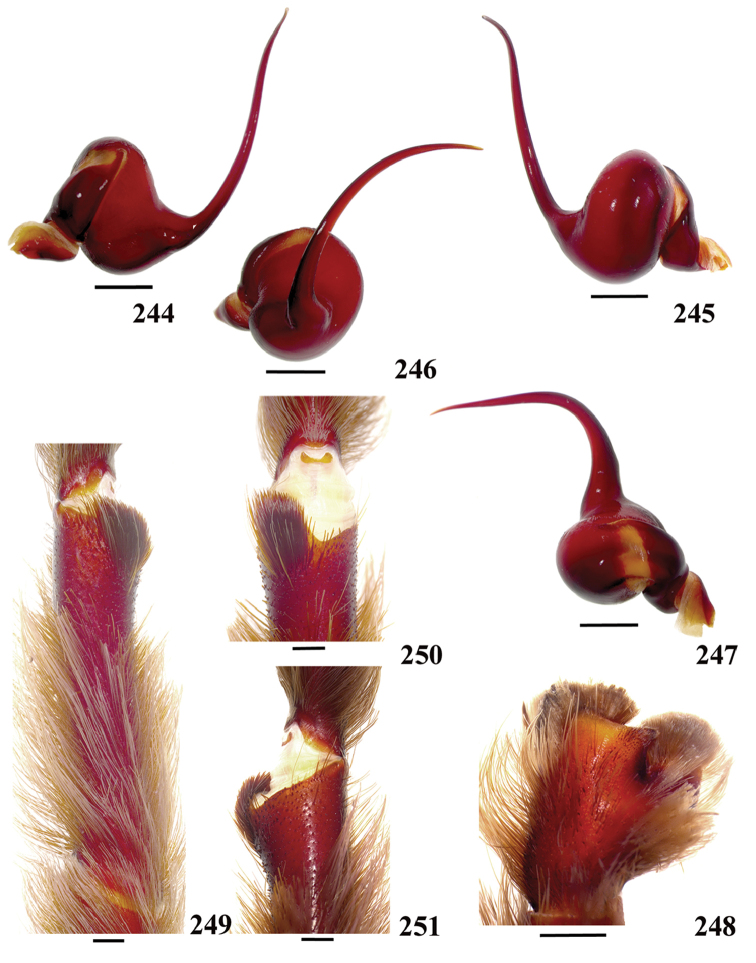
*Caribena versicolor* (Walckenaer, 1837) comb. n., male neotype (MNHN–AR 4904). **244–247** left palpal bulb **244** prolateral **245** retrolateral **246** frontal **247** dorsal **248** left cymbium, dorsal **249–251** left tibial apophysis of leg I **249** prolateral **250** ventral **251** retrolateral. Scale bars = 1 mm.

##### Diagnosis.

Females can be distinguished from those of *Caribena laeta* comb. n. by spermathecae with accentuated outwards curvature medially and by lacking intumescence on apex (Fig. 230). Males differ from males of *Caribena laeta* comb. n. by developed prominence on tegulum (Fig. 246), embolus medial portion and tegulum’s margin form an acute angle in retrolateral view (Fig. 245) and by very curved shape of basal part of embolus in frontal view (Fig. 246).

##### Type material.

Holotype considered, herein, lost since it is not in MNHN–AR where it should be deposited according to description (Walckenaer 1837). Here, we establish the male AR 4904 as the neotype since no holotype is believed to be extant and a type is considered necessary to define the taxon objectively because the original name is involved in a complex taxonomic problem (article 75.1, ICZN 1999).

**Figures 252–255.**
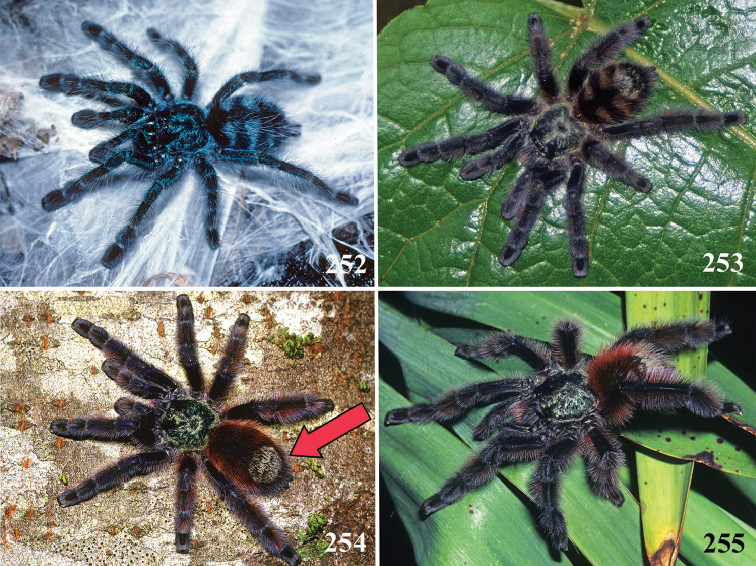
*Caribena versicolor* (Walckenaer, 1837) comb. n., habitus. **252** immature **253** juvenile **254** female **255** male. Arrow: urticating setae type II on conspicuous patch on dorsal abdomen. Photos: R. C. West.

##### Material examined.

Martinique, 6 females, 2 males (one male established here as neotype), 1 immature male, Gambey col., (MNHN–AR 4904, Box 314).

##### Additional material.

CUBA: 1 male, 2 females (MNHN–AR); MARTINIQUE: Le Carbet [14°42'N, 61°10'W], Morne Carbet, 1500 m, forest, 2 males, 18 March 1967 (AMNH); 1 male, Beatty col., 1967 (AMNH), from trees, 1 female (AMNH); Absalon [14°41'N, 61°6'W], 1 immature male, Vaunes, 16 June 1960 (AMNH Ma); Anse Céron [14°50'N, 61°13'W], 1 km SE, 1 male, J. Paterson col., 7 July 1998 (AMNH RW07), same data, 1 male (AMNH RW06); 1 male, Le Prêcheur, west of Mountain Pelée, P. Charbonnet col., 21 August 1998, on side of tree (MNRJ 06914).

##### Remarks.

In order to describe *Mygale versicolor*, Walckenaer (1837) used a female from Guadeloupe and a male from Brazil. Even though he considered *Aranea hirtipes* Fabricius, 1787 described from French Guiana as a synonym of *Mygale versicolor*, Walckenaer (1837) did not include Cayenne nor Brazil in the distribution area, only Guadeloupe and Martinique. Concerning the description, the female corresponds to *Caribena versicolor* comb. n., but the male did not, since it was described as having a two-branched tibial apophysis and occurs in Brazil. Therefore, the syntype series is composed of two different species, causing a complex taxonomic problem that lasted for years.

C. L. Koch (1842) apparently followed Walckenaer (1837) but formally extended the species distribution to Brazil. A few years later, the same author (C. L. Koch 1850) transferred this species to *Lasiodora*, creating the new combination *Lasiodora versicolor*.

In 1871, Ausserer (1871) erected the new genus *Homoeomma* Ausserer, 1871 with a single species, *Homoeomma versicolor* (Walckenaer, 1837). Ausserer (1871) examined a male and a female from Rio de Janeiro, Brazil and considered *Mygale versicolor* *sensu* C. L. Koch, 1842 and *Lasiodora versicolor* (C. L. Koch, 1850) synonyms of *Homoeomma versicolor*. Years later, O. Pickard-Cambridge (1881) explicitly considered as being distinct the concepts of *Mygale versicolor* from C. L. Koch (1842) and Walckenaer (1837).

He described a new species, *Homoeomma stradlingi* O. Pickard-Cambridge, 1881, based on the concept of *Mygale versicolor* used by C. L. Koch, and in a female and two males from Brazil (O. Pickard-Cambridge 1881). The species *Homoeomma versicolor* was included in the synonymic list with a question mark by O. Pickard-Cambridge (1881).

Simon (1892) considered that *Mygale versicolor* Walckenaer was erroneously reported as *Homoeomma* and it was, in fact, an *Avicularia* species very common in Lesser Antilles. As a consequence, it is likely Simon (1892) ignored the combination *Homoeomma versicolor* and considered *Homoeomma stradlingi* as the type species of *Homoeomma*

A problem lies with the identity of *Mygale versicolor* Walckenaer, 1837. This name is constantly applied to aviculariine species found in Martinique. However, as mentioned before, the type series consists of two different species: one aviculariine; and the other a theraphosine. As the description is vague and syntype series is here considered lost, we decide to establish a neotype in order to preserve the name’s stability and its usage.

##### Female.

**Redescription.** AR 4904. Carapace: 19.07 long, 16.75 wide, 5.33 high. Chelicera: 6.90 long. Legs (femur, patella, tibia, metatarsus, tarsus, total): I: 14.29, 8.76, 10.86, 9.49, 6.12, 49.52. II: 13.16, 7.74, 10.32, 9.23, 5.92, 46.37. III: 11.96, 7.64, 9.49, 9.40, 5.18, 43.67. IV: 14.86, 7.98, 12.21, 13.25, 5.99, 54.29. Palp: 10.16, 6.58, 6.79, –, 7.42, 30.95. Midwidths: femora I–IV= 3.61, 3.74, 3.80, 3.59, palp= 2.90; patellae I–IV= 3.67, 3.62, 3.30, 3.48, palp= 3.06; tibiae I–IV= 3.08, 2.69, 2.91, 3.06, palp= 2.83; metatarsi I–IV= 2.37, 2.27, 2.60, 2.03; tarsi I–IV= 2.86, 2.58, 2.75, 2.89, palp= 2.92. Abdomen: 22.09 long, 17.02 wide. Spinnerets: PMS, 1.45 long, 1.01 wide, 0.10 apart; PLS, 2.56 basal, 0.77 middle, 3.04 distal; midwidths 2.10, 1.75, 1.52, respectively.

Carapace: 1.14 times longer than wide; cephalic region not raised, carapace striae inconspicuous.

Fovea: deep, slightly recurve, 2.33 wide.

Eyes: eye tubercle 1.11 high, 2.20 long, 3.18 wide. Clypeus 0.64. Anterior row of eyes procurve, posterior slightly recurve. Eye size and interdistances: AME 0.78, ALE 0.83, PME 0.36, PLE 0.82, AME–AME 0.47, AME–ALE 0.51, AME–PME 0.22, ALE–ALE 1.83, ALE–PME 0.75, PME–PME 1.78, PME–PLE 0.12, PLE–PLE 2.38, ALE–PLE 0.43, AME–PLE 0.67.

Maxilla: length to width: 1.84. Cuspules: 100–200 spread over ventral inner heel. Labium: 2.07 long, 3.19 wide, with 115 cuspules spaced by one diameter from each other on anterior third. Labium sternal groove shallow, flat, sigilla not evident.

Chelicera: basal segment with 11 teeth and some small teeth on promargin. Sternum: 10.21 long, 6.83 wide. Sigilla: only posterior pair evident, rounded, less than one diameter from margin.

Legs: Formula: IV=I II III. Length leg IV to leg I: 1.10. Clavate trichobothria: distal 1/2 tarsi I–IV. Scopulae: Tarsi I–IV fully scopulate. Metatarsi I–II fully scopulate, III 1/2, IV 1/3 distal scopulate. IV divided by a row of setae.

Type II urticating setae: 1.33–1.58 long, 0.006–0.009 wide.

Spermathecae (Fig. 230): two completely separated, not-twisted long spermathecae, with walls lacking projections or lobes and accentuated outwards curvature medially. Midwidth as wide as its base width and weakly-sclerotized area shorter than half the length of well-sclerotized area.

Color pattern (Fig. 254): carapace brown with golden short body setae with very intense green sheen. Carapace border with long setae the same color as dorsal carapace short body setae, but with iridescent sheen. Coxae, labium, sternum and maxillae light brown, same color of ventral femora. Legs and palps with gold short body setae with green sheen and brown long guard-setae with very intense iridescent sheen. Leg rings on distal femora, tibiae and metatarsi same color as the rest of segment. Dorsal abdomen with vivid red long guard-setae with very intense iridescent sheen homogeneously distributed and black, short body setae. Ventral abdomen brown. Urticating setae form very distinctive small bronze patch on dorso posterior area of abdomen.

##### Male.

**Description.** AR 4904. Carapace: 16.01 long, 15.05 wide, 4.50 high. Chelicera: 6.16 long. Legs (femur, patella, tibia, metatarsus, tarsus, total): I: 14.39, 8.80, 11.38, 10.76, 6.18, 51.51. II: 13.64, 8.34, 11.03, 10.31, 5.80, 49.12. III: 12.28, 6.97, 9.43, 10.27, 5.57, 44.52. IV: 15.01, 7.65, 12.92, 13.89, 5.41, 54.88. Palp: 9.26, 5.83, 7.00, –, 2.93, 25.02. Midwidths: femora I –IV= 3.08, 3.13, 3.20, 2.92, palp= 2.24; patellae I–IV= 3.27, 2.88, 2.92, 3.06, palp= 2.30; tibiae I–IV= 2.59, 2.43, 2.32, 2.59, palp= 2.17; metatarsi I–IV= 1.61, 1.76, 1.85, 1.71; tarsi I–IV= 2.02, 2.01, 2.06, 2.07, palp= 2.23. Abdomen: 18.41 long, 13.01 wide. Spinnerets: PMS, 1.94 long, 0.92 wide, 0.30 apart; PLS, 2.40 basal, 1.14 middle, 2.64 distal; midwidths 1.52, 1.36, 1.14, respectively.

As in female, except:

Carapace: 1.06 times longer than wide.

Fovea: 1.27 wide.

Eyes: eye tubercle 1.14 high, 2.30 long, 3.01 wide. Clypeus 0.54. Eye size and interdistances: AME 0.78, ALE 0.70, PME 0.31, PLE 0.62, AME–AME 0.41, AME–ALE 0.51, AME–PME 0.13, ALE–ALE 1.94, ALE–PME 0.68, PME–PME 1.73, PME–PLE 0.16, PLE–PLE 2.14, ALE–PLE 0.18, AME–PLE 0.55.

Maxilla: length to width: 1.77. Labium: 1.89 long, 2.73 wide, with 95 cuspules. Labium sternal groove with two large separate sigilla.

Chelicera: basal segment with 10 teeth and some small teeth on promargin. Sternum: 7.49 long, 6.41 wide. Sigilla: three pairs, large posterior.

Legs: Length leg IV to leg I: 1.07. Scopula: tarsus IV with few, sparse setae. Metatarsus IV divided by a row of setae.

Type II urticating setae (Fig. 18): 1.61–1.75 long, 0.007–0.011 wide.

Palp (Figs 244–247): globous bulb with small subtegulum and developed prominence on tegulum. Embolus: not flattened, lacking keels, 4.64 long in retrolateral view, about 3 times tegulum’s length. Medial portion and tegulum’s margin form an acute angle in retrolateral view. Proximal part very curved in frontal view; thin distal width, tapering distally; basal, middle, and distal width of 0.70, 0.22, 0.08, respectively. Tegulum: 2.42 long, 1.51 high in retrolateral view. Cymbium subtriangular with subequal lobes, having developed sharp process on retrolateral lobe, bearing thin setae (Figs 248, 306).

Tibial apophysis (Fig. 250): single branch on prolateral leg I, with weakly-developed base and grouped spiniform setae distally. Male metatarsus I touches retrolaterally tibial apophysis’ setae when folded.

##### Color pattern ontogeny.

Juveniles with metallic sheen, all articles with same blackish color (Fig. 252) and abdomen dorsum with dorsal central longitudinal black stripe connected with all transversal black stripes.(Fig. 253). When mature, they lose this pattern (Figs 254–255).

##### Distribution.

Martinique (Fig. 226).

##### Remark.

Contrasting with Walckenaer’s description (Walckenaer 1837), this species is not found on Guadeloupe Island (P. Marechal, pers. comm.). Thus, it is possible that Walckenaer (1837) used a specimen from Martinique, which is close to Guadeloupe Island and also French territory, but recorded as coming from Guadeloupe. Similar situations were very common in past centuries.

Occurrence of *Caribena versicolor* comb. n. in Cuba is still doubtful since the specimens here analyzed are very old and the only ones known from Cuba in the entirety of arachnological collections examined. The record of *Avicularia rutilans*, junior synonym of *Caribena versicolor* comb. n. in Nova Granada, former name of Colombia, is also doubtful. There is no other record of this species in Colombia nor in any other continental territory. An explanation could be that this specimen was dispatched from Bogota, an important South Amercian commercial center in eighteenth and nineteenth centuries, which is near the Antilles.

##### Natural history.

It was poorly known until a few years ago. The situation changed since an extensive and detailed ecological and populational study was carried out by Maréchal et al. (2009) on Martinique. They pointed out that *Caribena versicolor* comb. n. prefers mesophyll forests, building its retreat in Bromeliacea leaves, between tree branches, inside bamboo trunks, over tree trunks and also within human constructions (Maréchal et al. 2009). Its life cycle is about two years, reaching sexual maturity around 18 months, after approximately ten molts; the reproductive period is in March and eggsac eclosion is in May and June (Maréchal et al. 2009).

##### Remarks.

Two color forms are known, one with specimens having leg and palp hairs in bright red and the other with specimens with darker hairs on legs and palps.

#### Ybyrapora gen. n.

Taxon classificationAnimaliaORDOFAMILIA

http://zoobank.org/68B8A50A-E657-4091-8931-58859EF55AAC

Figs 256 , 257–259 , 260–267 , 268–275 , 276–283 , 295–298 , 305 , 308 , 319

##### Diagnosis.

*Ybyrapora* gen. n. can be distinguished from all other aviculariine genera, except *Avicularia*, *Caribena* gen. n., *Iridopelma* and *Typhochlaena* by procurve anterior row of eyes. It can be distinguished from *Typhochlaena* by digitiform apical article of PLS. From *Iridopelma* by males lacking tibial apophysis on tibiae II and females by long spermathecae, with accentuated outwards curvature medially. It differs from *Caribena* gen. n. by stout urticating setae on abdomen dorsum of male and female, and by lacking sharp process bearing thin setae on retrolateral lobe of cymbium in males. From *Avicularia* females it can be distinguished by having virtually non-sclerotized spermathecae (Figs 257–259). From *Avicularia* males (except *Avicularia minatrix*, *Avicularia lynnae* sp. n., *Avicularia caei* sp. n., and *A hirschii*) it differ by lacking developed tibial apophysis (Fig. 308). Males of *Ybyrapora diversipes* comb n. can be distinguished from those of four species by having very long embolus strongly curved in frontal view (Fig. 278). Males of *Ybyrapora sooretama* comb. n. and *Ybyrapora gamba* comb. n. differ from those of *Avicularia lynnae* sp. n., *Avicularia caei* sp. n., and *Avicularia hirschii* by lacking well-developed process on cymbium (Fig. 305). They can be distinguished from *Avicularia minatrix* by color pattern of a single red (Fig. 297) or dark longitudinal stripe on the abdomen dorsum.

**Figure 256.**
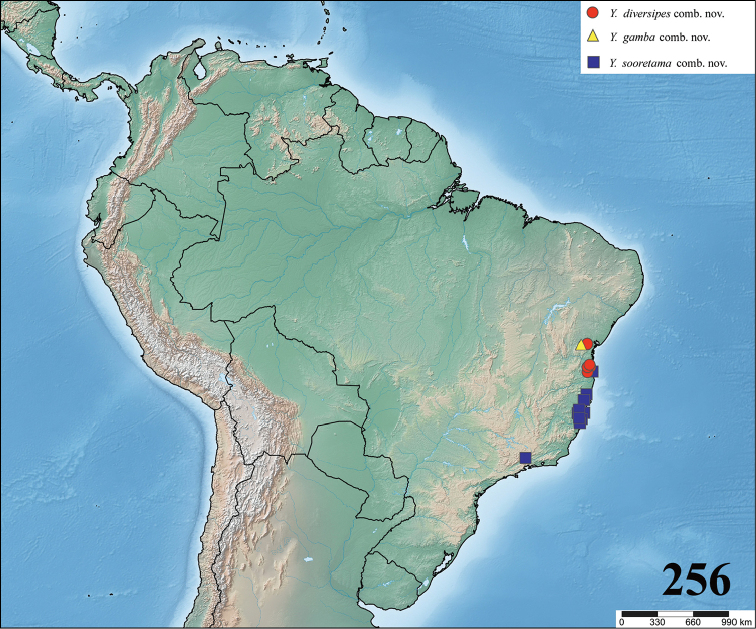
Map showing records of *Ybyrapora* gen. n.

##### Etymology.

From the ancient Tupi, the classic indigenous language from Brazil, “ybyrá”, meaning “tree”; and “pora”, meaning “those that lives in”. Thus, Ybyrapora means “those that lives in trees”, regarding the arboreal habit of the three species of the genus.

##### Description.

**Figures 257–259.**
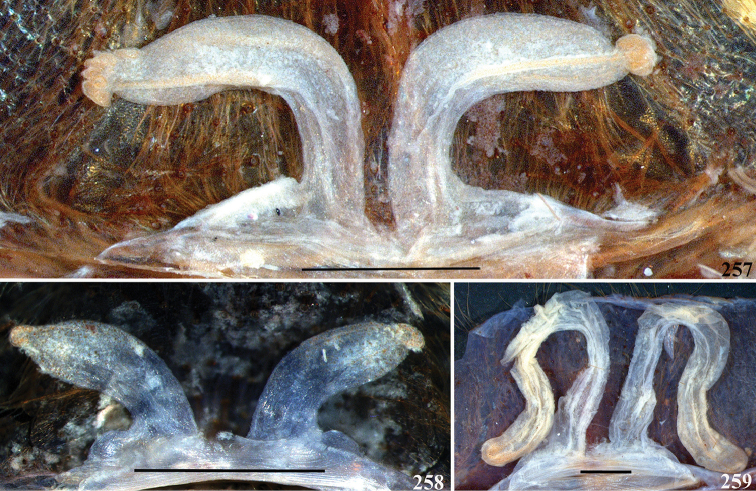
*Ybyrapora* gen. n., spermathecae variation. **257** *Ybyrapora sooretama* (Bertani & Fukushima, 2009) comb. n., Reserva Biológica de Sooretama, state of Espírito Santo, Brazil, paratype (MNRJ 12930) **258** *Ybyrapora gamba* (Bertani & Fukushima, 2009) comb. n., RPPN Jequitibá, Elísio Medrado, state of Bahia, Brazil, paratype (MZUSP 31116) **259** *Ybyrapora diversipes* (C. L. Koch, 1842) comb. n., Ilhéus, state of Bahia, Brazil (IBSP 11754). Scale bars = 1 mm.

Carapace slightly longer than wide, cephalic region slightly raised. Cephalic and thoracic striae inconspicuous or conspicuous. Fovea shallow and straight. Chelicera without rastelum. Eye tubercle distinct, slightly raised, wider than long. Anterior row of eyes procurve, posterior slightly recurve or recurve. Clypeus absent or narrow. Labium subquadrate, wider than long, with 35–130 cuspules on anterior third center, spaced by more than one diameter from each other on anterior third. Maxillary lyra absent. Maxilla subretangular, anterior lobe distinctly produced into conical process, inner angle bearing 80–200 cuspules. Sternum longer than wide, posterior angle acute, not separating coxae IV. Sigilla anterior not evident, middle fusiform, posterior ellipsoidal, one to 1.5 diameter from margin. Leg formula: I=IV II III (most species) or IV I II III (*Ybyrapora sooretama* comb. n. female). Clavate trichobothria on the distal 2/3 to 1/2 of tarsi I–IV. Tarsi I–IV fully scopulate, IV divided or not by band of setae. Metatarsi I–II 4/5 to fully scopulate, III 2/3, IV 2/3 to 1/3 distal scopulate. Metatarsi IV divided by row of setae. Scopulae of tarsi and metatarsi I–II extended very laterally giving them spatulate appearance. Femora IV without retrolateral scopulae. Stridulatory setae absent. Spiniform setae absent. ITC absent; STC without denticles. Posterior lateral spinneret distally elongating, digitiform. Typical type II urticating setae on dorsal abdomen. Tibia I with discrete elevation covered by a cluster of setae in apical portion, on prolateral side (*Ybyrapora diversipes* comb. n.) (Fig. 282) or lacking modifications (other species) (Fig. 266). Tibial apophysis on leg II absent. Globous bulb with small subtegulum and developed prominence on tegulum (*Ybyrapora sooretama* comb. n.) (Fig. 262) or without prominence (other species) (Figs 270, 278). Embolus not flattened, lacking keels, about 3 to 3.5 times tegulum’s length (*Ybyrapora sooretama* comb. n. and *Ybyrapora gamba* comb. n.) (Figs 261, 269) or more than 4 times tegulum’s length (*Ybyrapora diversipes* comb. n.) (Fig. 277). Embolus medial portion and tegulum’s margin forming an obtuse angle in retrolateral view (*Ybyrapora sooretama* comb. n. and *Ybyrapora gamba* comb. n.) (Figs 261, 269) or forming very acute angle in retrolateral view (*Ybyrapora diversipes* comb. n.) (Fig. 277). Embolus proximal part very curved in frontal view (*Ybyrapora sooretama* comb. n. and *Ybyrapora diversipes* comb. n.) (Figs 262, 278) or slightly curved (*Ybyrapora gamba* comb. n.) (Fig. 270). Embolus with thin distal width, tapering distally (*Ybyrapora diversipes* comb. n. and *Ybyrapora gamba* comb. n.) (Figs 279, 271) or narrowing abruptly (*Ybyrapora sooretama* comb. n.) (Fig. 263). Cymbium subtriangular with subequal lobes, having well- developed rounded process on retrolateral lobe, bearing thick setae (*Ybyrapora diversipes* comb. n.) (Fig. 280) or lacking any process (*Ybyrapora sooretama* comb. n. and *Ybyrapora gamba* comb. n.) (Figs 264, 272). Spermathecae with accentuated outwards curvature medially, virtually not sclerotized, completely separated, not-twisted and with midwidth as wide as its base width (Figs 257–259). Spermathecae long (*Ybyrapora sooretama* comb. n. and *Ybyrapora gamba* comb. n.) (Figs 257–258) or very long (*Ybyrapora diversipes* comb. n.) (Fig. 259), with multilobular apex (*Ybyrapora sooretama* comb. n.) (Fig. 257) or lacking lobes (other species) (Figs 258–259). Drastic ontogenetic changes on abdominal color pattern. Immatures with abdomen with a central longitudinal reddish stripe inside a dark area with zigzag borders connected to transverse dark stripes (Fig. 295). Males with central longitudinal stripe on dorsal abdomen (*Ybyrapora sooretama* comb. n. and *Ybyrapora gamba* comb. n.) or lacking stripes (*Ybyrapora diversipes* comb. n.).

##### Type species.

*Avicularia sooretama* Bertani & Fukushima, 2009, here designated.

##### Species included.

*Ybyrapora sooretama* (Bertani & Fukushima, 2009) comb. n., *Ybyrapora diversipes* (C. L. Koch, 1842) comb. n. and *Ybyrapora gamba* (Bertani & Fukushima, 2009) comb. n.

##### Distribution and habitat.

Brazil, in Atlantic rainforest from Bahia to southern Rio de Janeiro (see Bertani and Fukushima 2009, appendix A, fig. 17) (Fig. 256).

##### Key for *Ybyrapora* gen. n. species

**Males**

**Table:** 

1	Embolus length more than 4 times tegulum’s length (Fig. 277)	***Ybyrapora diversipes* comb. n.**
–	Embolus length from 3.0 to 3.5 times tegulum’s length (Figs 261, 269)	**2**
2	Abdomen dorsum with dark central longitudinal stripe, tegulum with developed prominence, embolus with basal part very curved in frontal view (Fig. 262)	***Ybyrapora sooretama* comb. n.**
–	Abdomen dorsum with red central longitudinal stripe (Fig. 297), tegulum lacking prominence (Fig. 270), embolus with basal part slightly curved in frontal view (Fig. 270)	***Ybyrapora gamba* comb. n.**

**Females**

**Table:** 

1	Spermathecae extremely long, with distal apex reaching base (Fig. 259)	***Ybyrapora diversipes* comb. n.**
–	Short spermathecae, distal apex not reaching base	**2**
2	Spermathecae with multilobular apex (Fig. 257)	***Ybyrapora sooretama* comb. n.**
–	Spermathecae lacking lobes on apex (Fig. 258)	***Ybyrapora gamba* comb. n.**

#### Ybyrapora sooretama

Taxon classificationAnimaliaORDOFAMILIA

(Bertani & Fukushima, 2009) comb. n.

Figs 256 , 257 , 260–267 , 298 , 308

Avicularia sooretama Bertani & Fukushima, 2009: 29, figs 5–8, 17, Appendix II, figs B1–B4 (holotype male, Brazil, state of Espírito Santo, Reserva Biológica de Sooretama [18°59'S, 40°07'W], at night, AMNRJ, 18 April 2006, MNRJ 18435 and paratype female, Brazil, state of Espírito Santo, Pinheiros, Reserva Biológica Córrego do Veado (18°37'0.16"S, 40°14'1.60"W), 71 m a.s.l., AMNRJ, 22 October 2005, MNRJ 12930, examined); Bertani 2102: 5, 79; 80, 88; World Spider Catalog 2016.

##### Diagnosis

**(amended from Bertani and Fukushima 2009)**. Females of *Ybyrapora sooretama* comb. n. resemble those of *Ybyrapora gamba* comb. n. by elongated spermathecae with accentuated outwards curvature medially and by retaining vestiges of characteristic juvenile color pattern on abdomen. They differ from those of *Ybyrapora gamba* comb. n. by spermathecae bearing multilobular apex (Fig. 257). Males resemble those of *Ybyrapora gamba* comb. n. by embolus length 3.0 to 3.5 times tegulum’s length and lacking a very strong curvature in frontal view (Fig. 262). They differ from those of *Ybyrapora gamba* comb. n. by developed prominence on tegulum (Fig. 262) and by central longitudinal black stripe on dorsal abdomen.

##### Description, color pattern ontogeny, distribution and natural history.

See Bertani and Fukushima (2009) (Fig. 256).

##### Complementary description.

**Figures 260–267.**
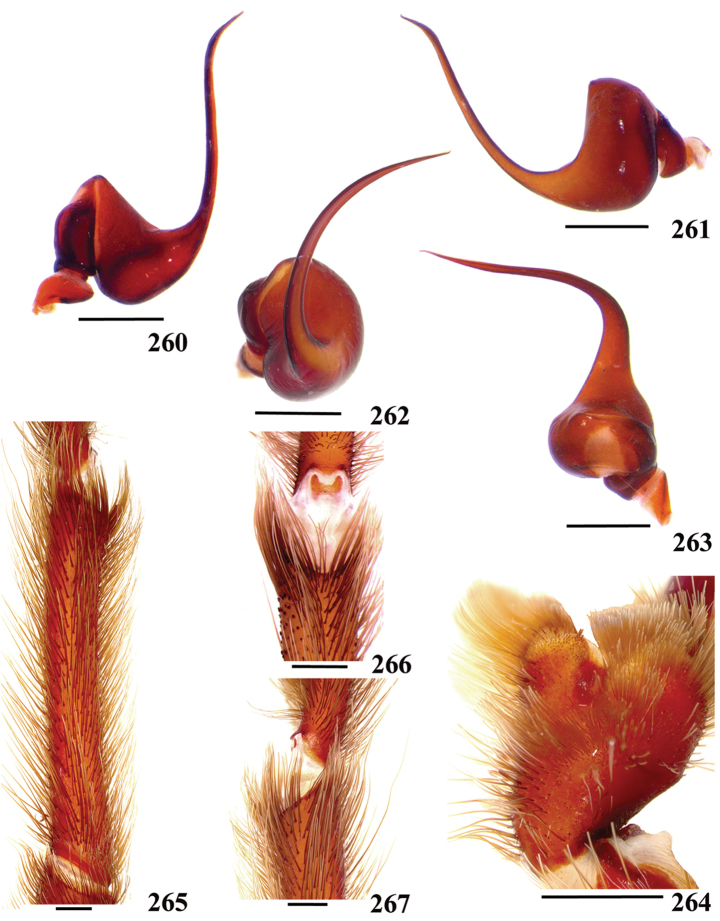
*Ybyrapora sooretama* (Bertani & Fukushima, 2009) comb. n., male holotype (MNRJ 18435). **260–263** right palpal bulb (mirrored) **260** prolateral **261** retrolateral **262** frontal **263** dorsal **264** right cymbium, dorsal (mirrored) **265–267** left tibia I **265** prolateral **266** ventral **267** retrolateral. Scale bars = 1 mm.

Male: Palp (Figs 260–263): globous bulb with small subtegulum and developed prominence on tegulum. Embolus: not flattened, lacking keels, 3.15 long in retrolateral view, about 3.5 times tegulum’s length. Medial portion and tegulum’s margin form an obtuse angle in retrolateral view. Proximal part very curved in frontal view; thin distal width, abruptly narrowing distally; basal, middle, and distal width of 0.63, 0.13, 0.03, respectively. Tegulum: 1.61 long, 0.94 high. Cymbium subtriangular with subequal lobes, lacking process on retrolateral lobe (Fig. 264).

Tibial apophysis in leg I absent (Figs 265–267).

Type II urticating setae: 0.633–0.681 long; 0.012–0.016 wide in male; 0.422–0.490 long; 0.009–0.012 wide in female.

Female: Spermathecae (Fig. 257): two completely separated, not-twisted, long spermathecae, with multilobular apex and accentuated outwards curvature medially. Spermatheca midwidth as wide as its base width, virtually non-sclerotized.

#### Ybyrapora gamba

Taxon classificationAnimaliaORDOFAMILIA

(Bertani & Fukushima, 2009) comb. n.

Figs 256 , 258 , 268–275 , 295–297

Avicularia gamba Bertani & Fukushima, 2009: 32, figs 9–12, 16–17, Appendix III, figs C1–C6 (holotype male, Brazil, state of Bahia, Elísio Medrado, RPPN Jequitibá, (12°52'3.20"S, 39°28'9.09"W), R. Bertani, C. S. Fukushima and R. H. Nagahama col., 7 October 2007, collected at night, found immature inside a retreat made with silk and leaves, matured in captivity on June 2009, MZUSP 31115, and paratype, female, Brazil, state of Bahia, Elísio Medrado, RPPN Jequitibá, (12°52'3.20"S, 39°28'9.09"W), R. Bertani, C. S. Fukushima and R. H. Nagahama col., 7 October 2007, collected at night, found immature inside a retreat made with silk and leaves, MZUSP 31116, examined); Bertani 2012: 5, 79; 80, 88; World Spider Catalog 2016.

##### Diagnosis

**(amended from Bertani and Fukushima 2009)**. Females of *Ybyrapora gamba* comb. n. resemble those of *Ybyrapora sooretama* comb. n. by elongated spermathecae with accentuated outwards curvature medially (Fig. 258) and by retaining vestiges of the characteristic juvenile color pattern on abdomen (Fig. 298). They differ from those of *Ybyrapora sooretama* comb. n. by spermathecae lacking multilobular apex (Fig. 258). Males resemble those of *Ybyrapora sooretama* comb. n. by embolus length 3.0 to 3.5 times tegulum’s length (Fig. 269) and by lacking strong curvature in frontal view (Fig. 270). They differ from those of *Ybyrapora sooretama* comb. n. by lacking prominence on tegulum (Fig. 270) and by having central longitudinal red stripe on dorsal abdomen (Fig. 297).

##### Diagnosis, material examined, description, color pattern ontogeny, distribution and natural history.

See Bertani and Fukushima (2009) (Fig. 256).

##### Complementary description.

**Figures 268–275.**
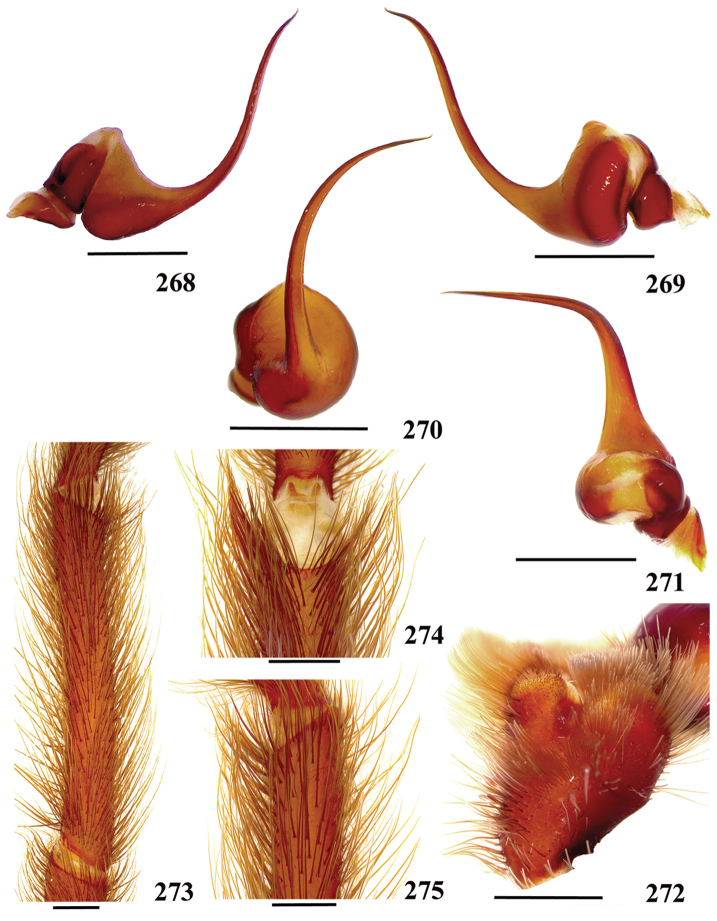
*Ybyrapora gamba* (Bertani & Fukushima, 2009) comb. n., male holotype (MZUSP 31115). **268–271** left palpal bulb **268** prolateral **269** retrolateral **270** frontal **271** dorsal **272** left cymbium, dorsal **273–275** left tibia leg I **273** prolateral **274** ventral **275** retrolateral. Scale bars = 1 mm.

Male: Palp (Figs 268–271): globous bulb with small subtegulum lacking prominence on tegulum. Embolus: not flattened, lacking keels, 2.34 long in retrolateral view, about 3.5 times tegulum’s length. Medial portion and tegulum’s margin form an obtuse angle in retrolateral view. Proximal part slightly curved in frontal view; thin distal width, tapering distally; basal, middle, and distal width of 0.44, 0.11, 0.03, respectively. Tegulum: 1.03 long, 0.59 high. Cymbium subtriangular with subequal lobes, lacking process on retrolateral lobe (Fig. 272).

Tibial apophysis in leg I absent (Figs 273–275).

Type II urticating setae: 0.605–0.750 long; 0.012–0.017 wide in male; 0.301–0.352 long; 0.006–0.009 wide in female (ecdise).

Female: Spermathecae (Fig. 258): two completely separated, not-twisted long spermathecae lacking lobes and accentuated outwards curvature medially. Spermatheca midwidth as wide as its base width, virtually not sclerotized.

#### Ybyrapora diversipes

Taxon classificationAnimaliaORDOFAMILIA

(C. L. Koch, 1842) comb. n.

Figs 256 , 259 , 276–283 , 319

Mygale diversipes C. L. Koch, 1841: 65, pl. CCCX, fig. 731 (lectotype female, Brazil, Bahia, Freir. leg., ZMB 2943, examined).
Eurypelma diversipes : C. L. Koch 1850: 73; Simon 1864: 67, 1892: 172; Ausserer 1871: 202; Roewer 1942: 239; Bonnet 1955: 1831; 1957: 2990.
Avicularia diversipes : F. O. Pickard-Cambridge 1896: 744; Bertani and Fukushima 2009: 26 (figs 1–4, 13–15, 17, Appendix I, figs A1–A6); Bertani 2102: 5, 79; 80, 88; World Spider Catalog 2016.

##### Diagnosis

**(amended from Bertani and Fukushima 2009)**. Females differ from those of *Ybyrapora sooretama* comb. n. and *Ybyrapora gamba* comb. n. by presenting very long, strongly curved outwards spermathecae with its distal portion almost reaching the spermathecae base (Fig. 259). Males differ from those of *Ybyrapora gamba* sp. comb. n. and *Ybyrapora sooretama* comb. n. by having embolus more than four times tegulum’s length, with strong curvature in frontal view (Fig. 278) and cymbium with well-developed process bearing thick setae on retrolateral lobe (Fig. 280).

**Figures 276–283.**
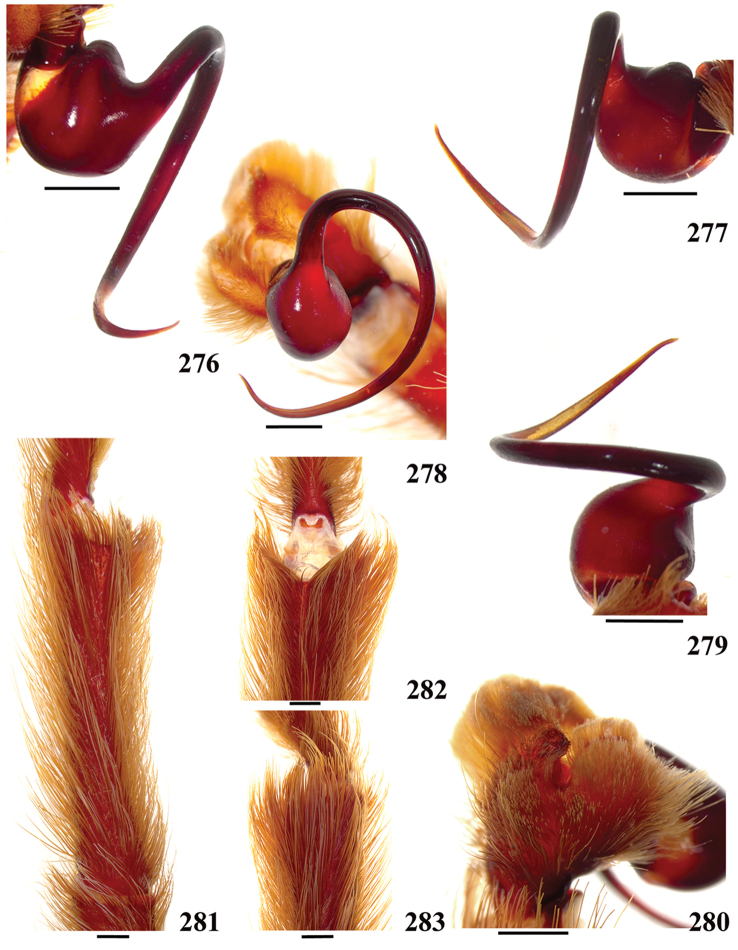
*Ybyrapora diversipes* (C. L. Koch, 1842) comb. n., male (IBSP 119271). **276–279** left palpal bulb **276** prolateral **277** retrolateral **278** frontal **279** dorsal **280** left cymbium, dorsal **281–283** left tibia I **281** prolateral **282** ventral **283** retrolateral. Scale bars = 1 mm.

##### Material examined.

Female, Brazil, state of Bahia, Ilhéus, CEPLAC [14°46'S, 39°13'W], R. Bertani & G. Puorto col., March 1991 (IBSP 11754); male, same locality, collectors and date (IBSP 119271 ref. 64.583).

##### Diagnosis, other material examined, description, color pattern ontogeny, distribution and natural history.

See Bertani and Fukushima (2009).

##### Complementary description.

Male: Palp (Figs 276–279): globous bulb with small subtegulum, lacking prominence on tegulum. Embolus: not flattened, lacking keels, 7.76 long in retrolateral view, about 7 times tegulum’s length. Medial portion and tegulum’s margin form very acute angle in retrolateral view. Proximal part very curved in frontal view; thin distal width, tapering distally; basal, middle, and distal width of 1.05, 0.35, 0.08 respectively. Tegulum: 1.89 long, 1.08 high. (Fig. 319). Cymbium subtriangular with subequal lobes, with a well-developed rounded process on retrolateral lobe, bearing thick setae (Fig. 280).

Tibia I with discrete elevation covered by a cluster of setae in apical portion, on prolateral side (Figs 281–283).

Type II urticating setae: 0.791–0.860 long; 0.017–0.020 wide in male; 0.427–0.520 long; 0.012–0.016 wide in female.

Female: Spermathecae (Fig. 259): two completely separated, not-twisted very long spermathecae, with walls lacking lobes and accentuated outwards curvature medially. Spermatheca midwidth as wide as its base width, virtually non-sclerotized.

#### Antillena

Taxon classificationAnimaliaORDOFAMILIA

Bertani, Huff & Fukushima gen. n.

http://zoobank.org/D95B044F-815A-48F1-85D7-37C1669F86B3

Figs 226 , 284 , 285–292 , 293–294

Avicularia rickwesti Bertani & Huff, 2013: 333, figs 2–19 (holotype female, Dominican Republic, Pedernales Province, Parque Nacional Jaragua, track into park (unmarked) between Manuel Goya (Manuell Goa [*sic*]) and Oviedo (17°48'41.5"N, 71°26'35.9"W), 83.3 m a.s.l., 09 July 2004, J. Huff and E. S. Volschenk leg., (collecting permit #01496), AMNH, and paratype female, Dominican Republic, Independencia Province: Parque Nacional Sierra de Baoruco, Rabo de Gato (18°18'39.1"N, 71°34'54.4"W), 408 m a.s.l., 10 July 2004, J. Huff & E. S. Volschenk leg., collection permit #01496, AMNH, examined); Kaderka 2016: 121, figs 2–11; World Spider Catalog 2016.

##### Etymology.

Named after the type locality, the Antilles, where Dominican Republic is located, and is considered feminine in gender.

##### Type species.

*Avicularia rickwesti* Bertani & Huff 2013, by monotypy.

##### Species included.

*Antillena rickwesti* (Bertani & Huff 2013) comb. n.

##### Diagnosis.

**Figure 284.**
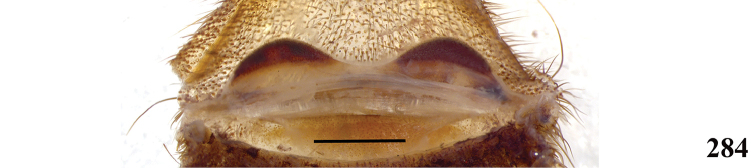
*Antillena rickwesti* (Bertani & Huff, 2013) comb. n., Dominican Republic (AMNH), spermathecae. Scale bars = 1 mm.

**Figures 285–292.**
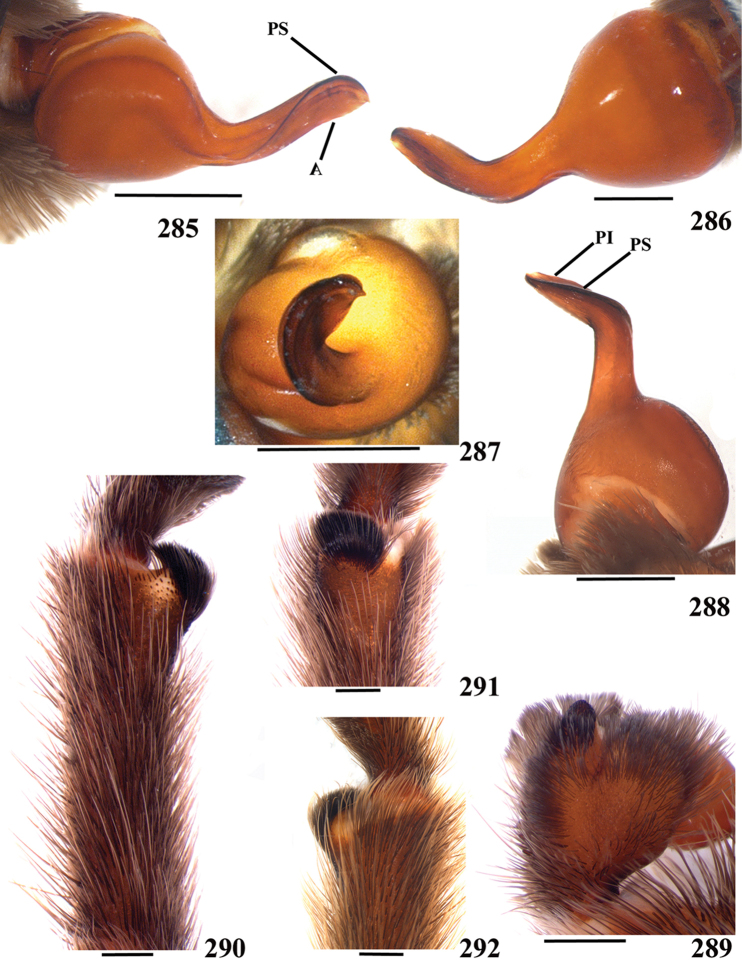
*Antillena rickwesti* (Bertani & Huff, 2013) comb. n., male (AMNH). **285–288** left palpal bulb **285** prolateral **286** retrolateral **287** frontal **288** dorsal **289** left cymbium, dorsal **290–292** left tibial apophysis of leg I **290** prolateral **291** ventral **292** retrolateral. Scale bars = 1 mm.

Female differs from those of other Aviculariinae species by having two very short and broad spermathecae, with distal half strongly sclerotized (Fig. 284). Additionally, they can be distinguished by spiniform setae in prolateral and/or retrolateral side of coxae I–IV. Males can be distinguished by the very flattened embolus with keels (Figs 285–288) as well as abdominal leaf pattern (Fig. 294).

##### Description.

see species description.

##### Material examined.

1 male, Dominican Republic, Pedernales Province, Jaragua National Park, Los Tres Charcos, road to Fondo Paradi (17°48'7.45"N, 71°26'5.41"W), R. C. West and J. Huff col., 20 February 2012, matured in captivity 10 April 2014 (AMNH).

##### Additional material.

DOMINICAN REPUBLIC: *Pedernales*: 5 km south of Manuel Goya [17°52'N, 71°29'W], 1 female, R. C. West col., 20 February 2012, in silk retreat of scrub tree (AMNH RW01); Jaragua National Park, Los Tres Charcos, road to Fondo Paradi (17°48'7.45"N, 71°26'5.41"W), 1 female, R. C. West and J. Huff col., 20 February 2012 (AMNH).

**Figures 293–294.**
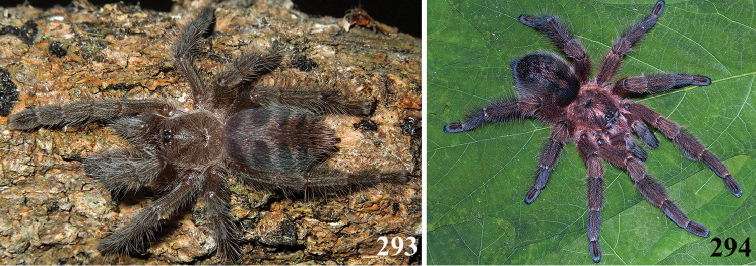
*Antillena rickwesti* (Bertani & Huff, 2013) comb. n., habitus. **293** immature **294** male. Photos: **293** A. Tosto; **294** R. C. West.

**Figures 295–298.**
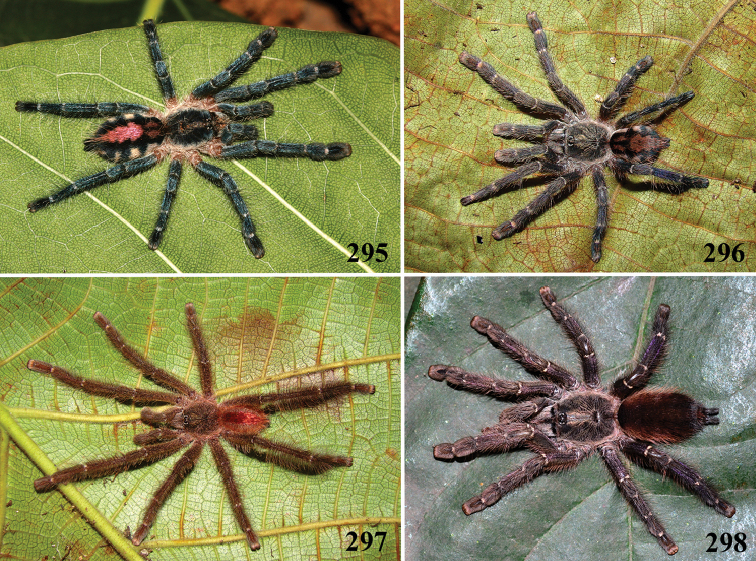
Dorsal abdominal patterns of *Ybyrapora* gen n. **295–297** *Ybyrapora gamba* (Bertani & Fukushima, 2009) comb. n. **295** immature **296** adult female **297** male **298** *Ybyrapora sooretama* (Bertani & Fukushima, 2009) comb. n., adult female. Photos: C. S. Fukushima.

##### Female.

See Bertani and Huff (2013).

**Figures 299–304.**
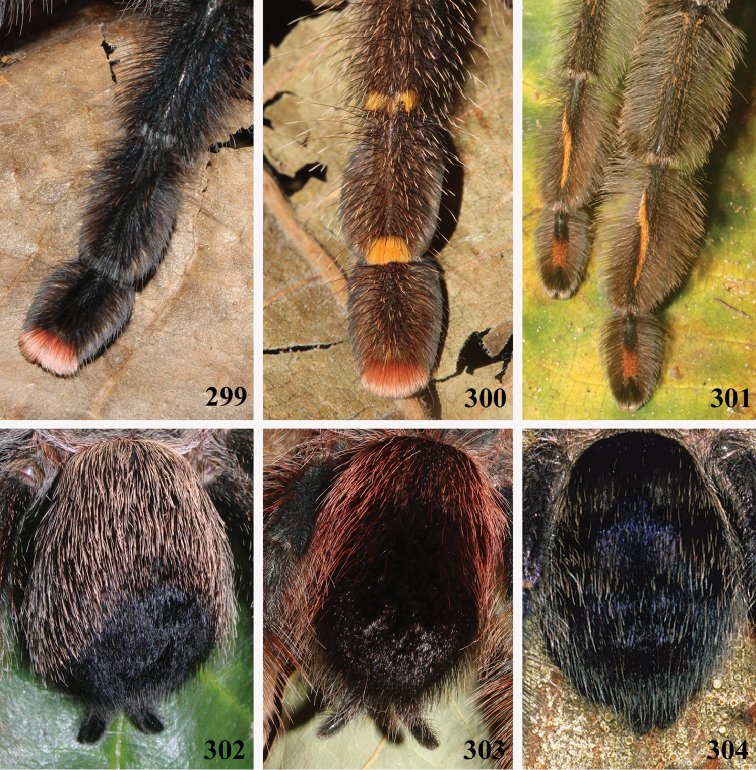
Legs and abdominal characters. **299–300** leg rings coloration on metatarsi and tibiae, and guard-setae coloration on legs **299** *Avicularia avicularia*, female, whitish leg rings and homogeneous coloration along guard-setae on legs **300** *Avicularia rufa*, female, yellow leg rings and guard-setae with darker base and contrasting whitish apex on legs **301** *Psalmopoeus irminia*, male, longer setae laterally projected on legs **302** *Avicularia rufa*, female, abdominal setae covering heterogeneously **303** *Avicularia avicularia*, female, abdominal setae covering heterogeneously **304** *Avicularia purpurea*, female, abdominal setae covering homogeneously. Photos: **299–301** R. Bertani; **302–303** C. S. Fukushima, **304** R. C. West.

**Figures 305–311.**
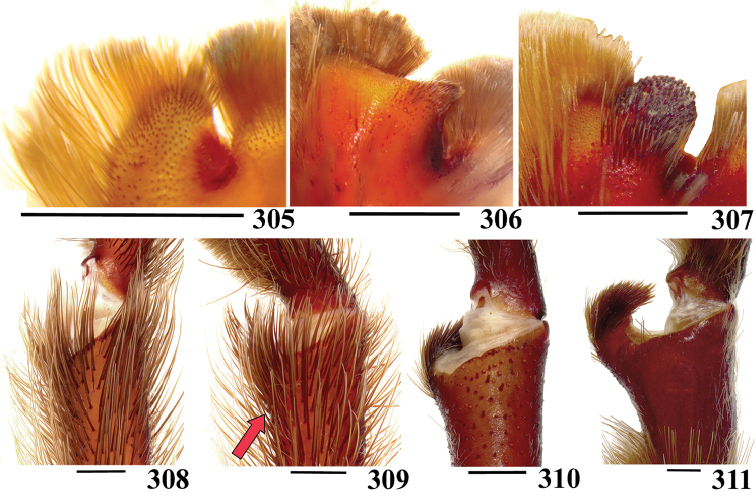
Aviculariine characters. **305–307** cymbium process, dorsal **305** *Ybyrapora gamba* comb. n., holotype (MZUSP 31115), absent **306–307** well-developed process **306** *Caribena versicolor* comb. n. (MNHN–AR 4904), sharp and bearing thin setae **307** *Avicularia juruensis* (CAS 6), rounded and bearing thick setae **308–311** tibia I, retrolateral **308** *Ybyrapora sooretama* comb. n., holotype (MNRJ 18435), lacking modifications **309** *Avicularia hirschii*, paratype (SMF 57125), thicker setae on a discrete elevation **310** *Caribena laeta* comb. n. (AMNH PR), developed branch **311** *Avicularia avicularia* (MNHN–AR 4894), well-developed branch. Scale bars = 1 mm. Arrow indicates discrete elevation.

##### Male.

**Description.** AMNH. Carapace: 10.4 long, 9.9 wide, 2.98 high. Chelicera: 6.7 long. Legs (femur, patella, tibia, metatarsus, tarsus, total): I: 9.9, 5.9, 7.7, 8.2, 4.8, 35.0. II: 9.6, 5.5, 7.6, 7.9, 4.6, 33.8. III: 8.1, 4.6, 6.6, 7.1, 4.1, 30.1. IV: 10.2, 5.2, 8.7, 8.4, 3.8, 36.3. Palp: 6.2, 3.6, 4.6, –, 2.5, 21.1. Midwidths: femora I–IV= 2.1, 1.9, 2.2, 2.1, palp=1.6; patellae I–IV=2.0, 2.0, 1.9, 2.0, palp=1.6; tibiae I–IV=1.8, 1.6, 1.6, 1.9, palp=1.7; metatarsi I–IV=1.1, 1.1, 1.0, 1.1; tarsi I–IV=1.1, 1.1, 1.2, 1.3, palp=1.3. Abdomen 14.9 long, 10.7 wide. Spinnerets: PMS, 0.9 long, 0.3 wide, 0.1 apart; PLS, 1.7 basal, 1.0 middle, 2.0 distal; midwidths 1.1, 0.8, 0.6, respectively.

Carapace: 1.24 times longer than wide; cephalic region raised. Fovea: deep, recurved, 1.6 wide.

Eyes: eye tubercle 0.6 high, 1.8 long, 2.5 wide. Clypeus absent. Anterior eye row procurve, posterior slightly recurve. Eye sizes and interdistances: AME 0.60, ALE 0.43, PME 0.28, PLE 0.30, AME–AME 0.34, AME–ALE 0.32, AME–PME 0.16, ALE–ALE 1.78, ALE–PME 0.65, PME–PME 1.49, PME–PLE 0.07, PLE–PLE 1.94, ALE–PLE 0.54, AME–PLE 0.42.

Maxilla: length to width: 1.76. Cuspules: 128 spread over ventral inner heel. Labium: 1.3 long, 1.8 wide, with 61 cuspules spaced by one diameter from each other on anterior half. Labio-sternal groove shallow, flat, sigilla not evident.

Chelicera: basal segment with 12 teeth, second, fourth and fifth the larger; parallel basal row of six tiny teeth on promargin.

Sternum: 5.5 long, 4.5 wide. Sigilla: anterior pair not evident, the other ellipsoidal, less than half diameter from margin; posterior one time and half the diameter of the middle.

Legs: Formula: I=IV II III. Length leg IV to leg I: 1.04. Clavate trichobothria: 2/3 distal tarsi I–IV. Leg coxae with spiniform setae; absent on leg I and prolateral leg II, poorly developed on retrolateral leg I, prolateral and retrolateral leg III and prolateral leg IV. Scopula: tarsi I–IV fully scopulate. Metatarsi I–II fully scopulate; III 4/5, IV 1/2 distal scopulate. IV divided by a row of setae.

Type II urticating setae: 0.77–0.88 long, 0.014–0.018 wide.

Palp (Figs 285–288): globous bulb with small subtegulum lacking prominence on tegulum. Embolus: flattened, bearing three sharp keels: a prolateral superior on its distal half; a prolateral inferior for full embolus length; and an apical for two-thirds of embolus length. Embolus 1.5 long in retrolateral view. Medial portion and tegulum’s margin form an acute angle in retrolateral view. Proximal part slightly curved in frontal view; thin distal width, tapering distally; basal, middle, and distal width of 0.44, 0.37, 0.18, respectively. Tegulum: 1.31 long, 1.18 wide in retrolateral view. Cymbium subtriangular with subequal lobes. Cymbium with well-developed rounded process on retrolateral lobe, bearing thick setae (Fig. 289).

Tibial apophysis (Figs 290–292): single branch on prolateral leg I, with well-developed base and grouped spiniform setae distally. Male metatarsus I touches retrolaterally tibial apophysis’ setae when folded.

Color pattern (Fig. 294): carapace dark brown covered with abundant light brown short body setae especially on its margins. Legs, palps and chelicerae dorsally brown covered with light brown short body setae. Legs and palps with brown long guard-setae. Leg rings on distal femora, tibiae and metatarsi whitish. Abdomen dorsally dark brown with light pattern. Coxae, labium, sternum, maxillae and legs ventrally brown with light brown setae. Tibiae, metatarsi and tarsi ventrally darker. Abdomen ventrally greyish, distal third darker. Living specimens shows intense metallic pinkish short body setae over carapace, dorsal legs and palps. Abdomen is dorsally black with lighter leaf pattern.

**Figures 312–315.**
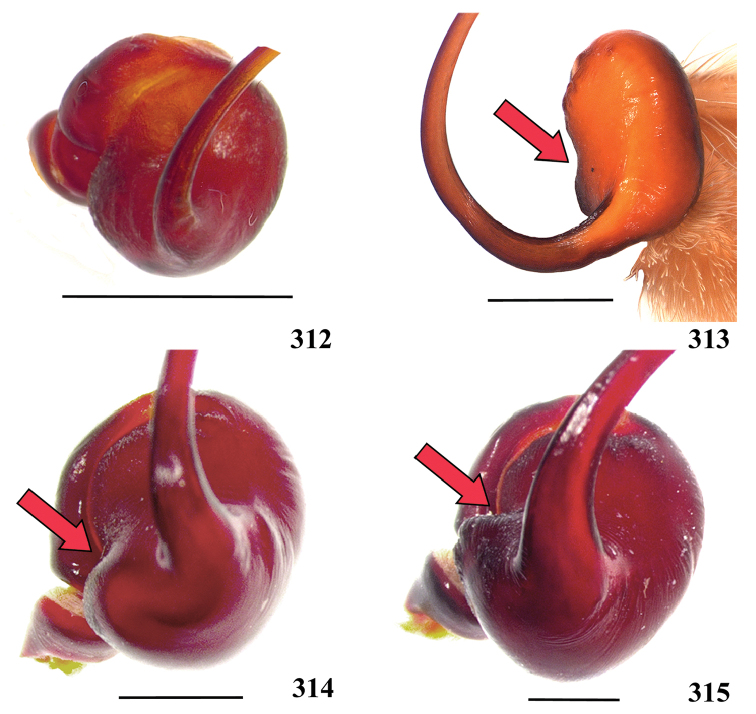
Male palpal bulb prominence in *Avicularia*, frontal. **312** *Avicularia minatrix* (MZUSP 70949), absent **313** *Avicularia caei* sp. n. holotype (MPEG 015637), weakly-developed **314** *Avicularia avicularia* (MPEG 2534), developed **315** *Avicularia variegata* stat. n. (INPA 4897), well-developed. Scale bars = 1 mm. Arrows indicate groove that forms bulb prominence.

**Figures 316–319.**
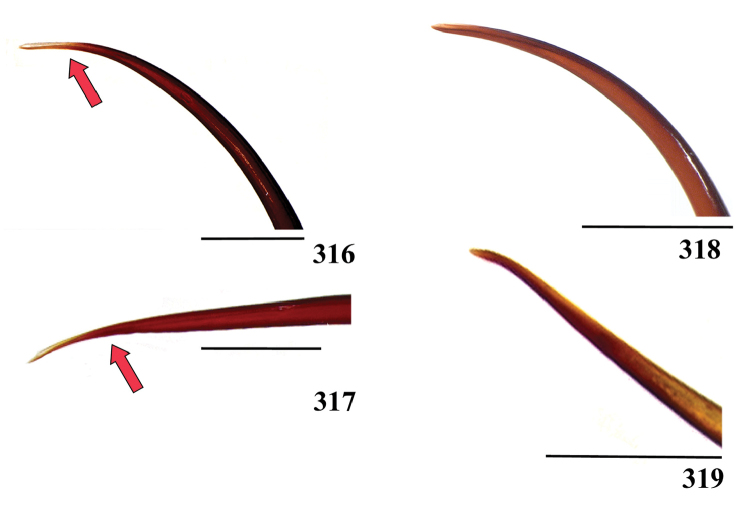
Embolus tip of male palpal bulb. **316–317** tip narrowing abruptly **316** *Avicularia avicularia* (MNRJ 13659A), frontal view **317** *Avicularia variegata* stat. n. (INPA 4897), dorsal view **318–319** tip tapering **318** *Caribena versicolor* comb. n. (MNHN–AR 4904), frontal view **319** *Ybyrapora diversipes* comb. n. (IBSP 119271), dorsal view. Scale bar = 1mm. Arrow indicates point of abrupt narrowing.

##### Color pattern ontogeny.

See Bertani and Huff (2013) (Fig. 293).

**Figure 320–323.**
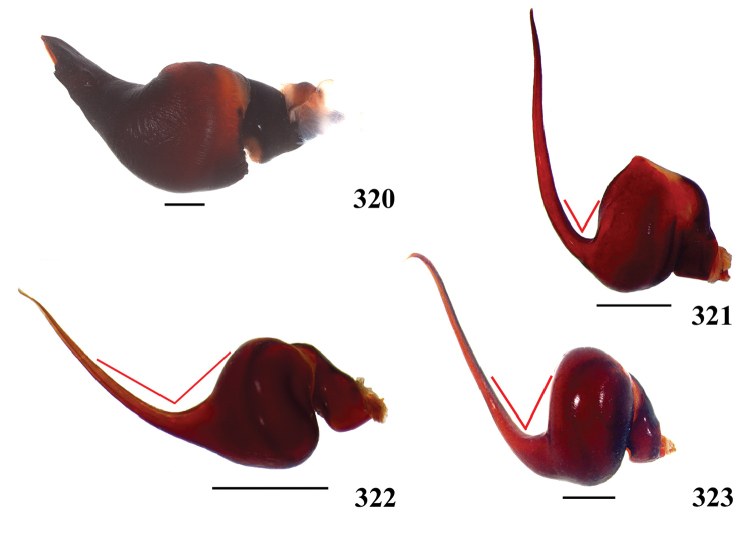
Male palpal bulb characters. **320** *Lasiodora* sp., retrolateral, left palp **321–323** angle between medial portion of embolus and tegulum’s margin in retrolateral view **321** *Pachistopelma bromelicola*, right palp, very acute angle **322** *Iridopelma hirsutum* (IBSP 8077), right palp, obtuse angle **323** *Avicularia avicularia*, right palp, acute angle (MNRJ 13659A). Scale bars = 1 mm. Red bars show angle between tegulum’s margin and embolus medial portion.

##### Distribution.

Dominican Republic (Fig. 226).

##### Natural history.

See Bertani and Huff (2013).

## Cladistics

**Figure 324.**
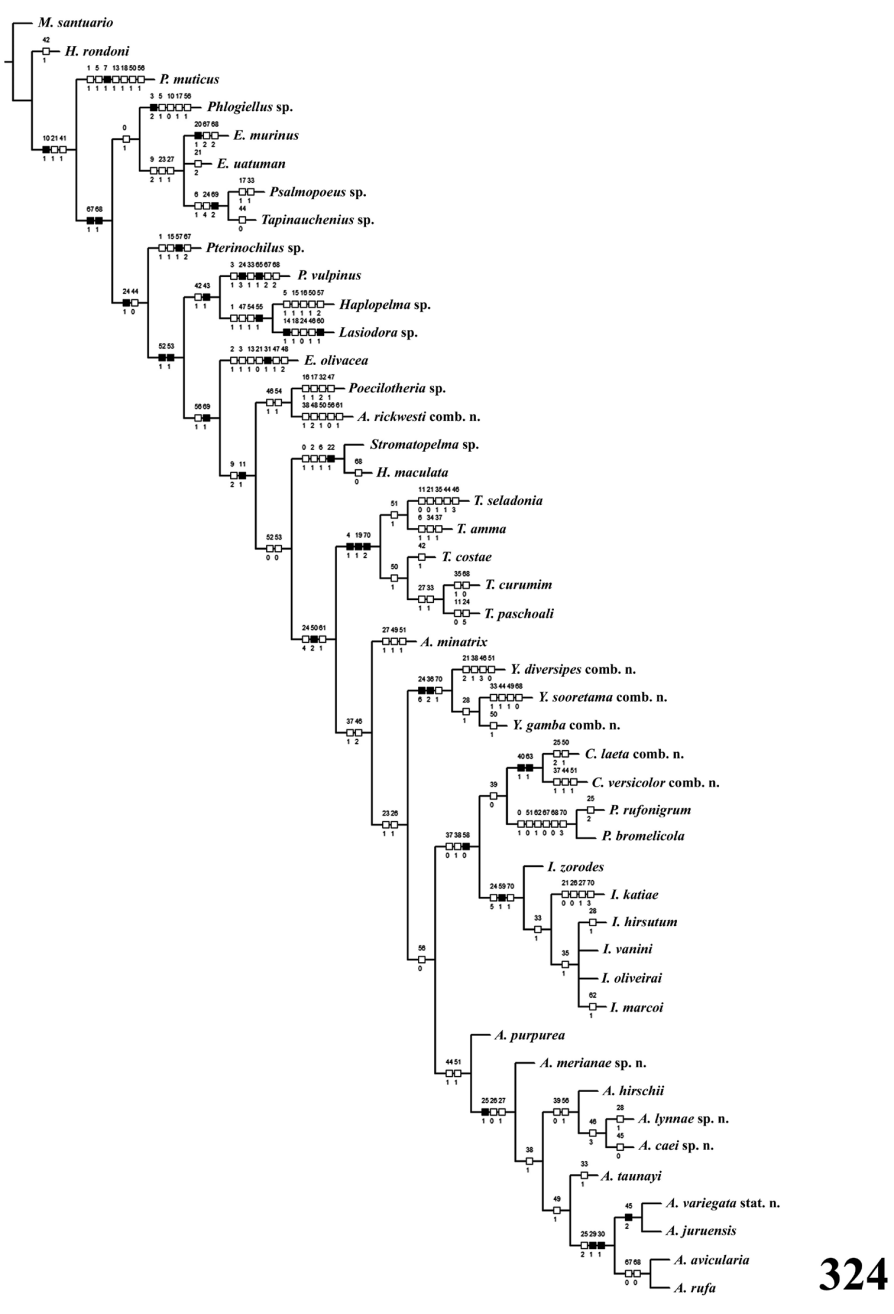
Strict consensus of 6 trees obtained with NONA and all characters as non-additive. Black square = synapomorphy, white square = homoplasy. Length = 229, CI = 41, RI = 67.

Searches using NONA (hold 10000, mult*500, hold/1000) resulted in 6 cladograms and their strict consensus (Nelsen, L= 229, CI = 41, RI= 67) is shown in Figure 324. The consensus cladogram is almost completely resolved. It shows Aviculariinae as non monophyletic, since *Ephebopus* spp., *Psalmopoeus* sp. and *Tapinauchenius* sp. are in a separated clade as the sister group of *Phlogiellus* sp. (Selenocosmiinae). *Antillena rickwesti* comb. n. is the sister group of *Poecilotheria* sp., and both taxa could be considered as aviculariines. The genus *Avicularia* is paraphyletic in this analysis, as *Avicularia minatrix* is recovered apart from the other species and as one of the most basal taxon of Aviculariinae. The *Avicularia* clade is supported by two homoplastic characters, the presence of prominence on tegulum (character 44, state 1) and male palpal bulb with embolus medial portion and tegulum’s margin form an acute angle in retrolateral view (character 51, state 1). The genus is the sister group of (*Iridopelma* (*Pachistopelma* + *Caribena* gen. n.)).

With Piwe, we found one or two cladograms with each concavity used (Table 3). Use of implied weights and different concavities resulted in more than one cladogram topology. The topologies obtained using concavities 1 (Suppl. material 1: concavity 1) and 2 (Suppl. material 2: concavity 2) are very similar, differing only in the relationship inside *Typhochlaena* genus; the one obtained with concavities 3 (Suppl. material 3: concavity 3) and 4 (Suppl. material 4: concavity 4) are very similar, differing only in position of *Poecilotheria* sp. and *Avicularia rickwesti* comb. n. The topologies obtained with concavities 5 (Suppl. material 5: concavity 5) and 6 are very distinct from the others.

**Table 3.** Length, fit and number of cladograms obtained using implied weights and different concavities on Piwe.

Concavity	Number of cladograms	Fit	Length
1	1	338.7	238
2	2	400.4	238
3	1	439.7	236
4	1	466.9	234
5	1	487.1	232
6	1	503.4	230

In topologies obtained using concavities 1 and 2, Aviculariinae is recovered as paraphyletic since *Avicularia rickwesti* comb. n. is in a clade with *Poecilotheria* sp., *Haplopelma* sp. and *Lasiodora* sp., genera belonging to three different theraphosid subfamilies. The clade (*Ephebopus* (*Psalmopoeus* + *Tapinauchenius*)) was not recovered as basal group, but in a more derived group of Aviculariinae.

The genus *Avicularia* is recovered as polyphyletic, with *Avicularia minatrix* in a basal position as the sister group of most Aviculariinae genera. *Avicularia purpurea* and *Avicularia merianae* sp. n. were recovered in a distinct clade in the middle of aviculariine, and the clade with *Avicularia hirschii*, *Avicularia lynnae* sp. n. and *Avicularia caei* sp. n. is in a trichotomy with *Caribena* gen. n. and *Pachistopelma* + *Iridopelma marcoi*.

The topologies obtained using concavities 3 and 4 are very similar, differing in the position of *Avicularia rickwesti* comb. n. and *Poeciolotheria* sp. In topology obtained with concavity 4, they form a clade inside Aviculariinae; in concavity 3, *Avicularia rickwesti* comb. n. is in a clade with some genera of the outgroup, making Aviculariinae paraphyletic. Their topologies resemble those obtained with concavities 1 and 2, differing mainly in position of the clade *Ephebopus* (*Tapinauchenius* + *Psalmopoeus*), which is basal in Aviculariinae with concavities 3 and 4, and differing in *Avicularia minatrix* position, which is basal in Aviculariinae with concavities 1 and 2.

**Figure 325.**
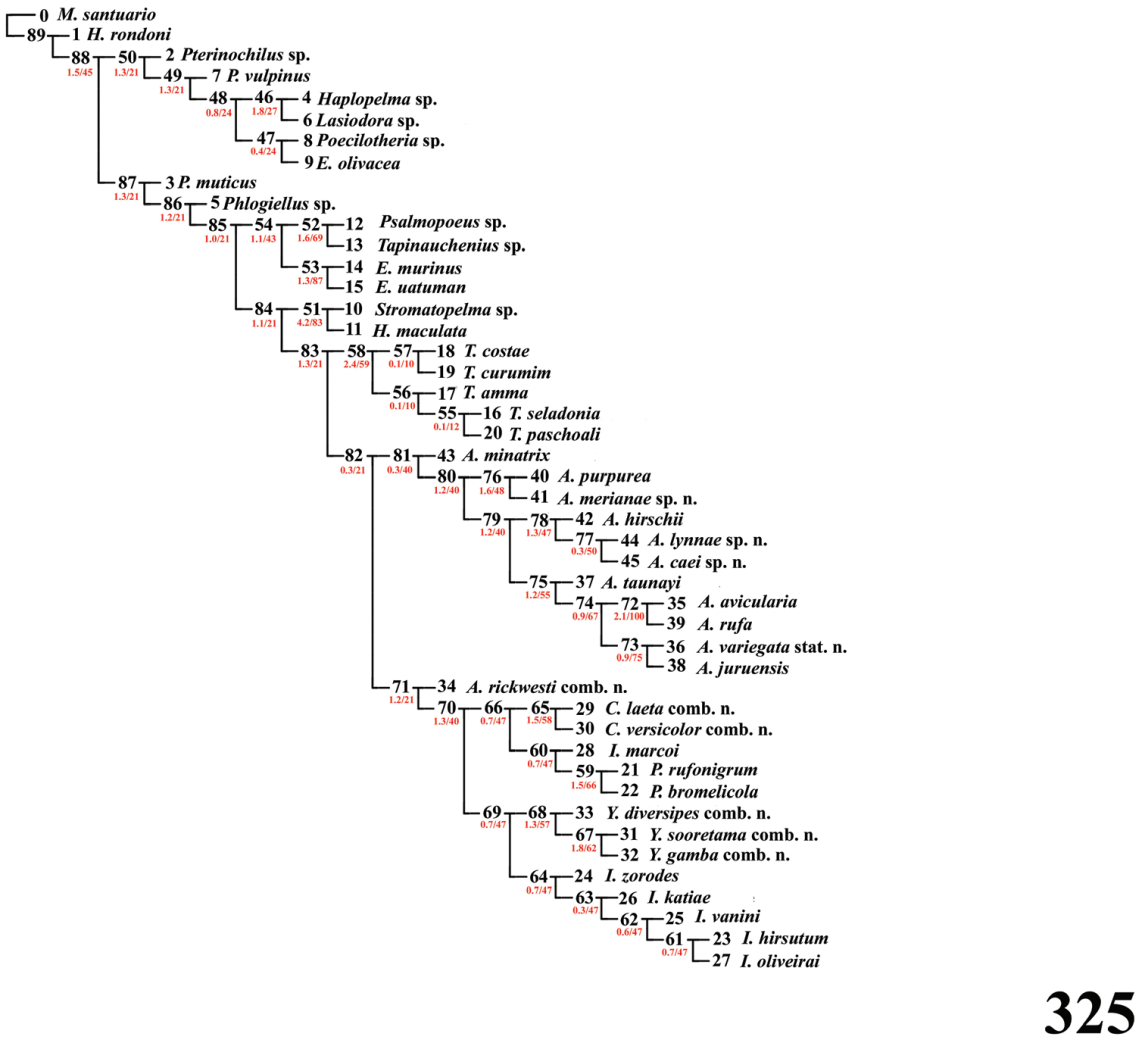
Single tree obtained with Piwe, all characters as non-additive and concavity 6. Fit = 5051.44 and length = 230. Bremer support values (absolute and relative) of each node are in red.

The topology obtained with concavity 5 resembles the topology obtained using concavity 6. It differs mainly in *Typhochlaena* position, which is recovered as a inner group of *Avicularia*, as well as in *Avicularia rickwesti* comb. n. position, which is the sister group of most aviculariine genera.

In agreement with other studies (Ramírez 2003, West et al. 2008, Bertani 2012), the shortest cladogram with highest fit on Piwe was obtained when using concavity 6 (Table 3, Fig. 325) and it is chosen as the preferred cladogram, of which the main discussion is done (Tables 4–5). In some cases, discussion about specific clades can include comments about topologies obtained with different concavities.

In the preferred cladogram, *Avicularia* is monophyletic (node 81 in Fig. 325) and is composed of 11 species, plus *Avicularia glauca* which was not included in the matrix: *Avicularia avicularia*, *Avicularia minatrix*, *Avicularia purpurea*, *Avicularia hirschii*, *Avicularia taunayi*, *Avicularia juruensis*, *Avicularia variegata* stat. n., *Avicularia rufa*, *Avicularia merianae* sp. n., *Avicularia lynnae* sp. n. and *Avicularia caei* sp. n. The sister-group of *Avicularia* is a clade formed by *Antillena* gen. n., *Caribena* gen. n., *Pachistopelma*, *Iridopelma* and *Ybyrapora* gen. n. The characters supporting *Avicularia* clade are juveniles with black tarsi contrasting with other lighter articles (character 27, state 1) (Fig. 44), with a reversion in *Avicularia purpurea*; spermathecae with accentuated outwards curvature medially (character 37, state 1) (Fig. 21) and male palpal bulb with embolus medial portion and tegulum’s margin form an acute angle in retrolateral view (character 51, state 1) (Fig. 323). All these characters are very homoplastic.

The species *Avicularia minatrix* was retrieved as the most basal species in *Avicularia*. *Avicularia purpurea* and *Avicularia merianae* sp. n. are very similar species regarding body dimensions, spermathecae and cymbium morphology as well as geographic distribution area, condition reflected in their close position on the cladogram, apart from most *Avicularia* species (node 76 in fig. 325).

Node 78 (Fig. 325) is composed of *Avicularia hirschii*, *Avicularia lynnae* sp. n. and *Avicularia caei* sp. n. They are very singular species, since they have discrete elevation on prolateral tibia covered by a cluster of setae which clearly cannot be considered a branch (Fig. 309), and process on retrolateral lobe of cymbium covered by thin setae (the homoplasy that supports the node) (Fig. 205). Both characters are distinct from those found in most species of genus. Characters related to genitalia are also singular in these species since males have very long embolus (*Avicularia lynnae* sp. n. and *Avicularia caei* sp. n.) (Figs 202, 211 respectively) and females have twisted spermathecae (*Avicularia hirschii* as in fig. 181). Despite all of these unusual features, this clade was recovered as belonging to *Avicularia* in the chosen cladogram as well as in the cladogram obtained using NONA (fig. 324). However, in the cladograms obtained using concavities 1–4, this clade formed a trichotomy with *Caribena* gen. n. and *Pachistopelma* + *Iridopelma marcoi*. Both *A lynnae* sp. n. and *Avicularia caei* sp. n. females are currently unknown. Even without female characters on matrix, these three species are retrieved together in all cladograms obtained, using equal or different weights. Thus, their close relationship is a robust hypothesis. However, the position of this clade inside *Avicularia* genus can change with the future inclusion on matrix of characters related to females of *A lynnae* sp. n. and *Avicularia caei* sp. n. There is also the possibility that this clade could be retrieved as a new genus.

The clade defined by node 75 (Fig. 325) and having *Avicularia taunayi*, *Avicularia avicularia*, *Avicularia rufa*, *Avicularia juruensis* and *Avicularia variegata* stat. n. is retrieved in all cladograms, with equal or different weights. The clade is supported by a single homoplasy, embolus tip narrowing abruptly (character 49, state 1) (Figs 316–317). Despite its fragile support on the cladogram, this group of species surely seems to be closely related, since bulb, cymbium, tibial apophysis and spermathecae morphology are very similar. Adding to this, corporal size, habitat and geographic distribution area are alike.

The sister group of *Avicularia* is formed of Caribbean and Brazilian Atlantic rainforest aviculariine species. The result obtained indicates three new genera in Aviculariinae (Fig. 325), which were, herein, described. *Caribena* gen. n. (node 65 in fig. 325) is composed of two Caribbean species, *Caribena laeta* comb. n. and *Caribena versicolor* comb. n. The synapomorphies supporting monophyly of the genus (node 65 in Table 4 and in Fig. 325) are the presence of cymbium with retrolateral sharp process (character 40, state 1) (Fig. 306), and presence of slender type II urticating setae, with more than 1 mm in length in females (character 63, state 1) (Fig. 243). This long and slender type II urticating setae also occur in males and immatures of both species. *Caribena* gen. n. is the sister group of *Pachistopelma* + *Iridopelma marcoi* in topology obtained when using concavity 6. In the topologies obtained using concavities 1–4, the clade *Pachistopelma* + *Iridopelma marcoi* forms a group with *Caribena* gen. n. as well as with the clade (*Avicularia hirschii* (*Avicularia lynnae* sp. n. + *Avicularia caei* sp. n.)), resulting in a trichotomy.

**Table 4.** Synapomorphies for cladogram of Fig. 325.

Taxa or Node	Character	Change	Taxa or Node	Character	Change	Taxa or Node	Character	Change
*Holothele rondoni*	33	0 → 1	*Typhochlaena curumim* (cont.)	27	0 → 1	Node 55	11	1 → 0
	42	0 → 1		33	0 → 1	Node 56	51	2 → 1
*Pterinochilus* sp.	1	0 → 1		35	0 → 1	Node 57	50	2 → 1
	15	0 → 1		68	1 → 0	Node 58	4	0 → 1
	57	0 → 1	*Typhochlaena paschoali*	24	4 → 5		19	0 → 1
*Pelinobius muticus*	1	0 → 1		27	0 → 1		70	0 → 2
	7	0 → 1		33	0 → 1	Node 59	0	0 → 1
	13	0 → 1	*Pachistopelma rufonigrum*	25	0 → 2		21	1 → 0
	18	0 → 1	*Iridopelma hirsutum*	28	0 → 1		68	1 → 0
	33	0 → 1	*Iridopelma zorodes*	46	2 → 0	Node 60	62	0 → 1
	50	0 → 1	*Iridopelma katiae*	21	1 → 0	Node 61	46	2 → 0
*Haplopelma* sp.	5	0 → 1		26	1 → 0		67	1 → 2
	15	0 → 1		27	0 → 1	Node 62	35	0 → 1
	16	0 → 1		70	1 → 3	Node 63	33	0 → 1
	50	0 → 1	*Iridopelma marcoi*	33	0 → 1	Node 64	59	0 → 1
	57	0 → 2		35	0 → 1	Node 65	40	0 → 1
*Phlogiellus* sp.	3	0 → 2	*Avicularia minatrix*	49	0 → 1		63	0 → 1
	10	1 → 0	*Avicularia lynnae* sp. n.	28	0 → 1	Node 66	39	1 → 0
	17	0 → 1	*Avicularia caei* sp. n.	45	1 → 0	Node 67	28	0 → 1
*Lasiodora* sp.	14	0 → 1	*Caribena laeta* comb. n.	25	0 → 2		38	1 → 0
	18	0 → 1		50	2 → 1	Node 68	36	0 → 2
	24	1 → 0	*Caribena versicolor* comb. n.	21	1 → 0		37	0 → 1
	32	2 → 1		37	0 → 1		56	0 → 1
	41	1 → 0		44	0 → 1	Node 69	70	0 → 1
	46	0 → 1		51	2 → 1	Node 70	23	0 → 1
	60	0 → 1	*Ybyrapora sooretama* comb. n.	33	0 → 1		26	0 → 1
	64	0 → 1		44	0 → 1		58	1 → 0
*Phrixotrichus vulpinus*	3	0 → 1		49	0 → 1	Node 71	38	0 → 1
	24	1 → 3		68	1 → 0	Node 72	67	1 → 0
	33	0 → 1	*Ybyrapora gamba* comb. n.	50	2 → 1		68	1 → 0
	41	1 → 0	*Ybyrapora diversipes* comb. n.	21	1 → 2	Node 73	45	1 → 2
	64	0 → 1		46	2 → 3	Node 74	29	0 → 1
	65	0 → 1		51	2 → 0		30	0 → 1
	68	1 → 2	*Avicularia rickwesti* comb. n.	46	2 → 1	Node 75	49	0 → 1
*Poecilotheria* sp.	9	1 → 2		48	0 → 2	Node 76	36	0 → 1
	11	0 → 1		50	2 → 1	Node 77	46	2 → 3
	16	0 → 1		52	0 → 1	Node 78	39	1 → 0
	17	0 → 1		53	0 → 1	Node 79	38	0 → 1
	24	1 → 2		54	0 → 1	Node 80	23	0 → 1
	46	0 → 1	*Avicularia taunayi*	33	0 → 1		44	0 → 1
*Encyocratella olivacea*	2	0 → 1	*Avicularia purpurea*	26	0 → 1	Node 81	27	0 → 1
	3	0 → 1		27	1 → 0		37	0 → 1
	13	0 → 1	Node 46	1	0 → 1		51	2 → 1
	21	1 → 0		55	0 → 1	Node 82	46	0 → 2
	31	0 → 1	Node 47	56	0 → 1	Node 83	0	1 → 0
*Heteroscodra maculata*	68	1 → 0		69	3 → 1		50	0 → 2
*Psalmopoeus* sp.	17	0 → 1	Node 48	32	0 → 2		61	0 → 1
	33	0 → 1		47	0 → 1	Node 84	11	0 → 1
*Tapinauchenius* sp.	44	1 → 0		67	2 → 1		44	1 → 0
*Ephebopus murinus*	20	0 → 1	Node 49	52	0 → 1		69	3 → 1
	67	1 → 2		53	0 → 1	Node 85	9	1 → 2
	68	1 → 2	Node 50	24	0 → 1	Node 86	0	0 → 1
*Ephebopus uatuman*	21	1 → 2		67	0 → 2		45	0 → 1
*Typhochlaena seladonia*	21	1 → 0	Node 51	2	0 → 1		67	0 → 1
	26	0 → 1		6	0 → 1	Node 87	12	0 → 1
	35	0 → 1		22	0 → 1		56	0 → 1
*Typhochlaena amma*	06	0 → 1	Node 52	6	0 → 1	Node 88	10	0 → 1
	34	0 → 1		69	3 → 2		21	0 → 1
	37	0 → 1	Node 53	66	0 → 1		41	0 → 1
*Typhochlaena costae*	42	0 → 1	Node 54	23	0 → 1	Node 89	8	0 → 1
*Typhochlaena curumim*	06	0 → 1		27	0 → 1		46	2 → 0
	25	0 → 2		56	1 → 0		69	0 → 3

The other new genus, *Ybyrapora* gen. n. (node 68 in Table 4 and in Fig. 325), includes small aviculariine species of Brazilian Atlantic rainforest, *Ybyrapora diversipes* comb. n., *Ybyrapora sooretama* comb. n. and *Ybyrapora gamba* comb. n. Genus synapomorphies are the presence of virtually non-sclerotized spermathecae (character 36, state 2), with accentuated outwards curvature medially (character 37, state 1, a homoplasy with *Avicularia*) (Figs 257–259), absent or weakly-developed tibial apophysis on leg I (character 56, state 1, a homoplasy with many Aviculariinae taxa) (Figs 308–309, respectively). This genus is retrieved as a monophyletic taxon and is the sister group of *Iridopelma* (except *Iridopelma marcoi*, a special case discussed below) in all trees obtained using implied weight. In NONA analysis, the genus was also recovered as monophyletic, but as the sister group of (*Avicularia* (*Iridopelma* (*Pachistopelma* + *Caribena* gen. n.))).

Since its description, the classification of *Avicularia rickwesti* comb. n. has been a point of discussion. At first, it was included in *Avicularia* genus since its inclusion in other aviculariine genera has no support due to the combination of characters other than spermathecae. The completely aspinose legs, procurve first eye row, digitiform distal article of the posterior lateral spinnerets and presence of urticating setae type II on abdomen dorsum indicate the species should be included in either the *Avicularia* or *Iridopelma* genera (Bertani and Huff 2013). The authors included it in *Avicularia* due to biological features in common with *Avicularia* species and due to the fact that *Iridopelma* only occurs in Brazil (Bertani and Huff 2013). As it was a very distinct species, researchers were waiting to collect a male to confirm or reject the classification of *Avicularia rickwesti* comb. n. In this analysis, this species is always retrieved as a distinct and isolated taxon (node 34 in Table 4 and in Fig. 325), since it has very unusual combination of characteristics such as small embolus length in retrolateral view (character 46, state 1) (Fig. 286), embolus very flattened (character 48, state 2) and slightly curved (character 50, state 1) (Fig. 287), and presence of prolateral superior, prolateral inferior and apical keels on palpal bulb (characters 52, 53 and 54, state 1) (Figs 285, 288). This is the only species in Aviculariinae that has keels in the male palpal bulb. In fact, general morphology of male and female genitalia of *Avicularia rickwesti* comb. n. is unique and very distinct from the other Aviculariinae, but is frequently found in other subfamilies, such as Theraphosinae and Ornithoctoninae, and also in *Poecilotheria* spp. *Antillena rickwesti* comb. n. switches its position in different cladograms. In those obtained with concavities 1 and 2, *Avicularia rickwesti* comb. n. is the sister group of *Poecilotheria* (*Haplopelma* + *Lasiodora*), in a basal position of the cladogram, and out of Aviculariinae clade. Using concavity 3, it is also retrieved in a basal position, but its sister group is ((*Poecilotheria* + *Encyocratela*) + (*Haplopelma* + *Lasiodora*)). In topologies obtained with concavities 4 and 5, *Avicularia rickwesti* comb. n. is retrieved as the sister group of most Aviculariinae genera; in the topology obtained using concavity 4 it is combined with *Poecilotheria*, but it is alone in the topology obtained using concavity 5. However, in the preferred tree obtained using concavity 6 (Fig. 325), *Avicularia rickwesti* comb. n. is retrieved as the sister group of Caribbean genera *Caribena* gen. n. and Brazilian Atlantic rainforest genera *Pachistopelma*, *Ybyrapora* gen. n. and *Iridopelma*. Thus, based on its isolated position on the cladogram, allied to its endemic occurrence in Dominican Republic, we decide to erect a new genus for this species, *Antillena* gen. n.

**Table 5.** Fits, Steps and Extra Steps for characters of cladogram of Fig. 325.

Character	Fit	Steps	Extra Steps	Character	Fit	Steps	Extra Steps
0	7.5	3	2	36	10.0	2	0
1	7.5	3	2	37	6.6	4	3
2	8.5	2	1	38	7.5	3	2
3	8.5	3	1	39	8.5	2	1
4	10.0	1	0	40	10.0	1	0
5	7.5	3	2	41	7.5	3	2
6	6.6	4	3	42	6.6	4	3
7	—	—	—	43	8.5	2	1
8	—	—	—	44	4.6	8	7
9	8.5	3	1	45	7.5	4	2
10	8.5	2	1	46	4.0	12	9
11	7.5	3	2	47	10.0	1	0
12	10.0	1	0	48	7.5	4	2
13	8.5	2	1	49	7.5	3	2
14	—	—	—	50	5.4	7	5
15	8.5	2	1	51	6.0	6	4
16	8.5	2	1	52	8.5	2	1
17	7.5	3	2	53	8.5	2	1
18	8.5	2	1	54	7.5	3	2
19	10.0	1	0	55	10.0	1	0
20	—	—	—	56	5.0	7	6
21	4.6	9	7	57	8.5	3	1
22	10.0	1	0	58	10.0	1	0
23	7.5	3	2	59	10.0	1	0
24	5.4	12	5	60	—	—	—
25	6.0	6	4	61	10.0	1	0
26	6.6	4	3	62	10.0	1	0
27	5.4	6	5	63	10.0	1	0
28	7.5	3	2	64	8.5	2	1
29	10.0	1	0	65	—	—	—
30	10.0	1	0	66	10.0	1	0
31	—	—	—	67	5.4	7	5
32	10.0	2	0	68	4.2	10	8
33	4.0	10	9	69	8.5	4	1
34	7.5	3	2	70	8.5	4	1
35	6.0	5	4				

The species *Iridopelma marcoi* is a problematic taxon. Since many important characters for cladistic analysis such as tibial apophysis, cymbium process and palpal morphology as well as presence of tibial apophysis in tibia II (an usual sinapomorphy for *Iridopelma*) are exclusive to males, it is expected that the species would not be retrieved as part of the genus since its male is unknown. Besides this, *Iridopelma marcoi* female does not have type II urticating setae on dorsal abdomen, a characteristic of most Aviculariinae species. Even though this type of setae is lacking in females, it could be present in males and immatures of this species, like in *Pachistopelma* species. Unfortunately, since males are unknown, this information is lacking in the analysis. On the other hand, as pointed out by Bertani (2012), the spermathecae shape, as well as some somatic characters indicate *Iridopelma marcoi* is, in fact, an *Iridopelma* species and the discovery of the male might confirm its position, solving this question.

The composition of subfamily Aviculariinae (except *Avicularia rickwesti* comb. n., see discussion above) is the same in all trees obtained with equal or different weights, except in the topology obtained with concavity 4, in which *Poecilotheria* sp. is inside Aviculariinae clade. In the chosen cladrogram (Fig. 325), Aviculariinae subfamily is composed of genera *Avicularia, Psalmopoeus*, *Tapinauchenius*, *Ephebopus*, *Stromatopelma*, *Heteroscodra*, *Typhochlaena*, *Pachistopelma*, *Iridopelma*, *Antillena* gen. n., *Ybyrapora* gen. n. and *Caribena* gen. n.

The present analysis differs from the last available Aviculariinae cladogram (Bertani 2012), in which the subfamily was not retrieved as monophyletic. In that cladogram, Aviculariinae was paraphyletic, the clade *Encyocratella* (*Haplopelma* + *Poecilotheria*) was the sister-group of Aviculariinae and the clade with *Psalmopoeus*, *Tapinauchenius* and *Ephebopus* spp. was the sister group of *Pelinobius*+ *Phlogiellus*. The cladogram obtained, herein, agrees with the one presented by West et al. (2008), in which the subfamily is retrieved as a monophyletic clade, supported by a single character—the presence of well-developed scopulae on tarsi and metatarsi very extended laterally, mainly those of legs I and II (Fig. 299). Here this character also supports the subfamily clade (node 85 in Fig. 325). The well-developed scopula also appears independently in *Poecilotheria* sp., but this can be a convergency related to the arboreal habit of specimens of *Poecilotheria* sp. and most aviculariine taxa.

In the analysis carried out here, the sister group of Aviculariinae is *Phogiellus* sp., a selenocosmiine species. They share presence of straight anterior row of eyes (character 0, state 1, with a reversion in a large internal clade and a posterior change to state 1 in *Pachistopelma* spp.) (Fig. 12), a developed prominence on tegulum of male palpal bulb (character 45, state 1) (Fig. 314) and leg IV roughly the same length as leg I (character 67, state 1, with some changes in internal clades). In the cladograms obtained with concavities 1 and 2, the sister group of Aviculariinae is *Phlogiellus* sp. + *Pelinobius muticus*.

However, *Phlogiellus* sp. is a controversial taxon. It is recovered in distinct positions in different cladistic analysis. In Guadanucci (2014), it forms a clade with *Lyrognathus* sp., *Poecilotheria* sp. (other controversial taxon) and *Psalmopoeus* sp., making Aviculariinae paraphyletic. In West et al. (2008), *Phlogiellus* sp. is also the sister group of Aviculariinae, but in Bertani (2012) this taxon is sister group of *Pelinobius muticus* and both form the sister group of the clade ((*Psalmopoeus* + *Tapinauchenius*) *Ephebopus*), making Aviculariinae paraphyletic. The inclusion of other Selenocosmiinae species in the cladistic analysis should confirm if subfamily is the sister group of Aviculariinae as suggested here.

Many clades were recovered in all cladograms despite different approaches used. The clades that can be considered very stable are the genera *Ybyrapora* gen. n., *Caribena* gen. n., *Pachistopelma* and *Iridopelma* (excluding *Iridopelma marcoi*, discussed above), all recovered as monophyletic using both approaches. Other clades as *Typhochlaena*, (*Ephebopus murinus + Ephebopus uatuman* + (*Psalmopoeus* sp. + *Tapinauchenius* sp.)); (*Stromatopelma* sp. + *Heteroscodra* sp.); and (*Avicularia taunayi* ((*Avicularia variegata* st. n. + *Avicularia juruensis*)(*Avicularia avicularia* + *Avicularia rufa*))) were also recovered using equal or implied weights. The clade ((*Avicularia hirschii* (*Avicularia lynnae* sp. n. + *Avicularia caei* sp. n.)) is recovered in all topologies except in that obtained using concavity 5.

The decision of erecting new genera was done in order to preserve the taxonomic stability since these clades are recoverd as monophyletic in all topologies, although their relationship with other clades can change. Besides, the new genera have very distinct morphologic, geographic and ecologic characteristics from *Pachistopelma* and *Iridopelma*, which were recently revised and are well-stablished genera (Bertani 2012). The decision of considering *Caribena* gen. n. species as belonging to *Pachistopelma* (an endemic Brazilian genus which lives exclusively inside bromeliads) and *Ybyrapora* gen. n. species as being *Iridopelma* species, or consider all the species of the three new genera as *Pachistopelma* would cause more taxonomic problems in future analyses if they are recovered as non-related distinct lineages.

Mygalomorph species are one of the most problematic taxa among spiders for reliable species delimitation (Hamilton et al. 2014). Understanding species limits in any taxonomic group is a complex and a researcher’s biased task (Satler et al. 2013), especially when based on morphological traits. It requires examination of as many specimens as possible, familiarity with sisters groups’ morphology and knowledge of distribution and variation of the characters used in taxonomy of the specific group. Mygalomorph spiders are morphologically conserved at shallow phylogenetic levels, so, it is very difficult to delimit species’ boundaries and consequently to describe a new species based on morphological aspects (Hamilton et al. 2014, Hendrixson et al. 2015). This is even moreso relevant when there are few specimens available to analyze, which is the case of most mygalomorph species. It is common to decide not to describe a new species because there is no sufficient morphological evidences to do so. Thus, it is not a surprise when we note many new species of a previously morphologically analyzed taxon being detected when using molecular approaches (Hedin and Carlson 2011; Hendrixson and Bond 2005; Starret and Hedin 2007). A good example of a cryptic biodiversity discovery was by Hamilton et al. (2011), when they studied *Aphonopelma* genus. It is a widespread and specious taxon that has received little attention and “has been largely reliant upon sparse and sometimes poorly defined morphological data” (Hamilton et al. 2011). *Avicularia* seems to be exactly the same case. *Avicularia* spermathecae, male palpal bulb, and tibial apophysis shape as well as somatic characters (except coloration traits) are very similar among different populations. Thus, as occurring with *Aphonopelma* (Hendrixson et al. 2015), it is very probable that we can only access the real *Avicularia* diversity when we use multiple approaches for an accurate definition of species boundaries.

Morphologically cryptic species are an increasingly recurrent problem on traditional zoological taxonomy (Satler et al. 2013). Here we detected that boundaries of many *Avicularia* species, especially *Avicularia avicularia*, could not be delimited using the current morphological tools and data. It is necessary to find new morphological characters and, combined with molecular, geographic and ecological data, to do a more extensive and integrative analysis of *Avicularia*.

An accurate assessment of species-level diversity is essential not only to specialists; it plays an important role in studies of ecology and biodiversity and consequently in conservation decisions and policy (Hendrixson and Bond 2005; Hamilton et al. 2011) as well as in the suitable species’ exploration for developing new technologies, materials, and ideas to confront environmental, medical, and engineering challenges (Wheeler et al. 2012). A more precise taxonomy and the proposal of a strongest phylogenetic hypothesis especially for theraphosid spiders are very urgent since constant habitat destruction and high rate of pet trade are pressing problems for spiders populations (Bond et al. 2005; Hamilton et al. 2011).

## Supplementary Material

XML Treatment for Aviculariinae

XML Treatment for Avicularia

XML Treatment for Avicularia avicularia

XML Treatment for Avicularia glauca

XML Treatment for Avicularia variegata

XML Treatment for Avicularia minatrix

XML Treatment for Avicularia taunayi

XML Treatment for Avicularia juruensis

XML Treatment for Avicularia rufa

XML Treatment for Avicularia purpurea

XML Treatment for Avicularia hirschii

XML Treatment for Avicularia merianae

XML Treatment for Avicularia lynnae

XML Treatment for Avicularia caei

XML Treatment for Avicularia vestiaria

XML Treatment for Iridopelma leporina

XML Treatment for Iridopelma plantaris

XML Treatment for Euathlus affinis

XML Treatment for Ischnocolus hirsutum

XML Treatment for Avicularia metallica

XML Treatment for Ischnocolus gracilis

XML Treatment for Grammostola subvulpina

XML Treatment for Avicularia arabica

XML Treatment for Thrixopelma aymara

XML Treatment for Avicularia aurantiaca

XML Treatment for Araneus hirtipes

XML Treatment for Avicularia testacea

XML Treatment for Avicularia detrita

XML Treatment for Avicularia hirsutissima

XML Treatment for Avicularia holmbergi

XML Treatment for Ischnocolus doleschalli

XML Treatment for Avicularia rapax

XML Treatment for Avicularia ochracea

XML Treatment for Avicularia walckenaerii

XML Treatment for Avicularia azuraklaasi

XML Treatment for Avicularia braunshauseni

XML Treatment for Avicularia geroldi

XML Treatment for Avicularia huriana

XML Treatment for Avicularia ulrichea

XML Treatment for Avicularia soratae

XML Treatment for Avicularia fasciculata

XML Treatment for Avicularia fasciculata clara

XML Treatment for Avicularia surinamensis

XML Treatment for Caribena

XML Treatment for Caribena laeta

XML Treatment for Caribena versicolor

XML Treatment for Ybyrapora

XML Treatment for Ybyrapora sooretama

XML Treatment for Ybyrapora gamba

XML Treatment for Ybyrapora diversipes

XML Treatment for Antillena

## Supplementary materials

Supplementary material 1 Tree obtained with Piwe, all characters as non-additive and concavity 1 Data type: molecular data This dataset is made available under the Open Database License (http://opendatacommons.org/licenses/odbl/1.0/). The Open Database License (ODbL) is a license agreement intended to allow users to freely share, modify, and use this Dataset while maintaining this same freedom for others, provided that the original source and author(s) are credited.Caroline Sayuri Fukushima, Rogério Bertani

Supplementary material 2 Tree obtained with Piwe, all characters as non-additive and concavity 2 Data type: molecular data This dataset is made available under the Open Database License (http://opendatacommons.org/licenses/odbl/1.0/). The Open Database License (ODbL) is a license agreement intended to allow users to freely share, modify, and use this Dataset while maintaining this same freedom for others, provided that the original source and author(s) are credited.Caroline Sayuri Fukushima, Rogério Bertani

Supplementary material 3 Tree obtained with Piwe, all characters as non-additive and concavity 3 Data type: molecular data This dataset is made available under the Open Database License (http://opendatacommons.org/licenses/odbl/1.0/). The Open Database License (ODbL) is a license agreement intended to allow users to freely share, modify, and use this Dataset while maintaining this same freedom for others, provided that the original source and author(s) are credited.Caroline Sayuri Fukushima, Rogério Bertani

Supplementary material 4 Tree obtained with Piwe, all characters as non-additive and concavity 4 Data type: molecular data This dataset is made available under the Open Database License (http://opendatacommons.org/licenses/odbl/1.0/). The Open Database License (ODbL) is a license agreement intended to allow users to freely share, modify, and use this Dataset while maintaining this same freedom for others, provided that the original source and author(s) are credited.Caroline Sayuri Fukushima, Rogério Bertani

Supplementary material 5 Tree obtained with Piwe, all characters as non-additive and concavity 5 Data type: molecular data This dataset is made available under the Open Database License (http://opendatacommons.org/licenses/odbl/1.0/). The Open Database License (ODbL) is a license agreement intended to allow users to freely share, modify, and use this Dataset while maintaining this same freedom for others, provided that the original source and author(s) are credited.Caroline Sayuri Fukushima, Rogério Bertani
