# Supplementary figures and images for: Taxonomic revision and cladistic analysis of Avicularia Lamarck, 1818 (Araneae, Theraphosidae, Aviculariinae) with description of three new aviculariine genera
Source: Zookeys. 2017 Mar 2;(659):1–185. doi: 10.3897/zookeys.659.10717 (PMC5345366; doi:10.3897/zookeys.659.10717)

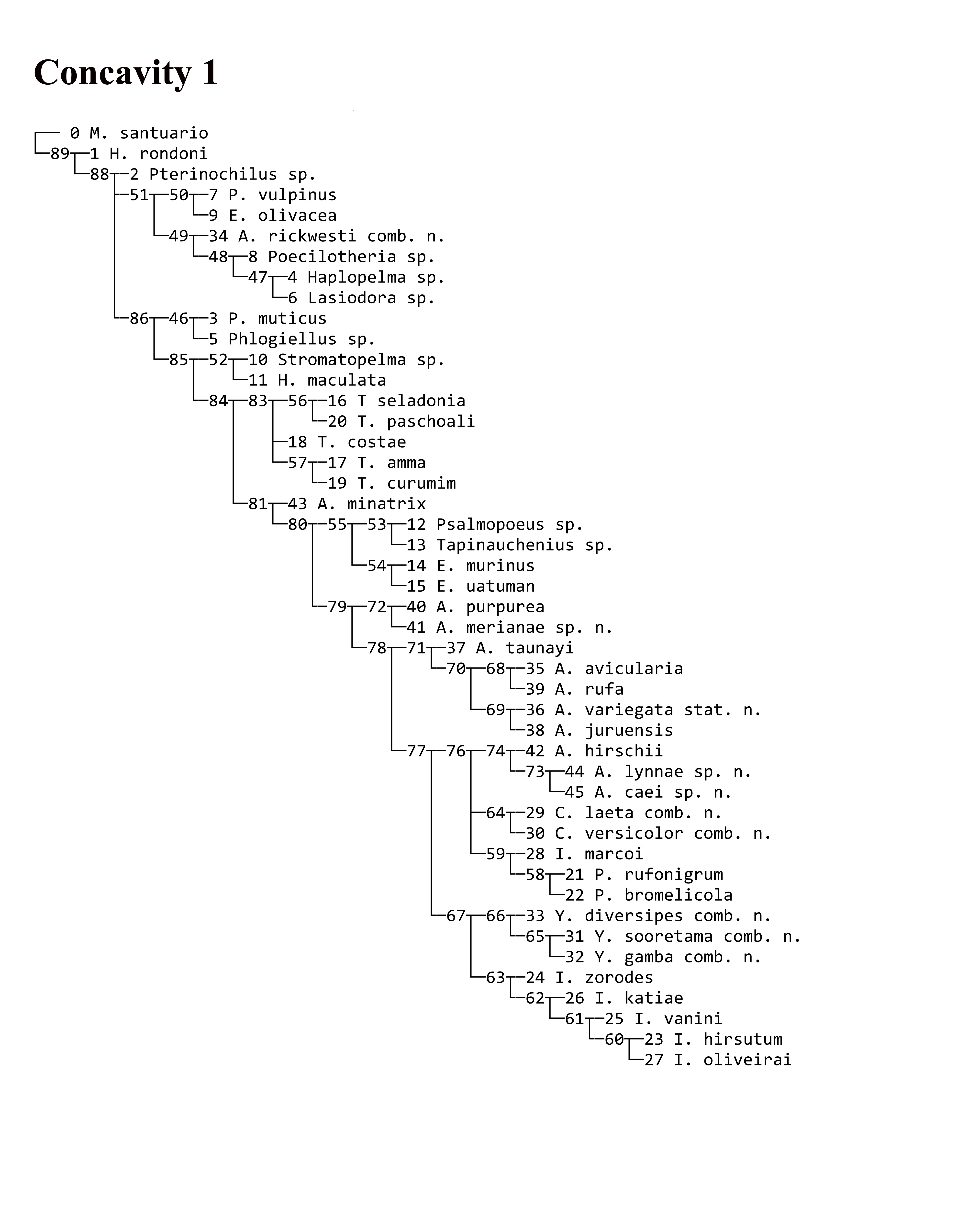

Supplement: Supplementary material 1 — Tree obtained with Piwe, all characters as non-additive and concavity 1 [file zookeys-659-001-s001.jpg]

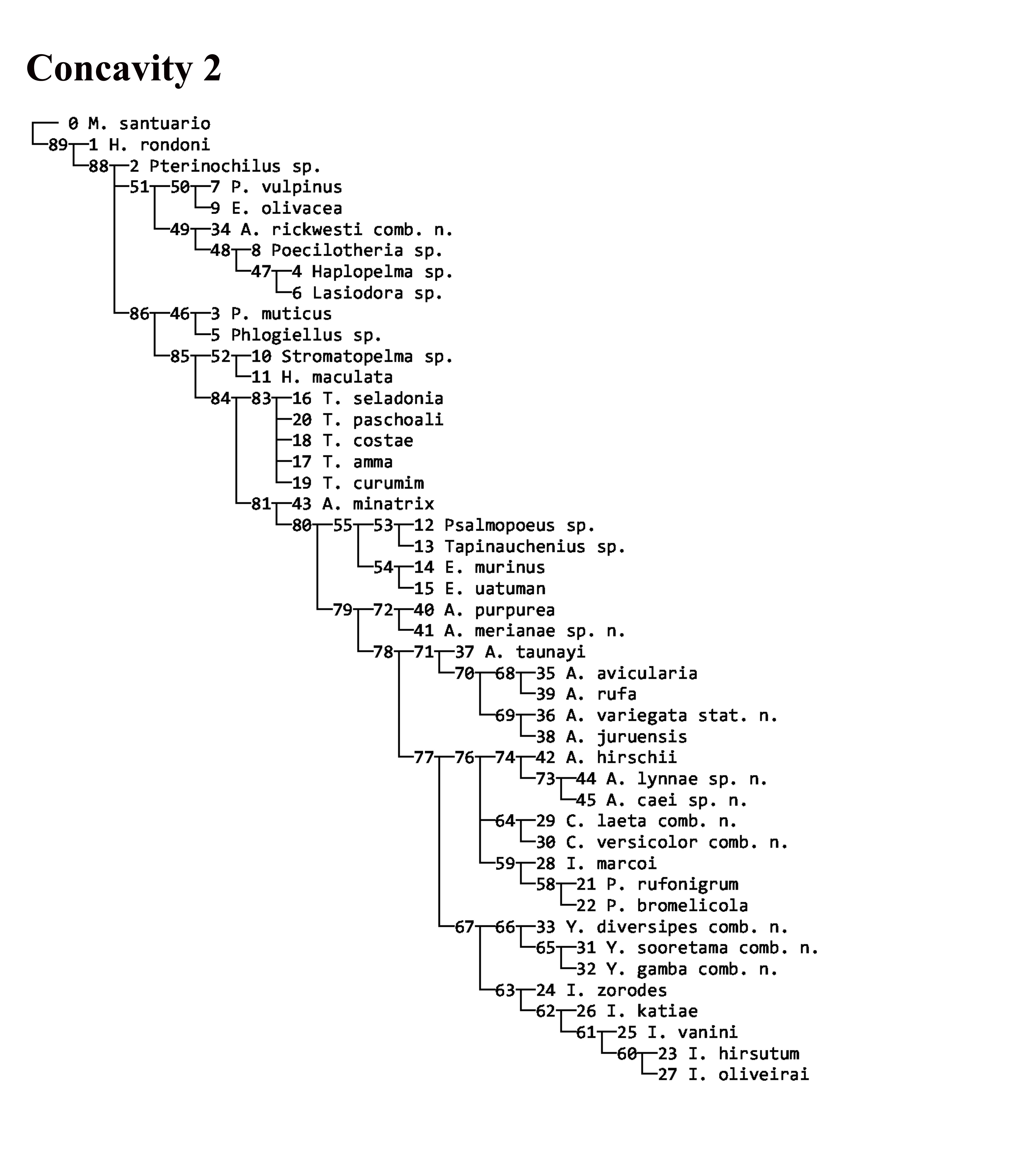

Supplement: Supplementary material 2 — Tree obtained with Piwe, all characters as non-additive and concavity 2 [file zookeys-659-001-s002.jpg]

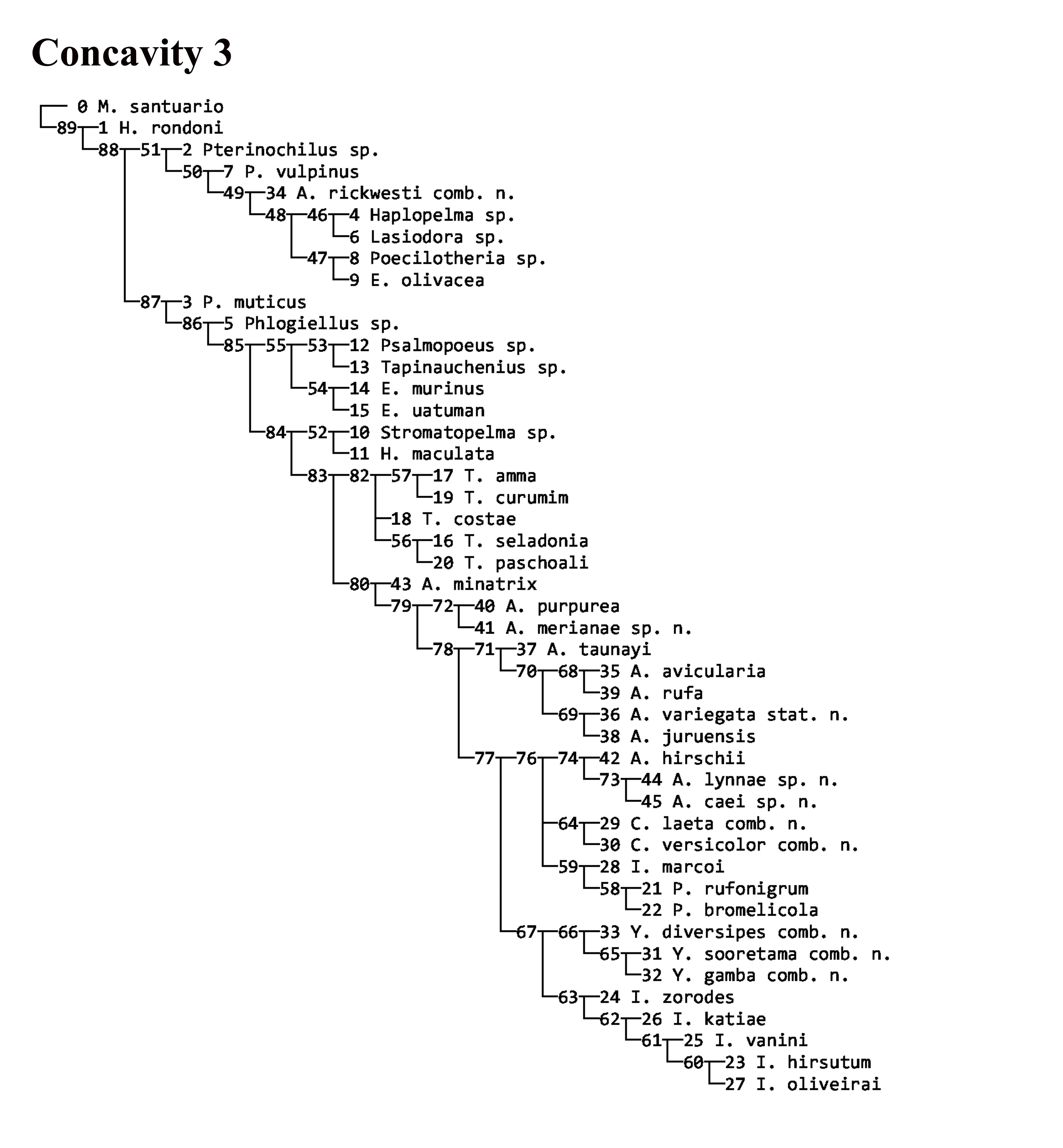

Supplement: Supplementary material 3 — Tree obtained with Piwe, all characters as non-additive and concavity 3 [file zookeys-659-001-s003.jpg]

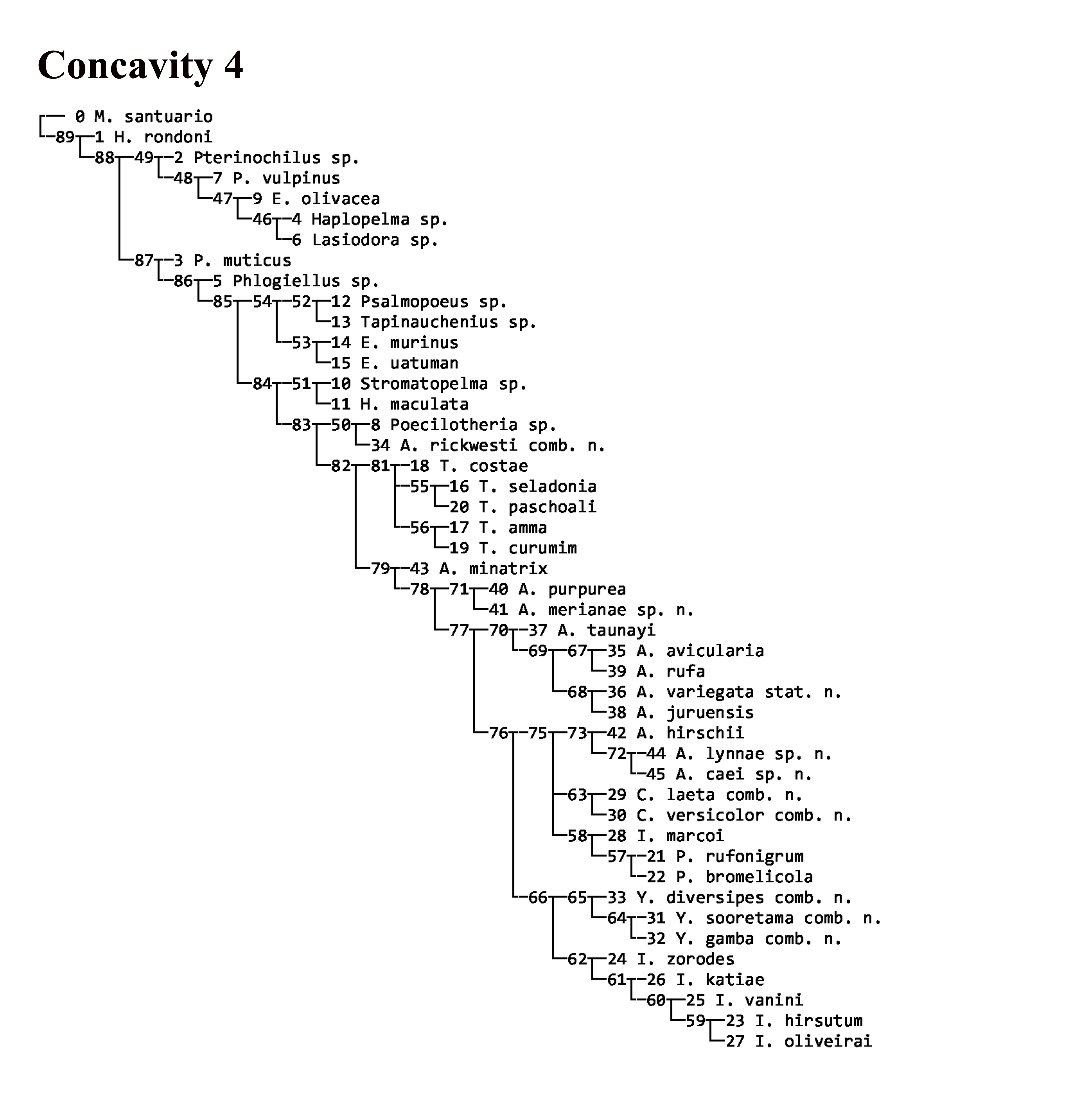

Supplement: Supplementary material 4 — Tree obtained with Piwe, all characters as non-additive and concavity 4 [file zookeys-659-001-s004.jpg]

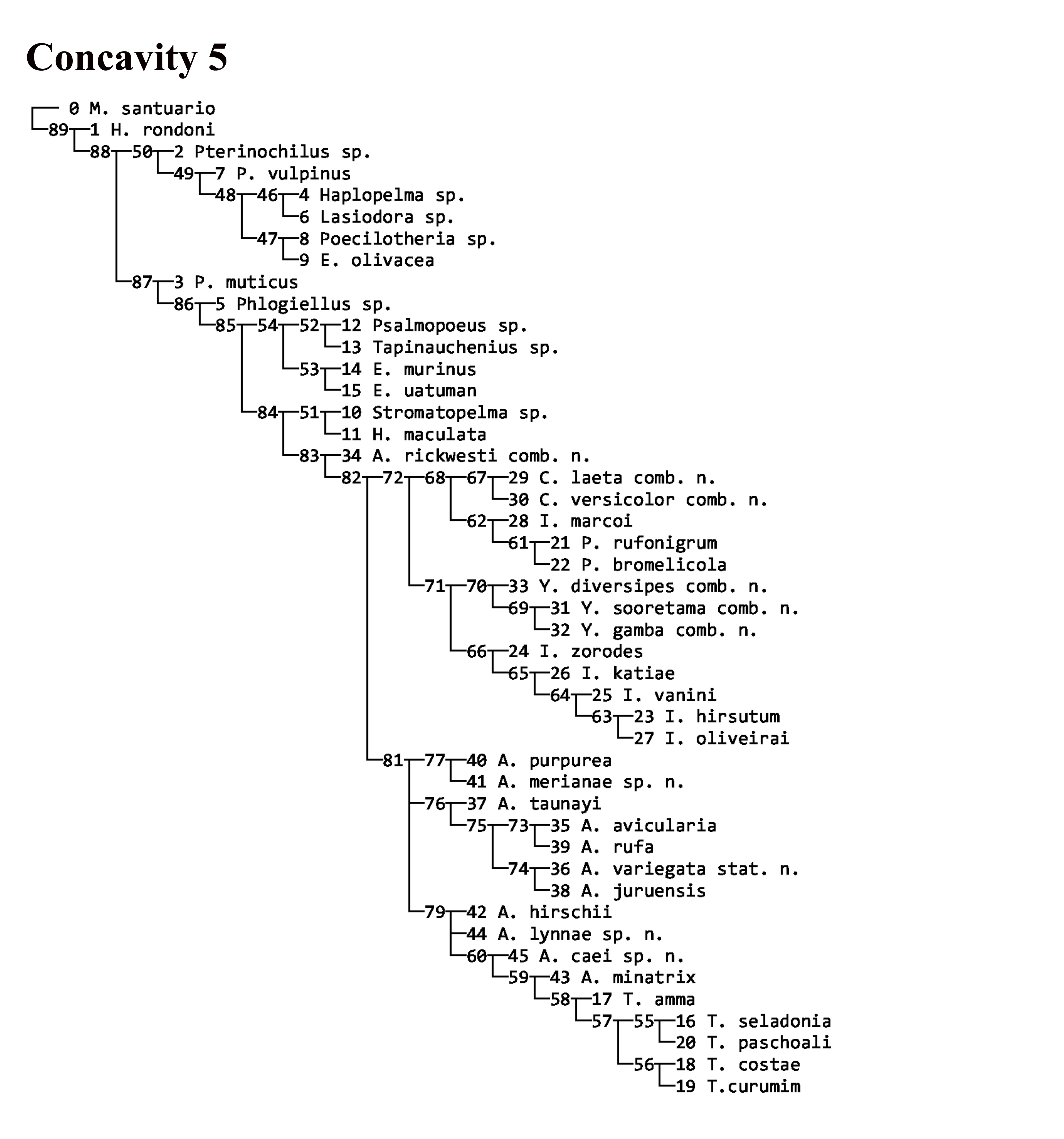

Supplement: Supplementary material 5 — Tree obtained with Piwe, all characters as non-additive and concavity 5 [file zookeys-659-001-s005.jpg]
